# Catalytic hydroboration of aldehydes with electron-rich alkoxysilylenes: from mechanistic insights to highly active catalysts

**DOI:** 10.1039/d6sc02376b

**Published:** 2026-07-15

**Authors:** Leon Kapp, Brian Schmetten, Christoph Wölper, Gebhard Haberhauer, Stephan Schulz

**Affiliations:** a Institute for Inorganic Chemistry, University of Duisburg-Essen Universitätsstraße 5-7 45117 Essen Germany stephan.schulz@uni-due.de; b Institute for Organic Chemistry, University of Duisburg-Essen Universitätsstraße 5-7 45117 Essen Germany; c Center for Nanointegration Duisburg-Essen (Cenide), University of Duisburg-Essen Carl-Benz-Str. 199 47057 Duisburg Germany

## Abstract

Main group metal complexes receive increasing interest due to their potential technical application in catalytic reactions. Here we report the synthesis and characterisation of a series of alkoxysilylenes L^1^(Cl)GaCH(R)OSiL^2^ (L^1^ = HC[C(Me)NDipp]_2_, Dipp = 2,6-^*i*^Pr_2_C_6_H_3_, L^2^ = PhC(N^*t*^Bu)_2_; R = Et 3, Ph 4, 3-Br-C_6_H_4_5, 4-hexyl-C_6_H_4_6), whose steric and electronic properties were systematically varied, and their high activity in the catalytic hydroboration of aldehydes. This comparative study showed excellent TOF values of up to 98 000 h^−1^ at 25 °C with a very low catalyst loading of only 0.001 mol%. Mechanistic studies verified the catalytic reaction mechanism, which was further proven by the isolation and structural characterisation (sc-XRD) of oxasilirane L^2^Si[OC(H)C_10_H_15_]OC(H)C_6_H_6_Ga(Cl)L^1^ (7), the key reaction intermediate formed *via* [2 + 1] cycloaddition of alkoxysilylene 4 with 1-adamantanecarboxaldehyde at −30 °C. Quantum chemical calculations proved that the catalytic activity of the alkoxysilylenes L^1^(Cl)GaCH(R)OSiL^2^ is strongly affected by the steric demand of the alkoxy unit (R). In addition, the electronic nature of the aldehyde was found to control the rate-limiting step.

## Abstract 2

Alkoxysilylenes effectively catalyse the hydroboration of aldehydes with HBPin at room temperature, exhibiting TOF values of up to 98 000 h^−1^, *via* initial formation of an alkoxyoxasilirane, the active species as proven by mechanistic studies.

## Graphical abstract

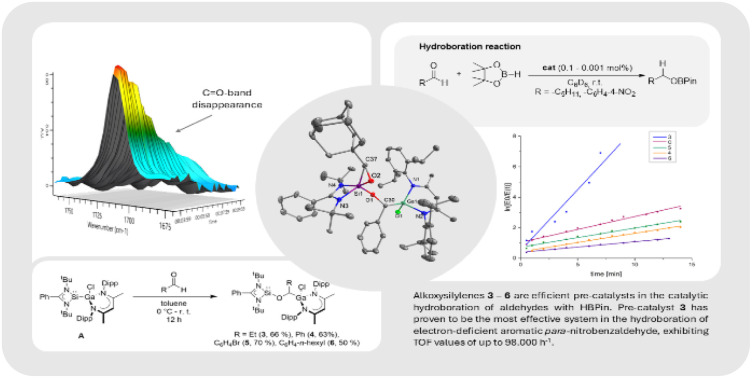

## Introduction

**Chart 1:**
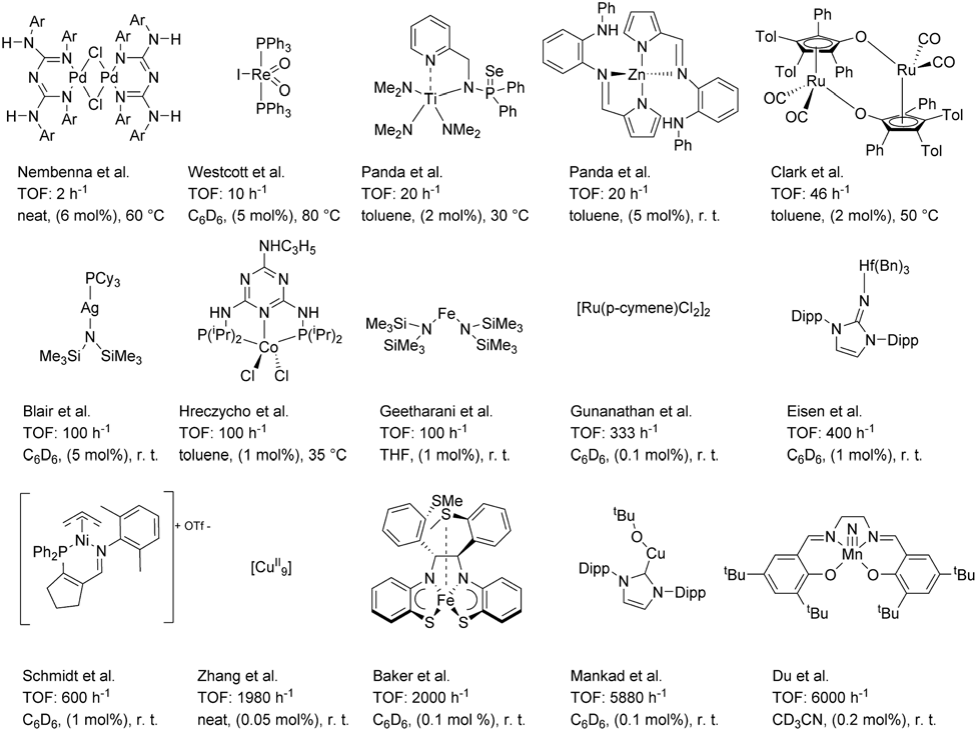
Selected transition metal complexes based on their highest performances (calculated TOF values) in the catalytic hydroboration of aldehydes.^14–28^

Hydroboration has become a cornerstone transformation in organic synthesis since its introduction by H. C. Brown in 1956.^1^ However, the exceptionally high reactivity and limited chemoselectivity of initially used borane (BH_3_) forced the development of user-friendly hydroboration agents, *e.g*., pinacolborane (HBPin) and catecholborane (HBcat).^2^ HBPin and HBcat are much easier to handle and show a higher functional group tolerance compared to BH_3_. Since their activity in hydroboration is rather low, catalytic activation is typically required. The synthesis of a Rh(iii)hydridoboryl complex, which was formed by oxidative addition of HBcat to the Wilkinson catalyst, [RhCl(PPh_3_)_3_], represented a key breakthrough in this research area.^3^ This rhodium(iii) complex enabled the first catalytic hydroboration of a terminal olefin, reported by Nöth *et al.* in 1985.^4^ Since then, many transition metal complexes with different metal centres and coordination environments have been reported to catalyse the hydroboration of unsaturated organic substrates, in particular aldehydes, under different reaction conditions.^5–28^ These complexes also showed different reaction mechanisms, offering diverse approaches to fine-tune their reactivity and selectivity. To enable a more consistent and meaningful comparison, calculated turnover frequencies (TOFs) for the catalytic hydroboration of aldehydes by selected transition-metal-based catalysts are summarised in Chart 1. The reaction temperature, solvent and catalyst loading are explicitly annotated. The calculated TOF values reported for transition metal-based catalysts span several orders of magnitude, ranging from low activities of about 2 h^−1^ to highly active systems exceeding 6000 h^−1^. Highest efficiencies were reported for manganese, copper, and iron complexes, while late transition metal complexes, including ruthenium and palladium, only showed moderate catalytic performances.

**Chart 2:**
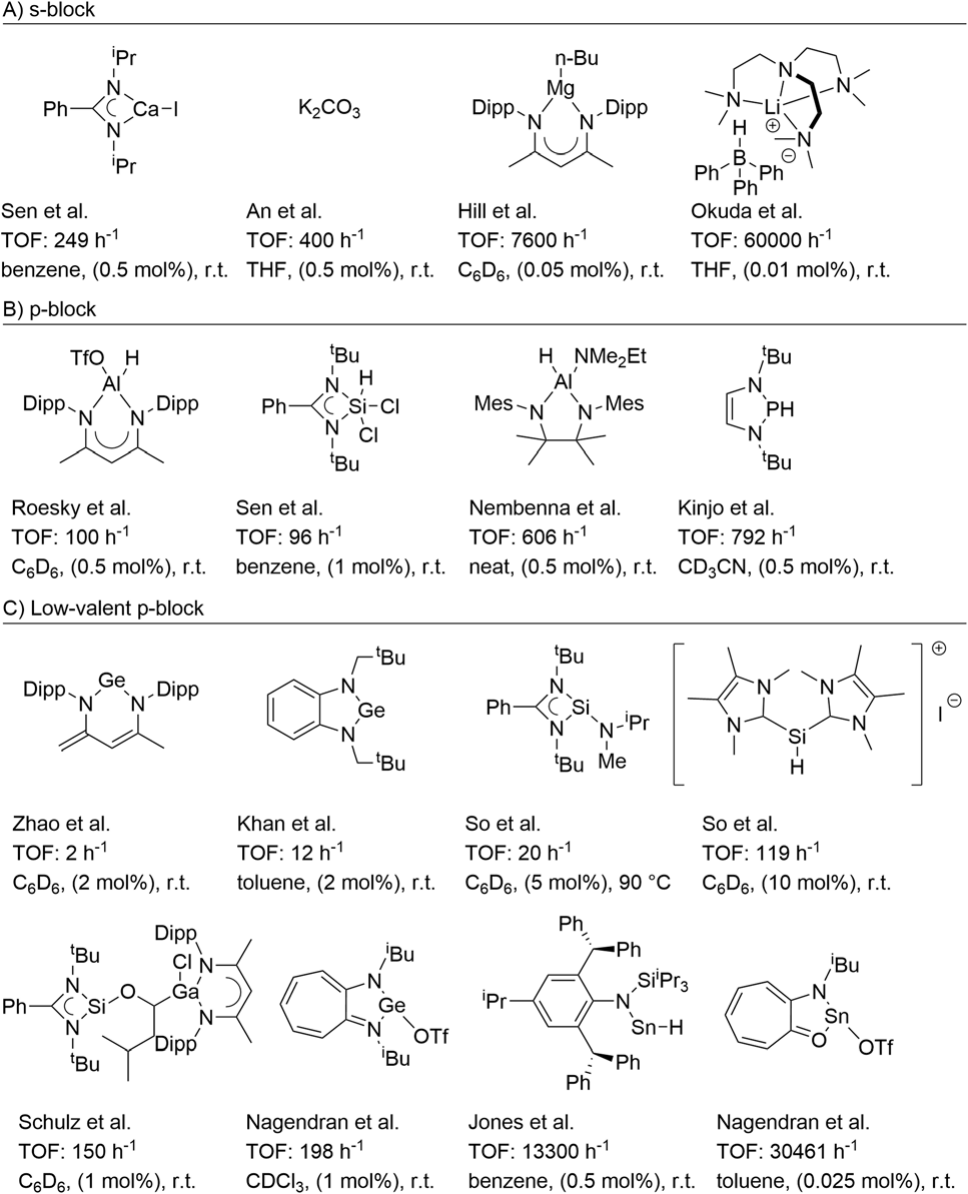
Highest activities of s- and p-block element complexes in the catalytic hydroboration of aldehydes ranked by the calculated TOF values.^48–61,79^

In addition to d-block metal catalysts, an increasing number of hydroboration catalysts based on s-block^29–51^ and p-block^52–78^ elements have been reported in recent years (Chart 2). Among the s-block metal catalysts, Ca and K complexes showed moderate TOF values (249 h^−1^, 400 h^−1^),^48,49^ whereas significantly higher activities were observed for Mg (7600 h^−1^)^50^ and Li complexes (60 000 h^−1^),^51^ which showed the highest TOF value with the lowest catalyst loading (0.01 mol%) in the hydroboration of aldehydes, reported to date.^51^ In contrast, p-block element complexes containing group 13 (Al: 100 h^−1^, 606 h^−1^),^53,54^ group 14 (Si: 96 h^−1^),^52^ and group 15 elements (P: 792 h^−1^)^55^ only showed moderate catalytic activities.

**Chart 3:**
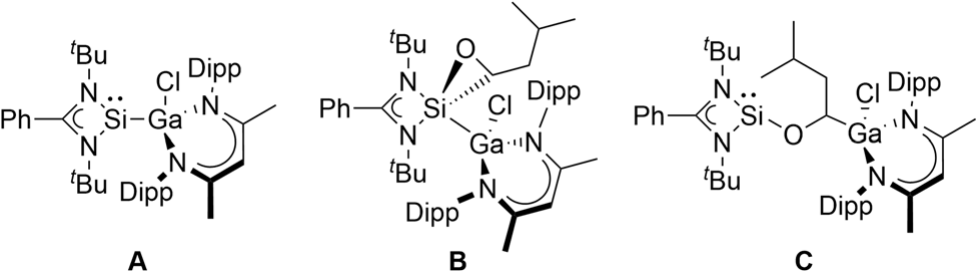
Compounds A, B and C reported in the previous work.^60^

Low-valent p-block compounds such as divalent tetrylenes received increasing interest in recent years in small molecule activation reactions including catalytic reactions due to their Lewis ambiphilic behaviour, resulting from the presence of a vacant p-orbital and an electron lone pair.^78,80–85^ They showed TOF values in the catalytic hydroboration of aldehydes ranging from 2 h^−1^ up to 30 461 h^−1^.^56,63^ Stannylenes often exhibited very high activities, as was initially reported by Jones *et al.* for the acyclic amido(hydrido)stannylene [Ar*(^*i*^Pr_3_Si)NSnH] (Ar* = 4-^*i*^Pr-2,6-(CHPh_2_)_2_C_6_H_2_), which gave excellent TOF values of up to 13 300 h^−1^ with a low catalyst loading of only 0.5 mol%.^62^ Nagendran *et al.* reported an even higher catalytic activity of a cyclic stannylene (TOF 30 641 h^−1^),^63^ whereas lighter tetrylenes (Si, Ge) typically showed much lower activities. To the best of our knowledge, only three silylenes have been reported to be active catalysts in the hydroboration of aldehydes. So *et al.* reported TOF values of 20 h^−1^ and 119 h^−1^ for L^2^SiN(Me)^*i*^Pr and the NHC-coordinated silyliumylidene cation [(IMe)_2_SiH]^+^ (IMe: C{N(Me)C(Me)}_2_), which both required harsh reaction conditions (90 °C, catalyst loading 10 mol%),^58,59^ whereas we recently demonstrated the promising catalytic activity of alkoxysilylene L^1^(Cl)GaCH(CH_2_CHMe_2_)OSiL^2^C (L^1^ = HC[C(Me)NDipp]_2_, Dipp = 2,6-^*i*^Pr_2_C_6_H_3_), which was synthesised by reaction of gallasilylene L^1^(Cl)GaSiL^2^A with *iso*-valeraldehyde *via* intermediate formation of oxasilirane L^2^Si[OC(H)CH_2_CHMe_2_]Ga(Cl)L^1^B (Chart 3).^60^

**Scheme 1:**
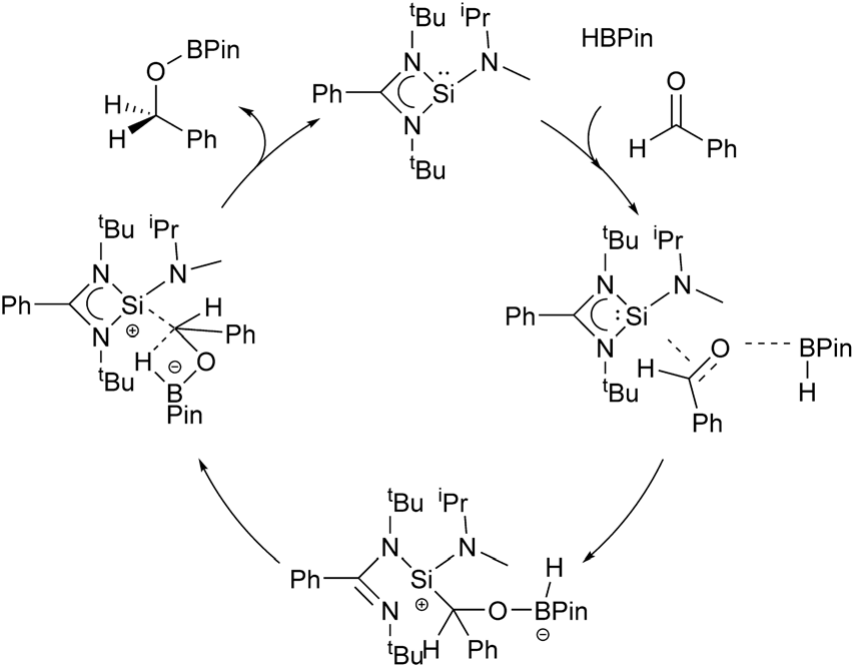
Calculated reaction mechanism of the catalytic hydroboration of benzaldehyde with HBPin by So *et al.*^58^

Alkoxysilylene C showed TOF values up to 150 h^−1^ under much milder reaction conditions (25 °C, 1 mol% catalyst loading). Two possible reaction mechanism based on the initially formed alkoxysilylene C were identified by quantum chemical calculations, which both showed the hydride transfer either from a silane (Si–H) or from the coordinated borane (B–H) as the rate-determining step with similar activation barriers (27.3 kcal mol^−1^*vs.* 26.7 kcal mol^−1^; see also Scheme 4). Unfortunately, the real mechanism could not be identified at that time since all attempts to spectroscopically identify or isolate the key reaction intermediate formed in these reactions failed. In contrast, So *et al.* reported a catalytic hydroboration mechanism for silylene L^2^SiN(Me)^*i*^Pr based on DFT calculation (Scheme 1).^59^

The silylene initially coordinated to the electrophilic carbonyl C atom of benzaldehyde, while the nucleophilic O atom simultaneously interacted with the vacant p orbital of HBPin, generating a zwitterionic intermediate. Hydride transfer from HBPin to the carbonyl C atom *via* formation of a four-membered transition state finally produced the borate ester (RCH_2_OBPin) with regeneration of the silylene (Scheme 1). The silane L^2^(H)Si was further proven to be catalytically inactive in the hydroboration reactions since no reactions were observed with benzaldehyde and/or HBPin even at 90 °C.^59^

Here we report the catalytic activity of a series of alkoxysilylenes L^1^(Cl)GaCH(R)OSiL^2^ (R = Et 3, Ph 4, 3-Br-C_6_H_4_5, 4-hexyl-C_6_H_4_6) with different steric and electronic properties in the hydroboration of aldehydes with HBPin. 3–6 were synthesised by equimolar reactions of gallasilylene L^1^(Cl)GaSiL^2^A with the respective aldehyde, which proceeded with intermediate formation of the corresponding oxasiliranes *via* [2 + 1] cycloaddition at the divalent Si atom as was proven by the isolation and structural characterisation of oxasiliranes 1 and 2. Detailed kinetic studies with *n*-hexanal and *para*-nitrobenzaldehyde provided deeper insight into the role of the inserted aldehyde on the alkoxysilylene's catalytic activity, and excellent TOF values of up to 98 000 h^−1^ were obtained for the hydroboration of *para*-nitrobenzaldehyde using alkoxysilylene 3. In addition, the catalytic reaction mechanism was finally identified *via* isolation (oxasilirane 7) and spectroscopic characterisation (silane 8a) of two reaction intermediates, which formed in the reaction of 4 with the sterically demanding 1-adamantanecarboxaldehyde. Moreover, the electronic nature of the aldehyde was found to control the rate-limiting step of the catalytic hydroboration reaction.

## Results and discussion

### Synthesis and spectroscopic characterisation

**Scheme 2:**
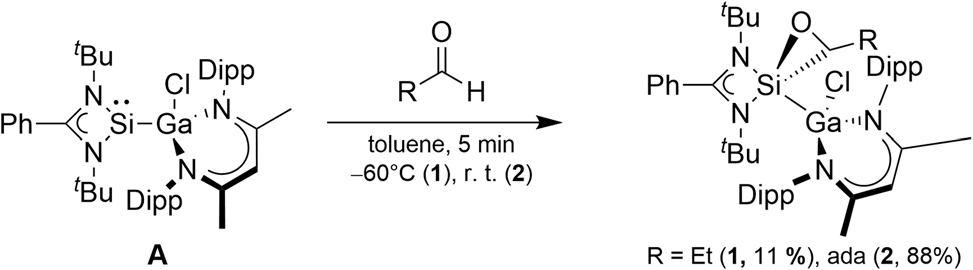
Syntheses of oxasiliranes 1 and 2.

**Scheme 3:**
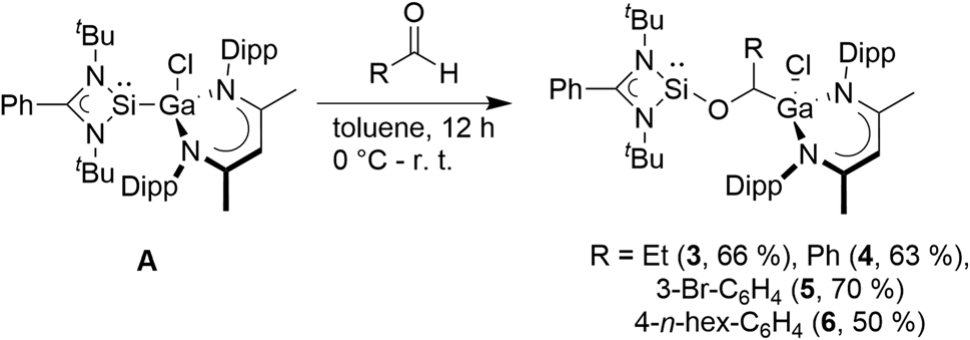
Reactions of gallasilylene A with different aldehydes to alkoxysilylenes 3–6.

The promising activity of alkoxysilylene C in the hydroboration of aldehydes and ketones^60^ prompted our interest to the role of the alkoxy group on the catalytic activity of the subsequently formed alkoxysilylene. We therefore studied reactions of gallasilylene A with sterically less hindered *n*-propanal to decrease the steric demand in proximity to the reactive centre and with sterically demanding 1-adamantanecarboxaldehyde (Scheme 2). Both reactions proceeded with formation of the oxasiliranes 1 and 2. However, while oxasilirane 1, which was spectroscopically identified at very low temperature (−60 °C), fully converts at 25 °C within 12 h to alkoxysilylene 3 (Scheme 3) as was proven by *in situ*^1^H NMR spectroscopy, oxasilirane 2 is even stable at 80 °C and showed no tendency to convert into the alkoxysilylene. 2 can also be exposed to air for hours without decomposition.

^1^H and ^13^C NMR spectra of 1 and 2 show the expected resonances of the ligands (L^1^, L^2^), the aldehyde H atom (1: 1.91 ppm, 2: 1.20 ppm) and the carbonyl C atom (1: 63.15 ppm, 2: 70.02 ppm). The ^29^Si NMR spectra show resonances at −112.16 ppm (1) and −110.03 ppm (2), in line with values of known oxasiliranes (−99 to −123 ppm).^60,86–92^

To further expand the number of alkoxysilylenes for the catalytic studies and to investigate the electronic properties of the inserted aldehyde, we reacted gallasilylene A with benzaldehyde, 3-bromo- and 4-*n*-hexyl-benzaldehyde at 25 °C, yielding alkoxysilylenes 4–6 in good yields (Scheme 3). The NMR spectra of alkoxysilylenes 3–6 exhibit the expected resonances including the aldehyde H atom in the ^1^H NMR (3: 4.13 ppm, 4: 5.14 ppm, 5: 5.10 ppm, 6: 5.14 ppm) and the carbonyl C atom in the ^13^C NMR spectra (3: 73.90 ppm, 4: 76.10 ppm, 5: 75.38 ppm, 6: 76.13 ppm). The ^29^Si NMR resonances (3: −16.98 ppm, 4: −14.09 ppm, 5: −13.35 ppm, 6: −13.70 ppm) agree well with chemical shifts reported for alkoxysilylenes and siloxysilylenes.^92,93^

### Mechanistic studies

**Scheme 4:**
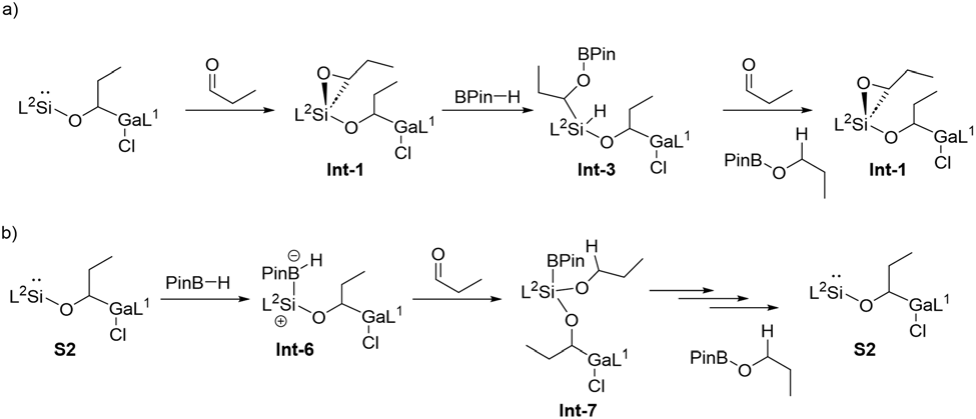
Simplified mechanism for the hydroboration of *n*-propanal with Int-2 (a) and S2 (b) as catalytically active species calculated by means of PBE0-D3BJ.^59^

Mechanistic studies with tetrylene complexes in the catalytic hydroboration of carbonyl compounds based on the isolation and characterisation of real reaction intermediates are rare. Hydridogermylene [Ar*(^*i*^Pr_3_Si)NGeH] was found to react *via* Ge–H and B–H σ-bond metathesis,^62^ whereas cyclic intramolecularly donor-stabilised tetrylenes [Ar^*i*Pr^EC(H)(Ph)PPh_2_] (E = Ge, Sn; Ar^*i*Pr^ = 2,6-(2,4,6-^*i*^Pr_3_C_6_H_2_)_2_C_6_H_3_) catalysed the hydroboration of aldehydes *via* initial aldehyde insertion into the dative phosphine-tetrylene bond. The stannylene also activated pinacolborane (HBPin) to give a Sn(ii) hydride, which acted as a catalyst in the hydroboration reaction.^71^*In situ* generated hydrostannylenes reacted with carbonyl compounds to alkoxystannylenes,^62,70,74^ which then reacted with HBPin to the corresponding boronic ester. In addition, a diaminogermylene-mediated catalytic hydroboration of aldehydes was reported to occur *via* adduct formation between HBPin and the Lewis acidic Ge(ii) centre, which facilitated the insertion of the aldehyde into the B–H bond.^56^ In contrast, alkoxysilylene C did not react with HBPin, and we hence assumed that the catalytic cycle started with the reaction between C and the aldehyde.^60^ Quantum chemical calculations identified two energetically almost identical reaction pathways (Scheme 4; full pathway see Fig. S74), but all attempts to isolate or spectroscopically identify any of the reaction intermediates failed.

**Scheme 5:**
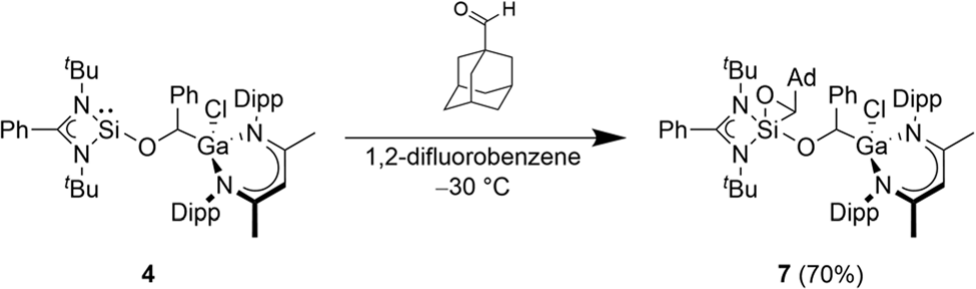
Reaction of alkoxysilylene 4 with 1-adamantanecarboxaldehyde to 7.

With alkoxysilylenes 3–6 with different steric and electronic properties in hand, we re-investigated the reactions and isolated oxasilirane L^2^Si[OC(H)C_10_H_15_]OC(H)C_6_H_6_Ga(Cl)L^1^ (7), which represents Int-1 in the catalytic cycle, from the [2 + 1] cycloaddition of alkoxysilylene 4 with bulky 1-adamantanecarboxaldehyde in 1,2-difluorobenzene at −30 °C (Scheme 5). Two diastereomers of oxasilirane 7 (63%, 37%), resulting from the presence of a stereo centre, were distinguished in the NMR spectra. They show the expected signals of the alkoxysilylene (^1^H NMR: 7a: 5.69 ppm, 7b: 5.48 ppm; ^13^C NMR: 7a: 72.36 ppm, 7b: 73.86 ppm) and the oxasilirane units (^1^H NMR: 7a: 2.16 ppm, 7b: 1.92 ppm and ^13^C NMR: 7a: 75.62 ppm, 7b: 76.63 ppm).

**Scheme 6:**
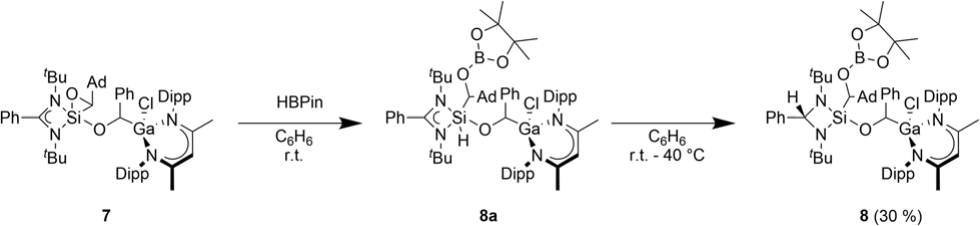
Reaction of L^2^Si[OCHC_10_H_15_]OCH[Ga(Cl)L^1^]C_6_H_6_ (7) with HBPin.

Oxasilirane 7 was then reacted with an equimolar amount of HBPin to prove the formation of silane 8a (Int-3).^60^ The reaction occurred only slowly at 25 °C and was therefore heated to 40 °C for 24 hours, resulting in the formation of compound 8, which was isolated as colourless crystalline solid in 30% yield (Scheme 6).

Compound 8 is formed by a subsequent hydride shift from the silicon atom of the proposed silane 8a to the amidinate ligand backbone (Scheme 6), likely initiated by the high reaction temperature. *In situ*^1^H NMR spectroscopy studies of the reaction of 7 with HBPin at 25 °C (Fig. S35) proved the formation of silane 8a due to the occurrence of two signals of the Si–H unit (3.87, 3.86 ppm) of diastereotopic 8a. The *in situ*^29^Si{H}DEPT NMR spectrum shows a signal at −64.6 ppm (Fig. S37), which is shifted compared to that of isolated 8 (−34.1 ppm, Fig. S33).

So *et al.* synthesised L^2^(H)SiC(H)PhO(BO_2_C_6_H_12_)(CON(Me)^*i*^Pr, which can be regarded as analogue of 8a, but this silane failed to catalyse the hydroboration of benzaldehyde with HBPin, excluding a possible hydride transfer from Si to the aldehyde.^59^ Instead, a hydride transfer from HBPin to the electrophilic carbonyl C atom occurred. We studied reactions of compound 8 (1 mol%) with HBPin and benzaldehyde but did not observe any reaction (Fig. S39), hence excluding its participation in the catalytic hydroboration mechanism. In contrast, a solution of *in situ* (25 °C) generated silane 8a readily reacted with four equivalents of HBPin and five equivalents of 1-adamantanecarboxaldehyde. After 15 minutes, the *in situ*^1^H NMR spectrum showed two diastereomeric resonances at 3.75 and 3.97 ppm (Fig. S37) in the Si–H region, which differ from those of silane 8a and probably stem from an aldehyde-coordinated species formed during the reaction. The spectrum also shows a resonance at 3.60 ppm, proving the borane ester formation (40% conversion after 2 h, Fig. S38), whereas the formation of 8 was not observed in this study, proving that the hydride shift from 8a to the aldehyde is faster than to the amidinate backbone (8).

**Scheme 7:**
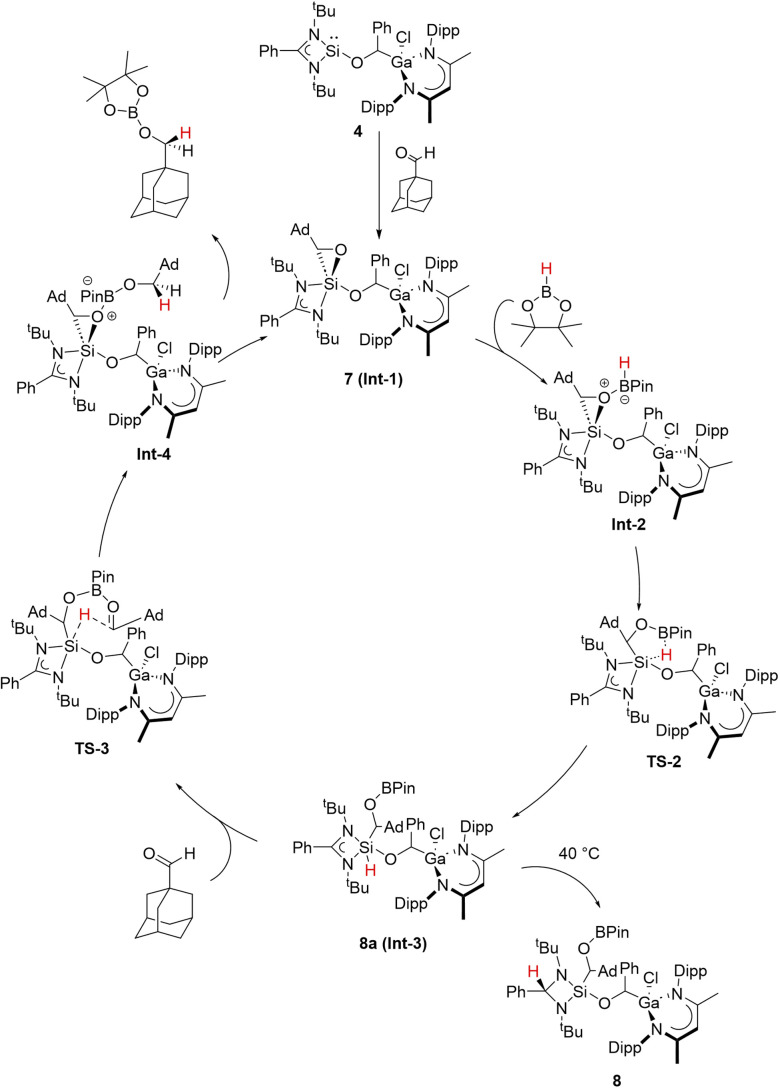
Experimentally confirmed reaction mechanism of the catalytic hydroboration of 1-adamantanecarboxaldehyde with HBPin with 4 as catalyst.

Scheme 7 displays the confirmed reaction mechanism of the catalytic hydroboration of 1-adamantanecarboxaldehyde with HBPin and alkoxysilylene 4, which is based on the isolation of oxasilirane 7, which represents the structural motif of Int-1 in our initially reported mechanism,^60^ and the spectroscopic identification of silane 8a. The alternative pathway *via* activation of the B–H unit can thus finally be excluded. The mechanism is comparable to transition metal-catalysed hydroboration reactions, which typically start with the formation of a metal hydride by reaction of the metal complex with HBPin, followed by insertion of the aldehyde into the metal–H bond.^94^ In our complexes, the Si atom in oxasilirane 7, formed by oxidative addition of the aldehyde to silylene 4, adopts the role of the transition metal, which activates HBPin with formation of silane 8a. 8a then transfers the hydride to an additional aldehyde, and the elimination of the borane ester leads to subsequent formation of oxasilirane 7 and closes the catalytic cycle.

### Crystallographic studies

**Fig. 1:**
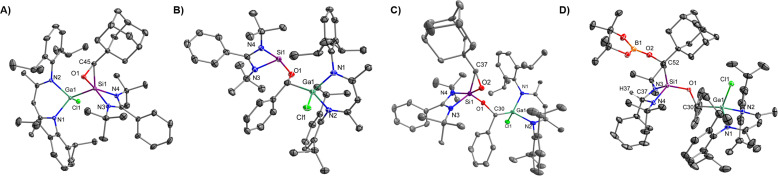
Structures of oxasilirane 2 (A), alkoxysilylene 4 (B), reaction intermediate 7 (C) and compound 8 (D) in the solid state with thermal ellipsoids at 50% probability level. Solvent molecules, hydrogen atoms and components of the minor diastereomer were omitted for clarity.

Crystals suitable for single crystal X-ray diffraction (sc-XRD) were obtained from saturated solutions in fluorobenzene (2 and 8) and 1,2-difluorobenzene (7) after storage at −18 °C or from saturated solutions in *n*-hexane (3 and 6), toluene (4) and benzene (5) after storage at 25 °C. Compounds 2 and 3 crystallise in the monoclinic space group *P*2_1_/*n* with one molecule (accompanied by one molecule of solvent (2)) in the asymmetric unit (Fig. 1). Alkoxysilylene 5 crystallises in the triclinic space group *P*1̄ with one molecule in the asymmetric unit, and compounds 4, 7 and 8 crystallise in the monoclinic space group *P*2_1_/*c* with two (4), one (7) and three (8) molecules in the asymmetric units. Selected bond lengths and bond angles of compounds 2–8 are summarised in Table 1. The SiN_2_C rings in 2–7 are almost planar, while the GaN_2_C_3_ rings adopt boat-type conformations. The Ga–Si bond length in oxasilirane 2 (2.4202(7) Å) is slightly elongated compared to oxasilirane B (2.4054(5) Å) but much shorter than in gallasilylene A (2.5170(4) Å).^60^ The Si–O (1.7092(11) Å) and Si–C (1.8393(12) Å) bond lengths in oxasilirane 2 differ from those of known oxasiliranes, which range from 1.6486(13) Å to 1.6520(10) Å (Si–O) and 1.8834(15) Å to 1.8924(18) Å (Si–C).^60,86–91^ The O–Si–C bond angle of 2 (49.46(5)°) is virtually identical with those of known oxasiliranes (49.52(11)°, 50.70(6)°, 50.31(6)°), while the Si–O–C bond angle of 2 (69.83(6)) is slightly smaller than those of other oxasiliranes (70.42–73.18°) but comparable to that of oxasilirane B (68.23(12)°).^60,86–91^ The Si–O bond length in alkoxysilylene 3 (1.669(3) Å) is in between those of alkoxysilylenes 4 (1.677(2) Å), 5 (1.6945(12) Å) and 6 (1.6830(10) Å) as well as the intermediates 7 (1.657(3) Å) and 8 (1.646(2) Å), respectively. The C–O bonds of the alkoxy unit in 3–8 (1.4325(19)–1.448(4) Å) are in the range of a typical C–O single bond (1.43 Å)^95,96^ and much shorter than the C–O bonds of the oxasilirane units in 2 (1.4891(13) Å) and 7 (1.520(8) Å). The Si–O–C bond angles in 7 (121.5(2)°) and 8 (125.37(18)°) are smaller than in 3–6 (3 129.5(2)°, 4 128.8(2)°, 5 128.92(10)°, 6 128.45(8)°).

Selected bond lengths [Å] and bond angles [°] of compounds 2–8

**Table:** 

Bond lengths [Å]	2	3	4	5	6	7	8
Si–O_Δ_^a^	1.7092(11)	—	—	—	—	1.698(4)	—
Si–O	—	1.669(3)	1.677(2)	1.6945(12)	1.6830(10)	1.657(3)	1.646(2)
C–O_Δ/2_^a^^b^	1.4891(13)	—	—	—	—	1.520(8)	1.440(4)
C–O	—	1435(5)	1.437(4)	1.4325(19)	1.4401(16)	1.438(5)	1.448(4)
Ga–C	—	1.995(4)	1.997(3)	1.9980(16)	1.9974(13)	2.029(4)	2.027(3)

a Δ = Part of oxasilirane-ring.

b Part of activated aldehyde (8).

**Table:** 

Bond angles [°]	2	3	4	5	6	7	8
Si–O–C	—	129.5(2)	128.8(2)	128.92(10)	128.45(8)	121.5(2)	125.37(18)
Si–O–C (Δ)^a^	69.83(6)	—	—	—	—	67.9(3)	—
O–C–Ga	—	109.62	109.1(2)	108.49(10)	108.66(8)	107.7(3)	110.57(18)

### Catalytic studies

**Scheme 8:**
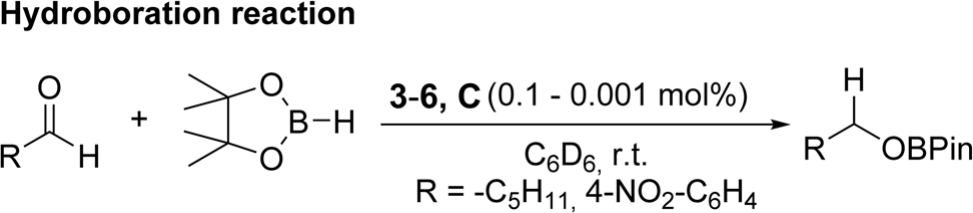
Hydroboration of *n*-hexanal and *para*-nitrobenzaldehyde with 3–6.

The catalytic activity of L^1^(Cl)GaCH(R)OSiL^2^ (R = Et 3, Ph 4, 3-Br-C_6_H_4_5, 4-hexyl-C_6_H_4_6) was compared in reactions of *n*-hexanal and *para*-nitrobenzaldehyde with HBPin at 25 °C and varying catalyst loadings (Scheme 8 and Table 2).

**Table 2:** Catalytic hydroboration of *n*-hexanal and *para*-nitrobenzaldehyde (RCHO) with HBPin with alkoxysilylenes 3–6 and C^a^

Entry	Catalyst	Catalytic load (mol%)	R	*t* (h)	Conversion (%)^c^	*k* (s^−1^ 10^−2^)	TOF (h^−1^)
1^b^	C	0.1	–C_5_H_11_	1	77	1.34	770
2^b^	3	0.1	–C_5_H_11_	1	92	2.14	920
3^b^	4	0.1	–C_5_H_11_	1	67	0.80	670
4^b^	5	0.1	–C_5_H_11_	1	78	0.86	780
5^b^	6	0.1	–C_5_H_11_	1	59	0.44	590
6	C	0.01	–C_6_H_4_-4-NO_2_	0.55	>99	16.94	18 182
7	3	0.01	–C_6_H_4_-4-NO_2_	0.12	>99	79.84	83 333
8^b^	3	0.001	–C_6_H_4_-4-NO_2_	1	98	9.47	98 000
9^b^	4	0.01	–C_6_H_4_-4-NO_2_	1	98	6.97	9800
10	5	0.01	–C_6_H_4_-4-NO_2_	0.66	>99	12.49	15 152
11^b^	6	0.01	–C_6_H_4_-4-NO_2_	1	>99	12.06	10 000
12	—	—	–C_6_H_4_-4-NO_2_	1	>57	—	—

a Reaction conditions: aldehyde/ketone 0.4 mmol, HBPin 0.44 mmol, room temperature in C_6_D_6_. Conversions are calculated based on the relative integration area of the product (OCH_2_) and the starting material signals (OCH) in the ^1^H NMR spectrum.

b Reaction time was limited to 1 h for better comparability.

c Reaction times for full conversion (>99%) are given in the SI (Table S1).

Alkoxysilylene C (entry 1) and a reaction without any catalyst (entry 12) were included in this study for comparison.^60^ The reaction time was typically limited to one hour unless >99% conversion had been reached before and the progress of the reaction was monitored by *in situ*^1^H NMR spectroscopy (one spectrum per minute). A comparison of the catalytic performances of alkoxysilylenes 3–6 and complex C (0.1 mol% catalyst loading) for the hydroboration of the aliphatic aldehyde *n*-hexanal (entries 1–5) showed moderate to good activities, with TOF values ranging from 590 to 920 h^−1^. Catalyst 3 containing the sterically less demanding alkoxide group showed the highest activity within this series (TOF = 920 h^−1^), followed by alkoxysilylenes 5 (780 h^−1^) and C (770 h^−1^), while the sterically most hindered alkoxysilylene 6 showed the lowest TOF value (590 h^−1^). These findings indicate that the initial reaction of the alkoxysilylenes 3–6 with one equivalent of the aldehyde, which yields the corresponding catalytically active oxasiliranes (Int-1), is kinetically hampered with increasing size of the alkoxide unit in alkoxysilylenes 3–6.

**Fig. 2:**
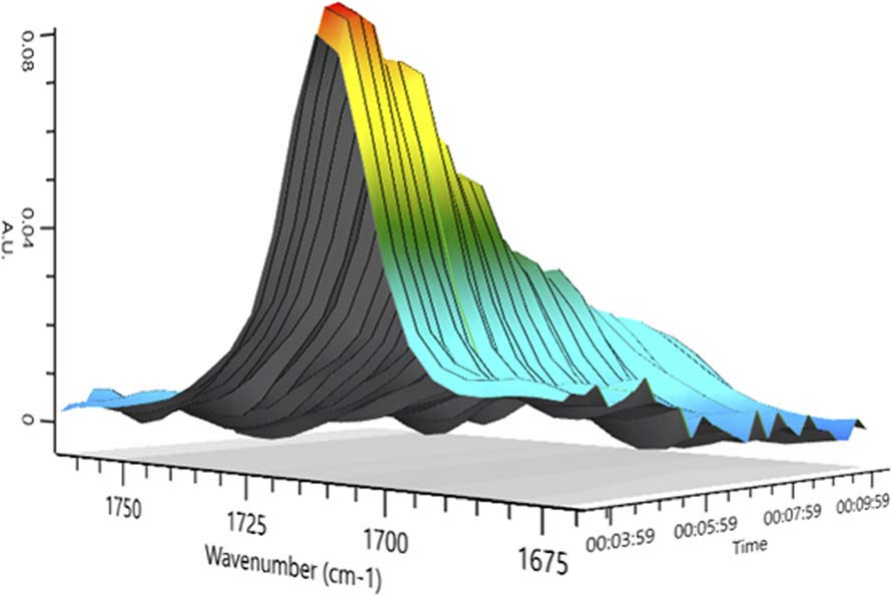
*In situ* IR monitoring of the reaction progress in the catalytic hydroboration of *para*-nitrobenzaldehyde with 3 (0.01 mol%) shown by disappearance of the C=O-band.

Alkoxysilylenes 3–6 showed higher activities in the catalytic hydroboration of the aromatic aldehyde *para*-nitrobenzaldehyde (entries 6–11) even with reduced catalyst loading (0.01 mol%). Alkoxysilylenes 4–6 showed almost quantitative conversions of the aldehyde within one hour or less, with TOF values ranging from 9800 to 18 182 h^−1^. Notably, the sterically less hindered alkoxysilylene 3 showed excellent TOF values of 83 333 h^−1^ (0.01 mol%, 7 min) and 98 000 h^−1^ (0.001 mol%, 60 min). These results were supported by *in situ* IR experiments, in which the reaction was scaled up four times. The aldehyde carbonyl band (C=O) completely disappeared after roughly six minutes (Fig. 2). The reaction without any catalyst, which was performed for comparison, showed 57% conversion after 1 h and 99% after 7 h, respectively.

To further validate compound 3 as a broadly applicable hydroboration catalyst, an expanded substrate scope study was conducted (Table 3) under the same reaction conditions as mentioned before. In all cases quantitative conversions (>99%) were achieved. The reactions proceeded smoothly for a range of aliphatic aldehydes, including *n*-butanal, *iso*-valeraldehyde, and *n*-hexanal, with TOF values of 129, 118, and 752 h^−1^, respectively, demonstrating that both linear and branched aliphatic substrates are well tolerated. Notably, the activities observed for *iso*-valeraldehyde and *n*-hexanal represent a significant improvement over those previously reported with compound C (TOF = 11 and 150 h^−1^),^60^ which is primarily a consequence of optimised reaction conditions. The total concentrations of the aldehyde and borane were doubled compared to the initial study with compound C, while the catalyst loading was reduced to one-tenth. These changes collectively account for the large differences in TOF values observed across these substrates.

**Table 3:** Aliphatic and aromatic aldehydes applied in catalytic hydroboration reactions with 3 and HBPin^a^

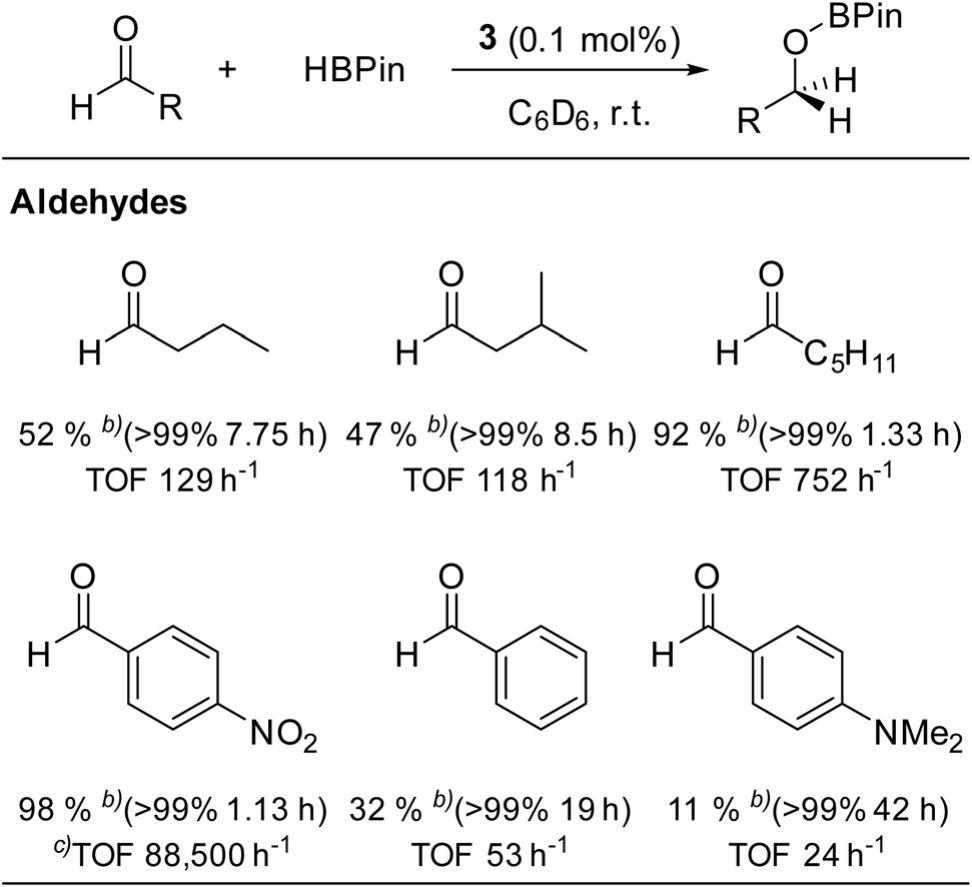

a Reaction conditions: aldehyde 0.4 mmol, HBPin 0.44 mmol, room temperature in C_6_D_6_. Conversions are given after 1 h reaction time for better comparability and are calculated based on the relative integration area of the product (OCH_2_) and the substrate signals (OCH) in the ^1^H NMR spectrum.

b Time for full conversion (>99%). TOF values were calculated for the time of full conversion.

c Catalyst load of 0.001 mol%.

We furthermore tested the catalytic hydroboration of different electron-neutral and electron-rich aromatic aldehydes. Benzaldehyde and 4-dimethylaminobenzaldehyde afforded full conversion with TOF values of 53 and 24 h^−1^, respectively, representing a substantial improvement over the activities previously observed with compound C under the earlier reaction conditions (TOF = 4 h^−1^ and <1 h^−1^, respectively).^60^ The markedly lower activities of these substrates relative to *para*-nitrobenzaldehyde (TOF = 88 500 h^−1^, 0.001 mol%) can be attributed to the reduced electrophilicity of their carbonyl groups. The exceptionally high TOF value for compound 3 with *para*-nitrobenzaldehyde was only achieved with purified aldehyde. We observed markedly varying activities during our investigations while using different commercially available batches of this aldehyde, which most likely result from batch-to-batch varying amounts of impurities. To address this issue, we recrystallised *para*-nitrobenzaldehyde and confirmed its purity by mass spectrometry to ensure a consistent quality prior to the catalytic testing. After recrystallisation, reproducible activities were obtained.

**Fig. 3:**
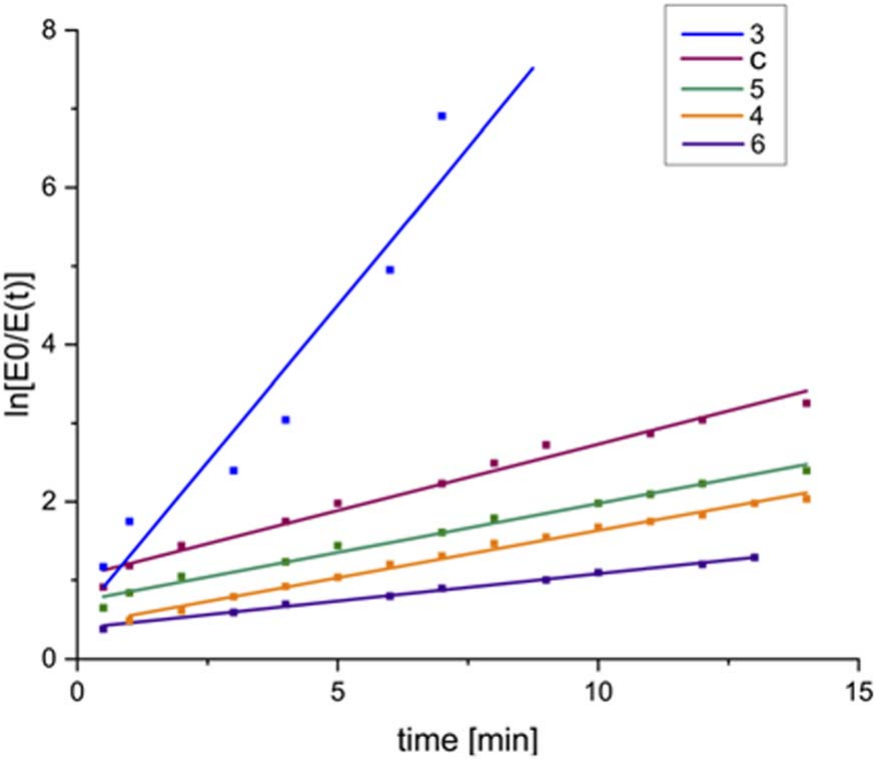
Linearised plot of the aldehyde consumption during catalytic hydroboration of *para*-nitrobenzaldehyde with 3–6 and C (0.01 mol%) monitored *via in situ*^1^H NMR.

The reaction rates of the catalytic hydroboration of *para*-nitrobenzaldehyde with HBPin and 0.01 mol% using catalysts 3–6 and C were determined by *in situ*^1^H NMR studies. Kinetic experiments at room temperature showed first order reaction rates according to the linearised plots (Fig. 3). The highest reaction rate was found for catalyst 3, while the sterically more hindered catalysts 4 and 6 exhibited lower reaction rates. Temperature-dependent kinetic investigations (Table S2) using *para*-nitrobenzaldehyde, HBPin, and 0.001 mol% of catalyst 3 revealed an activation energy of *E*_a_ = 14.6 kcal mol^−1^, which agrees very well with the calculated value. Notably, at 65 °C a TOF value of 400 000 h^−1^ was achieved, corresponding to full conversion within only 15 min.

Our comparative catalysis study indicates strong steric effects of the inserted aldehyde moiety in alkoxysilylenes 3–6. Alkoxysilylene 3 containing the sterically less demanding aldehyde unit was not only the most active catalyst within this series but, to the best of our knowledge, also represents the most active s-, p-, and d-block metal-based catalyst reported, to date. This is supported by quantum chemical calculations (PBE0 ^97^ with D4 ^98^), according to which the HOMO/LUMO gaps of alkoxysilylenes 3–6 and C are comparable (Fig. S74). An electronic effect of the bridging alkoxy unit can therefore be neglected, and the different reactivity regarding the catalysts 3–6 most likely relies on different steric demand of the different alkoxysilylenes.

**Fig. 4:**
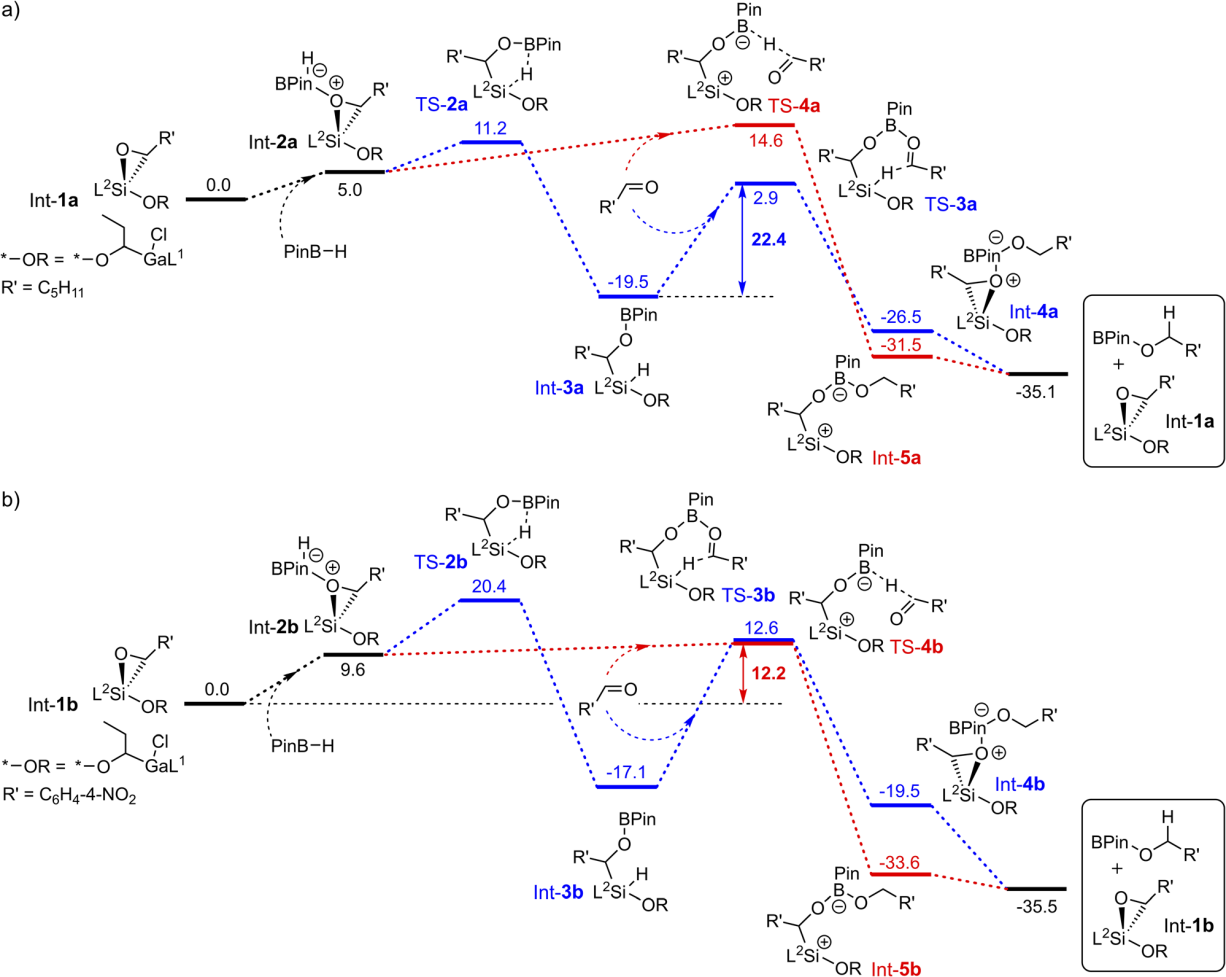
Gibbs energies (G_70%_) for the hydroboration of *n*-hexanal (a) and *para*-nitrobenzaldehyde (b) with Int-1 as catalytically active species calculated by means of PBE0-D3BJ(SMD, benzene as solvent)/def2-TZVP//PBE0-D3BJ/def2-SVP. Herein G_70%_ means that 70% calculated gas-phase entropy contributes to solution-phase free energy. The values are given in kcal mol^−1^. L^2^ = PhC(N^*t*^Bu)_2_, L^1^ = HC[C(Me)NDipp]_2_, Dipp = 2,6-^*i*^Pr_2_C_6_H_3_.

Furthermore, quantum chemical calculations were performed (PBE0-D3J;^99^ see SI) to determine the reason why the reduction of *para*-nitrobenzaldehyde proceeds orders of magnitude faster than that of *n*-hexanal (entries 2 and 8 in Table 2). For this purpose, the reaction of *n*-hexanal and *para*-nitrobenzaldehyde with catalyst 3 was calculated in detail (Fig. 4, S75, and S76). The first step in both cases is the addition of the aldehyde to form the oxasilirane Int-1 (Fig. S75). This is energetically much more favourable than the addition of HBPin followed by the reduction of the aldehydes (Fig. S76).

The addition of HBPin to the oxasilirane Int-1 yields the intermediate Int-2 (Fig. 4). Here, the pathway bifurcates: on the one hand side, the formation of the silanes Int-3 is possible, which transfer the hydride to the aldehyde in the next step *via* TS-3. On the other hand side, a direct transfer of the hydride to the aldehyde *via* TS-4 is also feasible. Which pathway is taken strongly depends on the electronic character of the group attached to the aldehyde. With electron-donating substituents, such as in *n*-hexanal, the formation of the silane (*via* TS-2a) is more favourable than direct hydride transfer (TS-4a), which is why reduction *via* silane (TS-3a) is the rate-limiting step. With electron-withdrawing groups, such as in *para*-nitrobenzaldehyde, direct hydride transfer (TS-4b) is more favourable than silane formation (TS-2b) and is therefore also the rate-determining step. As a result, the reduction of *para*-nitrobenzaldehyde proceeds orders of magnitude faster than that of *n*-hexanal, which is in perfect agreement with the experimental results.

## Experimental section

### Materials and methods

All manipulations were performed in a purified argon atmosphere using standard Schlenk and glovebox techniques. Toluene was dried using an mBraun Solvent Purification System. Deuterated benzene was dried over activated molecular sieves (4 Å) and degassed prior to use. The anhydrous nature of the solvents was verified by Karl Fischer titration. L^2^SiGa(Cl)L^1^A^100^ and L^2^SiOCH[Ga(Cl)L^1^]CH_2_CHMe_2_C^60^ were prepared according to literature methods. Microanalyses were performed at the Microanalysis Laboratory of the University of Duisburg-Essen. Melting points were measured using a Thermo Scientific 9300 apparatus. The synthesis and spectroscopic data of all new compounds are given in the SI.

### Spectroscopic methods

NMR spectra were recorded using a Bruker Ascend™ 400 (^1^H 400 MHz, ^13^C{^1^H} 100 MHz, ^29^Si{^1^H} 79 MHz) and a Bruker AvanceIII HD 600 spectrometer (^1^H 600 MHz, ^13^C{^1^H} 151 MHz, ^29^Si{^1^H} 119 MHz). Catalytic reactions were monitored by ^1^H (400 MHz) and ^11^B (96 MHz) NMR spectroscopy using a Jeol JNM-ECZL spectrometer. The spectra were referenced to internal C_6_D_5_H (^1^H: *δ* = 7.16; ^13^C: *δ* = 128.06). ^11^B{^1^H} and ^29^Si{^1^H} NMR spectra were referenced using IUPAC recommendation of NMR nomenclature. IR spectra were recorded with an ALPHA-T FT-IR spectrometer equipped with a single reflection ATR sampling module. The IR spectrometer was placed inside a glovebox to guarantee measurements under O_2_- and H_2_O-free conditions.

### Single crystal X-ray diffraction

The crystals were mounted on nylon loops in inert oil. Data of 2, 3, and 8 were collected on a Bruker AXS D8 Kappa diffractometer with APEX2 detector (monochromated Mo_Ka_ radiation, *λ* = 0.71073 Å) while those of 4, 5, 6, and 7 were collected on a Bruker AXS D8 Venture diffractometer with Photon II detector (monochromated Cu_Ka_ radiation, *λ* = 1.54178 Å, microfocus source) at 100(2) K. The structures were solved by Direct Methods (SHELXS-2013)^101^ and refined anisotropically by full-matrix least-squares on F^2^ (SHELXL-2017).^102,103^ Absorption corrections were performed semi-empirically from equivalent reflections on the basis of multi-scans and numerical from indexed faces (4) (Bruker AXS APEX3). Hydrogen atoms were refined using a riding model or rigid methyl groups. Further details are given in the SI.

## Conclusions

Alkoxysilylenes 3–6 are efficient pre-catalysts in the catalytic hydroboration of aldehydes with HBPin. Under catalytic conditions, they initially react to the corresponding oxasiliranes as was confirmed by the isolation of oxasilirane 7, which according to detailed mechanistic studies reacts with HBPin with hydride transfer from the borane to the silicon centre and formation of silane 8a, which was spectroscopically characterised. 8a then reacts with another aldehyde with hydride transfer to the electrophilic carbonyl C atom, followed by elimination of the borane ester and formation of oxasilirane 7, which closes the catalytic cycle. The isolation of oxasilirane 7 is furthermore interesting since the introduced stereo centre may allow asymmetric syntheses in the future.

The catalytic activity of the alkoxysilylenes strongly depends on the steric demand of the bridging alkoxy group. Alkoxysilylene 3 containing the sterically less demanding group was found to be the most active complex for the hydroboration of *n*-hexanal and *para*-nitrobenzaldehyde. TOF values as high as 98 000 h^−1^ under very mild reaction conditions (25 °C) and very low catalyst loading (0.001 mol%) were observed with alkoxysilylene 3 and *para*-nitrobenzaldehyde. This excellent activity outperforms that of any other s-, p-, and d-block metal complex reported, to date. A broader substrate study including different aliphatic and aromatic aldehyde furthermore demonstrates that catalyst 3 is widely applicable for the general hydroboration of aldehydes, tolerating a wide range of electronic and steric substrate variations.

The electronic nature of the aldehyde was found to strongly control the rate-limiting step: according to detailed quantum chemical calculations, electron-donating substituents favour the formation of the silane (*via* TS-2a) rather than the direct hydride transfer (TS-4a), whereas electron-withdrawing groups rather favour the direct hydride transfer (TS-4b) than the silane formation (TS-2b).

## Author contributions

L. Kapp – synthesis, characterisation, quantum chemical calculations (HOMO/LUMO gaps), writing manuscript draft. B. Schmetten – synthesis, characterisation. C. Wölper – sc-XRD measurements, writing manuscript draft. G. Haberhauer – quantum chemical calculations (reaction mechanism), writing manuscript draft. S. Schulz – supervision, revision of manuscript, resources, project administration. The manuscript was written through contributions of all authors. All authors have given approval to the final version of the manuscript.

## Conflicts of interest

The authors declare no competing financial interest.

## Supplementary Material

SC-017-D6SC02376B-s001

SC-017-D6SC02376B-s002

## Data Availability

CCDC 2481719 (2), 2481720 (3), 2481721 (4), 2481722 (5), 2481728 (6), 2481729 (7), and 2481730 (8) contain the supplementary crystallographic data for this paper.^104*a*–*g*^ Crystallographic data in cif format and other experimental data, *e.g.*, experimental details, NMR and IR spectra, as well as details from quantum chemical calculations supporting this article have been included as part of the supplementary information (SI). Supplementary information is available. See DOI: https://doi.org/10.1039/d6sc02376b.
