# Supplementary material for: Catalytic hydroboration of aldehydes with electron-rich alkoxysilylenes: from mechanistic insights to highly active catalysts
Source: Chem Sci. 2026 Jul 15;17(32):15263–74. doi: 10.1039/d6sc02376b (PMC13398413; doi:10.1039/d6sc02376b)
Supplement: Supplementary file 1 [file SC-017-D6SC02376B-s001.pdf]

Supplementary Information (SI) for Chemical Science.
This journal is © The Royal Society of Chemistry 2026

Supporting Information

Catalytic Hydroboration of Aldehydes with Electron-Rich Alkoxysilylenes: From Mechanistic Insights to Highly Efficient Catalysts

*Leon Kapp,[a] Brian Schmetten,[a] Christoph Wölper,[a] Gebhard Haberhauer,[b] Stephan Schulz[a,c]**

[a] L. Kapp, B. Schmetten, Dr. C. Wölper, Prof. Dr. S. Schulz, Institute of Inorganic Chemistry, University of Duisburg-Essen, Universitätsstraße 5-7, 45141 Essen (Germany).

[b] Prof. Dr. G. Haberhauer, Institute of Organic Chemistry, University of Duisburg-Essen, Universitätsstraße 5-7, 45141 Essen (Germany).

[c] Center for Nanointegration Duisburg-Essen (CENIDE), University of Duisburg-Essen, Carl-Benz-Straße 199, 47057 Duisburg (Germany).

Corresponding author: stephan.schulz@uni-due.de

Content

I. Experimental section 3-6

II. Spectroscopic Characterization

Fig. S1 – S4. ^{1}H, ^{13}C, ^{29}Si NMR and IR spectra of $L^2Si[OCHC_2H_5]Ga(Cl)L^1$ (**1**). 7, 8

Fig. S5 – S8. ^{1}H, ^{13}C, ^{29}Si NMR and IR spectra of $L^2Si[OCHC_{10}H_{15}]Ga(Cl)L^1$ (**2**). 9, 10

Fig. S9 – S12. ^{1}H, ^{13}C, ^{29}Si NMR and IR spectra of $L^2SiOCH[Ga(Cl)L^1]CH_2CH_3$ (**3**). 11, 12

Fig. S13 – S16. ^{1}H, ^{13}C, ^{29}Si NMR and IR spectra of $L^2SiOCH[Ga(Cl)L^1]C_6H_5$ (**4**). 13, 14

Fig. S17 – S20. ^{1}H, ^{13}C, ^{29}Si NMR and IR spectra of $L^2SiOCH[Ga(Cl)L^1]C_6H_4Br$ (**5**). 15, 16

Fig. S21 – S24. ^{1}H, ^{13}C, ^{29}Si NMR and IR spectra of $L^2SiOCH[Ga(Cl)L^1]C_6H_4C_6H_{13}$ (**6**). 17, 18

Fig. S25 – S30. ^{1}H, ^{13}C, ^{29}Si NMR and IR spectra of $L^2Si[OCHC_{10}H_{15}]OCH[Ga(Cl)L^1]C_6H_5$ (**7**). 19-21

Fig. S31 – S34. ^{1}H, ^{13}C, ^{29}Si and IR spectra of $L^2[H]Si[(C_6H_{12}O_2B)OCHC_{10}H_{15}]OCH[Ga(Cl)L^1]C_6H_5$ (**8**). 22, 23

Fig. S35, 36. *In situ* ^{1}H NMR spectra of the equimolar reaction of compound (**7**) and HBPin in C_6D_6 at room temperature 24

Fig. S37 – S49. ^{1}H NMR spectra of catalytic reactions and mechanistic studies. 25-32

II. Catalytic Studies

Table S1. Catalytic Table 33

Characterization of catalytic products. 35

Fig. S50 – S66. Investigations of reaction kinetics. 36-43

Table S2. Temperature dependent Catalytic Table 42

III. Single Crystal X-ray Diffraction

Table S3. Crystal data and structure refinement of **2-5**. 44, 45

Table S4. Crystal data and structure refinement of **6 – 8**. 46

Figure S67 – S73. Molecular structure of **2-8**. 47-50

IV. Quantum chemical calculations

Figure S74. HOMO-LUMO gaps of compounds **3-6** and **C**. 51

Figure S75. Calculated Gibbs energies ($G_{70\%}$) for the reaction of **3** with aldehydes 52

Figure S76. Calculated Gibbs energies ($G_{70\%}$) for the hydroboration of *n*-hexanal and *para*-nitrobenzaldehyde with **3** as catalytically active species 52

Table S5. Absolute energies of the calculated compounds by means of different methods 53-139

V. References 140, 141

I. Experimental section

Synthesis of L^{2}Si[OCHC$_2$H$_5$]Ga(Cl)L^1 (1). A solution of *n*-propanal (7.39 mg, 0.127 mmol) in 1 mL of toluene was added dropwise to a cooled (–60 °C) solution of L^1(Cl)GaSiL^2 (99.3 mg, 0.127 mmol) in 2 mL of toluene. The solution was stirred at −60 °C for 5 minutes and concentrated to 0.5 mL. After adding 1 mL of *n*-hexane, the solution was stored at –18 °C for 2 h to give oxasilirane **1** in the form of colorless crystals.

Yield 12 mg (0.015 mmol, 11 %). Mp. 132 °C. Elemental analysis for $C_{47}H_{74}ClGaN_4OSi$: Anal. Calcd. C 66.85, H 8.83, N 6.64 %. Found C 65.97, H 8.47, N 6.61 %.IR: ν [cm^{-1}] 3057, 2962, 2929, 2869, 1615, 1529, 1460, 1440, 1390, 1318, 1262, 1193, 1179, 1144, 1100, 1057, 1024, 941, 871, 796, 759, 728, 712, 641, 616, 596, 558, 532, 501, 449, 410. ^{1}H-NMR (600 MHz, C_6D_6): δ [ppm] = 7.37 – 6.82 (m, 11H, $C_6H_3(^i\mathrm{Pr})_2$, C_6H_5), 5.17 (s, 1H, γ-C*H*), 4.22 (sept, $^3J_{HH}$ = 6.8 Hz, 1H, C*H*(CH$_3$)$_2$), 3.95 (sept, $^3J_{HH}$ = 6.7 Hz, 1H, C*H*(CH$_3$)$_2$), 3.53 (sept, $^3J_{HH}$ = 6.8 Hz, 1H, C*H*(CH$_3$)$_2$), 3.39 (sept, $^3J_{HH}$ = 6.7 Hz, 1H, C*H*(CH$_3$)$_2$), 1.91 (dd, $^3J_{HH}$ = 6.2 Hz, 1H, OC*H*), 1.73 (dd, $^3J_{HH}$ = 13.9, 7.2 Hz, 1H, OCHC*H*$_2$CH$_3$), 1.70 (d, $^3J_{HH}$ = 6.7 Hz, 3H, CH(C*H*$_3$)$_2$), 1.67 (s, 3H, ArNCC*H*$_3$), 1.65 (s, 3H, ArNCC*H*$_3$), 1.63 (d, $^3J_{HH}$ = 6.7 Hz, 3H, CH(C*H*$_3$)$_2$), 1.56 (d, $^3J_{HH}$ = 6.6 Hz, 3H, CH(C*H*$_3$)$_2$), 1.54 (d, $^3J_{HH}$ = 4.8 Hz, 1H, OCHC*H*$_2$CH$_3$), 1.51 (d, $^3J_{HH}$ = 6.8 Hz, 3H, CH(C*H*$_3$)$_2$), 1.32 (d, $^3J_{HH}$ = 6.8 Hz, 3H, CH(C*H*$_3$)$_2$), 1.28 (t, $^3J_{HH}$ = 7.7 Hz, 3H, OCHCH$_2$C*H*$_3$), 1.25 (d, $^3J_{HH}$ = 6.7 Hz, 3H, CH(C*H*$_3$)$_2$), 1.16 (d, $^3J_{HH}$ = 6.8 Hz, 3H, CH(C*H*$_3$)$_2$), 1.14 (s, 9H, C(C*H*$_3$)$_3$), 1.12 (d, $^3J_{HH}$ = 6.7 Hz, 3H, CH(C*H*$_3$)$_2$), 0.72 (s, 9H, C(C*H*$_3$)$_3$). ^{13}C-NMR (151 MHz, C_6D_6): δ [ppm] = 174.16 (N*C*N), 169.15, 168.47 (ArN*C*CH$_3$), 146.06, 145.41, 144.75, 144.10 (NC*C*(CH(CH$_3$)$_2$)), 144.02, 143.71 (N*C*C(CH(CH$_3$)$_2$)), 134.04, 129.34, 128.75, 128.57, 127.33, 127.21 (*C*$_6$H$_5$), 129.53, 129.48, 124.89, 124.77, 124.59, 123.98 (*C*$_6$H$_3$), 99.67 (γ-*C*H), 63.51 (O*C*H), 53.59, 52.69 (*C*(CH$_3$)$_3$), 31.49, 31.13 (C(*C*H$_3$)$_3$), 30.34 (OCH*C*H$_2$CH$_3$), 29.18 , 28.22, 28.18, 28.06 (CH(CH$_3$)$_2$), 26.55 (OCHCH$_2$*C*H$_3$), 24.89, 24.30 (ArNC*C*H$_3$), 28.37, 26.55, 24.96, 24.24, 23.45, 21.43 (CH(*C*H$_3$)$_2$). ^{29}Si-NMR (119 MHz, C_6D_6): δ [ppm] = -112.16.

Synthesis of L^{2}Si[OCHC$_{10}$H$_{15}$]Ga(Cl)L^1 (2). 1-Adamantanecarboxaldehyde (41.8 mg, 0.26 mmol) and **L^1**(Cl)GaSi**L**^{2} (200 mg, 0.26 mmol) were dissolved in 3 mL of toluene. The solution was stirred at room temperature for 12 h, concentrated to 0.5 mL and stored at **-**18 °C for 12 h to give oxasilirane **2** in the form of colorless crystals.

Yield 217 mg (0.23 mmol, 88 %). Mp. 193 °C. Elemental analysis for $C_{55}H_{80}ClGaN_4OSi$: Anal. Calcd. C 69.79, H 8.52, N 5.92%. Found C 69.10, H 8.48, N 5.83 %. IR: ν 3064, 3027, 2962, 2902, 2883, 2846, 1617, 1541, 1520, 1462, 1435, 1386, 1361, 1320, 1284, 1260, 1242, 1227, 1200, 1179, 1105, 1057, 1040, 1020, 986, 937, 927, 911, 882, 870, 862, 824, 794, 759, 750, 705, 699, 686, 682, 649, 635, 599, 586, 569, 534, 508, 501, 457, 443, 417 . ^{1}H-NMR (400 MHz, C_6D_6): δ [ppm] = 7.38 - 6.89 (m, 11H, $C_6H_3(^i\mathrm{Pr})_2$ & C_6H_5), 5.21 (s, 1H, γ-C*H*), 4.31 (sept, $^3J_{HH}$ = 6.6 Hz, 1H, C*H*(CH$_3$)$_2$), 4.12 (sept, $^3J_{HH}$ = 6.6 Hz, 1H, C*H*(CH$_3$)$_2$), 3.44 (sept, $^3J_{HH}$ = 6.7 Hz, 2H, C*H*(CH$_3$)$_2$), 2.11 (s, 3H, C$_{10}$H$_{15}$ (C*H*)), 1.81 (s, 6H, C$_{10}$H$_{15}$ (C*H*$_2$)), 1.76 (d, $^3J_{HH}$ = 1.7 Hz, 3H, CH(C*H*$_3$)$_2$), 1.71 (d, $^3J_{HH}$ = 6.7 Hz, 3H, CH(C*H*$_3$)$_2$), 1.66 (s, 3H), 1.63 (s, 3H), 1.62 (d, $^3J_{HH}$ = 5.7 Hz, 3H, CH(C*H*$_3$)$_2$), 1.49 (d, $^3J_{HH}$ = 6.7 Hz, 3H, CH(C*H*$_3$)$_2$), 1.43 (d, $^3J_{HH}$ = 6.8 Hz, 3H, CH(C*H*$_3$)$_2$), 1.39 (s, 9H, C(C*H*$_3$)$_3$), 1.33 (d, $^3J_{HH}$ = 6.9 Hz, 3H, CH(C*H*$_3$)$_2$), 1.30 (d, $^3J_{HH}$ = 6.7 Hz, 3H, CH(C*H*$_3$)$_2$), 1.20 (s, 1H, OC*H*), 1.15 (dd, $^3J_{HH}$ = 6.7, 4.9 Hz, 3H, CH(C*H*$_3$)$_2$), 0.80 (s, 9H, C(C*H*$_3$)$_3$). ^{13}C-NMR (101 MHz, C_6D_6): δ [ppm] = 172.61 (N*C*N), 169.00, 168.11 (ArN*C*CH$_3$), 145.91, 144.75, 144.20 (NC*C*(CH(CH$_3$)$_2$)), 144.05, 143.80 (N*C*C(CH(CH$_3$)$_2$)), 133.47, 128.23, 127.15, 127.10, 126.92, 126.77 (*C*$_6$H$_5$), 129.39, 129.05, 124.47, 124.13, 124.01 (*C*$_6$H$_3$), 100.25 (γ-*C*H), 70.02 (O*C*H), 53.92, 52.13 (*C*(CH$_3$)$_3$), 40.74 (C$_{10}$H$_{15}$ (*C*H$_2$)), 37.74 (C$_{10}$H$_{15}$ (*C*H$_2$)), 37.00 ((OCH)*C*), 31.64, 31.47 (C(*C*H$_3$)$_3$), 29.77, 28.76, 27.96, 27.91 (*C*H(CH$_3$)$_2$), 29.12 (C$_{10}$H$_{15}$ (*C*H)), 28.86, 25.77, 25.16 , 24.13, 23.90, 23.56, 23.45, 24.94 (CH(*C*H$_3$)$_2$), 24.69, 24.24 (ArNC*C*H$_3$). ^{29}Si-NMR (79 MHz, C_6D_6): δ [ppm] = -110.03 (s).

Synthesis of L^{2}SiOCH[Ga(Cl)L^1]CH$_2$CH$_3$ (3). A solution of ***n*-**propanal (7.39 mg, 0.127 mmol) in 1 mL of toluene was added dropwise to a solution of L^1(Cl)GaSiL^2 (99.3 mg, 0.127 mmol) in 2 mL of toluene at 0 °C. The resulting solution was stirred for 12 h upon warming to room temperature. The solvent was removed *in vacuo* and the remaining solid was dissolved in 4 mL of *n*-hexane. After concentration to 0.5 mL, colorless crystals of **3** were formed within 12 h upon storage at room temperature.

Yield 60 mg (0.2 mmol, 66 %). Mp. 140 °C. Elemental analysis for $C_{47}H_{74}ClGaN_4OSi$: Anal. Calcd. C 66.85, H 8.83, N 6.64 %. Found C 65.8, H 8.46, N 6.64 %. IR: ν [cm^{-1}] 3059, 2962, 2927, 2867, 1610, 1526, 1462, 1438, 1384, 1316, 1258, 1235, 1204, 1177, 1109, 1074, 1020, 989, 937, 885, 846, 796, 761, 705, 629, 600, 552, 530, 501, 467, 443, 407. ^{1}H-NMR (400 MHz, C_6D_6): δ [ppm] = 7.24 – 6.80 (m, 11H, $C_6H_3(^i\mathrm{Pr})_2$, C_6H_5), 5.04 (s, 1H, γ-C*H*), 4.13 (t, 1H, OC*H*), 4.09 (sept, $^3J_{HH}$ = 6.7 Hz, 1H, C*H*(CH$_3$)$_2$), 4.00 (sept, $^3J_{HH}$ = 6.8 Hz, 1H, C*H*(CH$_3$)$_2$), 3.56 (sept, $^3J_{HH}$ = 6.8 Hz, 1H, C*H*(CH$_3$)$_2$), 3.47 (sept, $^3J_{HH}$ = 6.7 Hz, 1H, C*H*(CH$_3$)$_2$), 1.86 (quin, 2H, OCHC*H*$_2$CH$_3$), 1.66 (s,

3H, ArNCCH_3), 1.66 – 1.63 (m, 8H, CH(CH_3)$_2$), 1.62 (s, 4H, ArNCCH_3 & CH(CH_3)$_2$), 1.60 (d, $^3J_{HH}$ = 6.6 Hz, 3H, CH(CH_3)$_2$), 1.28 (d, $^3J_{HH}$ = 6.9 Hz, 3H, CH(CH_3)$_2$), 1.26 (d, $^3J_{HH}$ = 6.8 Hz, 3H, CH(CH_3)$_2$), 1.14 (d, $^3J_{HH}$ = 6.6 Hz, 3H, CH(CH_3)$_2$), 1.12 (d, 3H, CH(CH_3)$_2$), 1.12 (s, 10H, C(CH$_3$)$_3$ & CH(CH_3)$_2$), 1.03 (t, $^3J_{HH}$ = 7.6 Hz, 3H, OCHCH$_2$CH_3), 0.96 (s, 9H, C(CH$_3$)$_3$). ^{13}C-NMR (101 MHz, C_6D_6): δ [ppm] = 169.72, 169.65 (ArN*C*CH$_3$), 162.03 (N*C*N), 146.00, 145.75, 143.13, 142.92 (NC*C*(CH(CH$_3$)$_2$)), 142.28, 142.17 (N*C*C(CH(CH$_3$)$_2$)), 134.49, 130.13, 129.19, 127.76, 127.42, 127.37 (*C*$_6$H$_5$), 125.31, 125.15, 124.09, 123.84 (*C*$_6$H$_3$), 98.52 (γ-*C*H), 73.90 (O*C*H), 52.86, 52.43 (*C*(CH$_3$)$_3$), 31.94, 31.73 (C(*C*H$_3$)$_3$), 29.87 (OCH*C*H$_2$CH$_3$), 29.49, 29.41, 28.13, 28.09 (*C*H(CH$_3$)$_2$), 27.02, 26.55 (ArNC*C*H$_3$), 25.16, 25.08, 24.64, 24.54, 23.97, 23.74 (CH(*C*H$_3$)$_2$), 13.25 (OCHCH$_2$*C*H$_3$). ^{29}Si-NMR (79 MHz, C_6D_6): δ [ppm] = -16.98 (s).

Synthesis of L^{2}SiOCH[Ga(Cl)L^1]C$_6$H$_5$ (4). A solution of benzaldehyde (21.96 mg, 0.2 mmol) in 1.5 mL of toluene was added dropwise to a solution of L^1(Cl)GaSiL^2 (150 mg, 0.2 mmol) in 1.5 mL of toluene. The resulting solution was stirred for 12 h, after which the solvent was removed under vacuum and the remaining solid was washed with *n*-hexane and then dissolved in 1 mL of toluene. Colorless crystals of **4** were formed within 12 h upon storage at room temperature.

Yield 112 mg (0.13 mmol, 63 %). Mp. 224 °C. Elemental analysis for $C_{51}H_{70}ClGaN_4OSi$: Anal. Calcd. C 68.95, H 7.94, N 6.31 %. Found C 69.7, H 8.50, N 5.79 %. IR: ν [cm^{-1}] 3060, 3022, 2962, 2928, 2870, 1524, 1495, 1458, 1435, 1384, 1361, 1320, 1262, 1206, 1179, 1100, 1071, 1059, 1022, 999, 943, 933, 908, 869, 798, 763, 730, 697, 641, 616, 590, 532, 494, 465, 436. ^{1}H-NMR (600 MHz, C_6D_6): δ [ppm] 7.52 – 6.57 (m, 16H, C$_6$$H_3(^{i}Pr)_2$, C$_6$$H_5$, OCC$_6$$H_5$), 5.14 (s, 1H, OC*H*), 4.99 (s, 1H, γ-C*H*), 4.26 – 4.03 (sept, $^3J_{HH}$ = 6.8 Hz, 1H, C*H*(CH$_3$)$_2$), 3.82 (sept, $^3J_{HH}$ = 6.8 Hz, 1H, C*H*(CH$_3$)$_2$), 3.64 (sept, $^3J_{HH}$ = 6.8 Hz, 1H, C*H*(CH$_3$)$_2$), 3.47 (sept, $^3J_{HH}$ = 6.7 Hz, 1H, C*H*(CH$_3$)$_2$), 1.88 (d, $^3J_{HH}$ = 6.8 Hz, 6H, CH(CH_3)$_2$), 1.60 (s, 3H, ArNCCH_3), 1.58 (s, 3H, ArNCCH_3), 1.54 (d, $^3J_{HH}$ = 6.6 Hz, 3H, CH(CH_3)$_2$), 1.28 (d, $^3J_{HH}$ = 6.9 Hz, 3H, CH(CH_3)$_2$), 1.16 (d, $^3J_{HH}$ = 6.2 Hz, 6H, CH(CH_3)$_2$), 1.11 (d, $^3J_{HH}$ = 6.8 Hz, 3H, CH(CH_3)$_2$), 0.90 (s, 9H, C(CH_3)$_3$), 0.87 (s, 9H, C(CH_3)$_3$), 0.75 (d, $^3J_{HH}$ = 6.5 Hz, 3H, CH(CH_3)$_2$). ^{13}C-NMR (101 MHz, C_6D_6): δ [ppm] 170.39, 170.17 (ArN*C*CH$_3$), 164.63, 146.59, (*C*$_6$H$_5$), 144.51, 143.84, 142.70, 142.45 (N*C*C(CH(CH$_3$)$_2$)), 134.26, 131.08, 129.57 (*C*$_6$H$_3$), 129.34, 128.81, 127.73, 127.16 (*C*$_6$H$_5$), 125.96, 124.78, 124.69, 124.02 (*C*$_6$H$_5$), 98.48 (γ-*C*H), 76.10 (O*C*H), 53.12, 52.50 (*C*(CH$_3$)$_3$), 31.63, 31.36 (C(*C*H$_3$)$_3$), 29.96, 29.81, 28.22, 28.13 (*C*H(CH$_3$)$_2$), 27.60, 26.04, 25.56, 25.41 (CH(*C*H$_3$)$_2$), 24.91, 24.50 (ArN*C*CH$_3$), 24.28, 23.60 (CH(*C*H$_3$)$_2$). ^{29}Si-NMR (119 MHz, C_6D_6): δ [ppm] -14.09.

Synthesis of L^{2}SiOCH[Ga(Cl)L^1]C$_6$H$_4$Br (5). A solution of 3-bromobenzaldehyde (37 mg, 0.2 mmol) in 1.5 mL of toluene was added dropwise to a solution of **L^1**(Cl)GaSi**L^2** (150 mg, 0.2 mmol) in 1.5 mL of toluene at room temperature. The resulting solution was stirred for 12 h, after which the solvent was removed *in vacuo*. The resulting solid was washed with 5 mL of *n*-hexane, dissolved in 2 mL of benzene and concentrated to 0.5 mL. Pale-yellow crystals of **5** were formed within 12 h upon storage at room temperature.

Yield 137 mg (0.14 mmol, 70 %). Mp. 187 °C. Elemental analysis for $C_{51}H_{69}ClBrGaN_4OSi$: Anal. Calcd. C 63.33, H 7.19, N 5.79 %. Found C 64.2, H 7.35, N 5.22 %. IR: ν [cm^{-1}] 2962, 2926, 2865, 1589, 1524, 1460, 1435, 1384, 1361, 1318, 1260, 1204, 1179, 1100, 1066, 1005, 935, 894, 869, 798, 782, 761, 751, 730, 709, 693, 674, 639, 608, 531, 497, 479, 470, 441, 407. ^{1}H-NMR (400 MHz, C_6D_6): δ [ppm] 7.44 – 6.49 (m, 15H, C$_6$$H_3(^{i}Pr)_2$, C$_6H_5$, OCC$_6$$H_5$), 5.10 (s, 1H, OC*H*), 4.97 (s, 1H, γ-C*H*), 4.02 (sept, $^3J_{HH}$ = 6.6 Hz, 1H, C*H*(CH$_3$)$_2$), 3.78 (sept, $^3J_{HH}$ = 6.7 Hz, 1H, C*H*(CH$_3$)$_2$), 3.59 (sept, $^3J_{HH}$ = 6.8 Hz, 1H, C*H*(CH$_3$)$_2$), 3.48 (sept, $^3J_{HH}$ = 6.7 Hz, 1H, C*H*(CH$_3$)$_2$), 1.86 (d, $^3J_{HH}$ = 6.1 Hz, 3H, CH(CH_3)$_2$), 1.85 (d, $^3J_{HH}$ = 6.2 Hz, 3H, CH(CH_3)$_2$), 1.58 (s, 3H, ArNCCH_3), 1.55 (s, 3H, ArNCCH_3), 1.49 (d, $^3J_{HH}$ = 6.6 Hz, 3H, CH(CH_3)$_2$), 1.25 (d, $^3J_{HH}$ = 6.9 Hz, 3H, CH(CH_3)$_2$), 1.13 (dd, $^3J_{HH}$ = 10.0, 4.9 Hz, 9H, CH(CH_3)$_2$), 0.95 (s, 9H, C(CH_3)$_3$), 0.88 (d, $^3J_{HH}$ = 6.9 Hz, 3H, CH(CH_3)$_2$), 0.85 (s, 9H, C(CH_3)$_3$). ^{13}C-NMR (101 MHz, C_6D_6): δ [ppm] 170.47, 170.41 (ArN*C*CH$_3$), 165.25 (N*C*N), 149.78, 146.05, 145.78, 144.42, 143.51 (NC*C*(CH(CH$_3$)$_2$)), 142.16, 142.04 (N*C*C(CH(CH$_3$)$_2$)), 134.09, 130.64, 127.89, 124.79 (Br*C*$_6$H$_4$), 131.16, 127.27, 126.22 (*C*$_6$H$_5$), 129.48, 125.44, 123.96, 122.69 (*C*$_6$H$_3$), 98.53 (γ-*C*H), 75.38 (O*C*H), 53.31, 52.58 (*C*(CH$_3$)$_3$), 31.63, 31.41 (C(*C*H$_3$)$_3$), 29.98, 29.76, 28.21, 28.13 (*C*H(CH$_3$)$_2$), 27.47, 25.79, 25.55, 25.40, 25.33, 24.22, 23.53 (CH(*C*H$_3$)$_2$), 24.86, 24.51 (ArNC*C*H$_3$). ^{29}Si-NMR (79 MHz, C_6D_6): δ [ppm] -13.35.

Synthesis of L^{2}SiOCH[Ga(Cl)L^1]C$_6$H$_5$C$_6$H$_{13}$ (6). A solution of 4-*n*-hexyl-benzaldehyde (38 mg, 0.2 mmol) in 2 mL of toluene was added dropwise to a solution of L^1(Cl)GaSiL^2 (150 mg, 0.2 mmol) in 2 mL of toluene and the resulting solution was stirred for 12 h. The solvent was then removed *in vacuo,* and the resulting solid was dissolved in 4 mL of *n*-hexane and concentrated to 0.5 mL. Colorless crystals of **6** were formed within 12 h upon storage at -80 °C.

Yield 98 mg (0.1 mmol, 50 %). Mp. 229 °C. Elemental analysis for $C_{57}H_{82}ClGaN_4OSi$: Anal. Calcd. C 70.39, H 8.50, N 5.76 %. Found C 69.6, H 8.43, N 5.37 %. IR: ν [cm^{-1}] 2962, 2927, 2862, 1526, 1460, 1435, 1402, 1386, 1361, 1320, 1262, 1208, 1179, 1098, 1071, 1061, 1020, 999, 944, 928, 867, 849, 798, 759, 751, 709, 646, 626, 605, 569, 544, 496, 475, 439. ^1H-NMR (400 MHz, C_6D_6): δ [ppm] 7.40 – 6.63 (m, 15H, $C_6H_3(^iPr)_2$, C_6H_5, OCC_6H_4), 5.14 (s, 1H, OC*H*), 5.00 (s, 1H, γ-C*H*), 4.09 (sept, $^3J_{HH}$ = 6.7 Hz, 1H, C*H*$(CH_3)_2$), 3.83 (sept, $^3J_{HH}$ = 6.7 Hz, 1H, C*H*$(CH_3)_2$), 3.65 (sept, $^3J_{HH}$ = 6.8 Hz, 1H, C*H*$(CH_3)_2$), 3.51 (sept, $^3J_{HH}$ = 6.6 Hz, 1H, C*H*$(CH_3)_2$), 2.50 (t, $^3J_{HH}$ = 7.7 Hz, 2H, $ArCH_2C_5H_{11}$), 1.89 (d, $^3J_{HH}$ = 6.7 Hz, 6H, $CH(CH_3)_2$), 1.74 – 1.64 (m, 2H, $ArCH_2CH_2C_4H_9$), 1.61 (s, 3H, $ArNCCH_3$), 1.58 (s, 3H, $ArNCCH_3$), 1.56 (d, $^3J_{HH}$ = 6.5 Hz, 3H, $CH(CH_3)_2$), 1.28 (d, $^3J_{HH}$ = 6.8 Hz, 9H, $CH(CH_3)_2$ & $ArC_2H_4C_3H_6CH_3$), 1.17 (d, $^3J_{HH}$ = 6.8 Hz, 6H, $CH(CH_3)_2$), 1.13 (d, $^3J_{HH}$ = 6.8 Hz, 3H, $CH(CH_3)_2$), 0.92 (s, 9H, $C(CH_3)_3$), 0.89 (s, 3H, $ArC_5H_{10}CH_3$), 0.88 (s, 9H, $C(CH_3)_3$), 0.80 (d, $^3J_{HH}$ = 6.5 Hz, 3H, $CH(CH_3)_2$). ^{13}C-NMR (101 MHz, C_6D_6): δ [ppm] 170.59, 170.33 ($ArNCCH_3$), 164.52 (NCN),146.34, 146.27, 144.74, 144.06 ($NCC(CH(CH_3)_2)$), 142.99, 142.70 ($NCC(CH(CH_3)_2)$), 144.15, 134.57, 129.04, 127.89 (OCC_6H_4), 139.09 (CC_6H_5), 131.38, 127.43, 126.08 (C_6H_5), 129.80, 129.54, 128.13, 126.17, 125.58, 124.99 (C_6H_3), 98.70 (γ-CH), 76.13 (OCH), 53.34, 52.74 ($C(CH_3)_3$), 31.88, 31.61 ($C(CH_3)_3$), 36.62 ($ArCH_2C_5H_{11}$), 32.74 ($ArCH_2CH_2C_4H_9$), 30.19, 30.06, 28.45, 28.37 ($CH(CH_3)_2$), 27.83, 26.39 ($ArC_2H_4C_2H_4C_2H_5$), 25.79, 25.69, 25.64, 25.16, 24.75, 23.85, 23.58 ($CH(CH_3)_2$), 24.72, 24.50 ($ArNCCH_3$), 21.90 ($ArC_4H_8CH_2CH_3$), 14.89 ($ArC_5H_{10}CH_3$). ^{29}Si-NMR (119 MHz, C_6D_6): δ [ppm] -13.70.

Synthesis of $L^2Si[OCHC_{10}H_{15}]OCH[Ga(Cl)L^1]C_6H_5$ (7). A solution of 1-adamantanecarboxaldehyde (92.4 mg, 0.56 mmol) in 5 mL of 1,2-difluorobenzene was slowly added dropwise to a solution of $L^1(Cl)GaSiL^2$ (500 mg, 0.56 mmol) in 20 mL of 1,2-difluorobenzene at -30 °C and stirred for 30 min. Under continuous cooling, the solution was then concentrated to 1 mL. Colorless crystals of **7** were formed within 12 h upon storage at room temperature.

Yield 417 mg (0.39 mmol, 70 %). Mp. 242 °C. Elemental analysis for $C_{62}H_{86}ClGaN_4O_2Si$: Anal. Calcd. C 70.74, H 8.24, N 5.32 %. Found C 70.9, H 8.45, N 5.14 %. IR: ν 2966, 2923, 2900, 2846, 1618, 1526, 1506, 1483, 1460, 1438, 1402, 1390, 1363, 1347, 1320, 1268, 1204, 1179, 1100, 1086, 1074, 1057, 1032, 1022, 929, 887, 867, 840, 796, 788, 751, 712, 699, 654, 641, 613, 599, 565, 547, 528, 503, 451, 443, 419. **Diastereomer 7a**: ^1H-NMR (600 MHz, C_6D_6): δ [ppm] = 7.55 – 6.32 (m, 15H, 2 x $C_6H_3(^iPr)_2$ & 2 x C_6H_5), 5.69 (s, 1H, OC*H*Ga), 4.96 (s, 1H, γ-C*H*), 3.95 (m, 1H, C*H*$(CH_3)_2$), 3.54 (m, 1H, C*H*$(CH_3)_2$), 3.48 (m, 1H, C*H*$(CH_3)_2$), 3.39 (m, 1H, C*H*$(CH_3)_2$), 2.21 (s, 3H, $C_{10}H_{15}$ (C*H*)), 2.16 (s, 1H, Si(OC*H*)), 1.97 (s, 6H, $C_{10}H_{15}$ (CH_2)), 1.92 (d, $^3J_{HH}$ = 6.8 Hz, 3H, $CH(CH_3)_2$), 1.90 – 1.86 (m, $^3J_{HH}$ = 12.1 Hz, 3H, $C_{10}H_{15}$ (CH_2)), 1.79 – 1.77 (m, 3H, $C_{10}H_{15}$ (CH_2)), 1.74 (d, $^3J_{HH}$ = 6.6 Hz, 3H, $CH(CH_3)_2$), 1.65 (d, $^3J_{HH}$ = 6.9 Hz, 3H, $CH(CH_3)_2$), 1.59 (s, 3H, $ArNCCH_3$), 1.50 (s, 3H, $ArNCCH_3$), 1.30 (d, $^3J_{HH}$ = 6.9 Hz, 3H, $CH(CH_3)_2$), 1.26 (d, *J* = 6.9 Hz, 3H, $CH(CH_3)_2$), 1.19 (d, $^3J_{HH}$ = 6.7 Hz, 3H, $CH(CH_3)_2$), 1.15 (s, 9H, $C(CH_3)_3$), 1.08 (d, $^3J_{HH}$ = 7.0 Hz, 3H, $CH(CH_3)_2$), 0.76 (s, 9H, $C(CH_3)_3$), 0.71 (d, $^3J_{HH}$ = 6.6 Hz, 3H, $CH(CH_3)_2$).^{13}C-NMR (151 MHz, C_6D_6): δ [ppm] = 173.08 (NCN), 169.96, 169.51 ($ArNCCH_3$), 151.79, 151.69, 150.14, 150.04, 147.54, 145.98, 145.33, 143.28, 142.55, 133.63, 133.07, 129.50, 129.27, 128.35, 125.90, 124.64, 117.50, 117.39 ($C_6H_3(^iPr)_2$ & 2 x C_6H_5), 98.16 (γ-CH), 75.62 (Si(OCH)), 72.36 (OCHGa), 53.44, 52.44 ($C(CH_3)_3$), 41.61, 38.26, 36.79 ($C_{10}H_{15}$ (CH_2)), 29.56 ($C_{10}H_{15}$ (CH)), 31.54, 31.14, 28.33, 25.73, 25.25, 24.63, 24.42, 23.76 ($CH(CH_3)_2$), 24.03, 23.46 ($ArNCCH_3$).^{29}Si-NMR (119 MHz, C_6D_6): δ [ppm] = -113.97 (s). **Diastereomer 7b**: ^1H-NMR (600 MHz, C_6D_6): δ [ppm] = 7.41 – 6.70 (m, 16H, $C_6H_3(^iPr)_2$ & 2 x C_6H_5), 5.48 (s, 1H, OC*H*Ga), 5.01 (s, 1H, γ-C*H*), 4.27 – 4.09 (m, 1H, C*H*$(CH_3)_2$), 4.11 – 4.01 (m, 1H, C*H*$(CH_3)_2$), 3.11 – 2.85 (m, 2H, C*H*$(CH_3)_2$), 2.02 (s, 3H $C_{10}H_{15}$ (C*H*)), 1.92 (s, 1H, Si(OC*H*)), 1.81 (d, $^3J_{HH}$ = 11.5 Hz, 6H, $C_{10}H_{15}$ (CH_2)), 1.77 – 1.76 (m, 3H, -$CH(CH_3)_2$), 1.72 (s, 6H, $C_{10}H_{15}$ (CH_2)), 1.68 (d, $^3J_{HH}$ = 6.6 Hz, 3H, $CH(CH_3)_2$), 1.57 (s, 3H, $ArNCCH_3$), 1.57 (s, 3H, $ArNCCH_3$), 1.30 (d, $^3J_{HH}$ = 6.8 Hz, 3H, $CH(CH_3)_2$), 1.28 – 1.27 (m, 3H, $CH(CH_3)_2$), 1.26 (s, 9H, $C(CH_3)_3$), 1.10 (d, $^3J_{HH}$ = 7.8 Hz, 3H, $CH(CH_3)_2$), 1.09 (s, 9H, $C(CH_3)_3$), 1.01 (d, $^3J_{HH}$ = 6.7 Hz, 3H, $CH(CH_3)_2$), 0.92 (d, $^3J_{HH}$ = 6.7 Hz, 3H, $CH(CH_3)_2$), 0.91 – 0.89 (m, 3H, $CH(CH_3)_2$). ^{13}C-NMR (151 MHz, C_6D_6): δ [ppm] = 173.55 (NCN), 170.20, 170.14 ($ArNCCH_3$), 150.71, 146.48, 145.63, 142.68, 142.46, 141.78, 129.67, 129.33, 129.05, 128.57, 127.34, 127.32, 125.44, 125.38, 117.61, 117.47, 117.42, 117.28 ($C_6H_3(^iPr)_2$ & 2 x C_6H_5), 98.86 (γ-CH), 76.63 (Si(OCH)), 73.86 (OCHGa), 53.29, 53.07 ($C(CH_3)_3$), 41.47, 38.05, 36.44 ($C_{10}H_{15}$ (CH_2)), 29.43 ($C_{10}H_{15}$ (CH)), 31.86 , 29.91, 28.82, 28.15, 27.65, 26.65, 25.19, 24.65 , 24.58 ($CH(CH_3)_2$), 24.06, 23.68 ($ArNCCH_3$). ^{29}Si-NMR (119 MHz, C_6D_6): δ [ppm] = -113.97 (s).

Synthesis of $L^2[H]Si[(C_6H_{12}O_2B)OCHC_{10}H_{15}]OCH[Ga(Cl)L^1]C_6H_5$ (8). HBPin (6.08 mg, 0.05 mmol) and $L^2Si[OCHC_{10}H_{15}]OCH[Ga(Cl)L^1]C_6H_6$ (50 mg, 0.05 mmol) were placed in a *J*-Young NMR tube, dissolved in 1 mL of C_6D_6 and heated to 40 °C for 24 h. Afterwards the solvent was removed under vacuum, the remaining solid was dissolved in 3 mL of fluorobenzene and concentrated to 0.5 mL to give colorless crystals of **8** within 12 h upon storage at -18 °C.

Yield 17 mg (0.014 mmol, 30 %). Mp. 207 °C. Elemental analysis for $C_{68}H_{99}ClBGaN_4O_4Si$: Anal. Calcd. C 69.18, H 8.45, N 4.75 %. Found C 69.63, H 8.40, N 4.24 %. IR: ν 3064, 2962, 2902, 2883, 2846, 1541, 1456, 1435, 1400, 1386, 1361, 1320, 1284, 1260, 1227, 1179, 1105, 1089, 1076, 1057, 1040, 1020, 1010, 987, 967, 937, 927, 881, 870, 835, 794, 759, 705, 683, 649, 624, 599, 534, 501, 457, 443, 417. ^{1}H-NMR (600 MHz, C_6D_6): δ [ppm] = 8.02 – 6.41 (m, 15H, 2 x $C_6H_3(^i\text{Pr})_2$ & 2 x C_6H_5), 5.78 (s, 1H, OC*H*), 5.17 (s, 1H, OC*H*), 4.97 (s, 1H, , γ-C*H*), 4.15 (s, 1H, NC*H*N), 3.99 – 3.89 (m, 1H, C*H*$(CH_3)_2$), 3.74 (sept, $^3J_{HH}$ = 6.7 Hz, 1H, C*H*$(CH_3)_2$), 3.25 (sept, $^3J_{HH}$ = 6.8 Hz, 1H, C*H*$(CH_3)_2$), 2.85 (sept, $^3J_{HH}$ = 6.6 Hz, 1H, C*H*$(CH_3)_2$), 2.34 (d, $^3J_{HH}$ = 11.6 Hz, 3H), 2.25 (s, 4H, $C_{10}H_{15}$ (CH_2)), 2.09 (d, $^3J_{HH}$ = 11.5 Hz, 3H), 2.02 (s, 2H), 1.88 (t, $^3J_{HH}$ = 10.2 Hz, 3H), 1.69 (d, $^3J_{HH}$ = 6.6 Hz, 3H, $CH(CH_3)_2$), 1.49 (s, 3H, ArNCCH_3), 1.48 (d, $^3J_{HH}$ = 6.1 Hz, 3H, $CH(CH_3)_2$), 1.42 (s, 3H, ArNCCH_3), 1.36 – 1.30 (m, 6H, $CH(CH_3)_2$), 1.24 (d, $^3J_{HH}$ = 5.4 Hz, 6H), 1.13 (s, 9H, $C(CH_3)_3$), 0.99 (s, 12H, Pin), 0.91 (d, $^3J_{HH}$ = 6.7 Hz, 6H, $CH(CH_3)_2$), 0.72 (s, 9H, $C(CH_3)_3$). ^{13}C NMR (151 MHz, C_6D_6): δ [ppm] = 170.98 (s), 169.71 (s), 148.35 (s), 145.92 (s), 145.33 (s), 145.13 (s), 143.06 (s), 141.78 (s), 130.51 (s), 126.04 (s), 125.90 (s), 125.00 (s), 124.29 (s), 123.95 (s), 99.47 (s), 82.38 (s), 81.91 (s), 79.73 (s), 73.77 (s), 50.22 (s), 50.08 (s), 40.66 (s), 38.49 (s), 37.69 (s), 32.15 (s), 31.96 (s), 30.85 (s), 29.55 (s), 25.36 (s), 25.10 (s), 24.82 (s), 24.32 (s), 24.10 (s), 23.91 (s), 23.53 (s), 23.05 (s), 14.35 (s). ^{29}Si NMR (119 MHz, C_6D_6): δ [ppm] = -34.09 (s). ^{29}Si{H}DEPT90-NMR (79 MHz, C_6D_6): δ [ppm] = -64.61 (s).

Mechanistic Investigations

Determination of silane 8a formation and following hydroboration of 1-adamantanecarboxaldehyde. HBPin (2.43 mg, 0.02 mmol, 1 equiv.) and compound **7** (20 mg, 0.02 mmol) were added to a *J*-Young NMR tube and dissolved in 0.5 mL of C_6D_6. The resulting solution was shaken to form a homogeneous solution, and the progress of the reaction was monitored by ^{1}H NMR spectroscopy at 25 °C. After 15 min HBPin (13.17 mg, 0.08 mmol, 4 equiv.) and 1-adamantanecorboxaldehyde (15.6 mg, 0.1 mmol, 5 equiv.) were added to perform a catalytic hydroboration with a catalytic load of 20 mol% and the progress of the reaction was monitored by ^{1}H NMR spectroscopy at 25 °C.

I. Spectroscopic Characterization

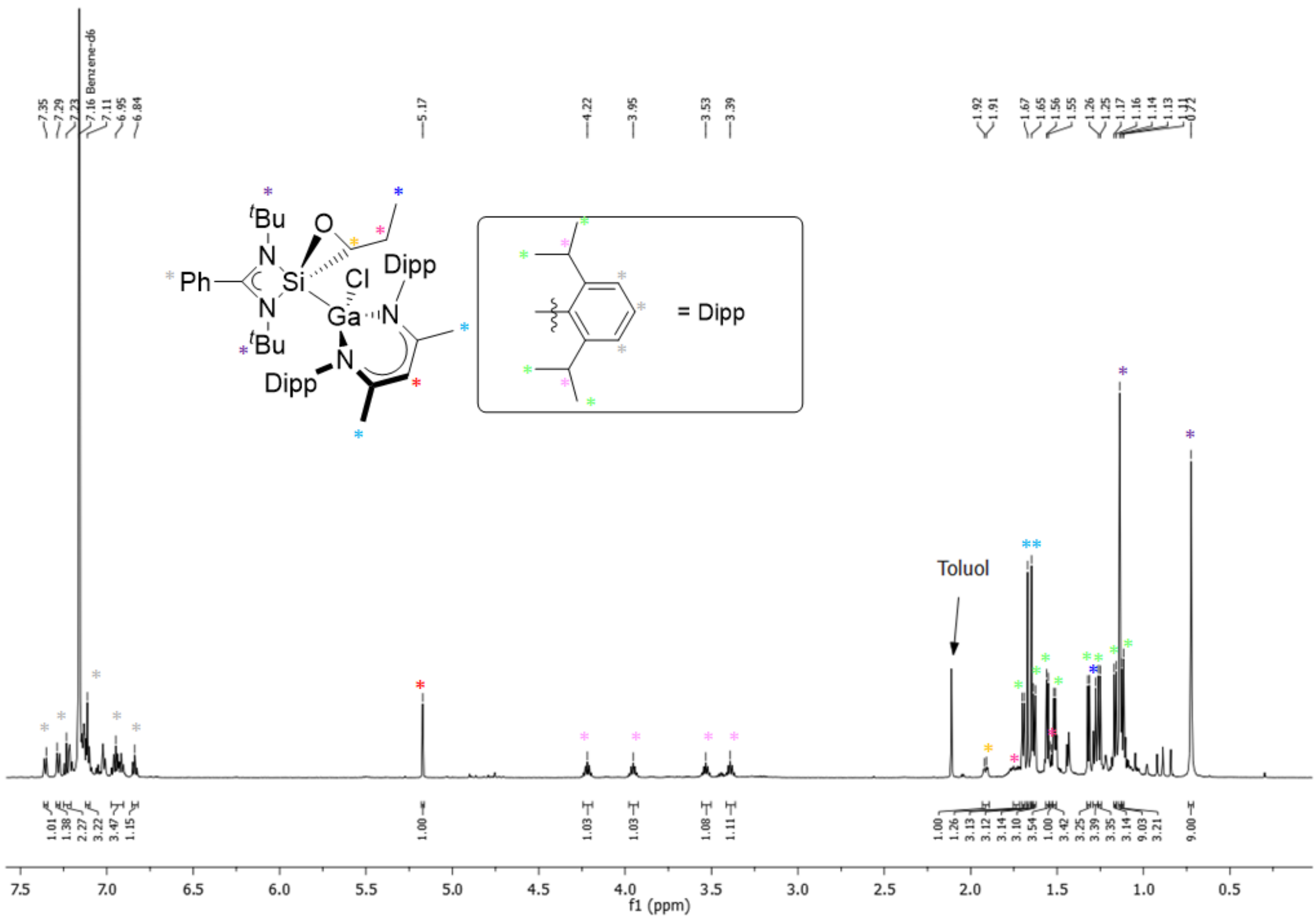

Figure S1. ^{1}H NMR spectrum of $L^{2}Si[OCHCH_2CH_3]Ga(Cl)L^{1}$ (**1**) in C_6D_6 at room temperature.

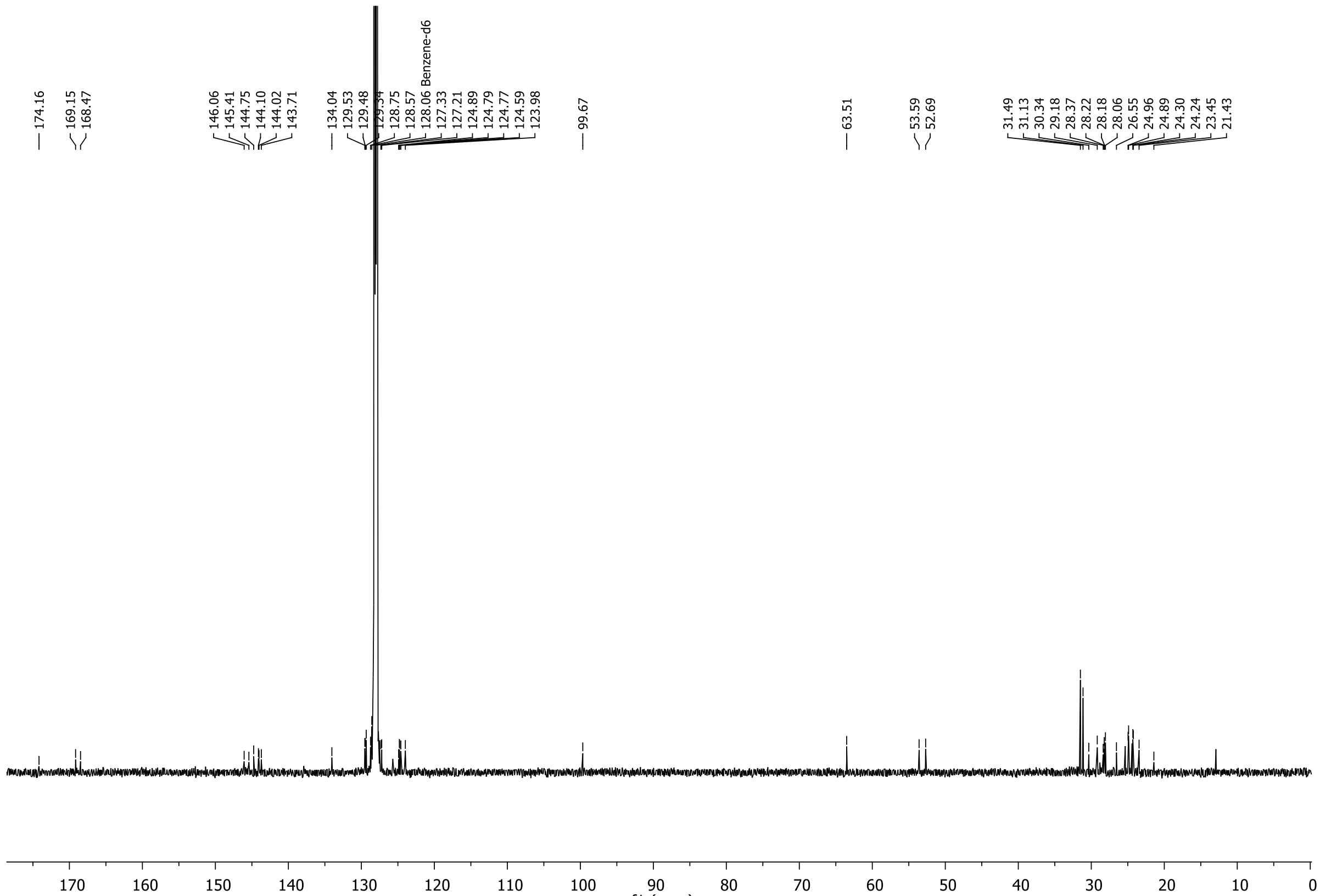

Figure S2. ^{13}C NMR spectrum of $L^{2}Si[OCHCH_2CH_3]Ga(Cl)L^{1}$ (**1**) in C_6D_6 at room temperature.

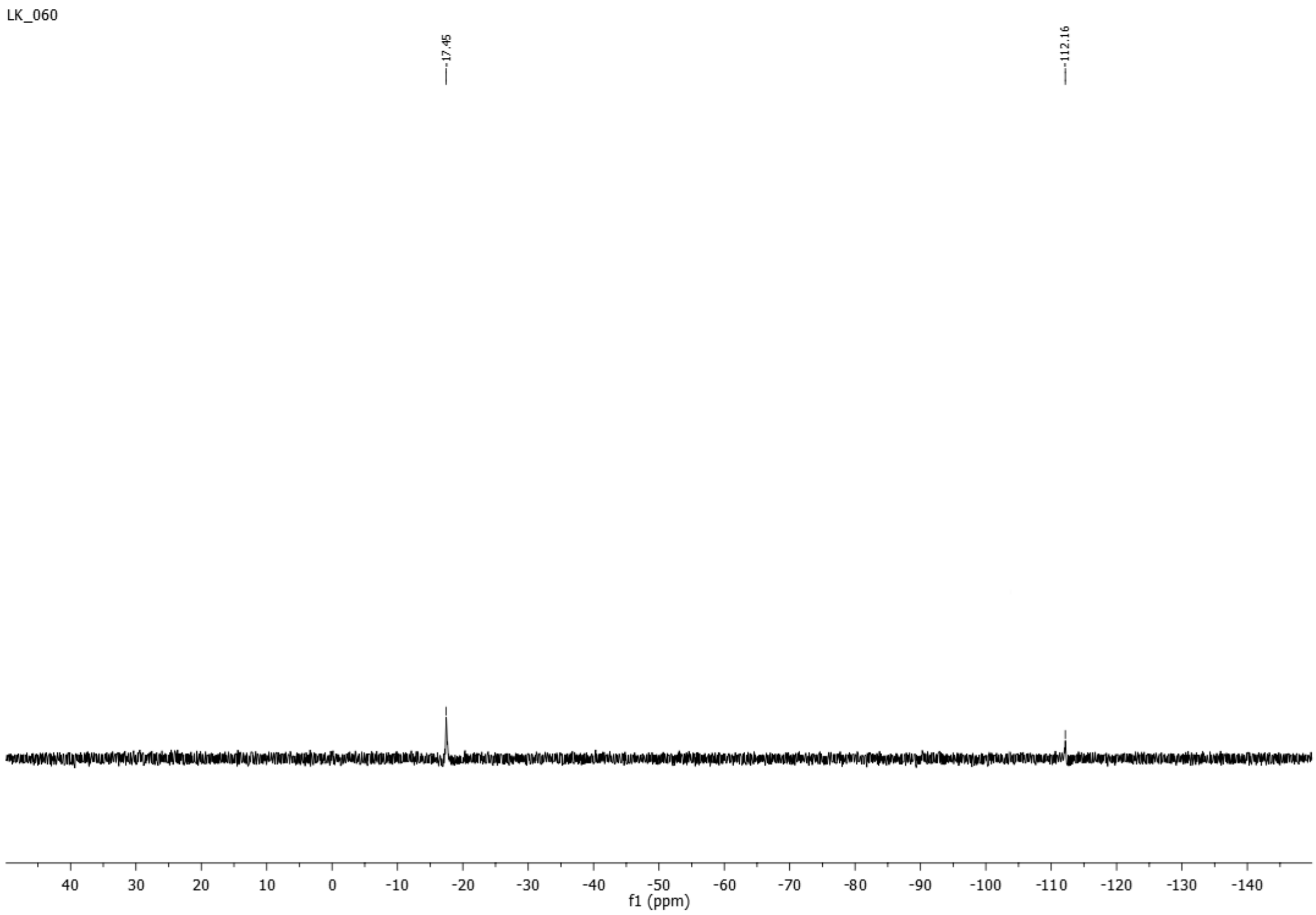

Figure S3. ^{29}Si NMR spectrum of $L^2Si[OCHCH_2CH_3]Ga(Cl)L^1$ (**1**) in $C_6\mathit{D}_6$ at room temperature, showing conversion of compound **1** to compound **3**.

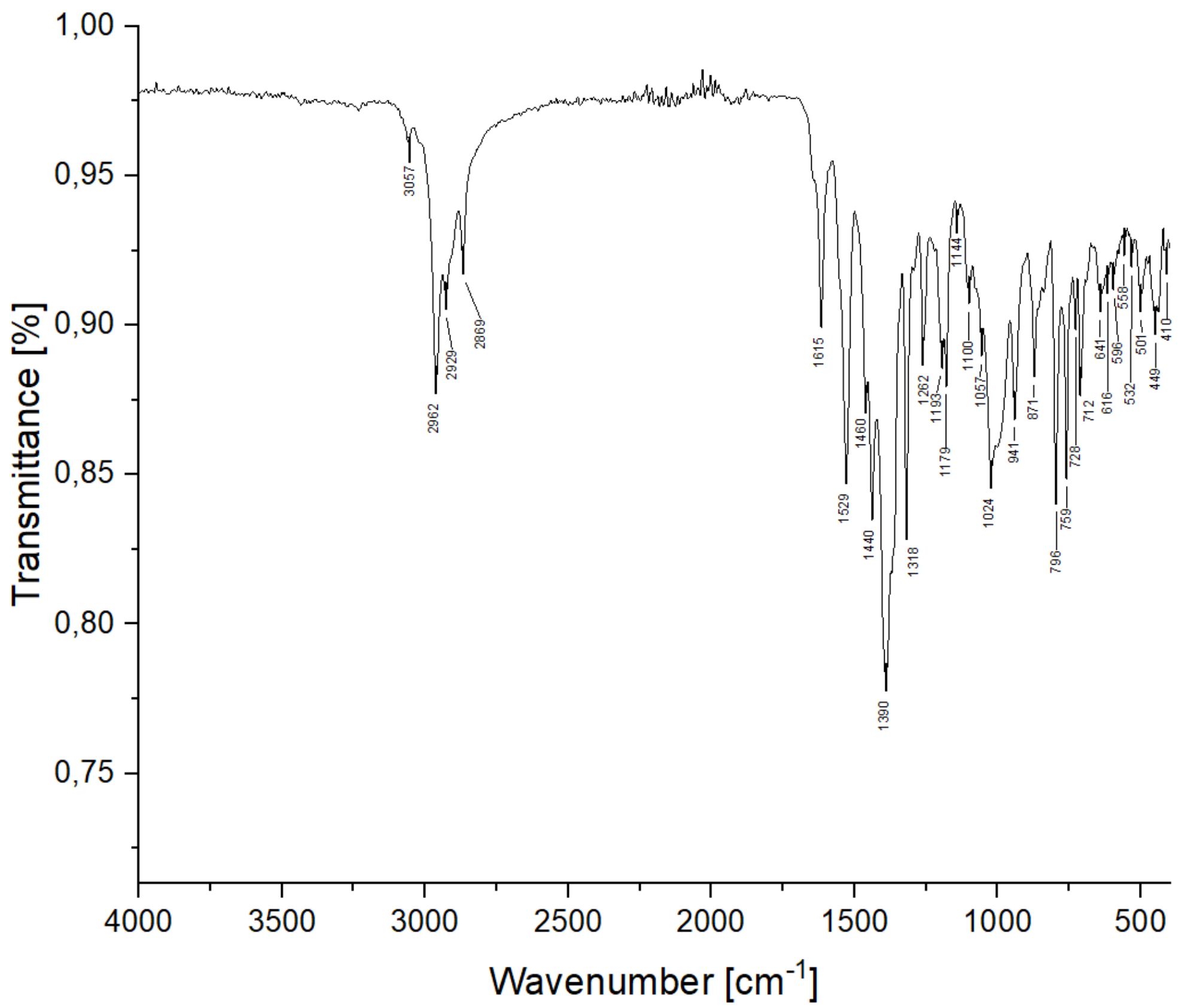

Figure S4. IR spectrum of $L^2Si[OCHCH_2CHMe_2]Ga(Cl)L^1$ (**1**).

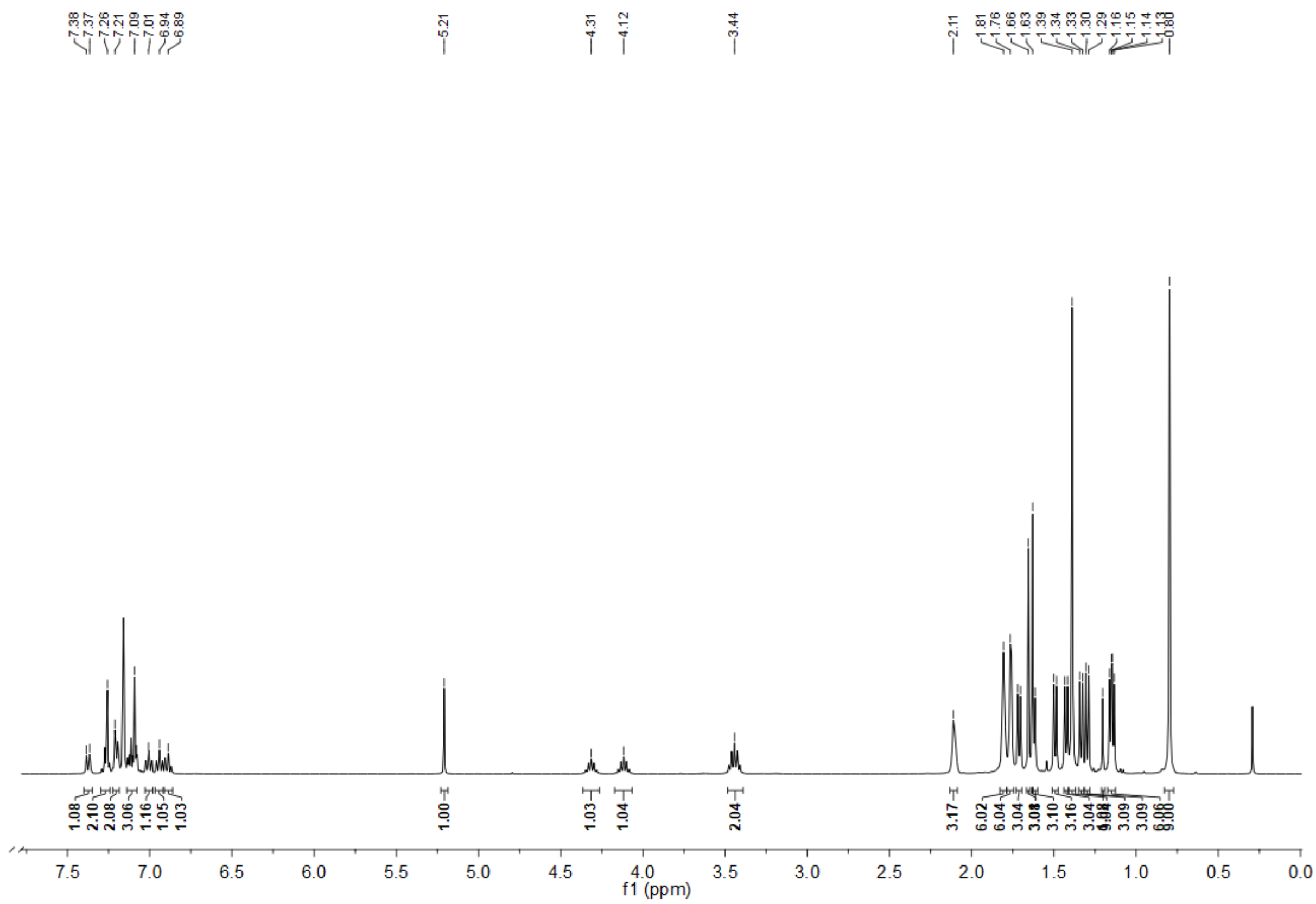

Figure S5. ^{1}H NMR spectrum of $L^2Si[OCHC_{10}H_{15}]Ga(Cl)L^1$ (**2**) in C_6D_6 at room temperature.

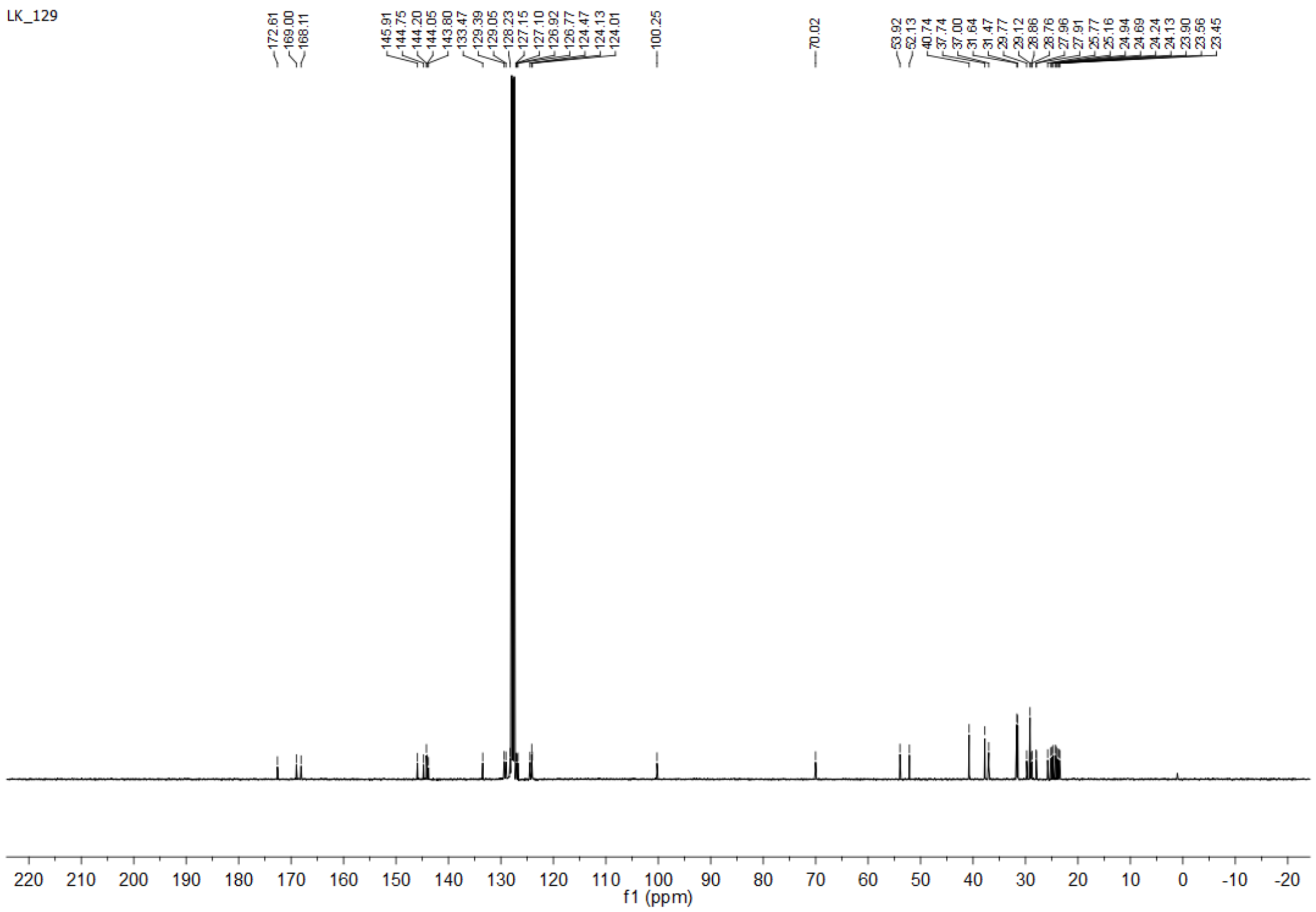

Figure S6. ^{13}C NMR spectrum of $L^2Si[OCHC_{10}H_{15}]Ga(Cl)L^1$ (**2**) in C_6D_6 at room temperature.

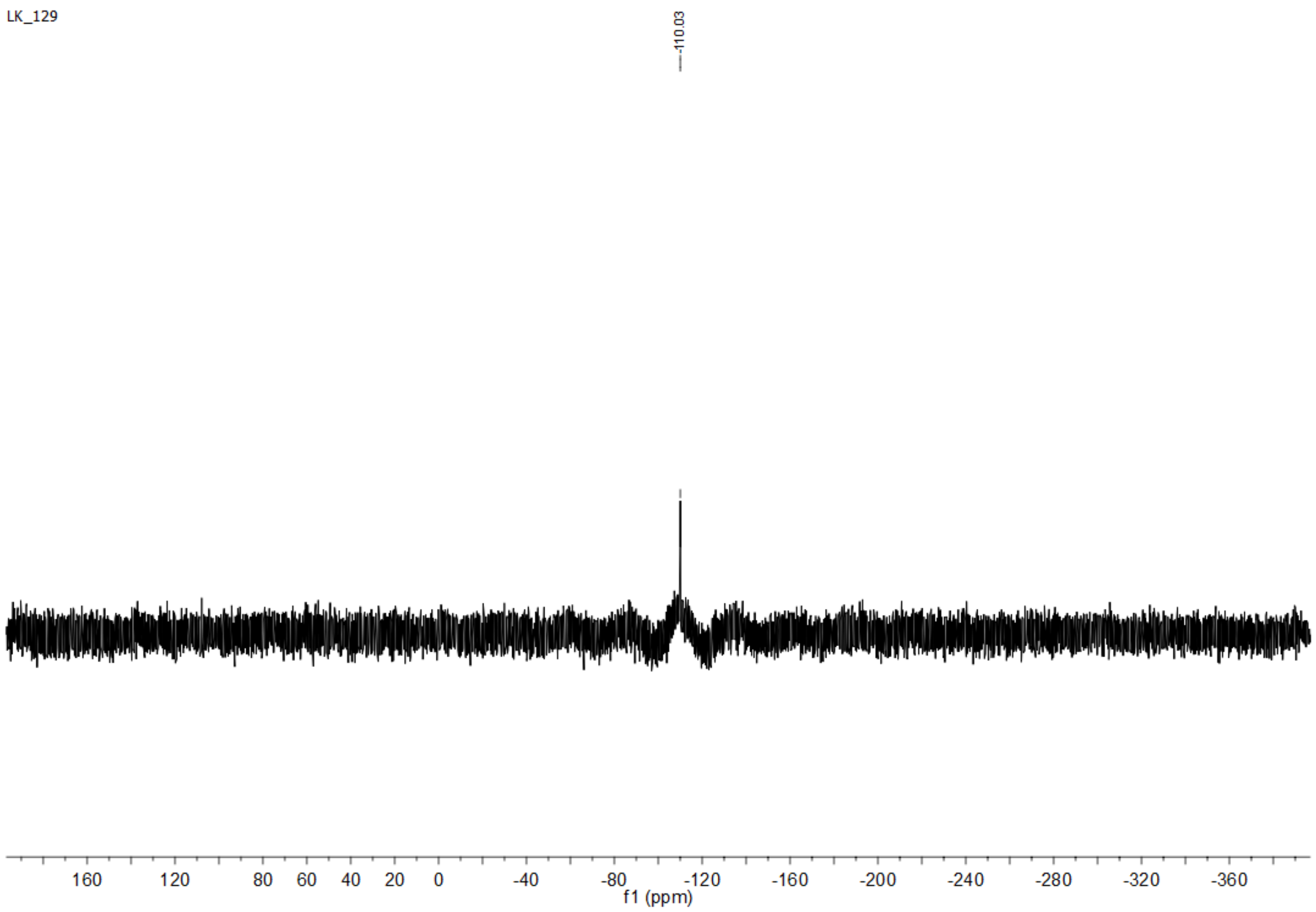

Figure S7. ^{29}Si NMR spectrum of $L^2Si[OCHC_{10}H_{15}]Ga(Cl)L^1$ (**2**) in C_6D_6 at room temperature.

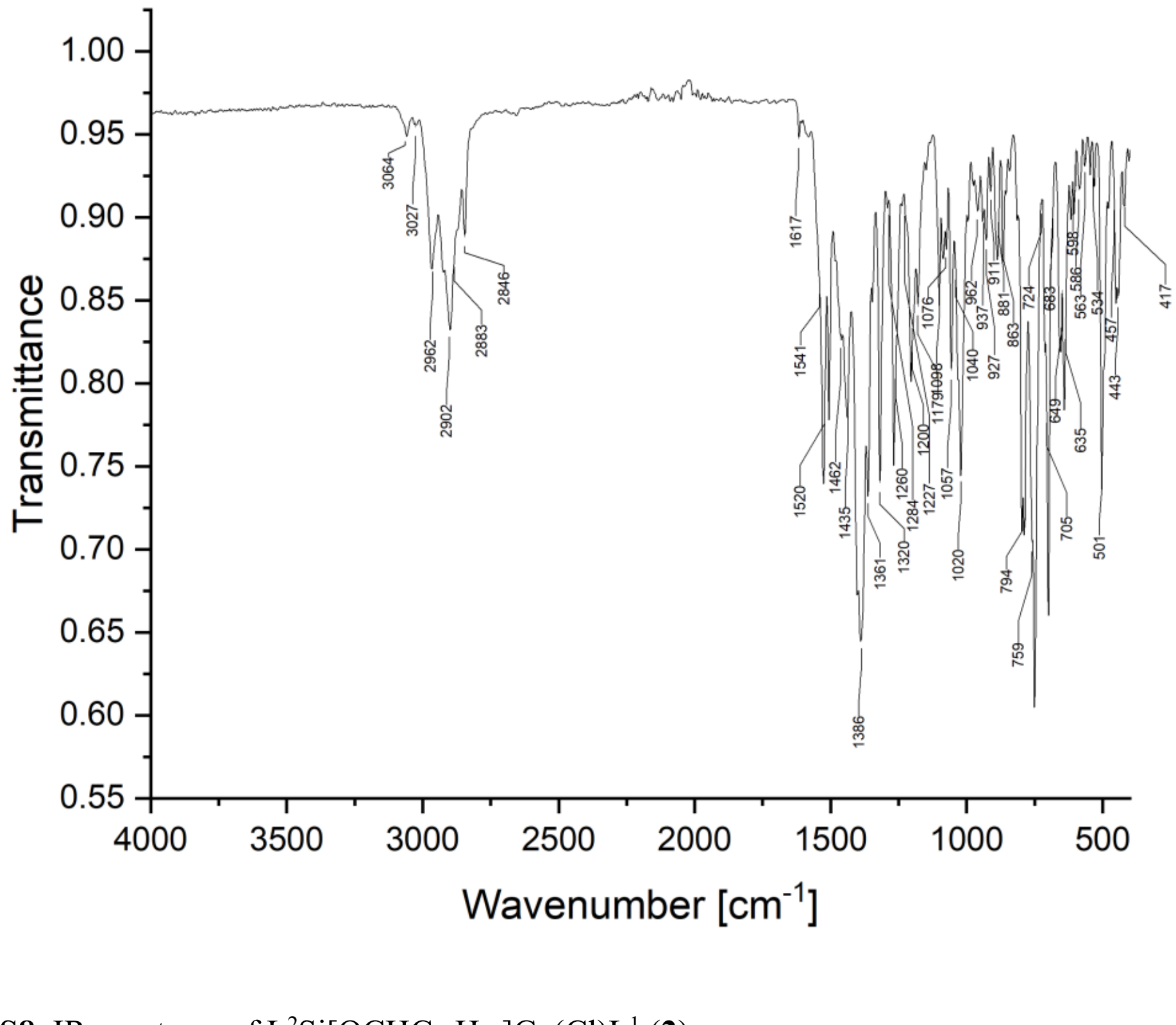

Figure S8. IR spectrum of $L^2Si[OCHC_{10}H_{15}]Ga(Cl)L^1$ (**2**).

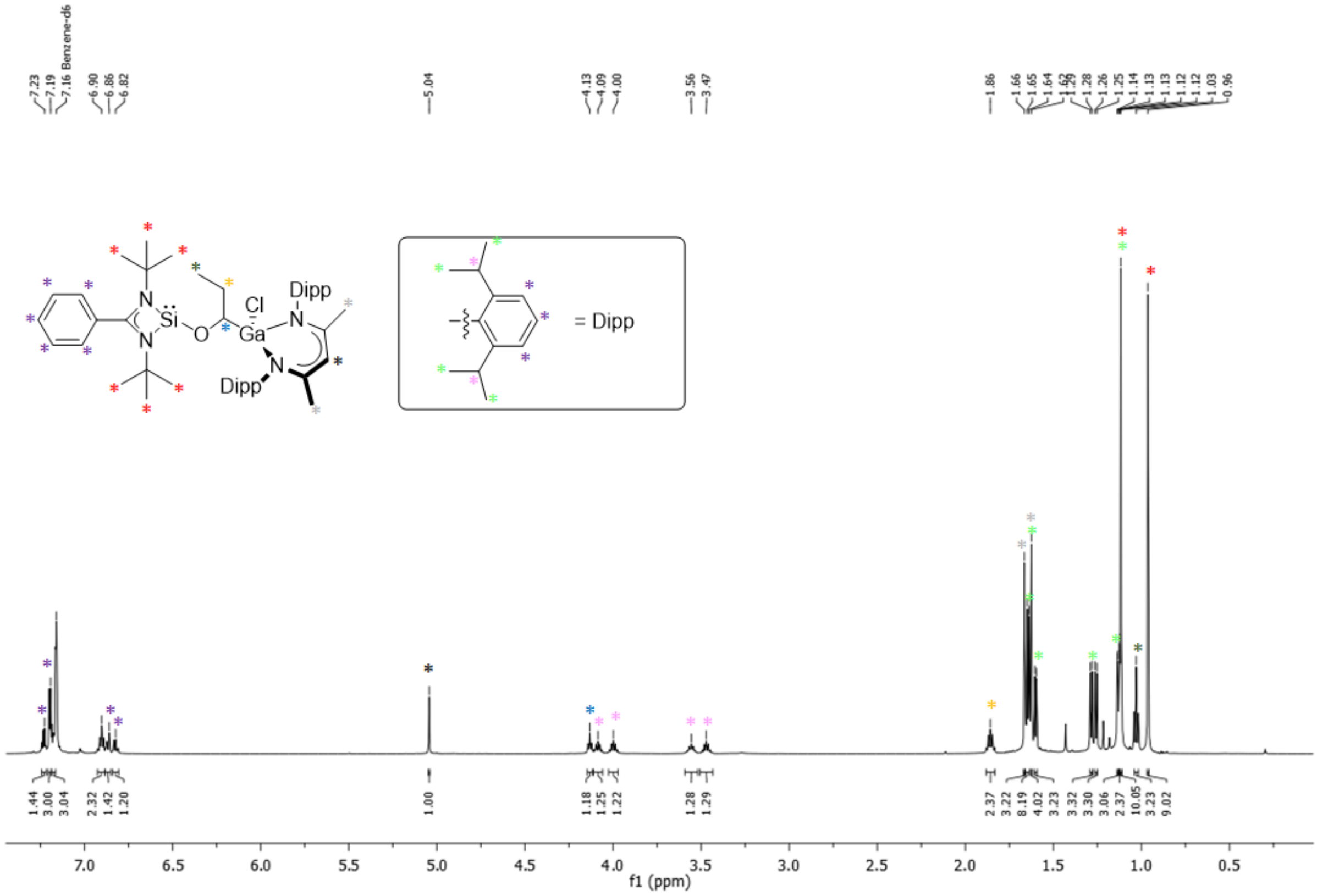

Figure S9. ^{1}H NMR spectrum of $L^2SiOCH[Ga(Cl)L^1]CH_2CH_3$ (**3**) in C_6D_6 at room temperature.

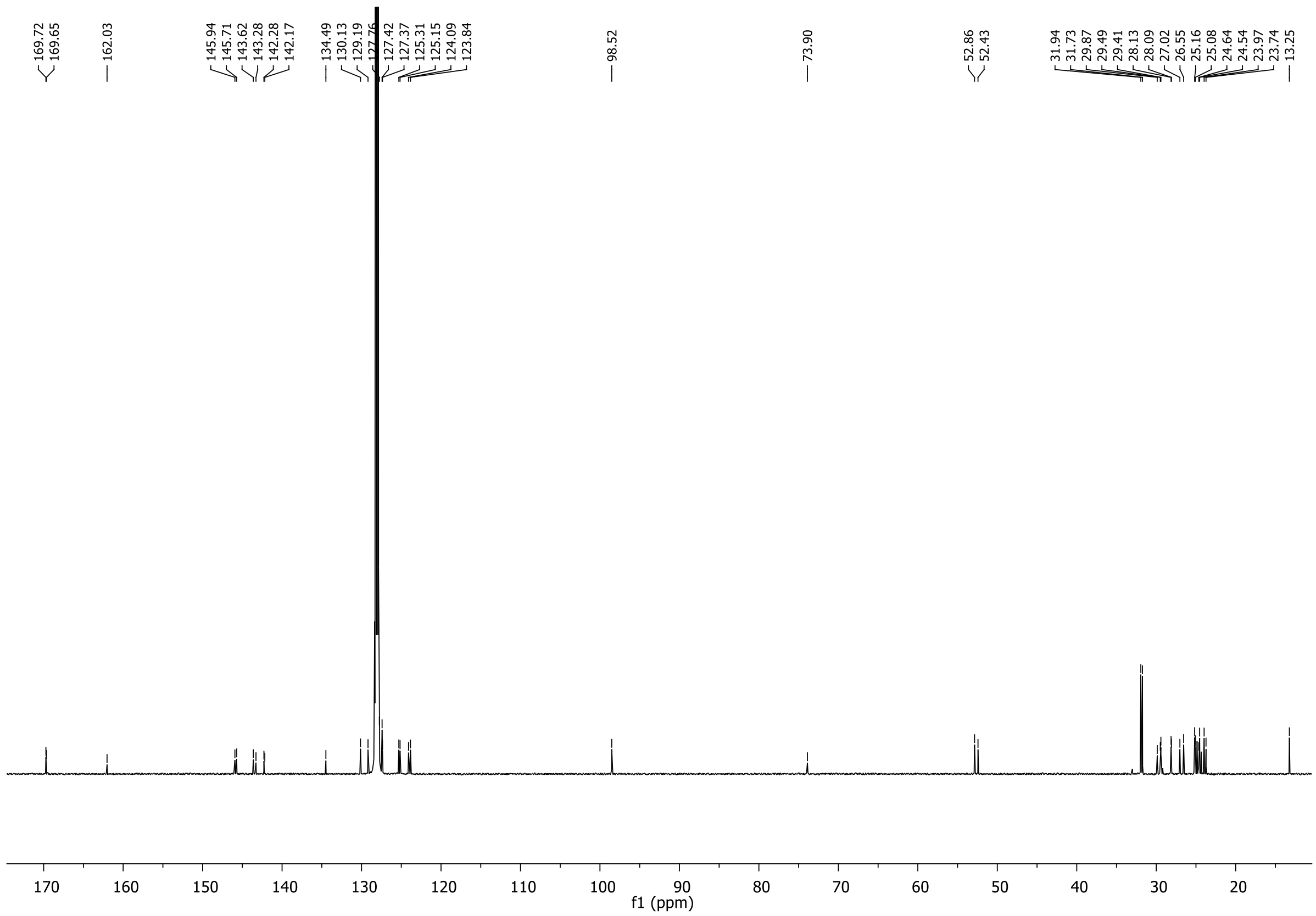

Figure S10. ^{13}C NMR spectrum of $L^2SiOCH[Ga(Cl)L^1]CH_2CH_3$ (**3**) in C_6D_6 at room temperature.

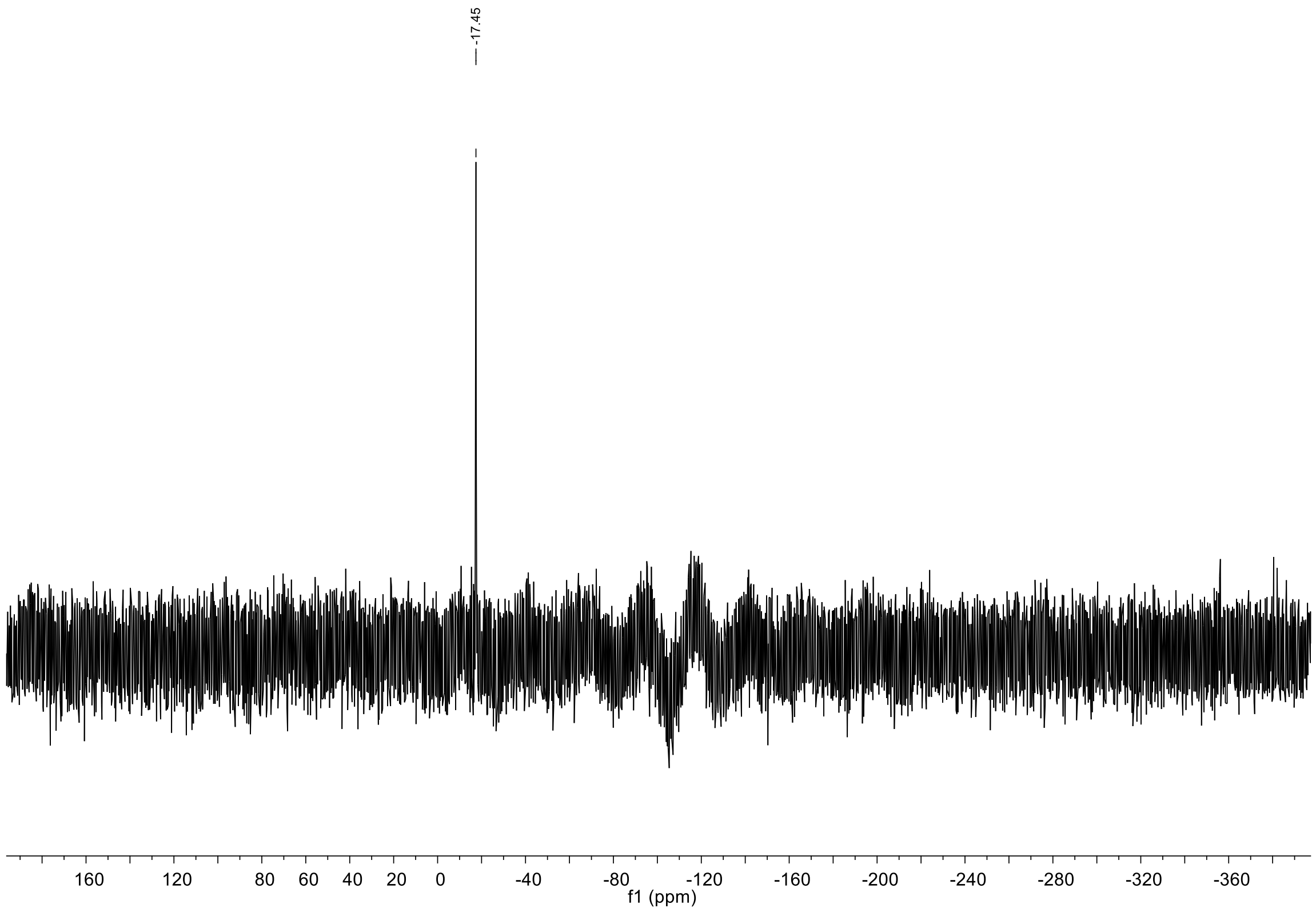

Figure S11. ^{29}Si NMR spectrum of $L^2SiOCH[Ga(Cl)L^1]CH_2CH_3$ (**3**) in C_6D_6 at room temperature.

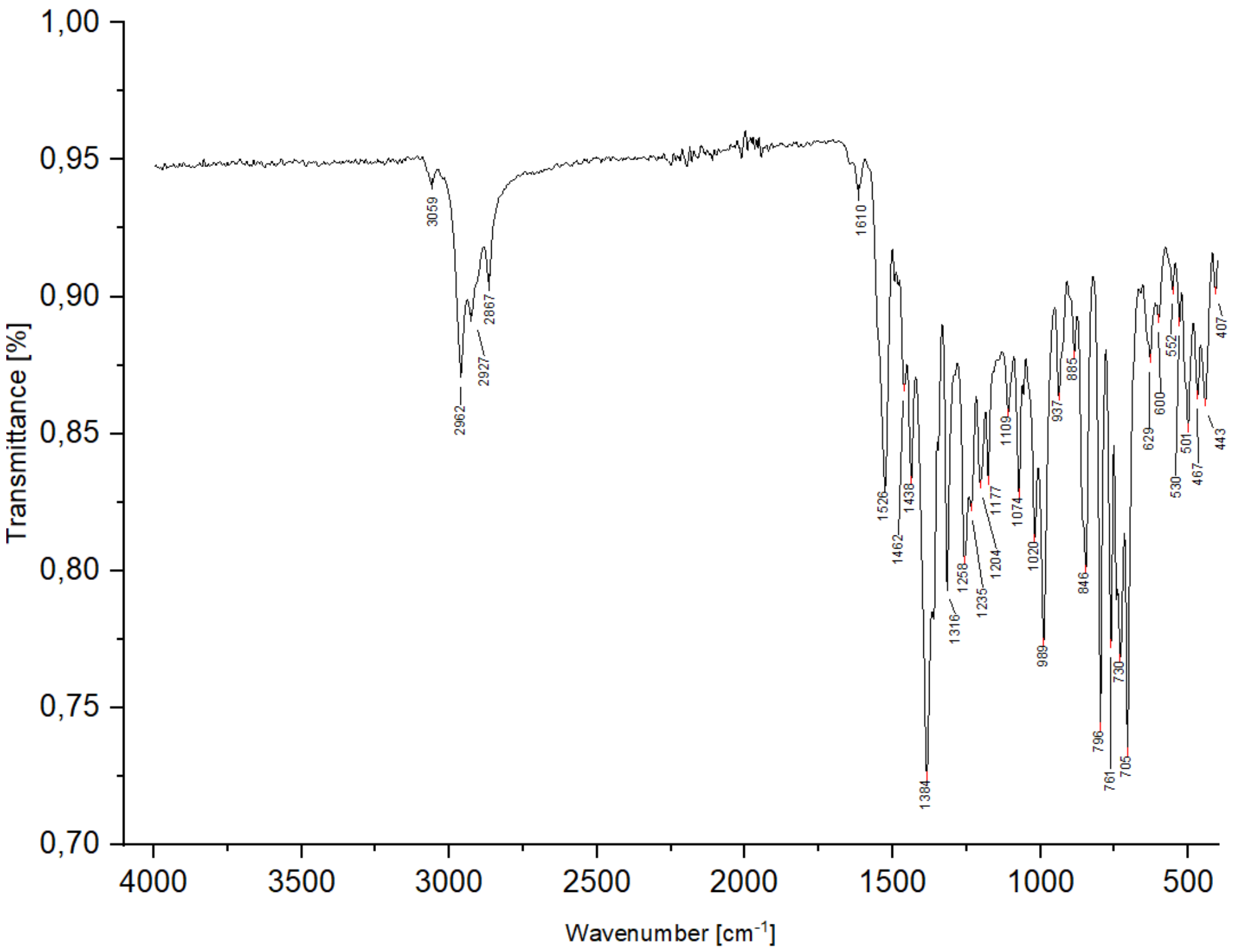

Figure S12. IR spectrum of $L^2SiOCH[Ga(Cl)L^1]CH_2CH_3$ (**3**).

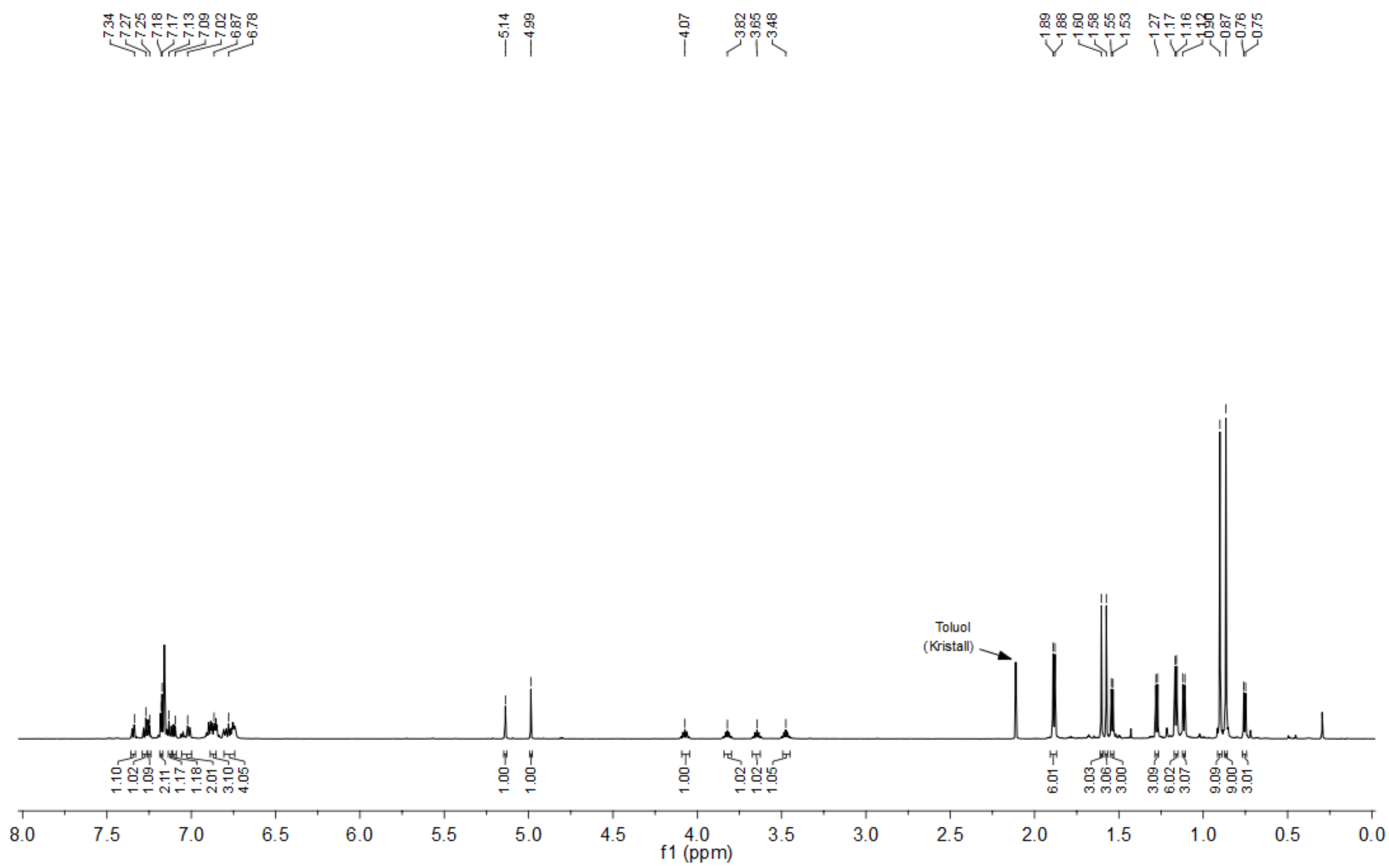

Figure S13. ^{1}H NMR spectrum of $L^2SiOCH[Ga(Cl)L^1]C_6H_5$ (**4**) in C_6D_6 at room temperature.

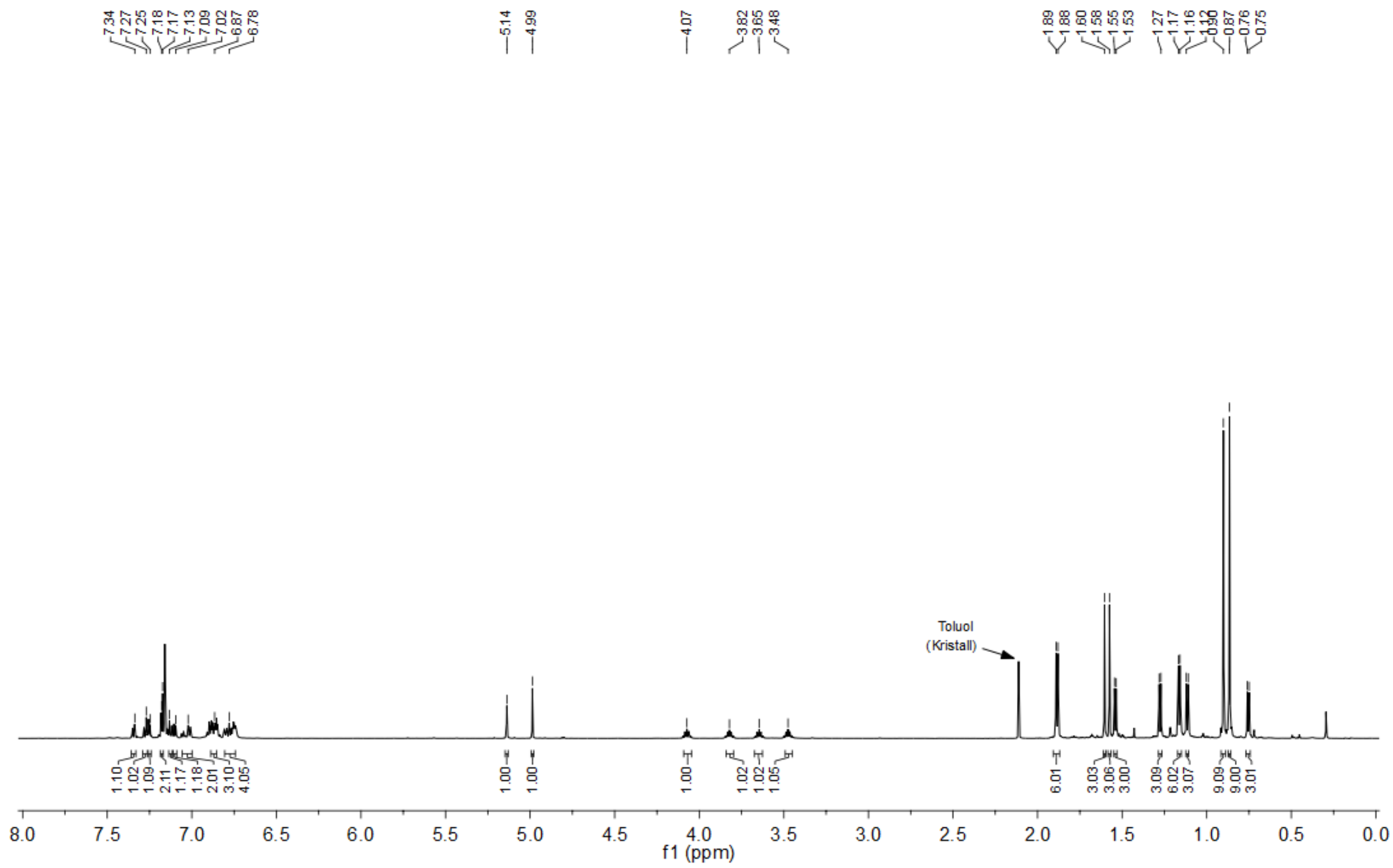

Figure S14. ^{13}C NMR spectrum of $L^2SiOCH[Ga(Cl)L^1]C_6H_5$ (**4**) in C_6D_6 at room temperature.

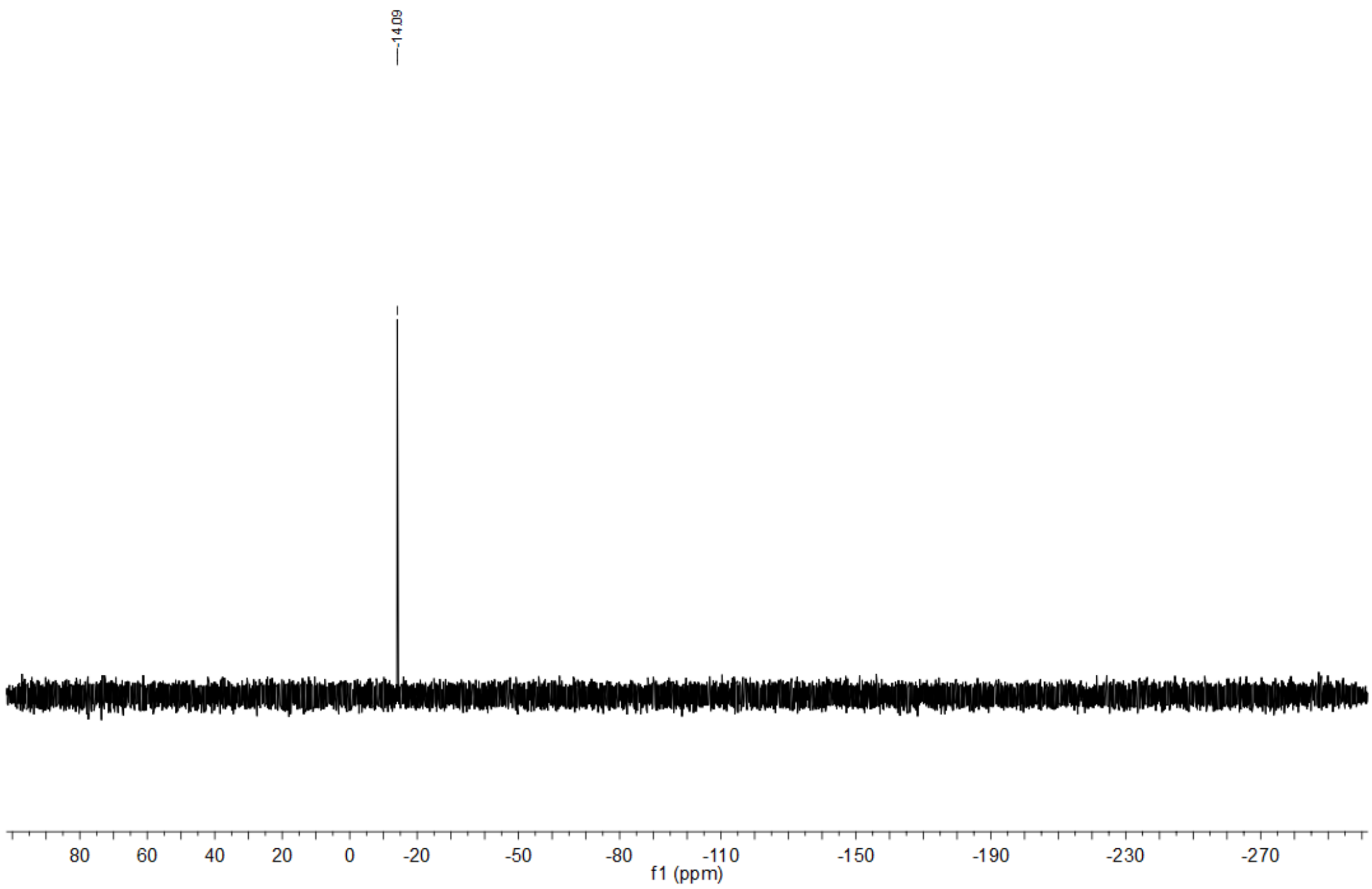

Figure S15. ^{29}Si NMR spectrum of $L^2SiOCH[Ga(Cl)L^1]C_6H_5$ (**4**) in C_6D_6 at room temperature.

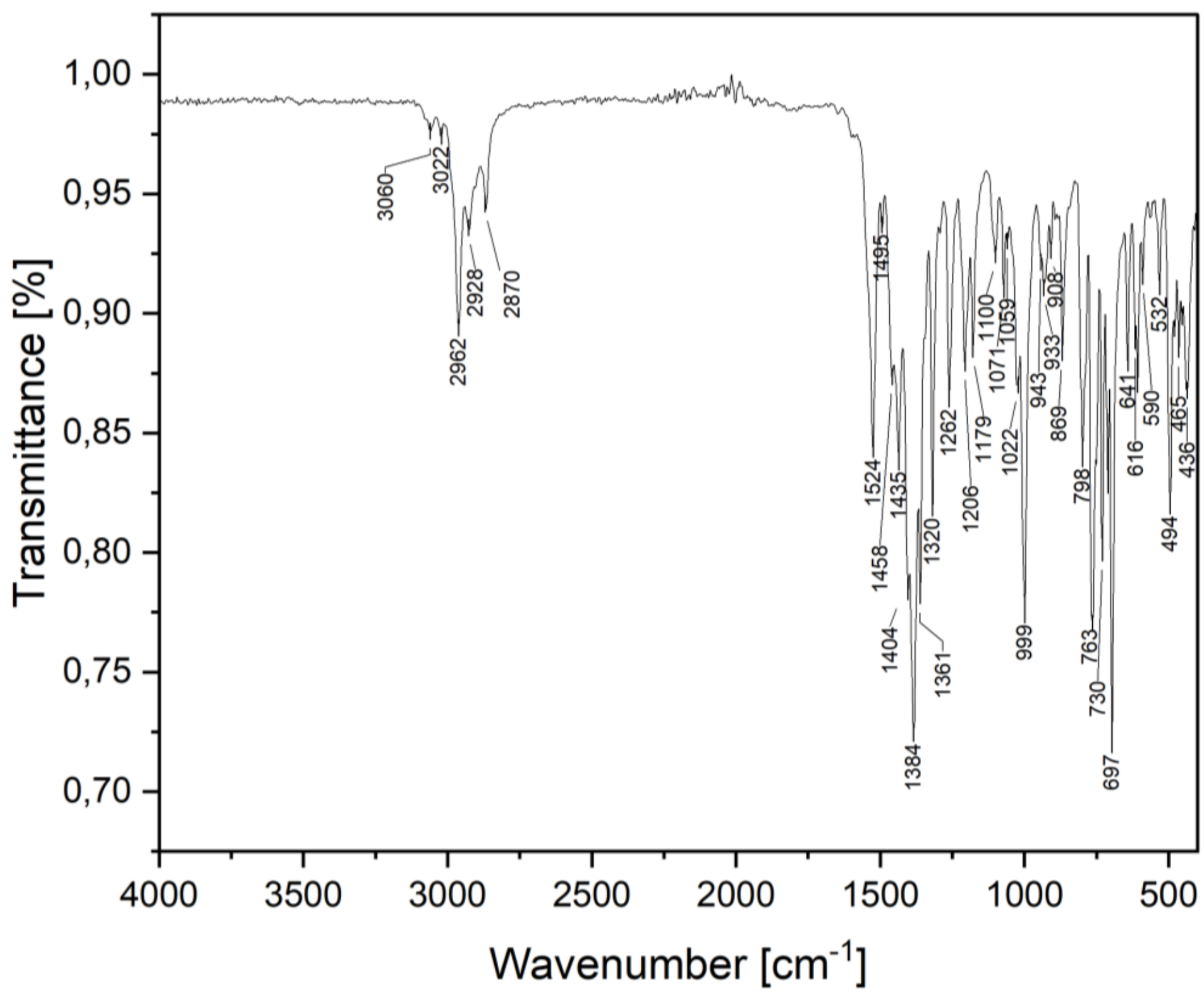

Figure S16. IR spectrum of $L^2SiOCH[Ga(Cl)L^1]C_6H_5$ (**4**).

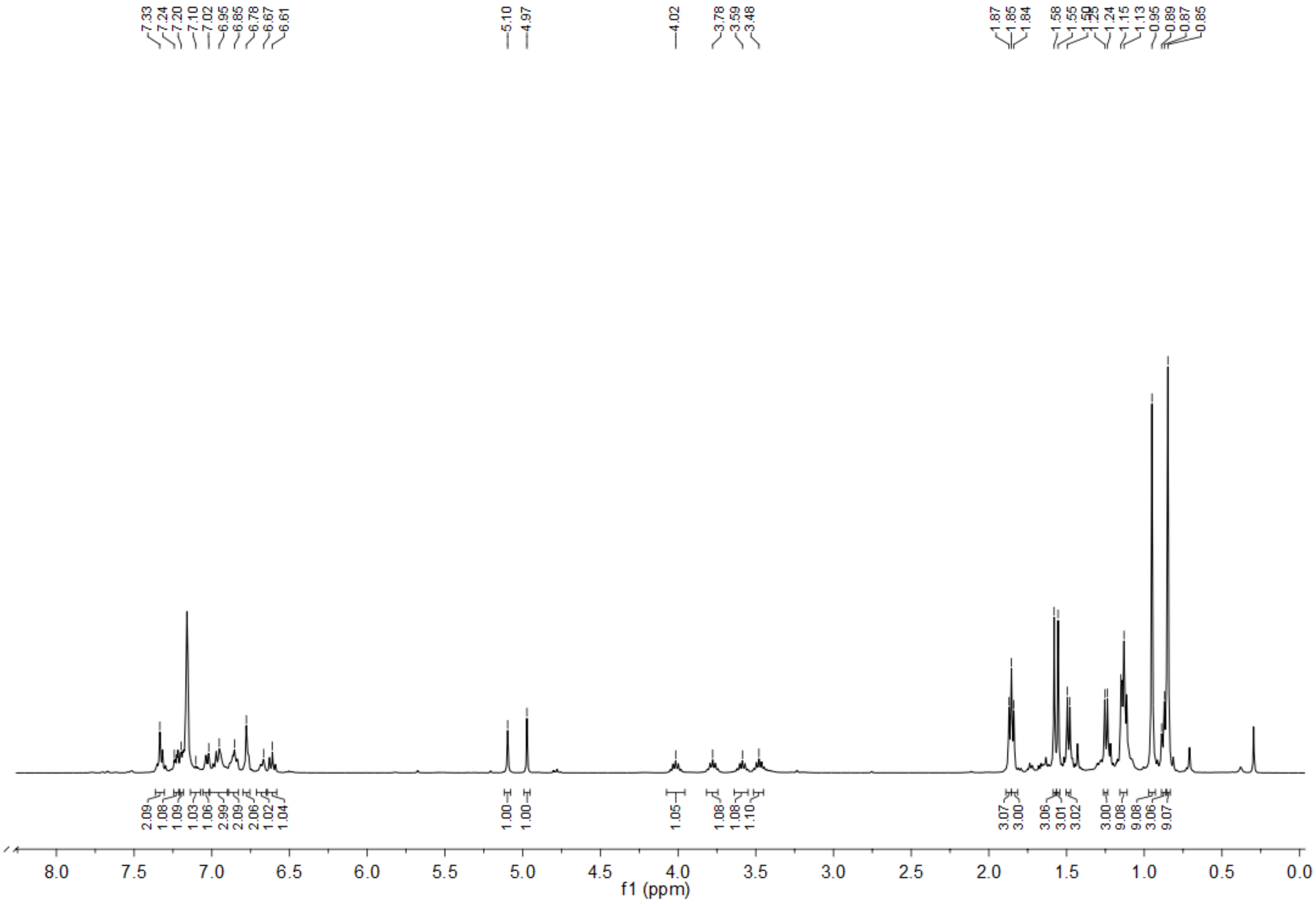

Figure S17. ^{1}H NMR spectrum of $L^2SiOCH[Ga(Cl)L^1]C_6H_4Br$ (**5**) in C_6D_6 at room temperature.

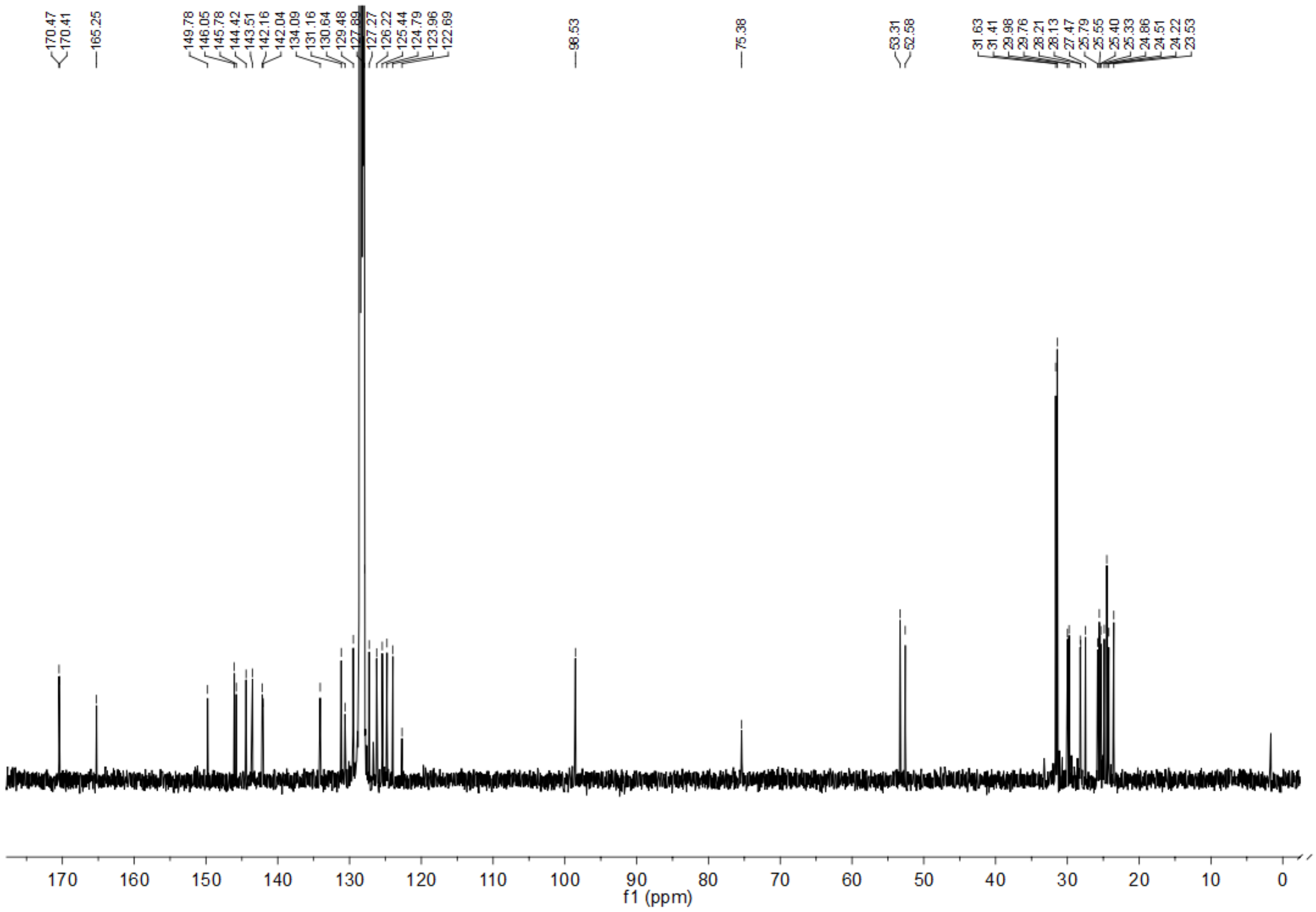

Figure S18. ^{13}C NMR spectrum of $L^2SiOCH[Ga(Cl)L^1]C_6H_4Br$ (**5**) in C_6D_6 at room temperature.

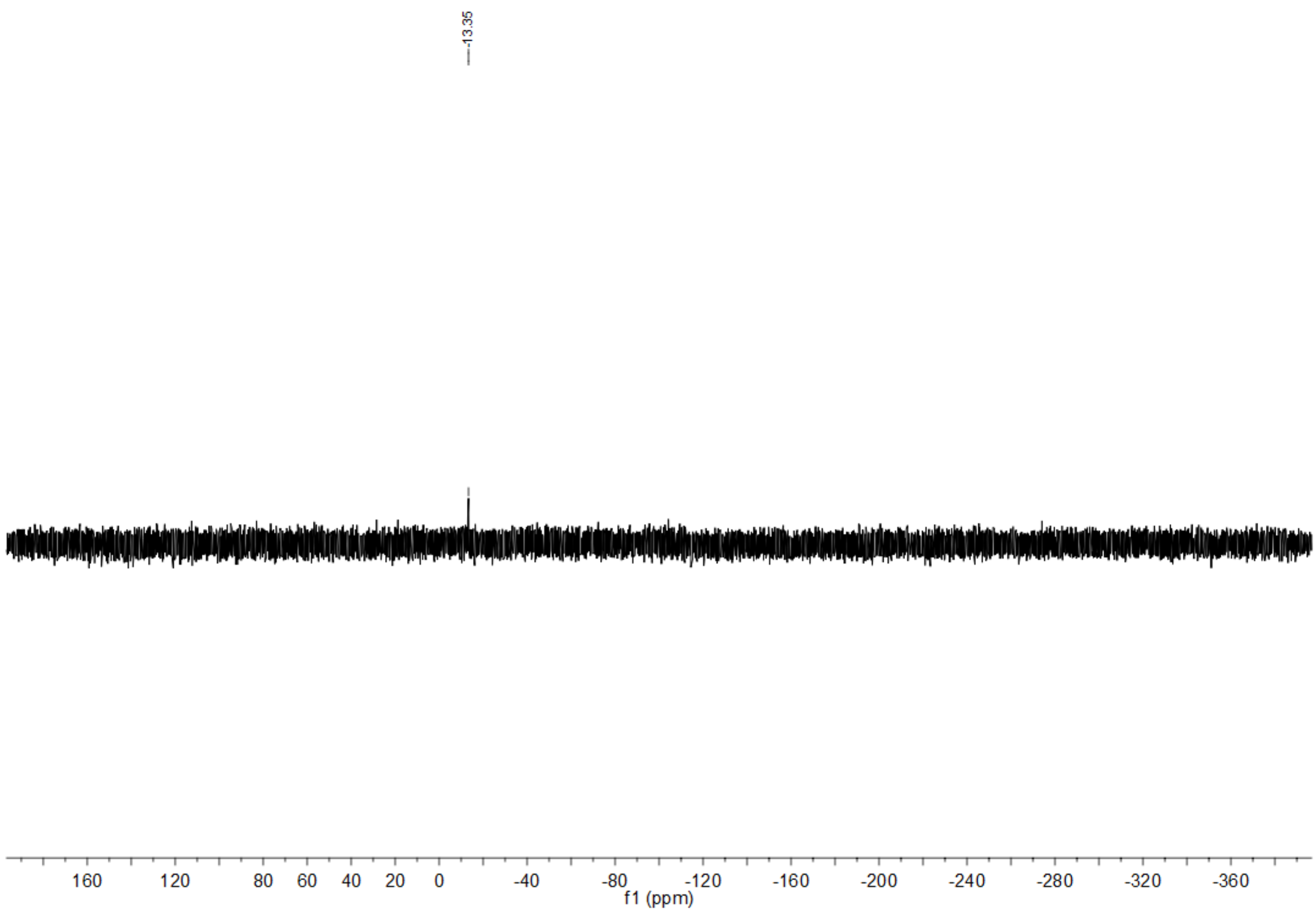

Figure S19. ^{29}Si NMR spectrum of $L^2SiOCH[Ga(Cl)L^1]C_6H_4Br$ (**5**) in C_6D_6 at room temperature.

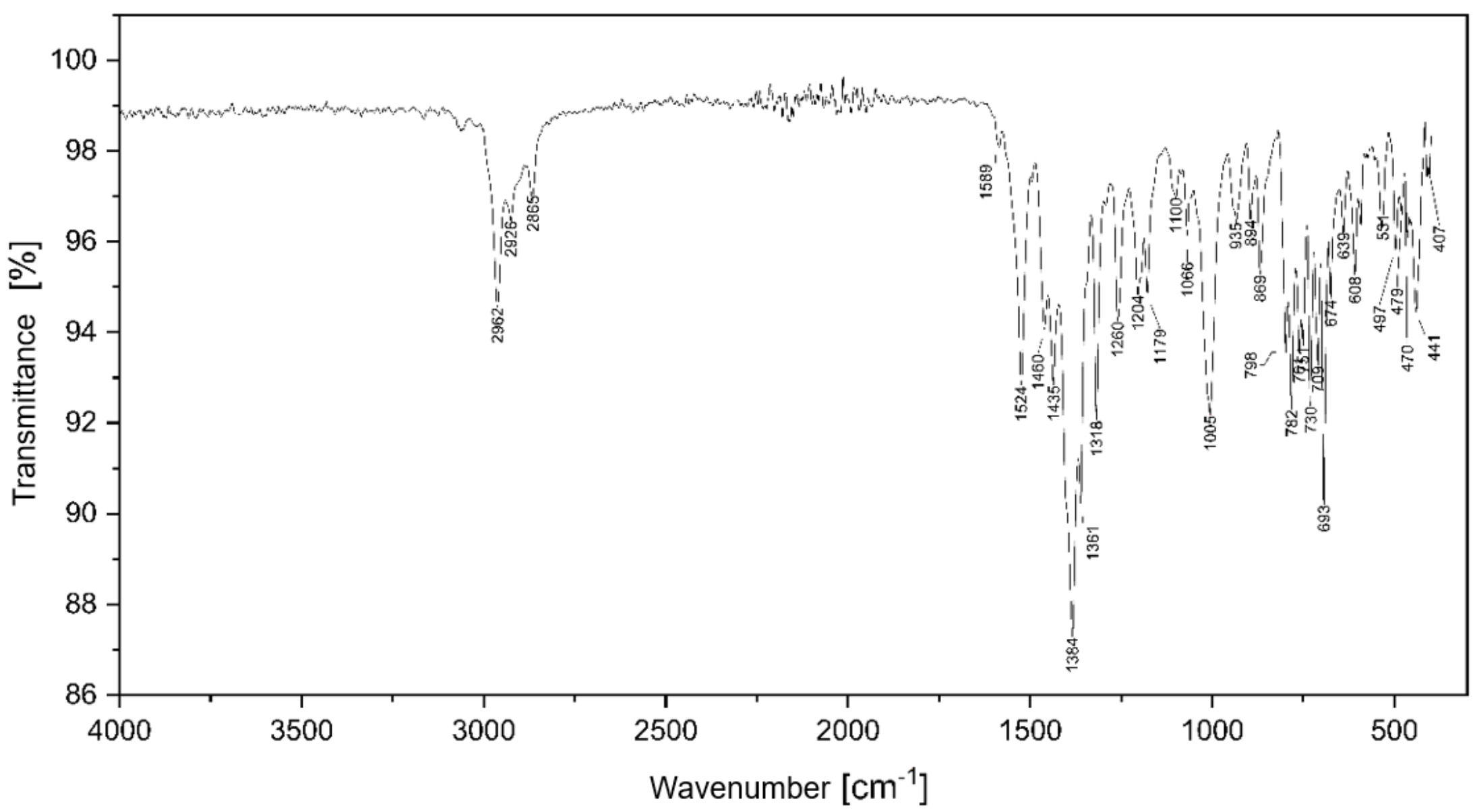

Figure S20. IR spectrum of $L^2SiOCH[Ga(Cl)L^1]C_6H_4Br$ (**5**).

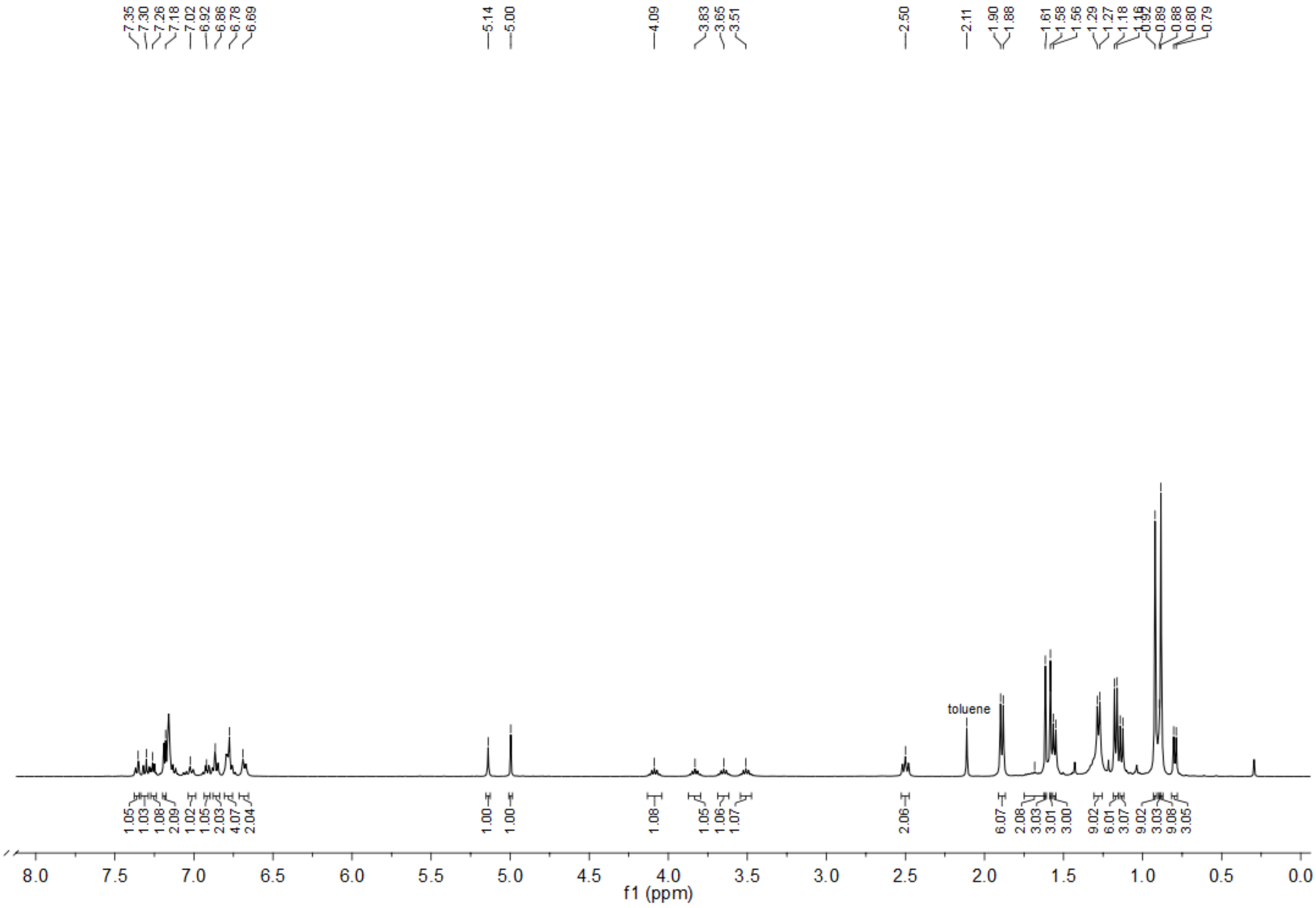

Figure S21. ^{1}H NMR spectrum of $L^2SiOCH[Ga(Cl)L^1]C_6H_4C_6H_{13}$ (**6**) in C_6D_6 at room temperature.

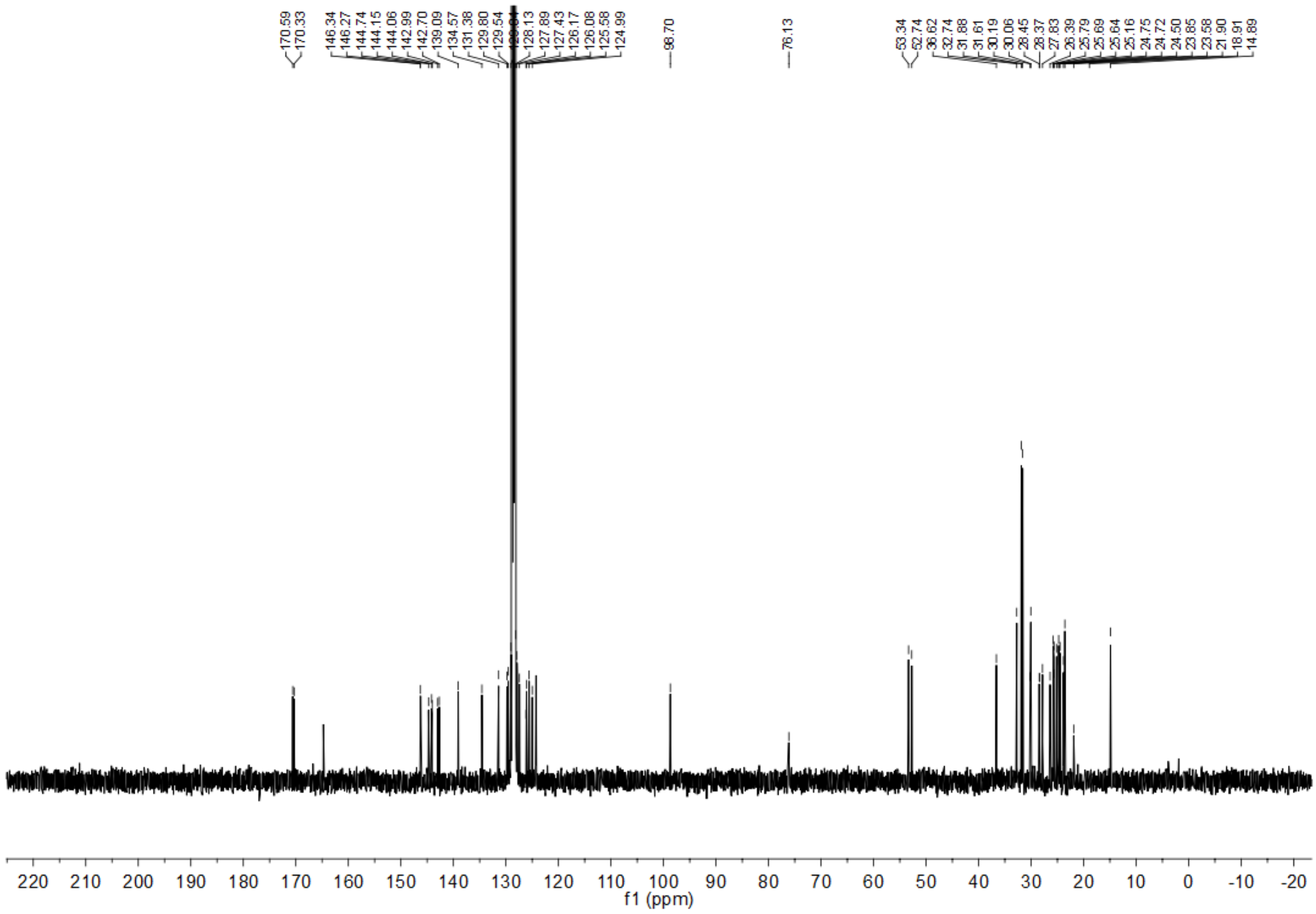

Figure S22. ^{13}C NMR spectrum of $L^2SiOCH[Ga(Cl)L^1]C_6H_4C_6H_{13}$ (**6**) in C_6D_6 at room temperature.

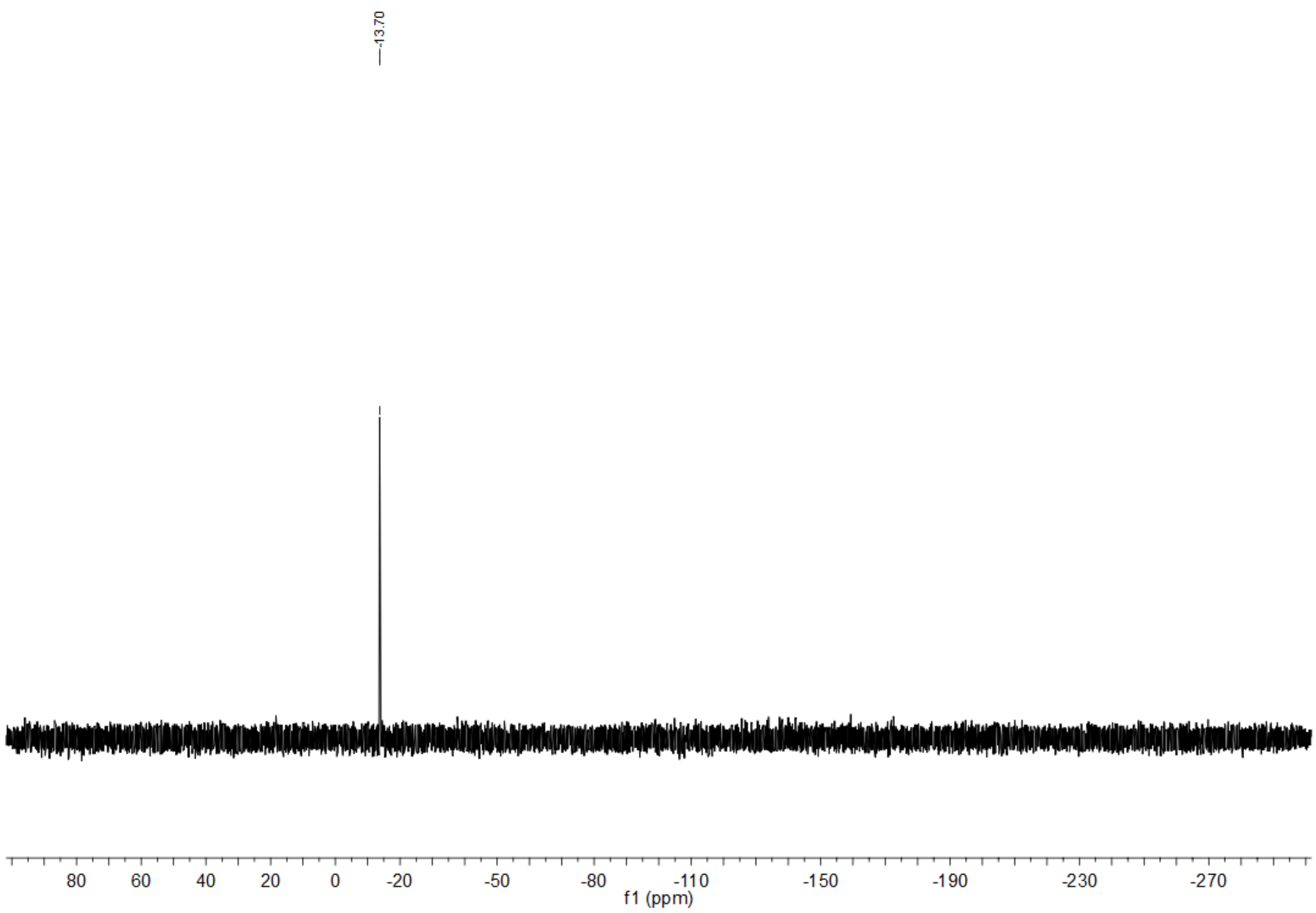

Figure S23. ^{29}Si NMR spectrum of $L^2SiOCH[Ga(Cl)L^1]C_6H_4C_6H_{13}$ (**6**) in C_6D_6 at room temperature.

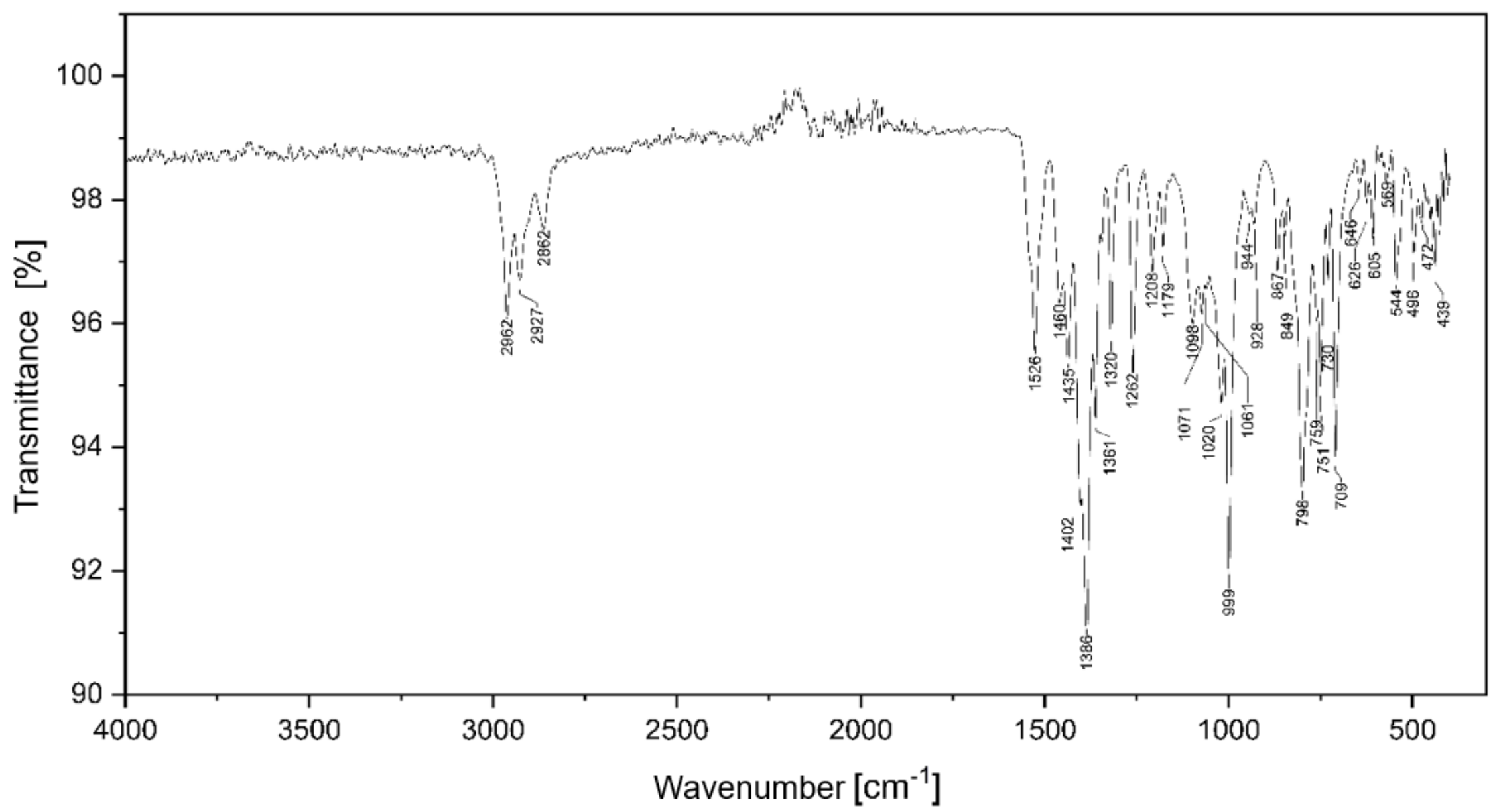

Figure S24. IR spectrum of $L^2SiOCH[Ga(Cl)L^1]C_6H_4C_6H_{13}$ (**6**).

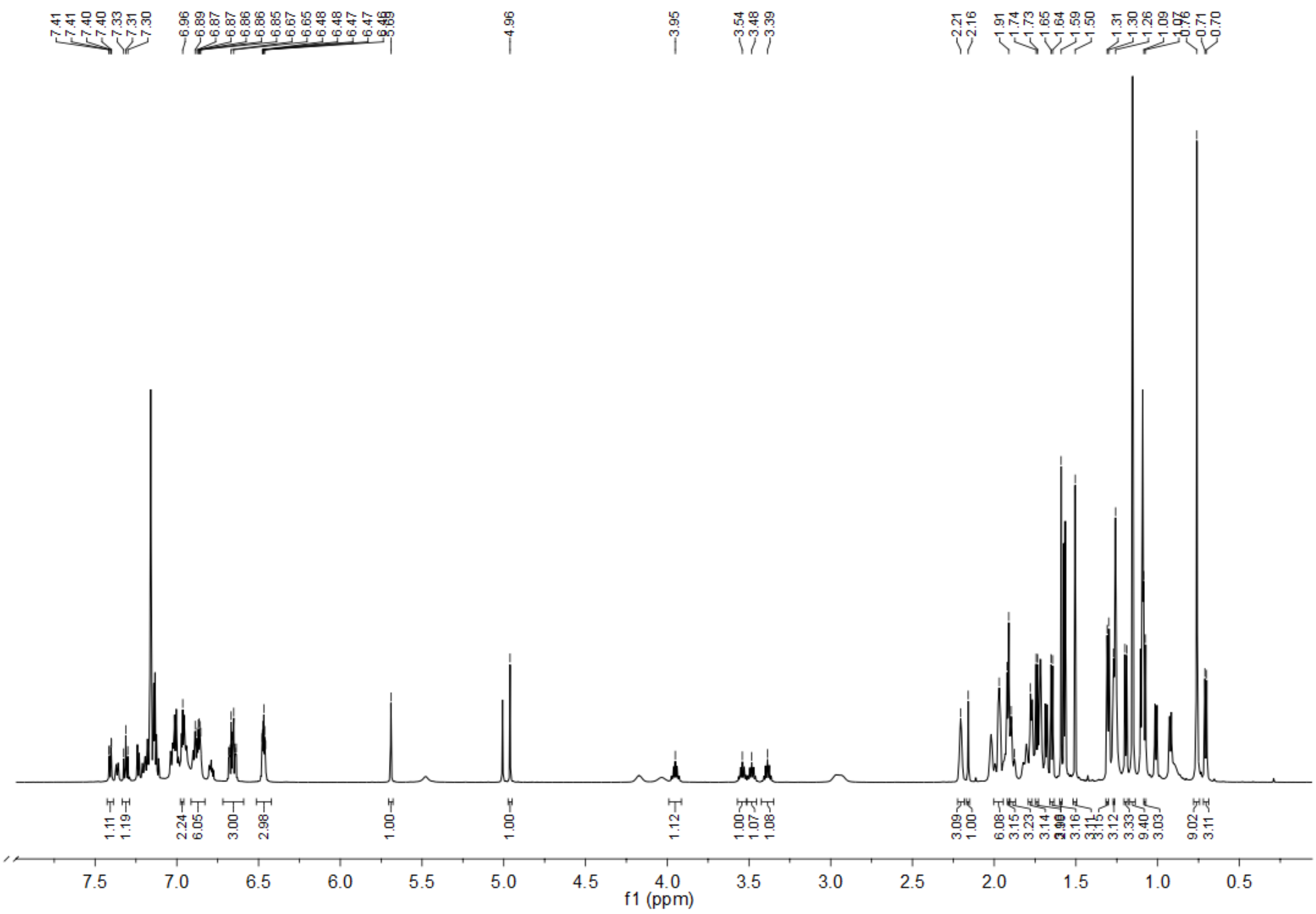

Figure S25. ^{1}H NMR spectrum of $L^2Si[OCHC_{10}H_{15}]OCH[Ga(Cl)L^1]C_6H_5$ (**7**) in C_6D_6 with diastereomere **7a** marked at room temperature.

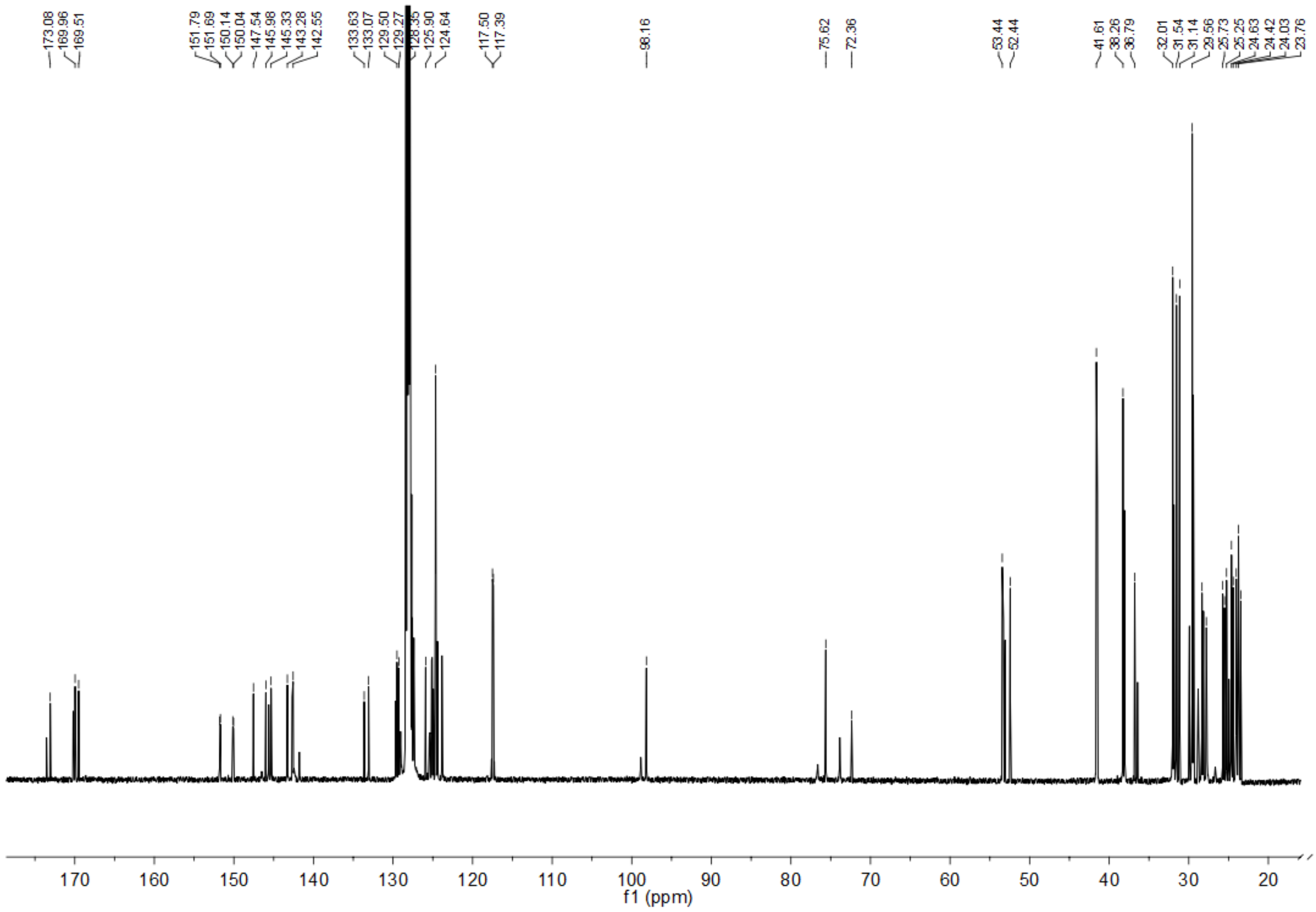

Figure S26. ^{13}C NMR spectrum of $L^2Si[OCHC_{10}H_{15}]OCH[Ga(Cl)L^1]C_6H_5$ (**7**) in C_6D_6 with diastereomere **7a** marked at room temperature.

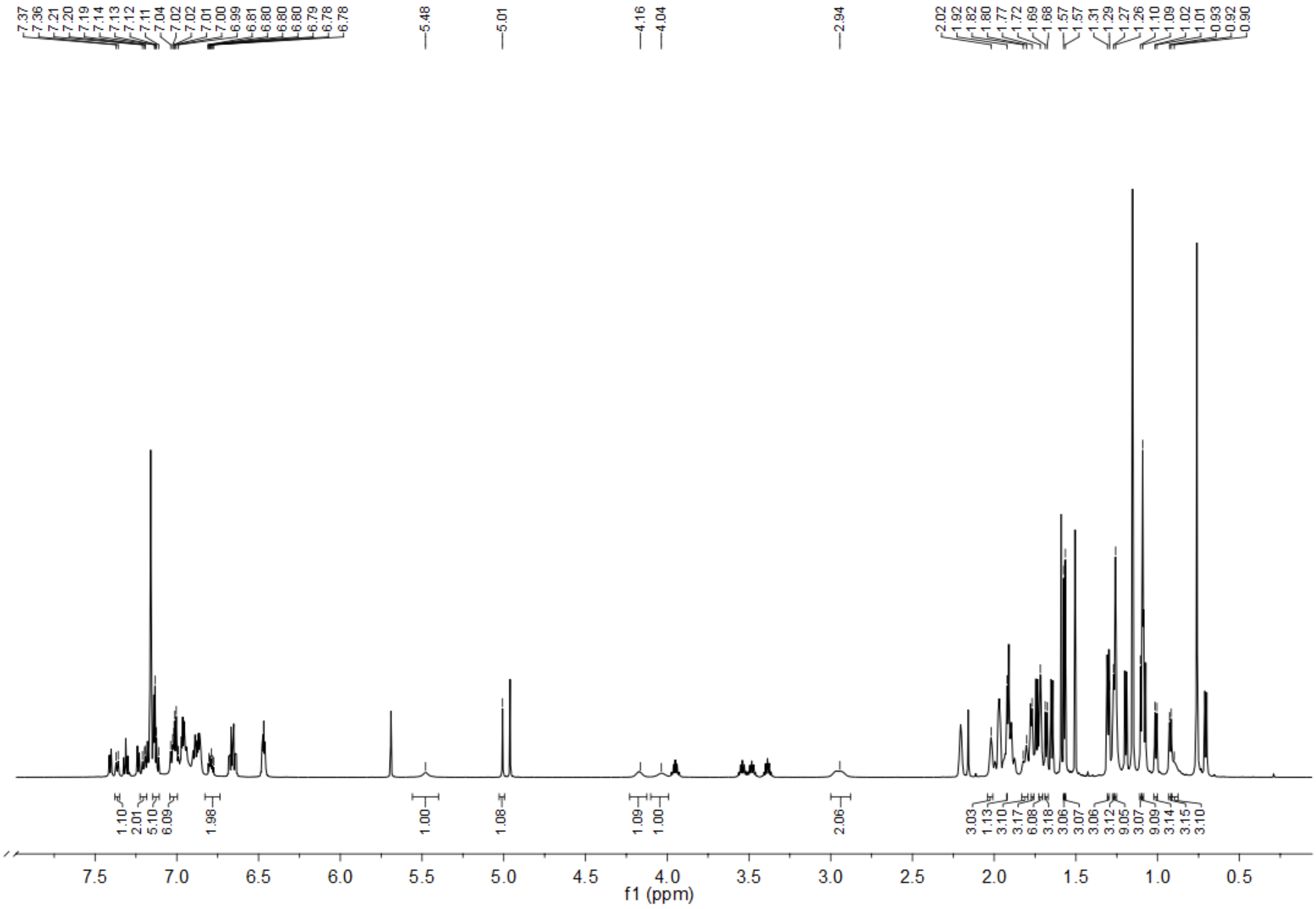

Figure S27. ^{1}H NMR spectrum of $L^2Si[OCHC_{10}H_{15}]OCH[Ga(Cl)L^1]C_6H_5$ (**7**) in C_6D_6 with diastereomere **7b** marked at room temperature.

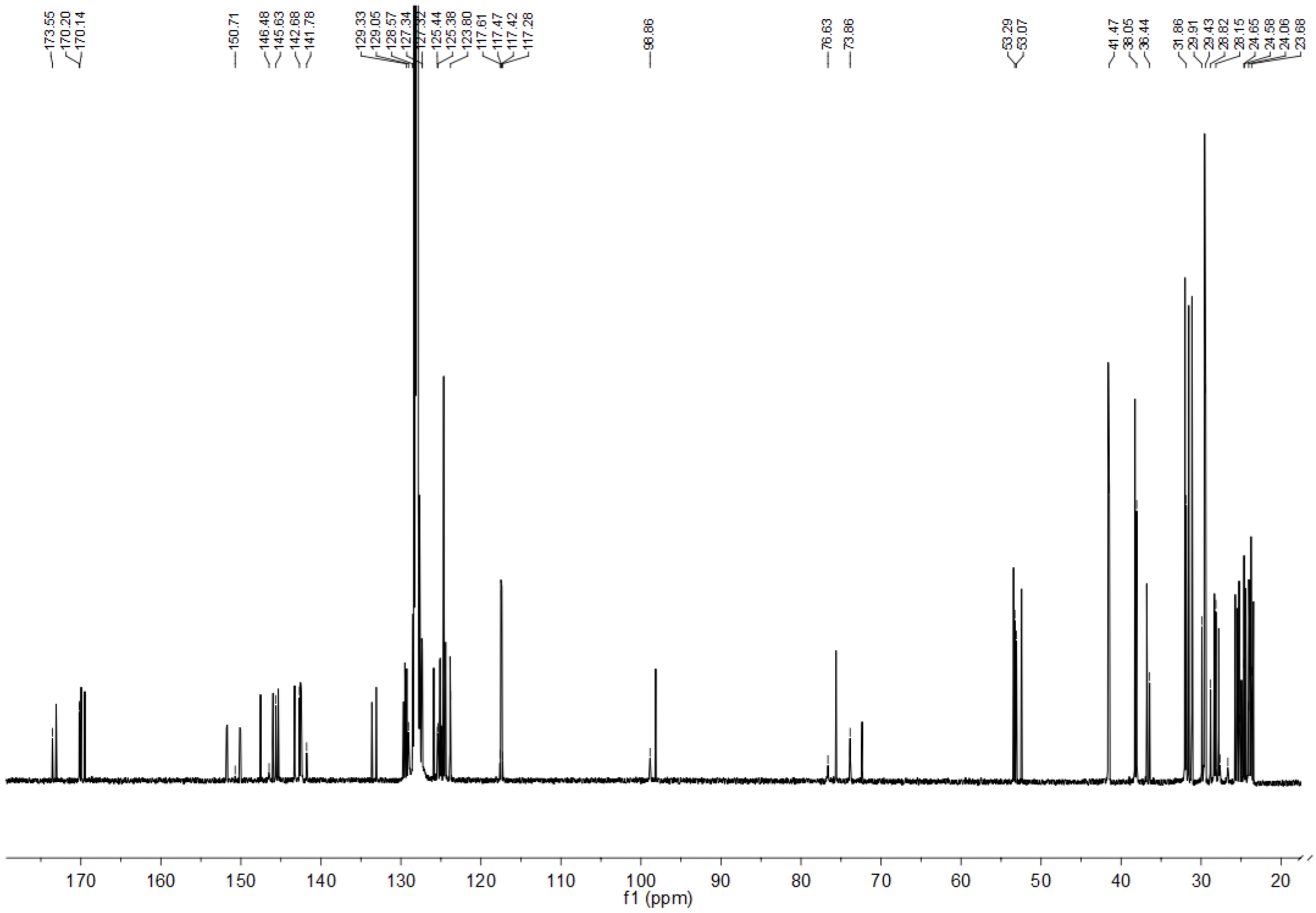

Figure S28. ^{13}C NMR spectrum of $L^2Si[OCHC_{10}H_{15}]OCH[Ga(Cl)L^1]C_6H_5$ (**7**) in C_6D_6 with diastereomere **7b** marked at room temperature.

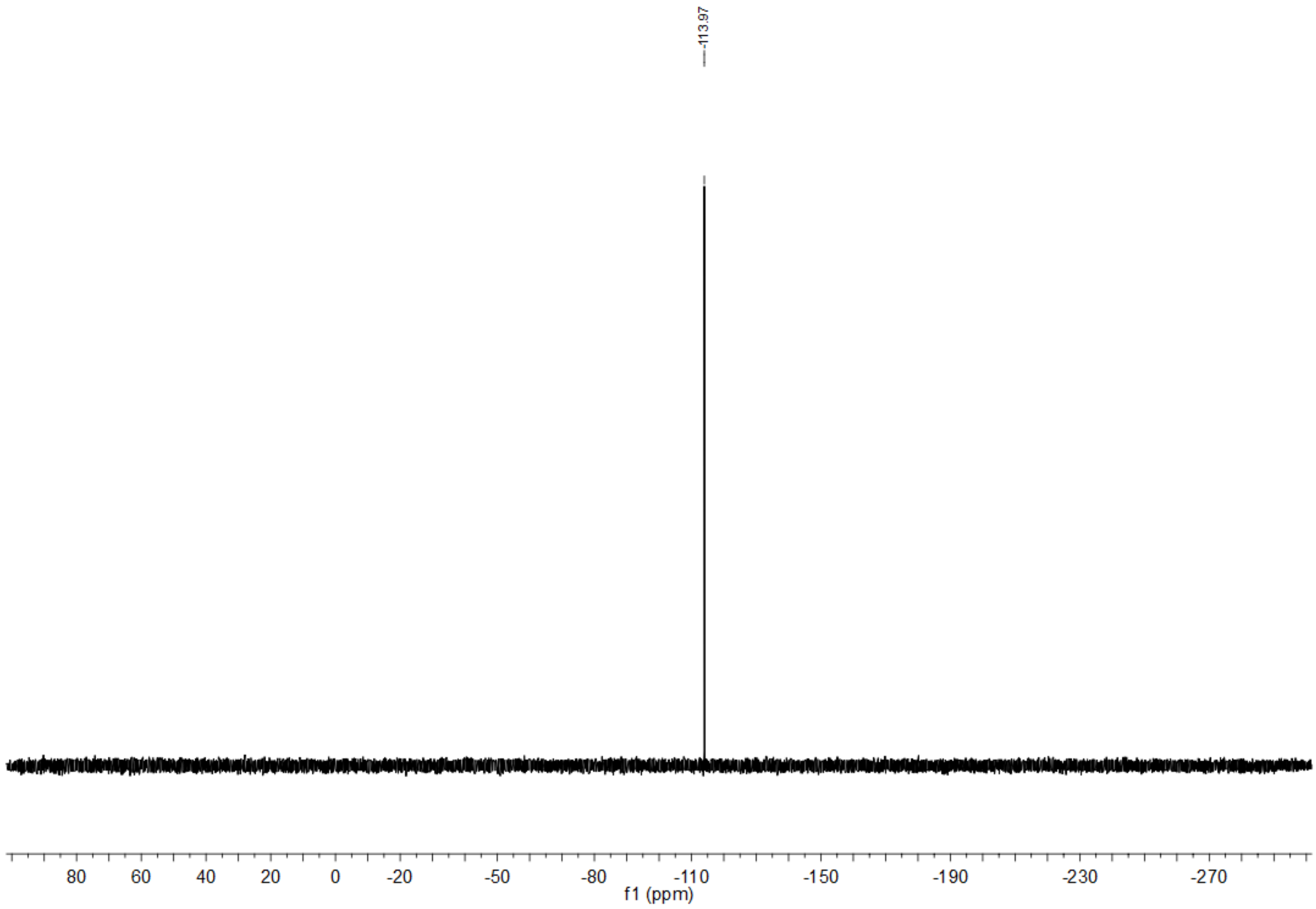

Figure S29. ^{29}Si NMR spectrum of $L^2Si[OCHC_{10}H_{15}]OCH[Ga(Cl)L^1]C_6H_5$ (**7**) in C_6D_6 at room temperature.

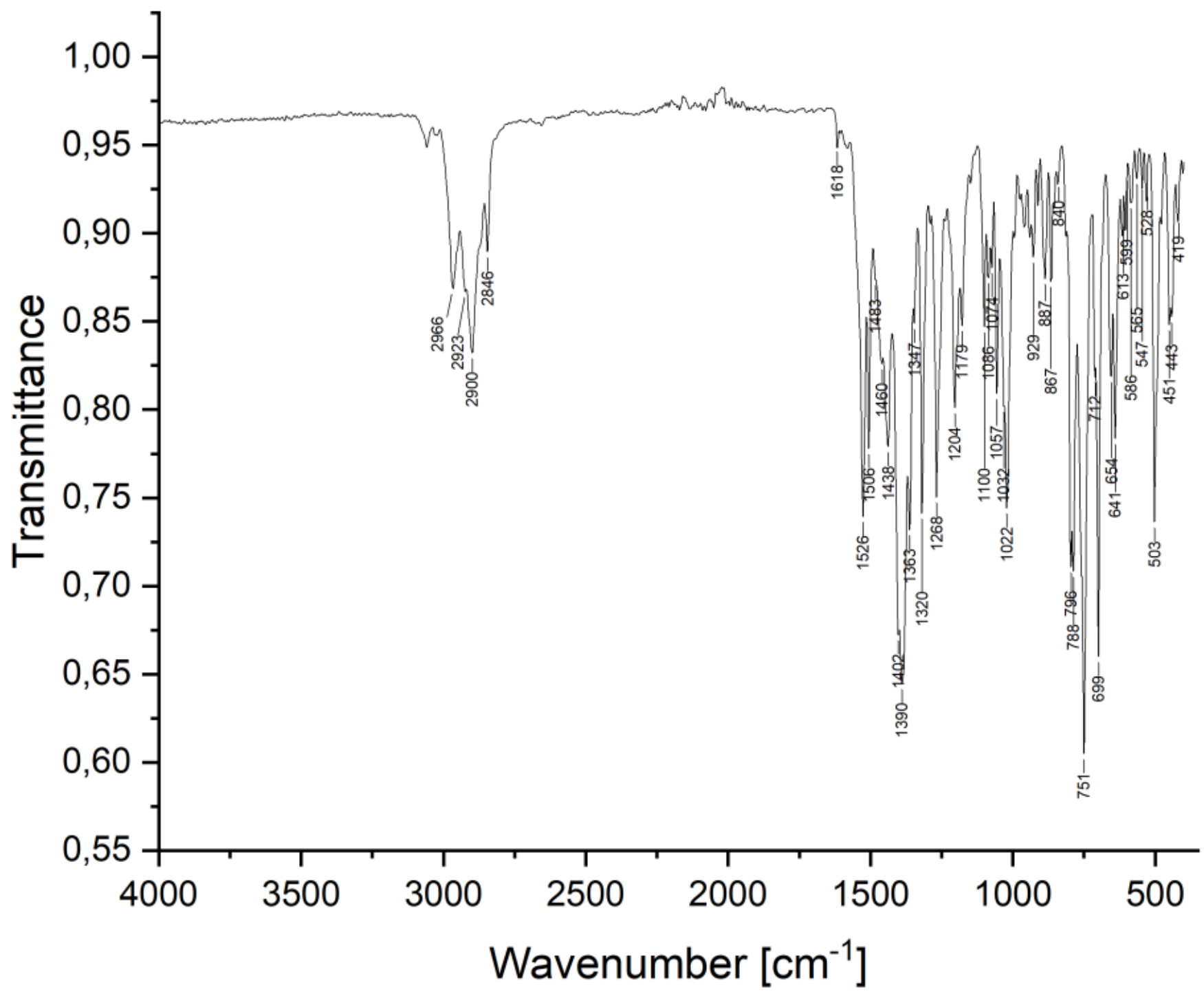

Figure S30. IR spectrum of $L^2Si[OCHC_{10}H_{15}]OCH[Ga(Cl)L^1]C_6H_5$ (**7**).

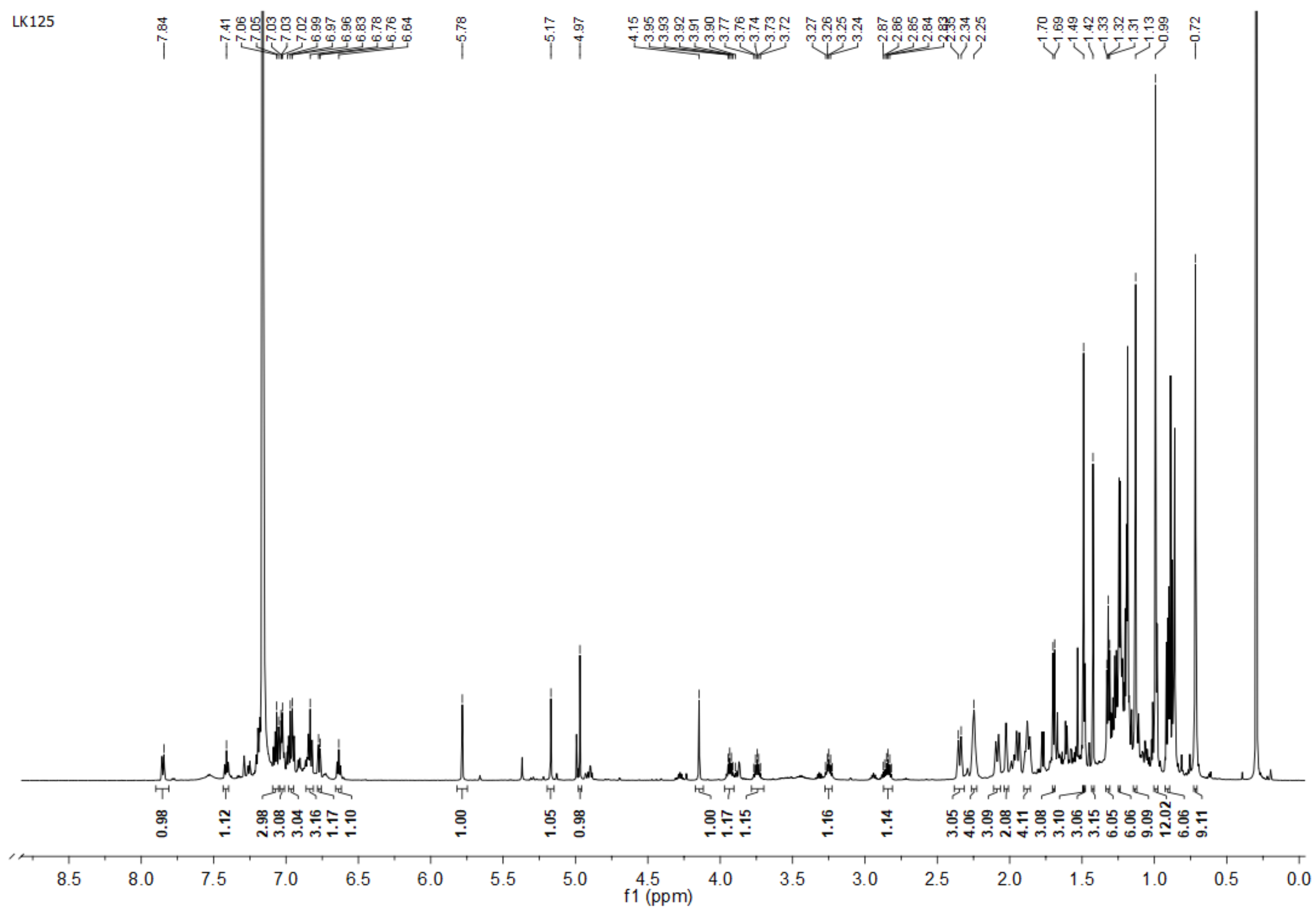

Figure S31. ^{1}H NMR spectrum of $L^2[H]Si[(C_6H_{12}O_2B)OCHC_{10}H_{15}]OCH[Ga(Cl)L^1]C_6H_5$ (**8**) in C_6D_6 (containing residues of the corresponding silane) at room temperature.

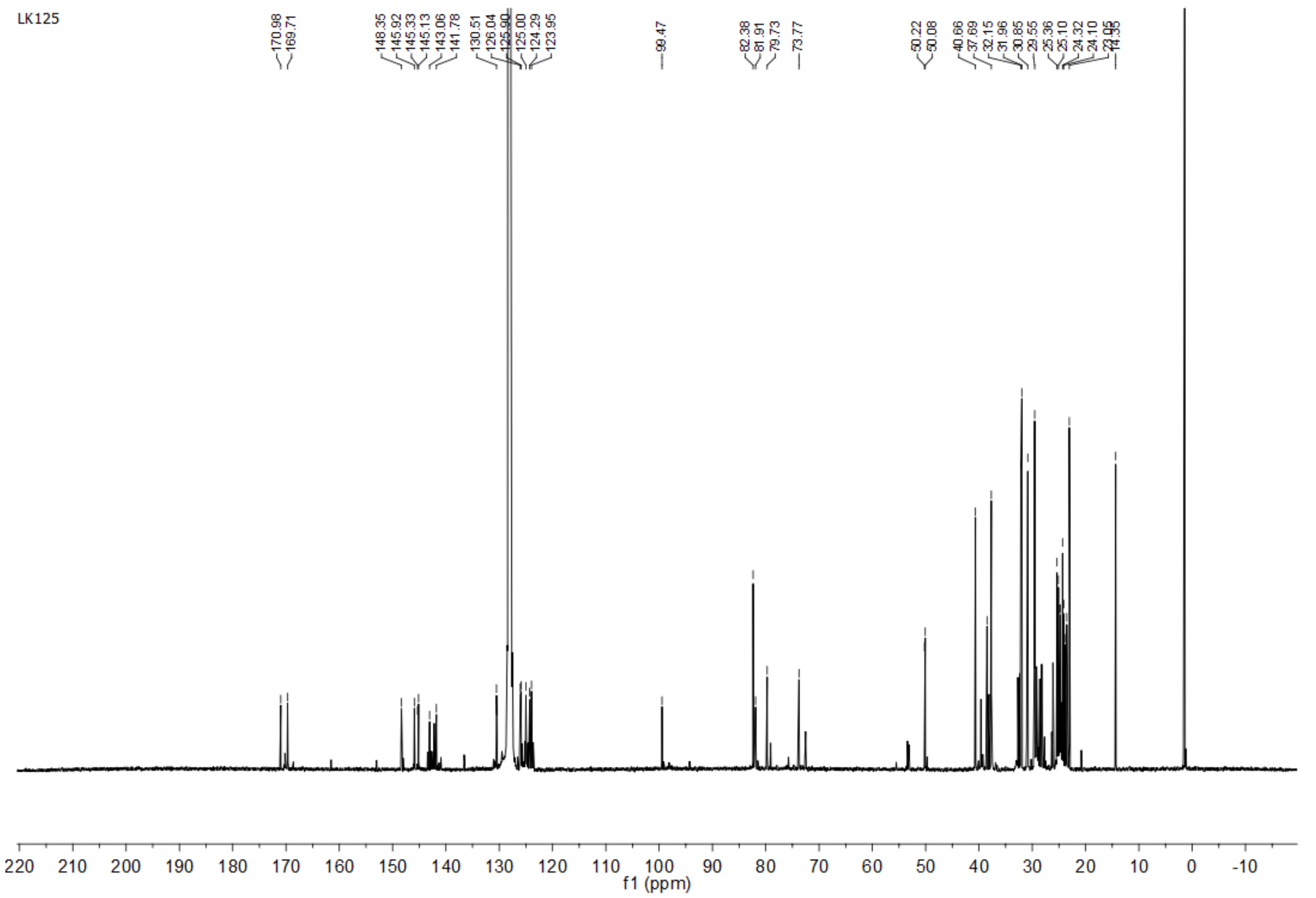

Figure S32. ^{13}C NMR spectrum of $L^2[H]Si[(C_6H_{12}O_2B)OCHC_{10}H_{15}]OCH[Ga(Cl)L^1]C_6H_5$ (**8**) in C_6D_6 (containing residues of the corresponding silane) at room temperature.

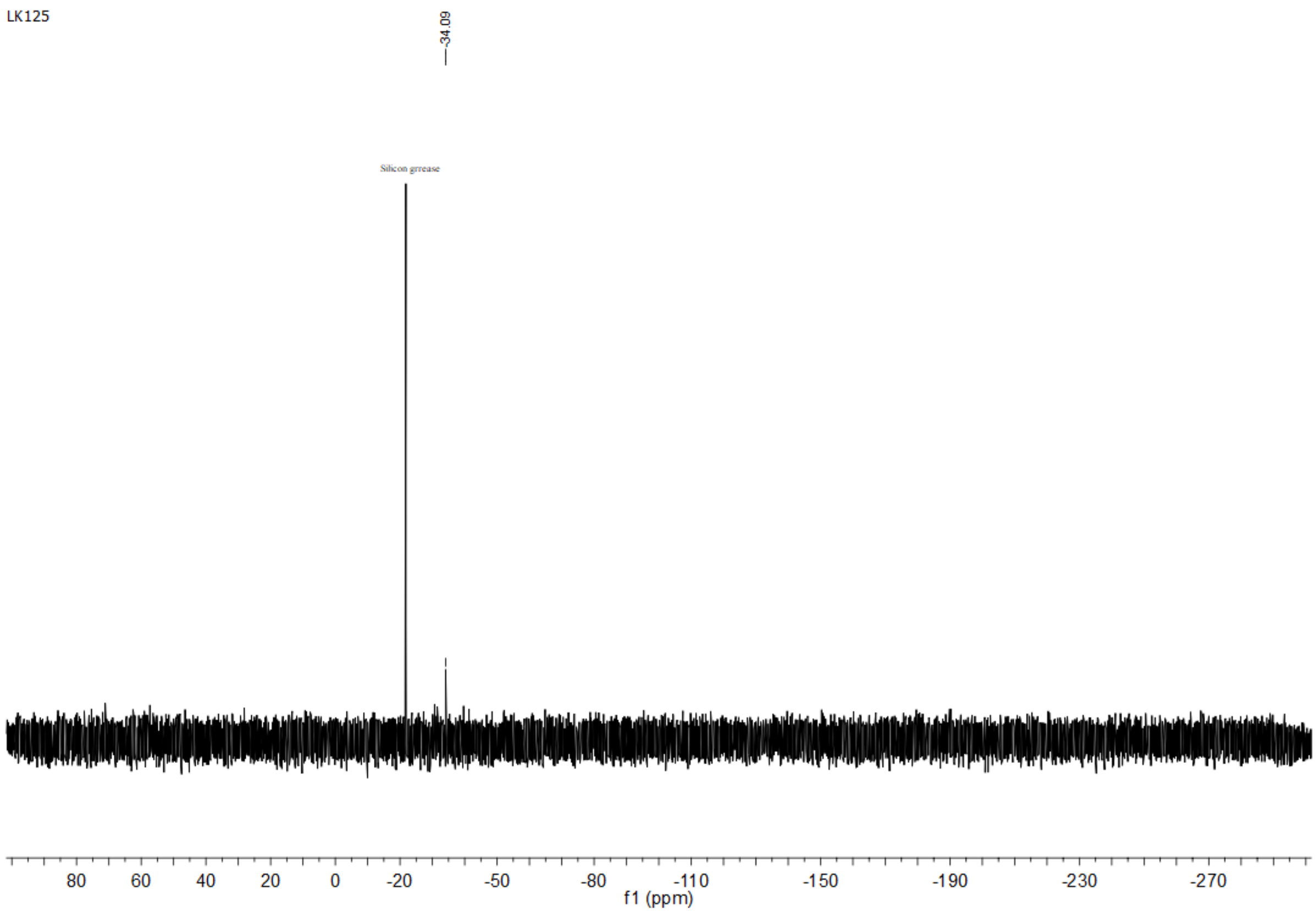

Figure S33. ^{29}Si NMR spectrum of $L^2[H]Si[(C_6H_{12}O_2B)OCHC_{10}H_{15}]OCH[Ga(Cl)L^1]C_6H_5$ (**8**) in C_6D_6 at room temperature.

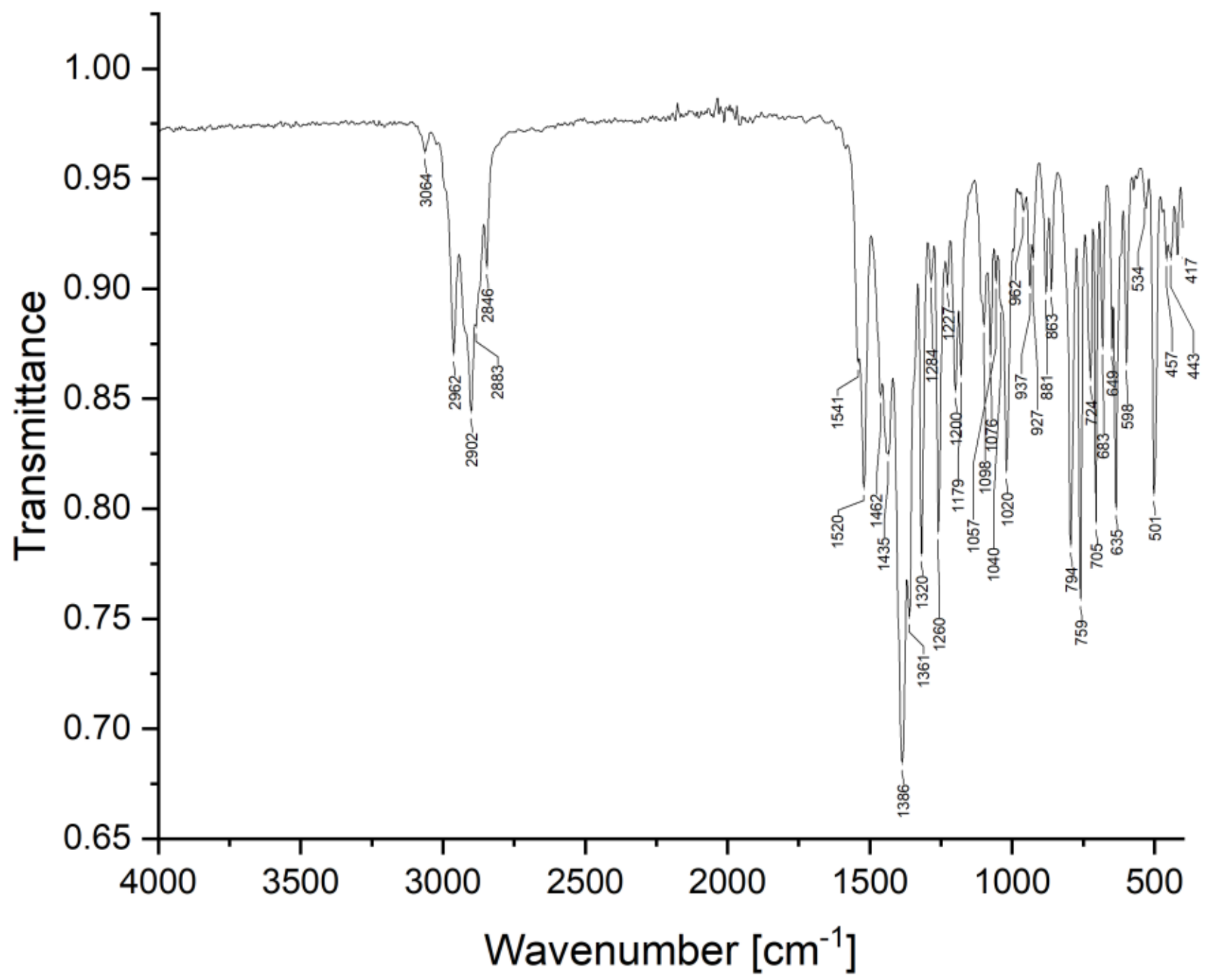

Figure S34. IR spectrum of $L^2[H]Si[(C_6H_{12}O_2B)OCHC_{10}H_{15}]OCH[Ga(Cl)L^1]C_6H_5$ (**8**).

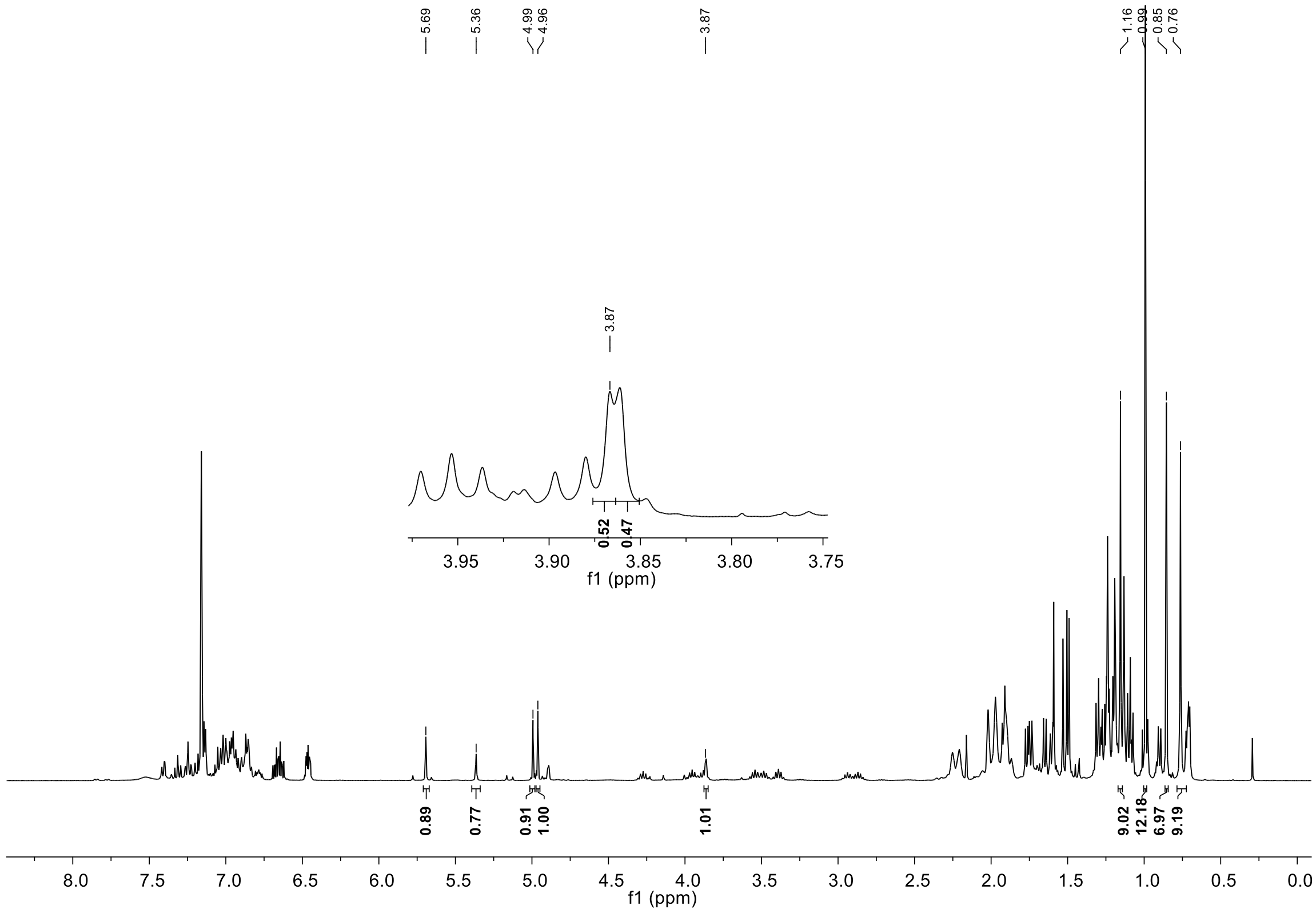

Figure S35. *In situ* ^{1}H NMR spectrum of the equimolar reaction of compound (**7**) and HBPin in C_6D_6 at room temperature after 10 min, showing slow conversion to the corresponding silane **8a** (3.87 & 3.86 ppm – two signals due to diastereomers).

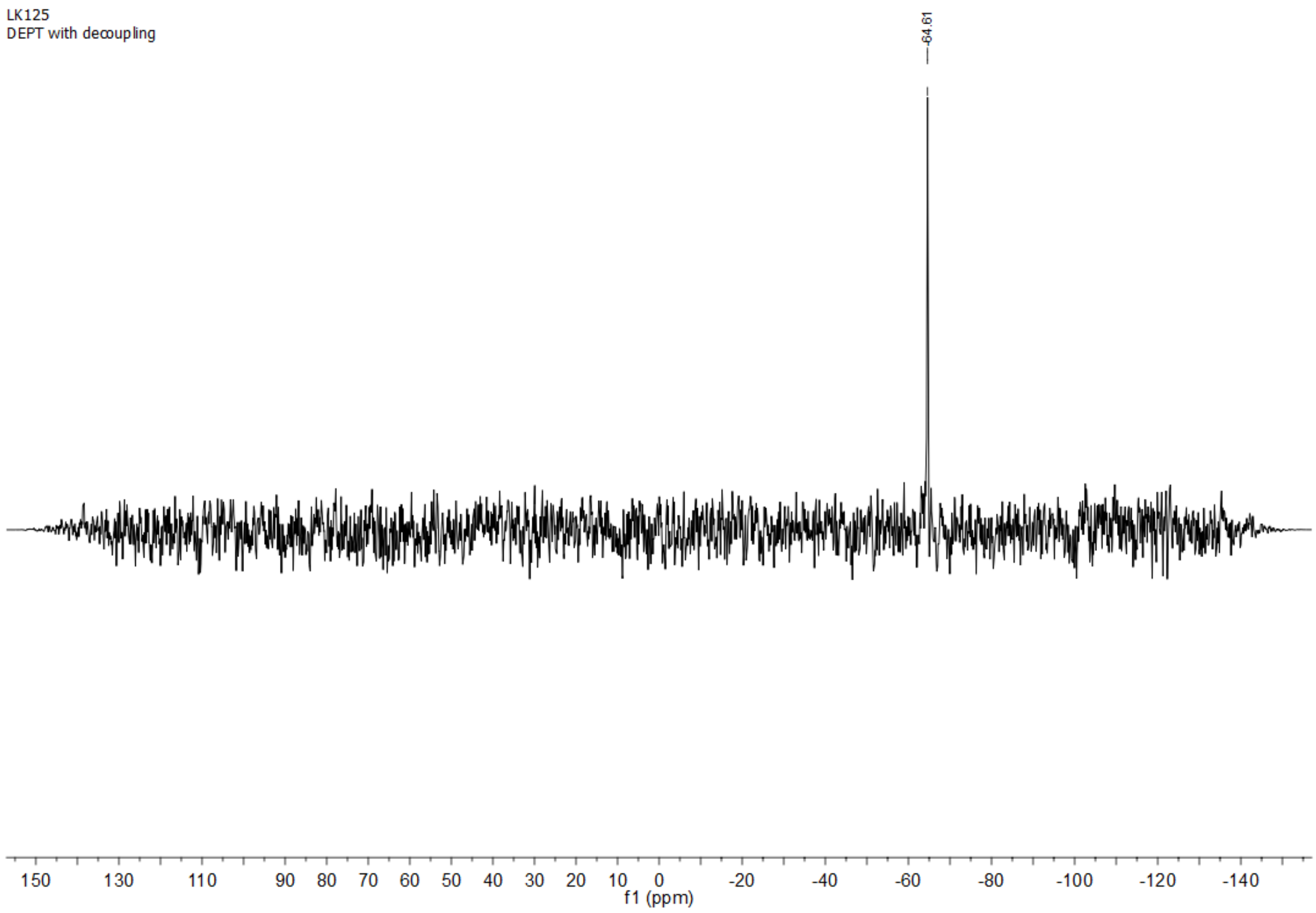

Figure S36. *In situ* ^{29}Si{H}DEPT90-NMR spectrum of the equimolar reaction of compound (**7**) and HBPin in C_6D_6 at room temperature, showing formation of the corresponding silane **8a**.

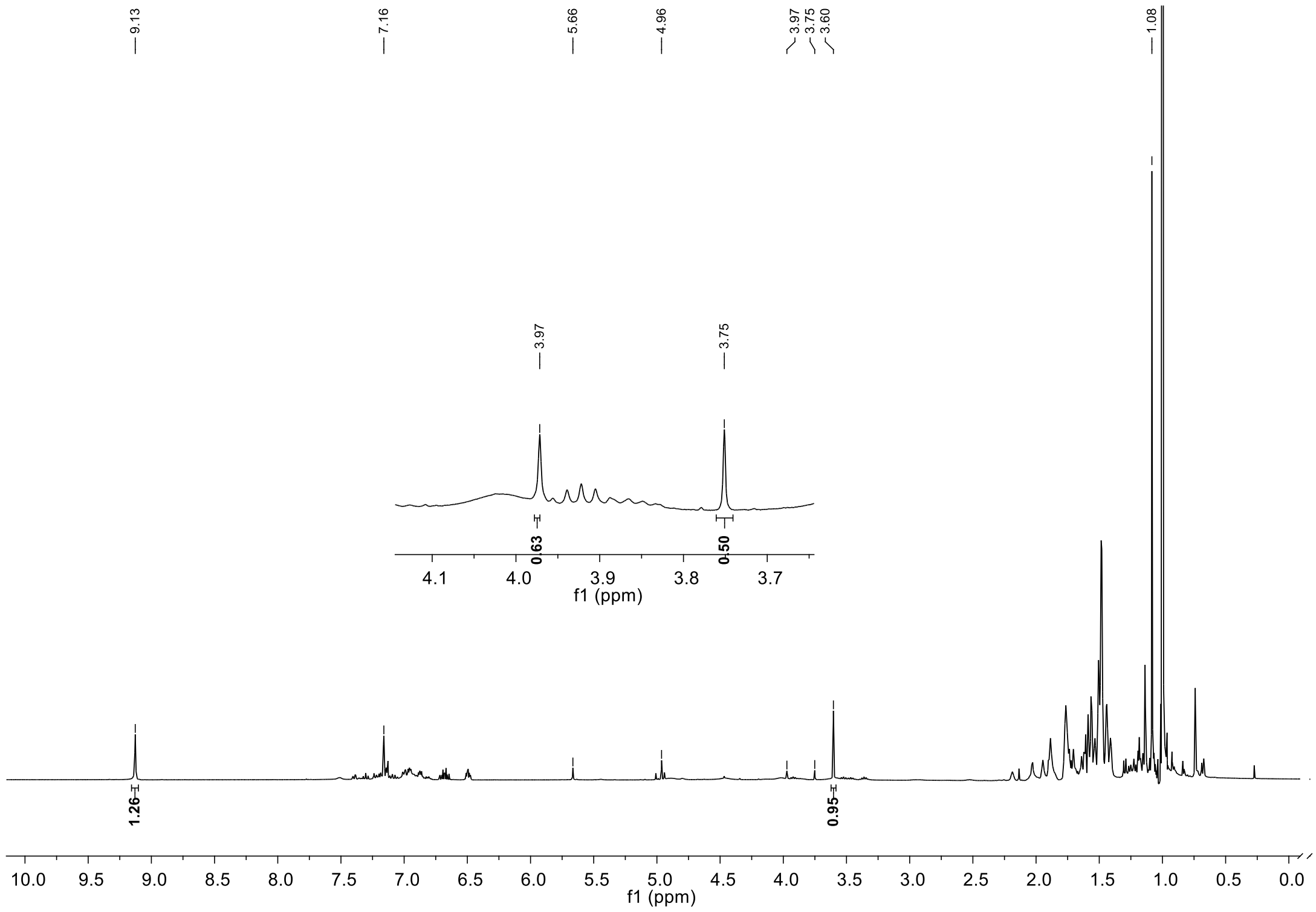

Figure S37. *In situ* ^{1}H NMR spectrum of the catalytic hydroboration of 1-adamantanecarboxaldehyde and HBPin with 20 mol% of **7** in C_6D_6 at room temperature after 15 min showing conversion to the corresponding boranester and formation of the aldehyde-coordinated silane **8a** (3.97 & 3.75 ppm – two signals due to diastereomers).

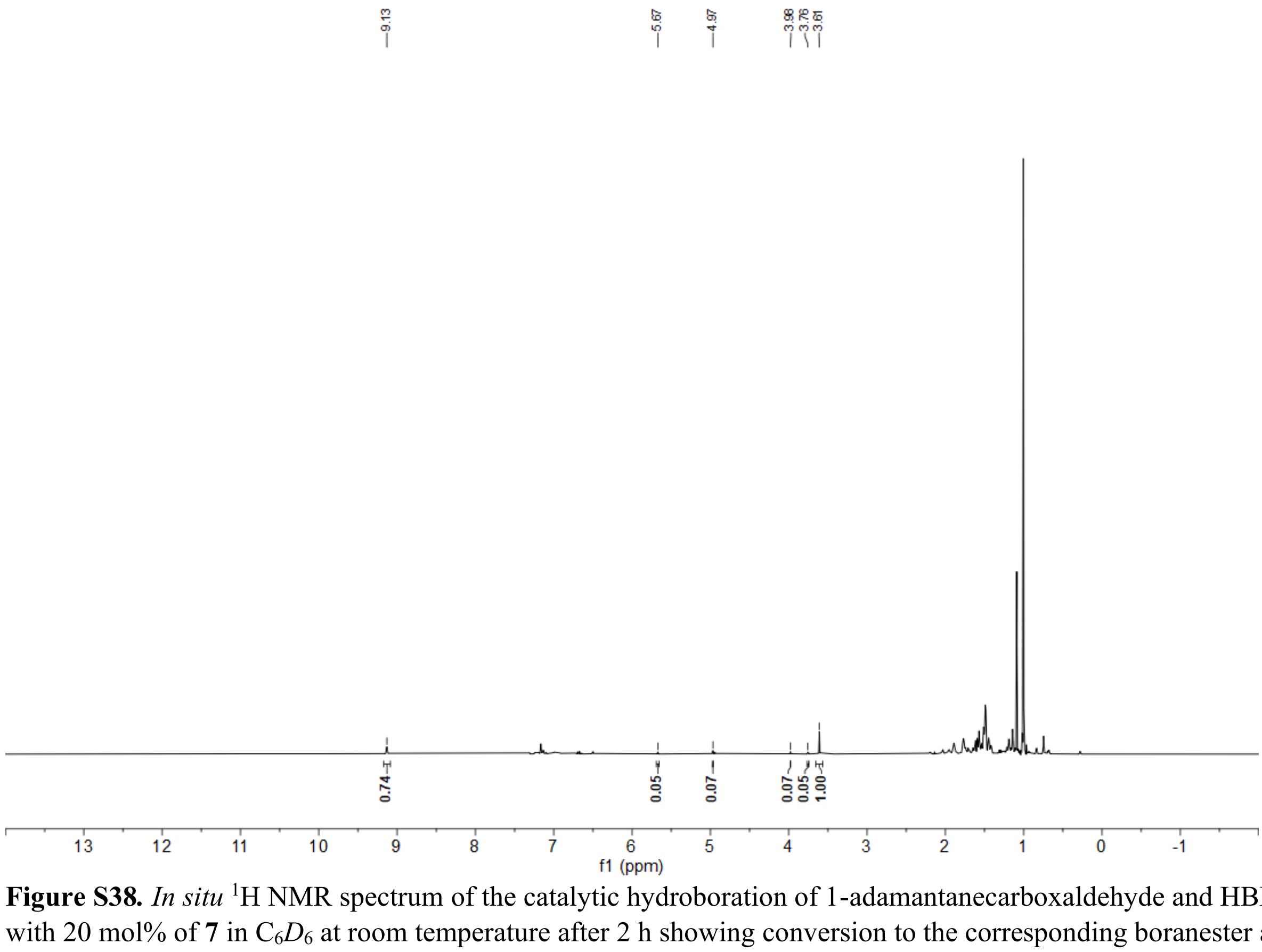

Figure S38. *In situ* ^{1}H NMR spectrum of the catalytic hydroboration of 1-adamantanecarboxaldehyde and HBPin with 20 mol% of **7** in C_6D_6 at room temperature after 2 h showing conversion to the corresponding boranester and formation of the aldehyde-coordinated silane **8a** (3.97 & 3.75 ppm – two signals due to diastereomers).

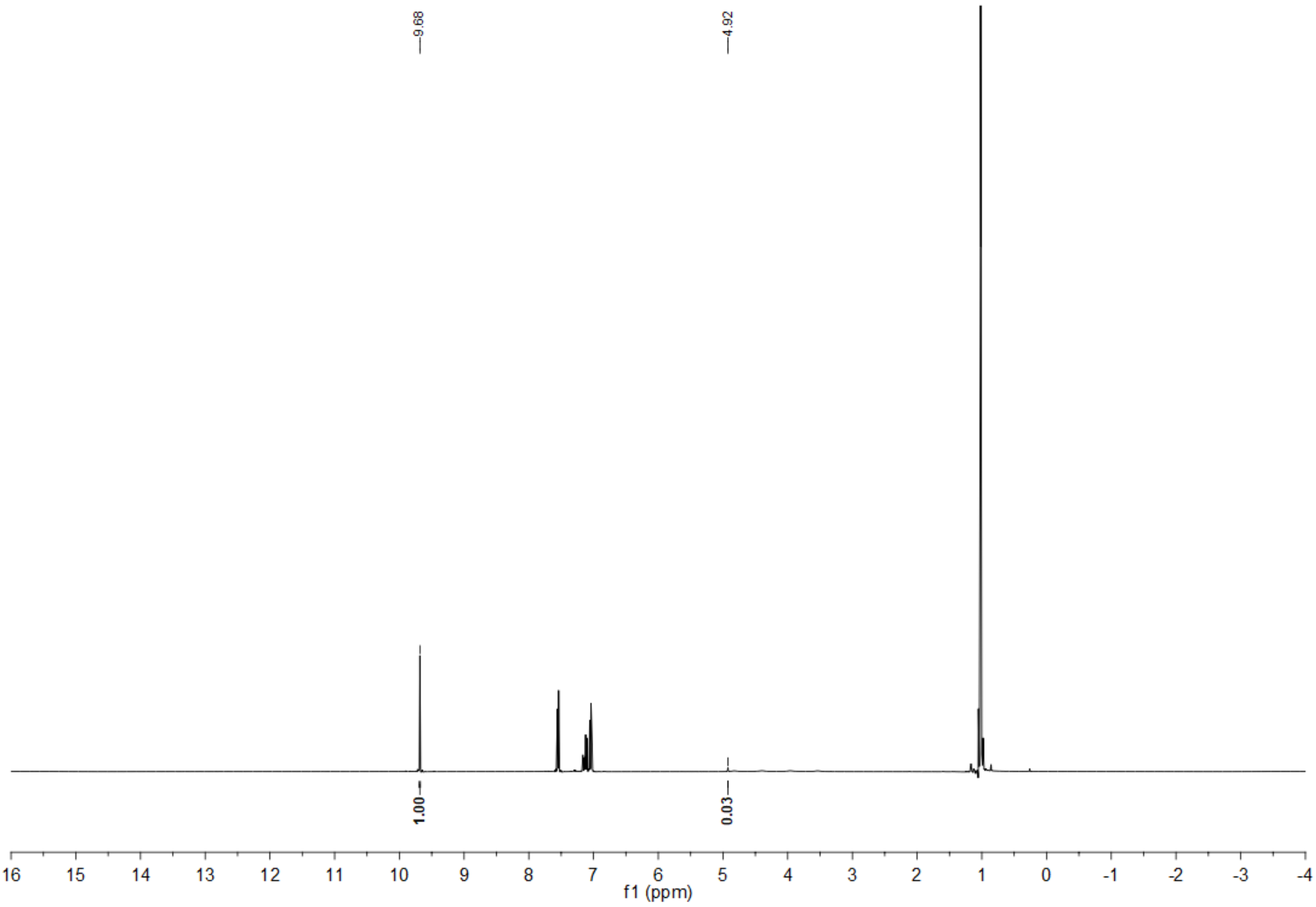

Figure S39. *In situ* ^{1}H NMR spectrum of the catalytic reaction (benzaldehyde and HBPin) with 1 mol% of **8** in C_6D_6 at room temperature showing no catalytic conversion after 20 h.

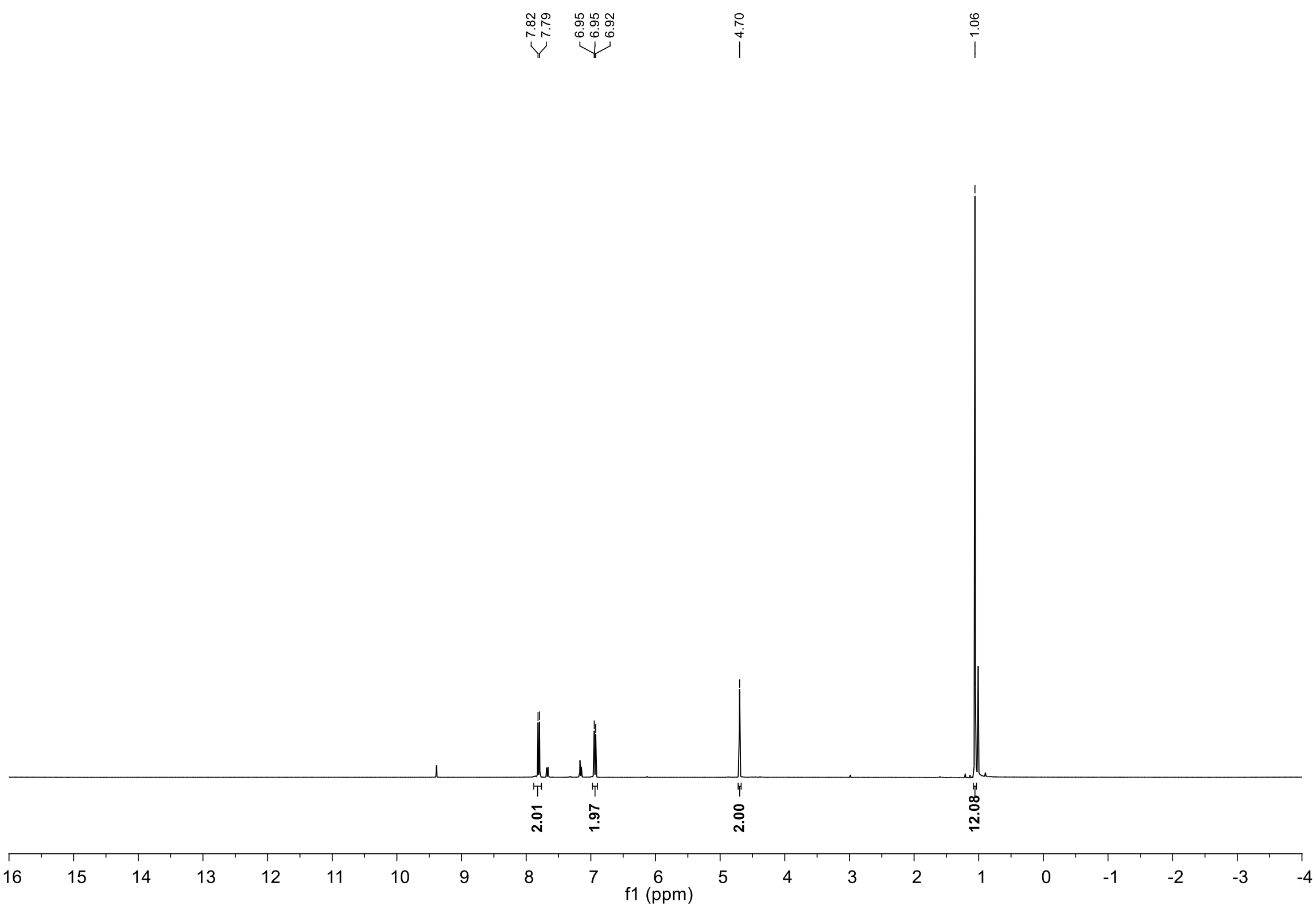

Figure S40. *In situ* ^{1}H NMR spectrum of the catalytic reaction (4-nitrobenzaldehyde and HBPin) with 0.01 mol% of **3** in C_6D_6 at room temperature.

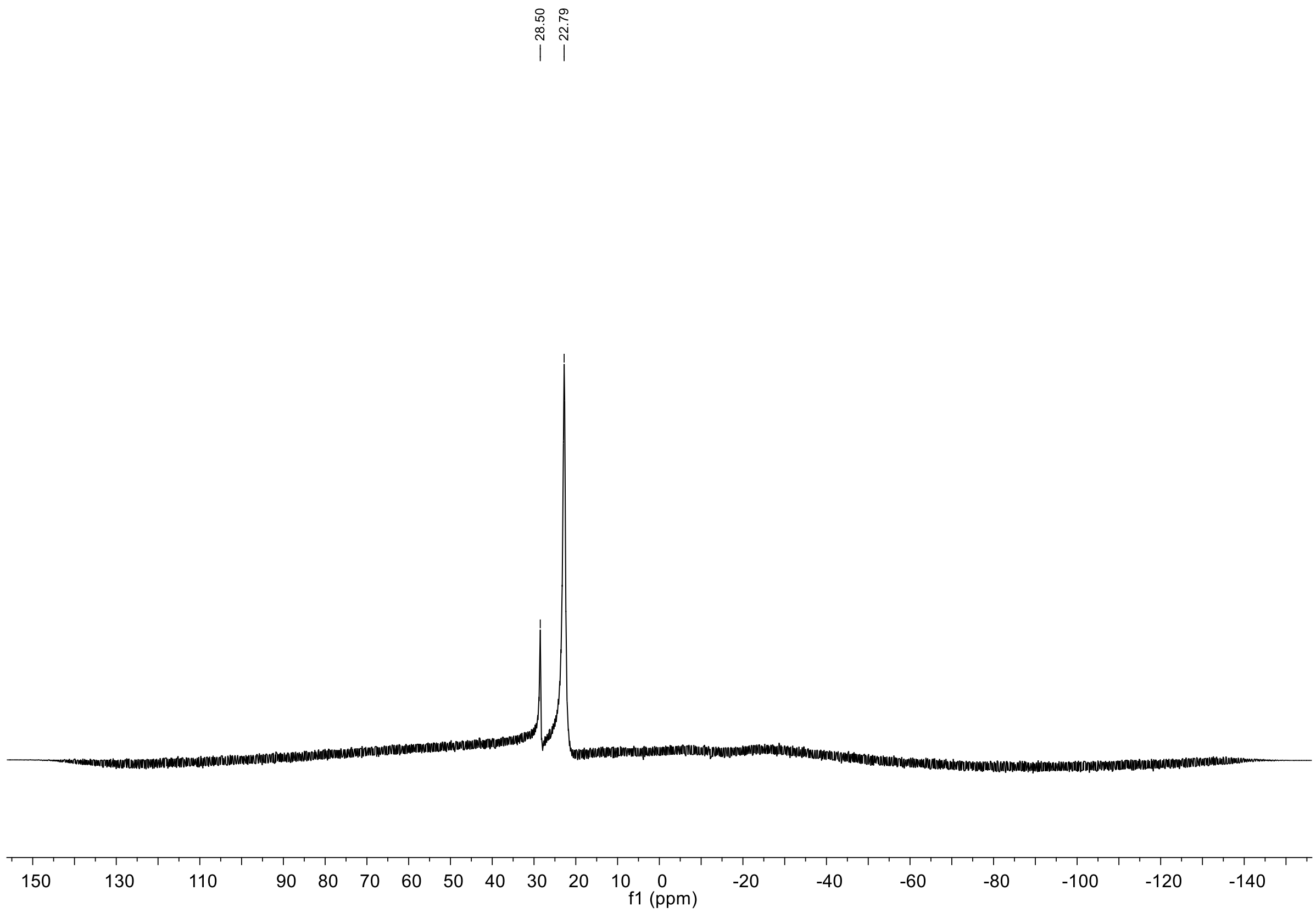

Figure S41. *In situ* ^{11}B NMR spectrum of the catalytic reaction (4-nitrobenzaldehyde and HBPin) with 0.01 mol% of **3** in C_6D_6 at room temperature.

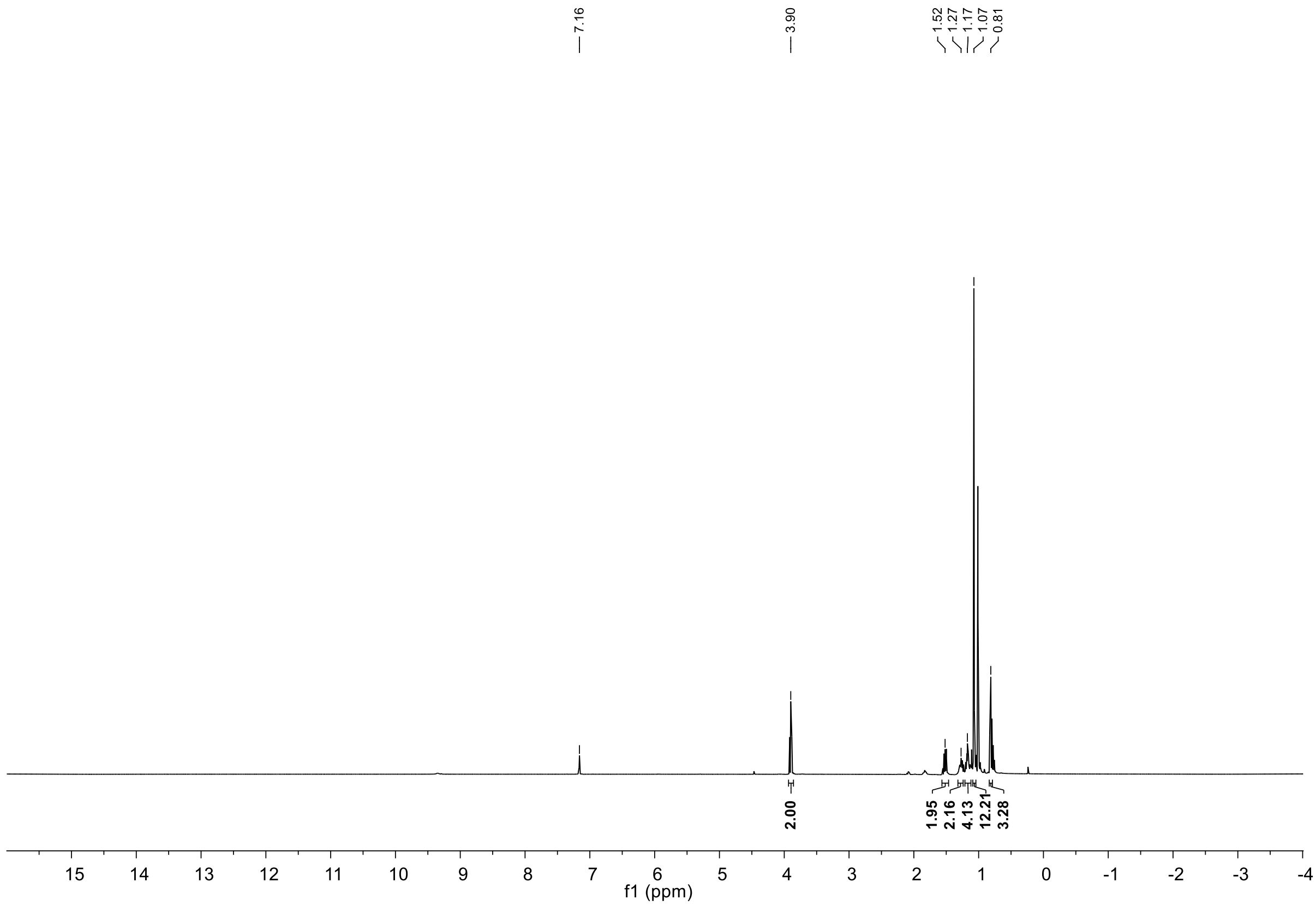

Figure S42. *In situ* ^{1}H NMR spectrum of the catalytic reaction (*n*-hexanal and HBPin) with 0.1 mol% of **3** in C_6D_6 at room temperature.

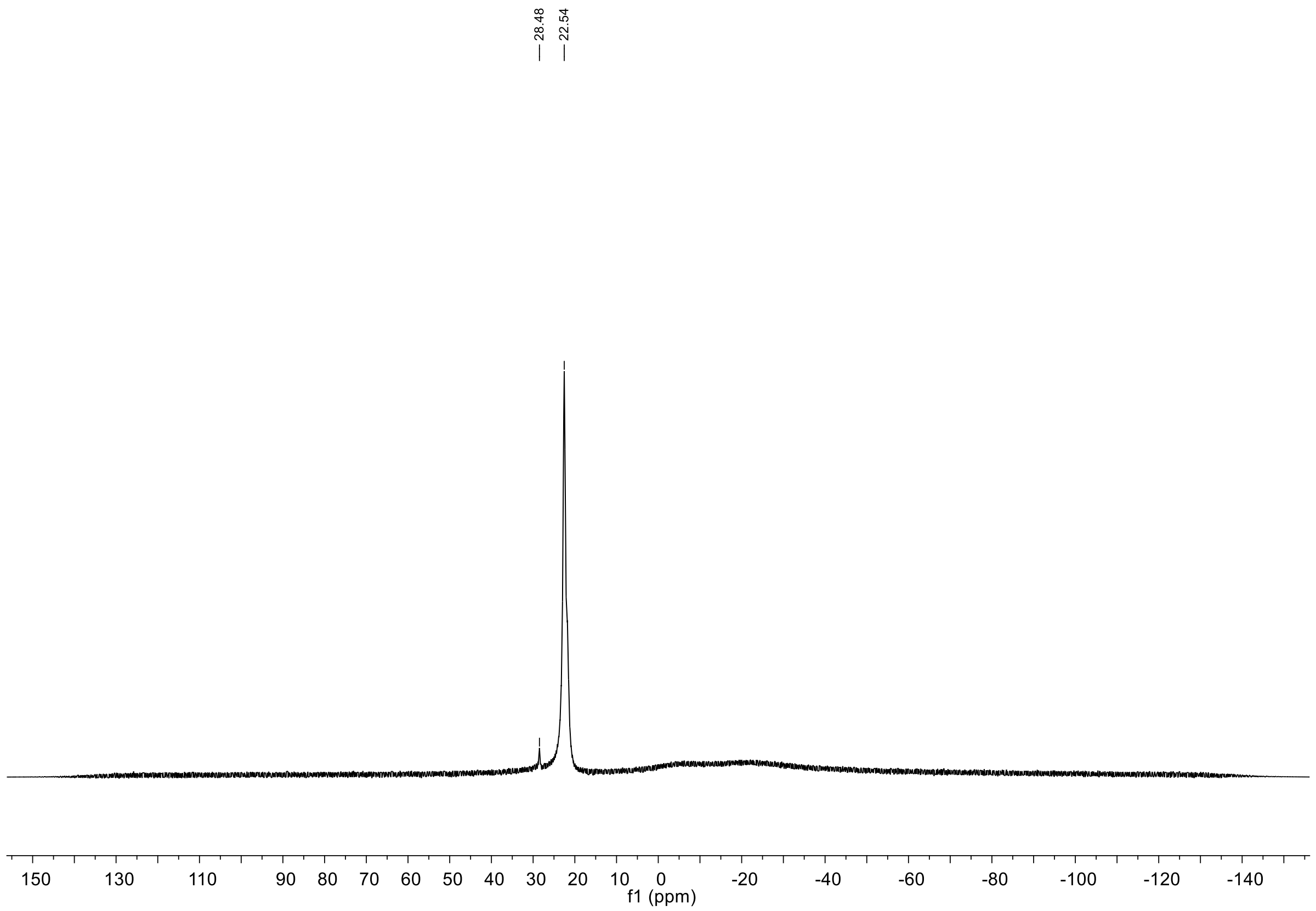

Figure S43. *In situ* ^{11}B NMR spectrum of the catalytic reaction (*n*-hexanal and HBPin) with 0.1 mol% of **3** in C_6D_6 at room temperature.

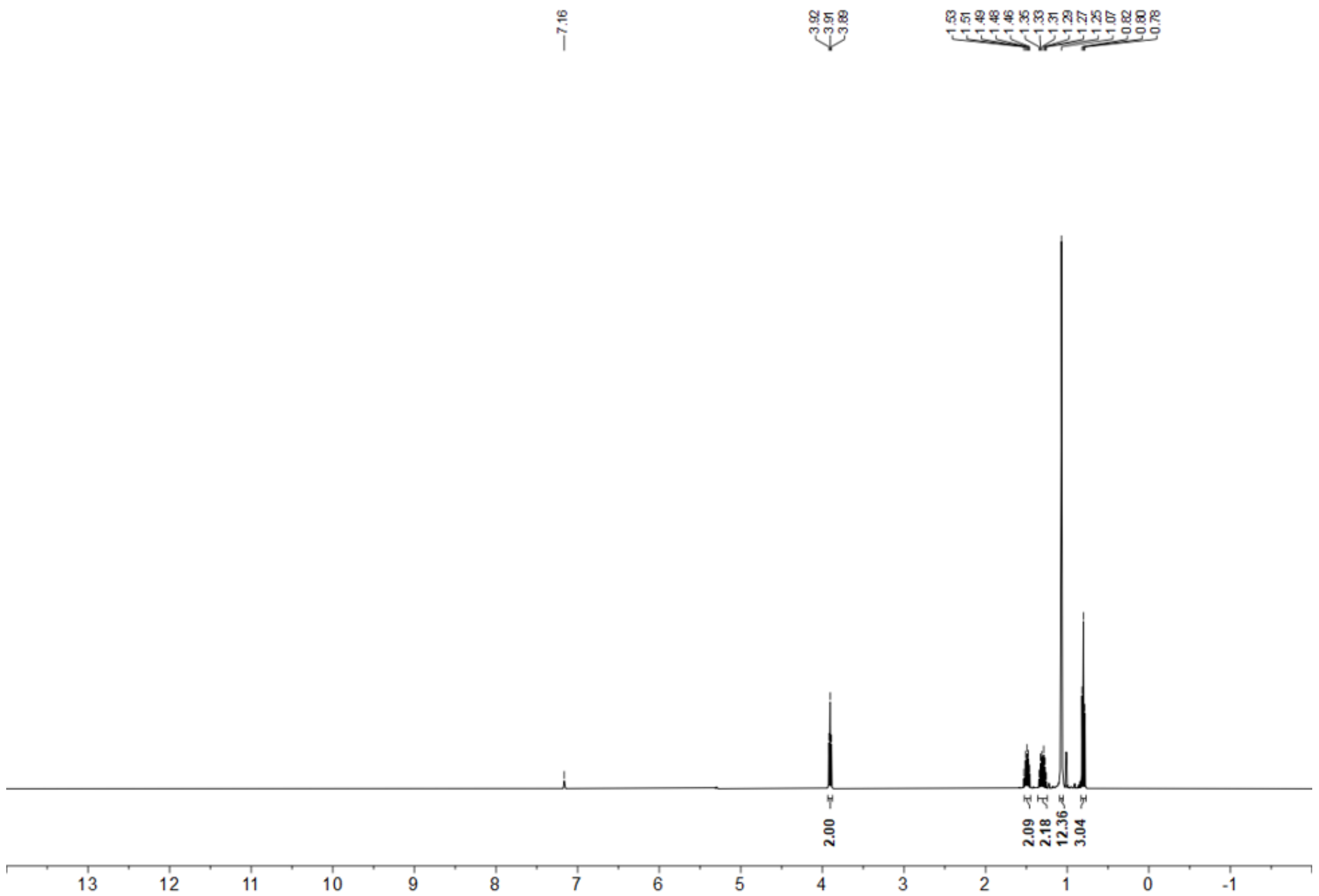

Figure S44. *In situ* ^{1}H NMR spectrum of the catalytic reaction (*n*-butanal and HBPin) with 0.1 mol% of **3** in C_6D_6 at room temperature.

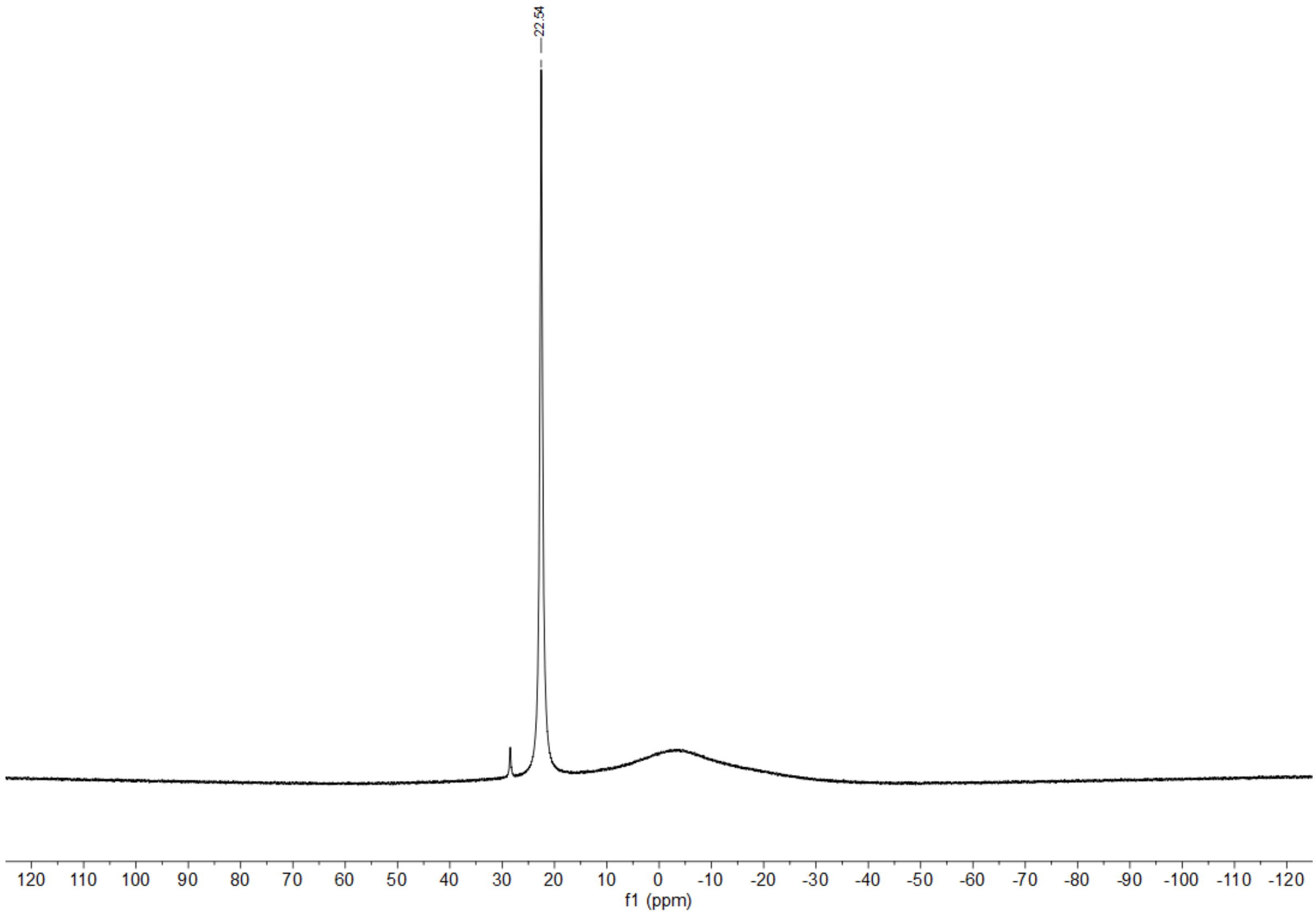

Figure S45. *In situ* ^{11}B NMR spectrum of the catalytic reaction (*n*-butanal and HBPin) with 0.1 mol% of **3** in C_6D_6 at room temperature.

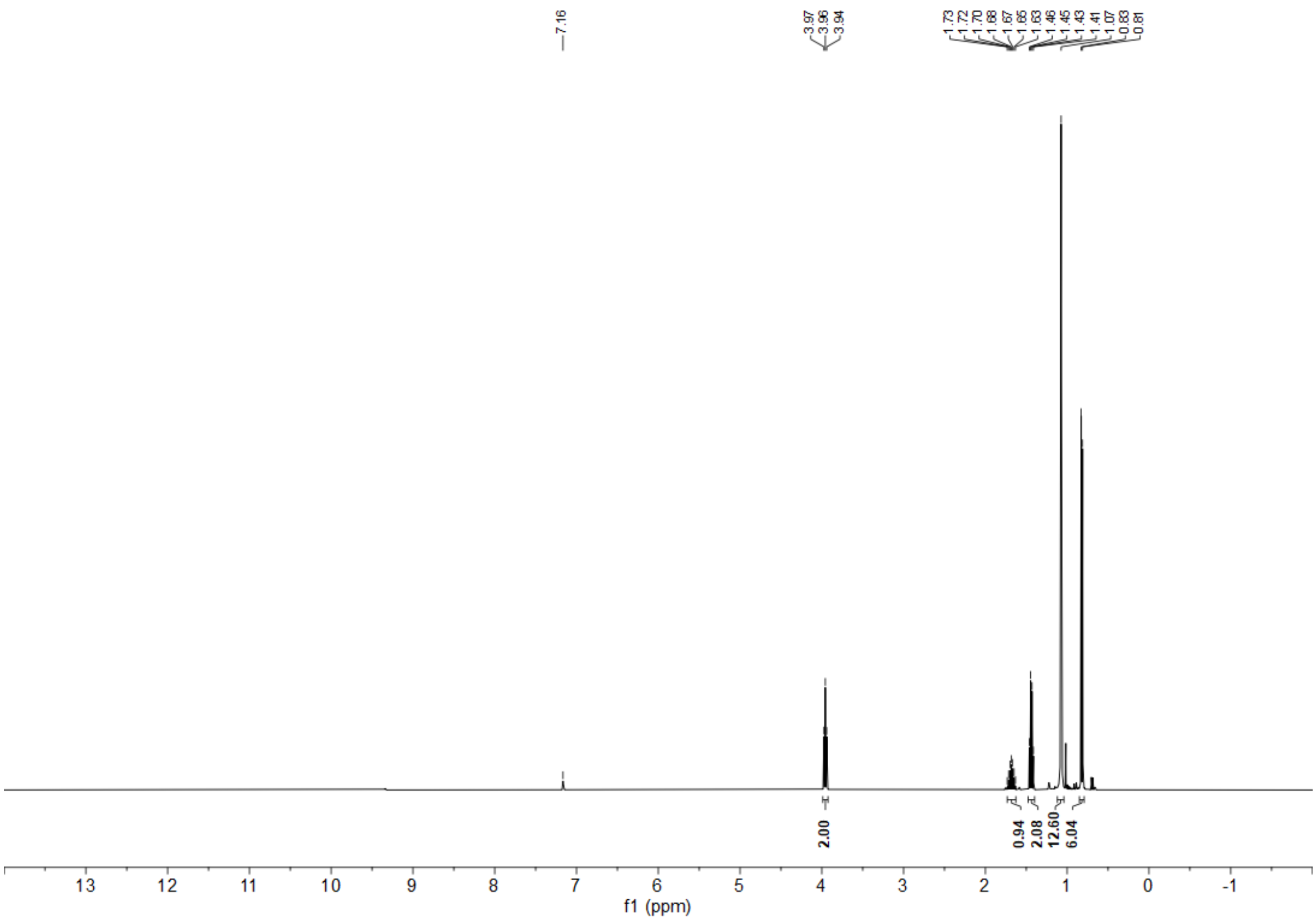

Figure S46. *In situ* ^{1}H NMR spectrum of the catalytic reaction (*iso*-valeraldehyde and HBPin) with 0.1 mol% of **3** in C_6D_6 at room temperature.

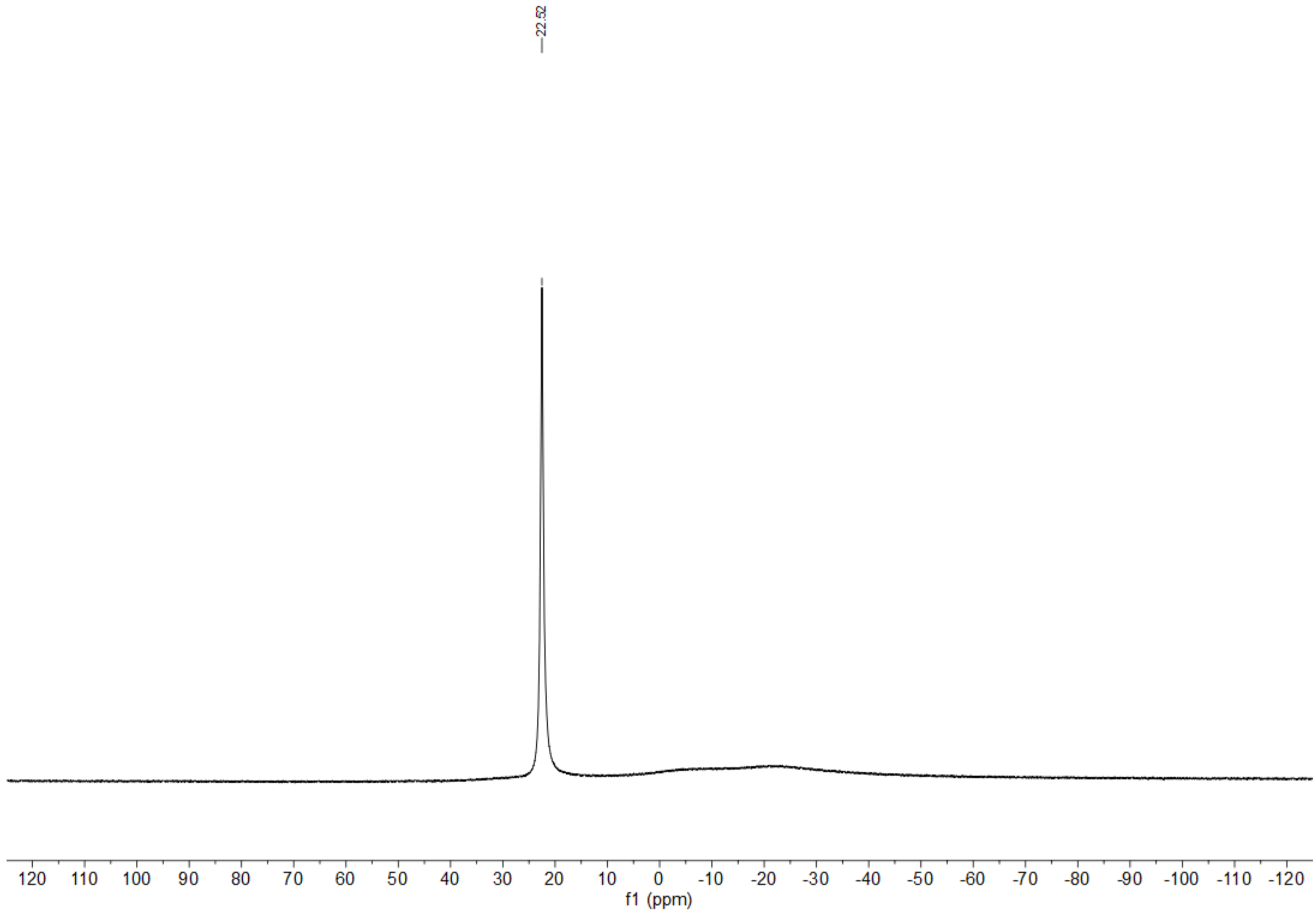

Figure S47. *In situ* ^{11}B NMR spectrum of the catalytic reaction (*iso*-valeraldehyde and HBPin) with 0.1 mol% of **3** in C_6D_6 at room temperature.

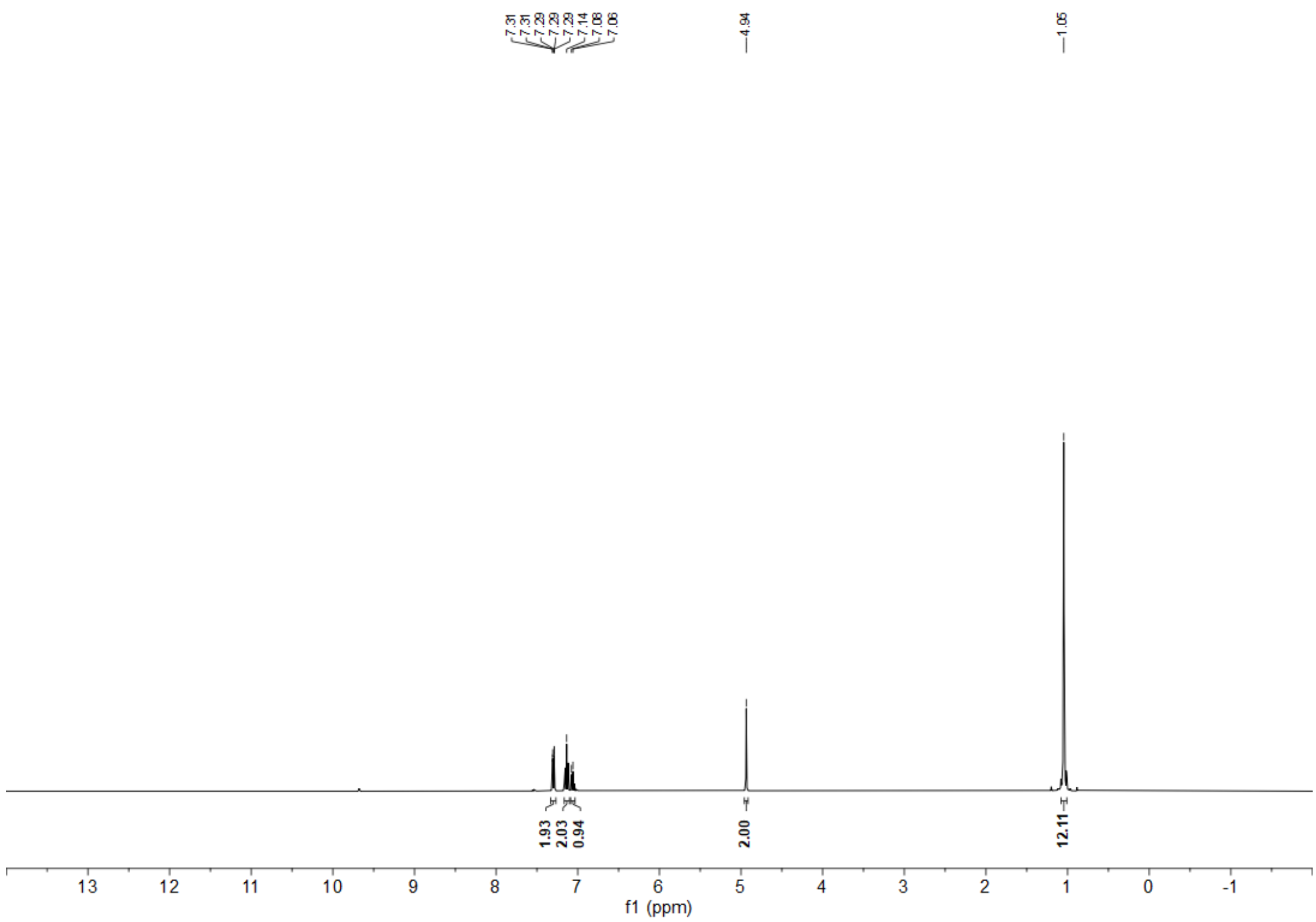

Figure S48. *In situ* ^{1}H NMR spectrum of the catalytic reaction (benzaldehyde and HBPin) with 0.1 mol% of **3** in C_6D_6 at room temperature.

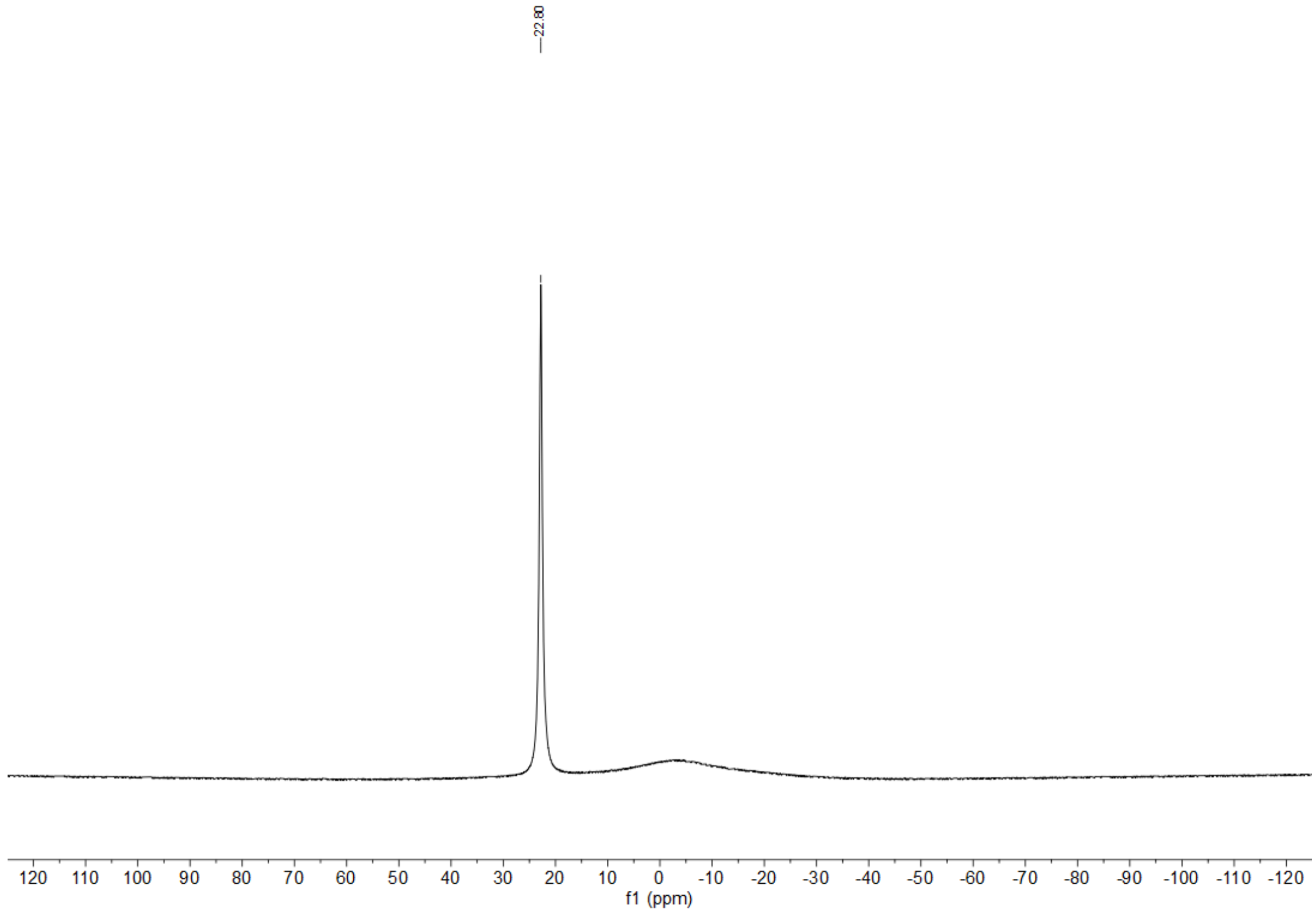

Figure S49. *In situ* ^{11}B NMR spectrum of the catalytic reaction (benzaldehyde and HBPin) with 0.1 mol% of **3** in C_6D_6 at room temperature.

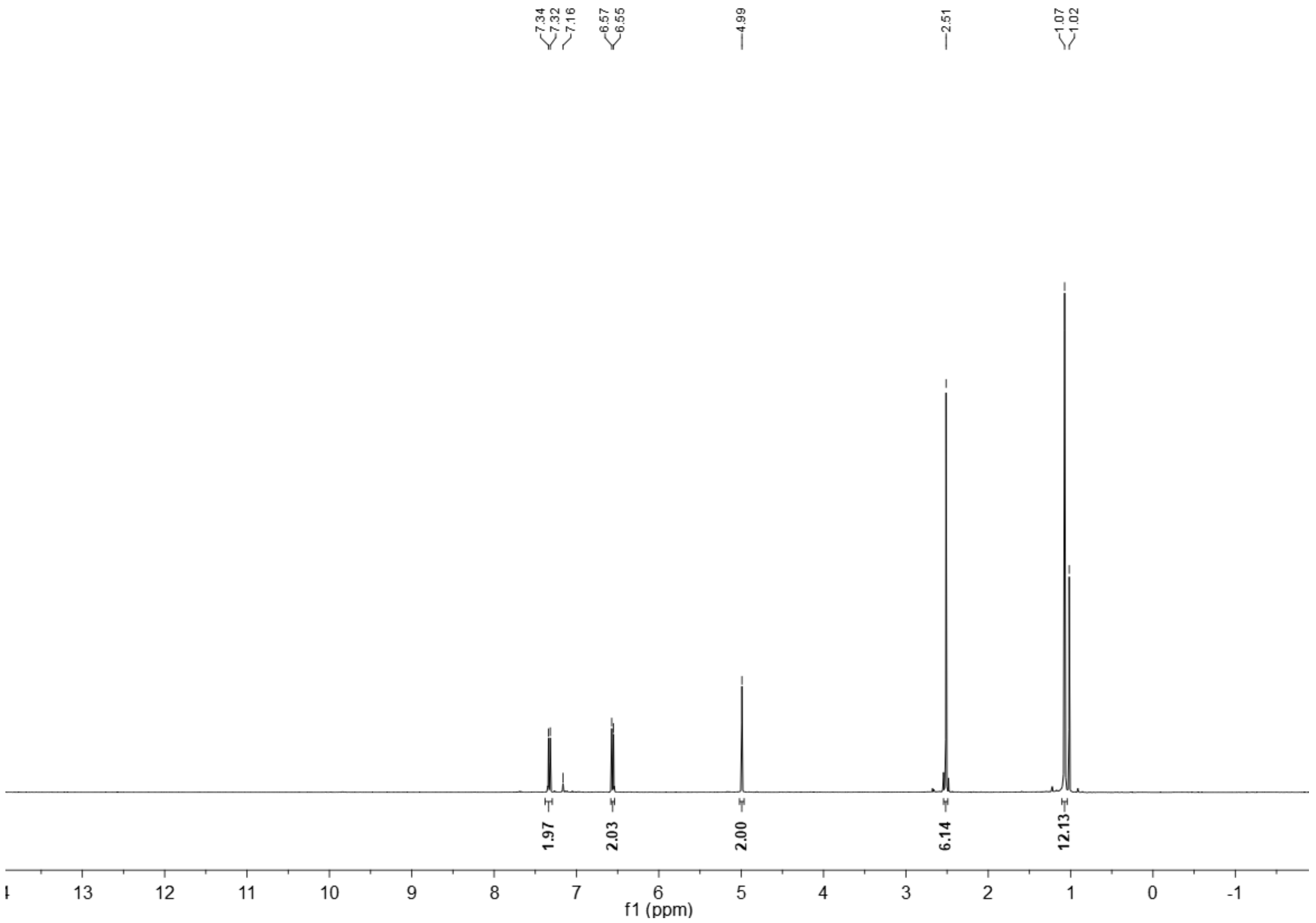

Figure S50. *In situ* ^{1}H NMR spectrum of the catalytic reaction (4-dimethylaminobenzaldehyde and HBPin) with 0.1 mol% of **3** in C_6D_6 at room temperature.

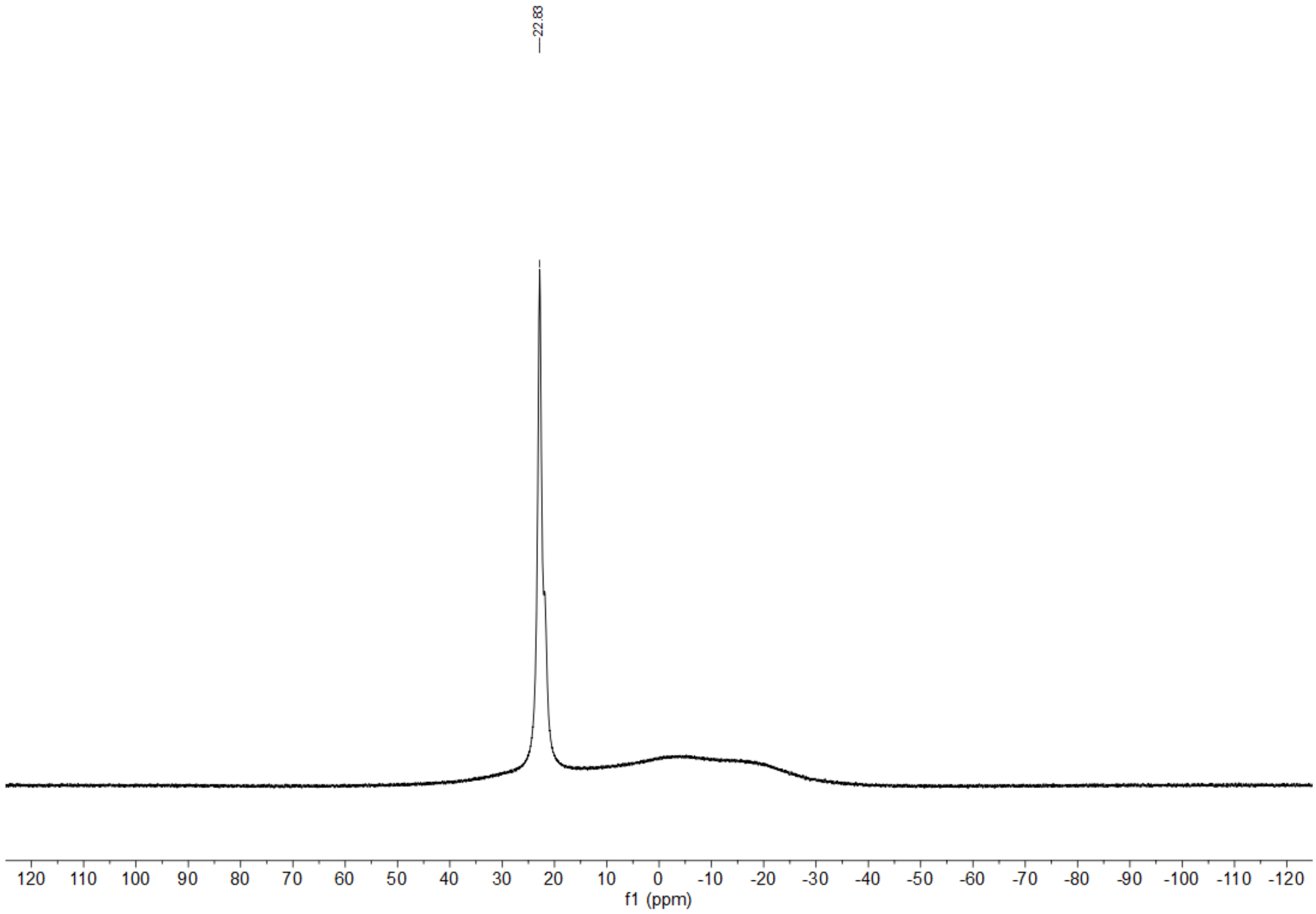

Figure S51. *In situ* ^{11}B NMR spectrum of the catalytic reaction (4-dimethylaminobenzaldehyde and HBPin) with 0.1 mol% of **3** in C_6D_6 at room temperature

II. Catalytic Studies

General procedure for the catalytic hydroboration of aldehydes and ketones. HBPin (56.4 mg, 0.44 mmol, 1.1 equiv.) and the substrate (carbonyl compound 0.40 mmol) were placed in a *J*-Young NMR tube, dissolved in 0.3 mL of C_6D_6 and 0.2 mL of a corresponding solution of the catalysts (**C**, **3**, **4**, **5**, **6**) was added. The solution was vortexed to form a homogeneous solution. The progress of the reaction was monitored by ^{1}H NMR spectroscopy every minute and conversion was determined by the relative integration area of product (OC*H*$_2$) and starting material signals (OC*H*) in the ^{1}H NMR spectrum. The values reported in literature confirm the chemical shifts of the products.[3-6]

Since BH_3 can catalyse the hydroboration of carbonyl compounds, HBPin was distilled prior to use. To further exclude the presence of BH_3 in our reaction we have performed a catalytic hydroboration reaction in the presence of two equivalents of TMEDA, which yielded the same activity and gave no TMEDA adducts after workup. This experiment was already addressed in our previous study.[2]

Table S1. Catalytic table for hydroboration of aldehydes using HBPin and different catalysts at room temperature**.**

Entry	Catalyst	Catalytic load (mol%)	Substrate	t(h)	Conversion (%)	TOF (h^{-1})
**1**	**C**	0.1	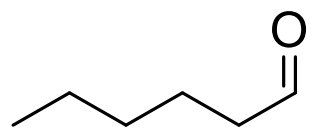	1	77	770
**2**	**C**	0.1	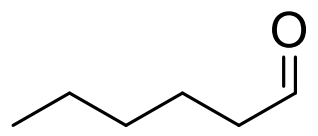	5.67	>99	176
**3**	**3**	0.1	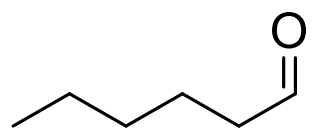	1	92	920
**4**	**3**	0.1	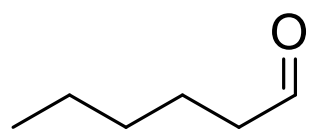	1.33	>99	752
**5**	**4**	0.1	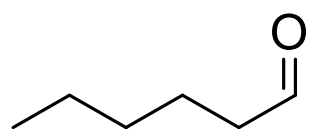	1	67	670
**6**	**4**	0.1	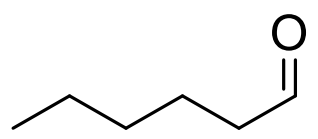	6.33	>99	158
**7**	**5**	0.1	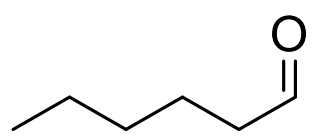	1	78	780
**8**	**5**	0.1	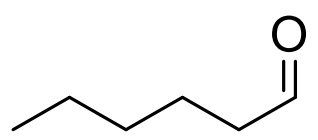	5.5	>99	182
**9**	**6**	0.1	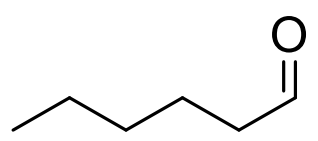	1	59	590
**10**	**6**	0.1	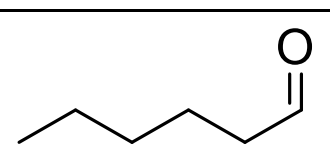	6.75	>99	148
**11**	**C**	0.01	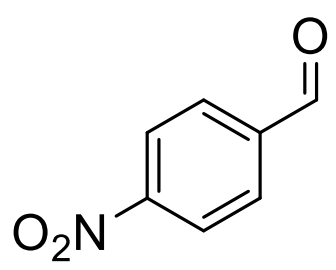	0.55	>99	18182
**12**	**3**	0.01	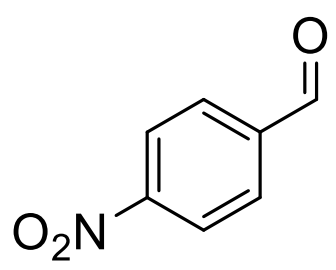	0.12	>99	83333
**13**	**3**	0.001	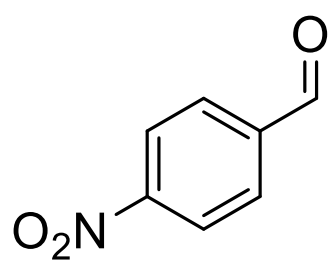	1	98	98000
**14**	**3**	0.001	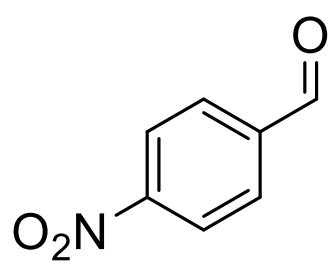	1.13	>99	88,500

**Entry**	**Catalyst**	**Catalytic load (mol%)**	**Substrate**	**t(h)**	**Conversion (%)**	**TOF (h^{-1})**
**15**	**4**	0.01	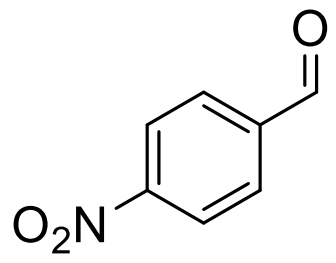	1	98	9800
**16**	**4**	0.01	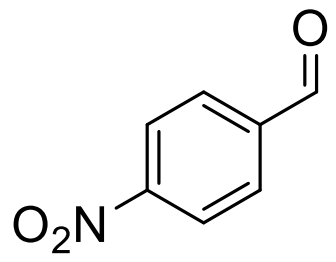	1.13	>99	8,850
**17**	**5**	0.01	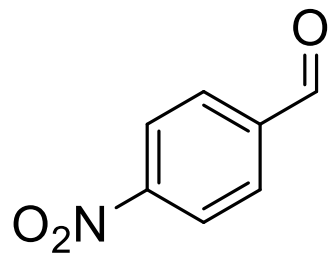	0.66	>99	15152
**18**	**6**	0.01	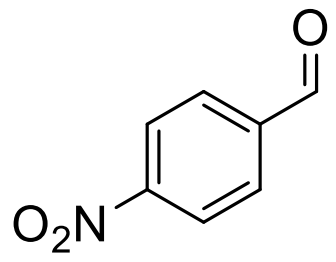	1	>99	10000
**19**	**no**	no	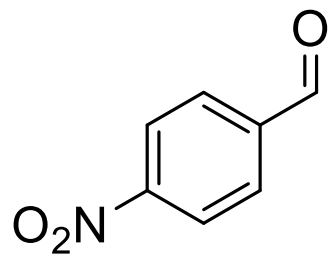	1	57	-
**20**	**no**	no	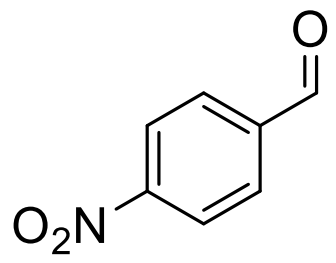	7	>99	-
**21**	**3**	0.1	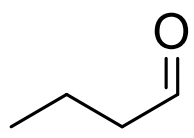	7.75	>99	129
**22**	**3**	0.1	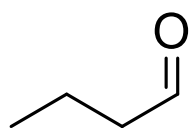	1	52	520
**23**	**3**	0.1	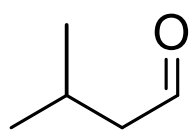	8.5	>99	118
**24**	**3**	0.1	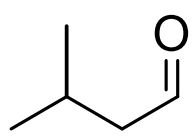	1	47	470
**25**	**3**	0.1	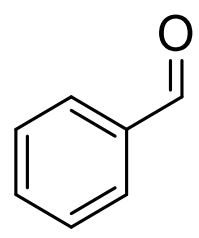	19	>99	53
**26**	**3**	0.1	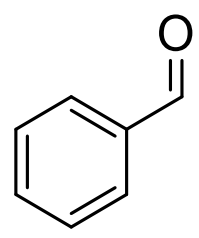	1	32	320
**27**	**3**	0.1	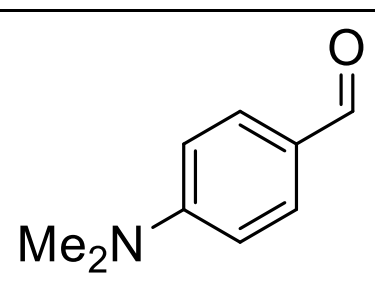	42	>99	24
**28**	**3**	0.1	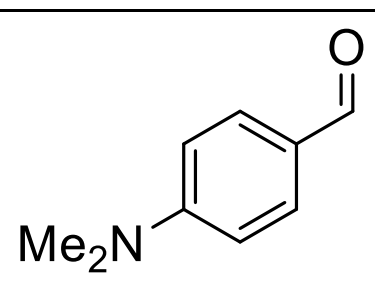	1	11	110

Characterization of products

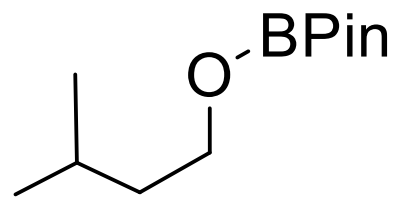

^{1}H NMR (400 MHz, C_6D_6): 3.96 (t, $^{3}J_{HH}$ = 6.6 Hz, 2H), 1.68 (dp, $^{3}J_{HH}$ = 13.4, 6.7 Hz, 1H), 1.44 (q, $^{3}J_{HH}$ = 6.7 Hz, 2H), 1.07 (s, 12H), 0.82 (d, $^{3}J_{HH}$ = 6.7 Hz, 6H).^{11}B NMR (128 MHz, C_6D_6): δ 22.52 (s).

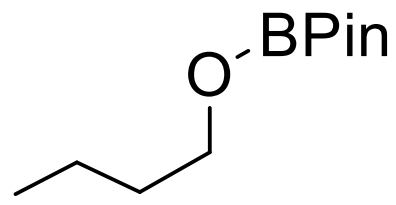

^{1}H NMR (400 MHz, C_6D_6): δ 3.91 (t, $^{3}J_{HH}$ = 6.5 Hz, 2H), 1.55 – 1.44 (m, 2H), 1.30 (dq, $^{3}J_{HH}$ = 14.4, 7.3 Hz, 2H), 1.07 (s, 12H), 0.80 (t, $^{3}J_{HH}$ = 7.4 Hz, 3H). ^{11}B NMR (128 MHz, C_6D_6): δ 22.54 (s).

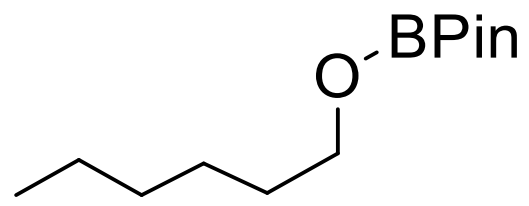

^{1}H NMR (400 MHz, C_6D_6): δ 3.90 (t, $^{3}J_{HH}$ = 6.5 Hz, 2H), 1.52 (m, 2H), 1.27 (m, 2H), 1.17 (m, 4H), 1.07 (s, 12H), 0.82 (t, $^{3}J_{HH}$ = 6.7 Hz, 3H). ^{11}B NMR (128 MHz, C_6D_6): δ 22.54 (s).

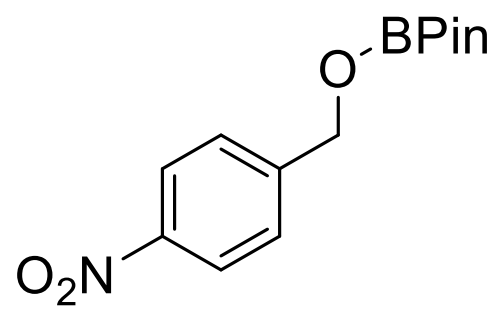

^{1}H NMR (400 MHz, C_6D_6): δ 7.80 (d, 3 $^{3}J_{HH}$ = 8.7 Hz, 2H), 6.92 (d, 3 $^{3}J_{HH}$ = 8.5 Hz, 2H), 4.69 (s, 2H), 1.05 (s, 12H). ^{11}B NMR (128 MHz, C_6D_6): δ 22.79 (s).ff

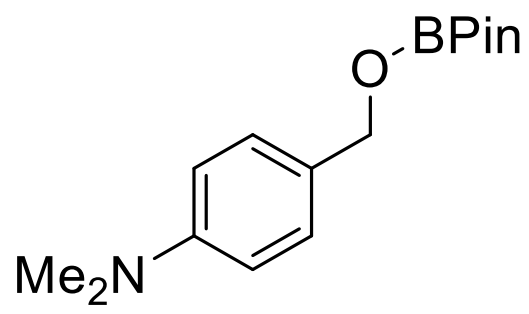

^{1}H NMR (400 MHz, C_6D_6): δ 7.33 (d, $^{3}J_{HH}$ = 8.8 Hz, 2H), 6.56 (d, $^{3}J_{HH}$ = 8.8 Hz, 2H), 4.99 (s, 2H), 2.51 (s, 6H), 1.07 (s, 12H). ^{11}B NMR (128 MHz, C_6D_6): δ 22.83 (s).

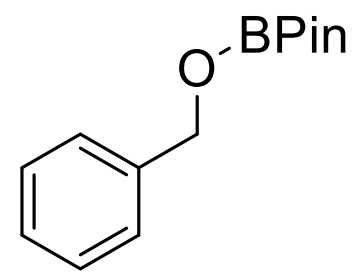

^{1}H NMR (400 MHz, C_6D_6): δ 7.33 – 7.26 (m, 2H), 7.14 (m, 2H), 7.07 (d, $^{3}J_{HH}$ = 7.3 Hz, 1H), 4.94 (s, 2H), 1.05 (s, 12H). ^{11}B NMR (128 MHz, C_6D_6): δ 22.80 (s).

Kinetic Studies

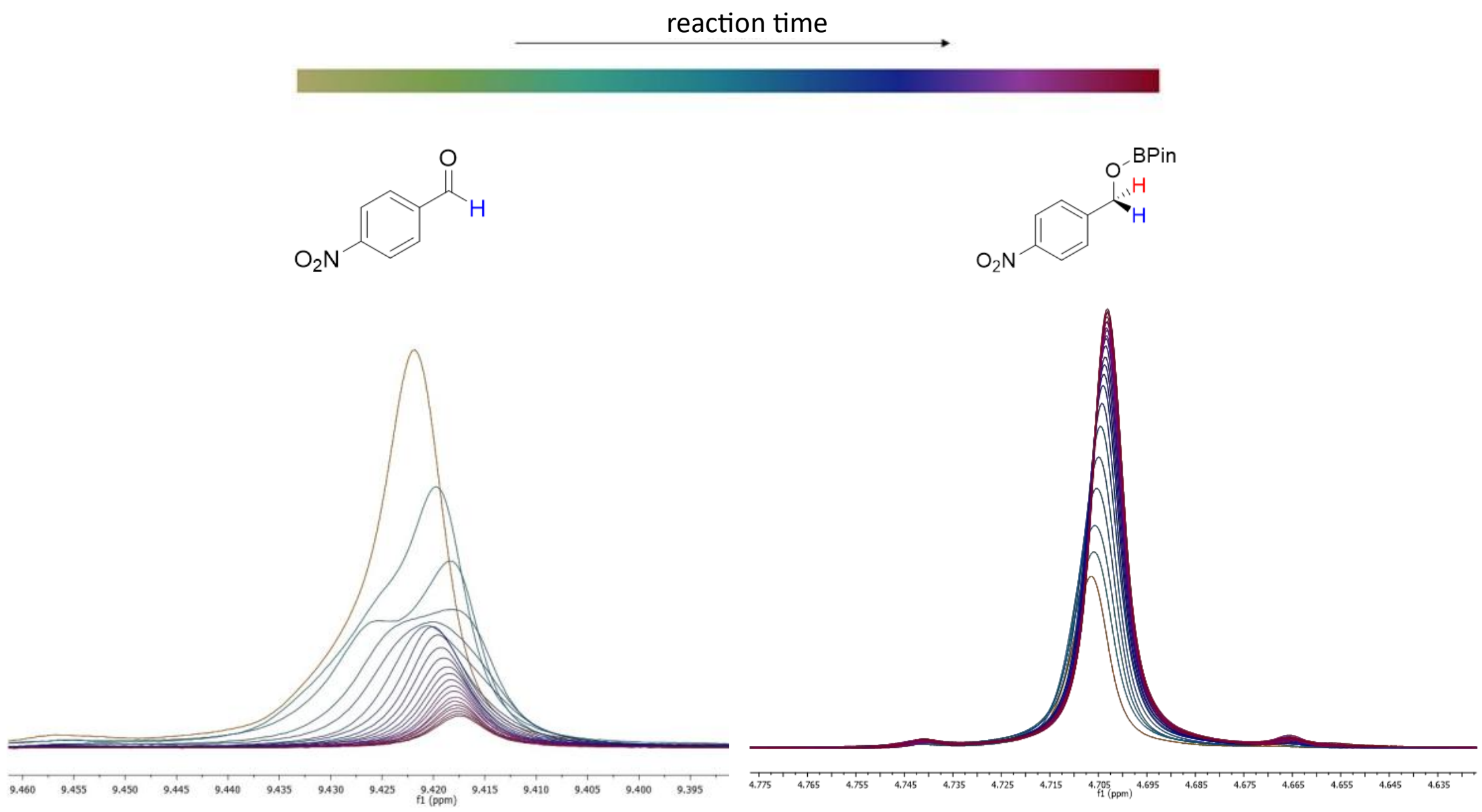

Figure S52. ^{1}H NMR spectra showing the conversion of *para*-nitrobenzaldehyde in C_6D_6 at 25 °C.

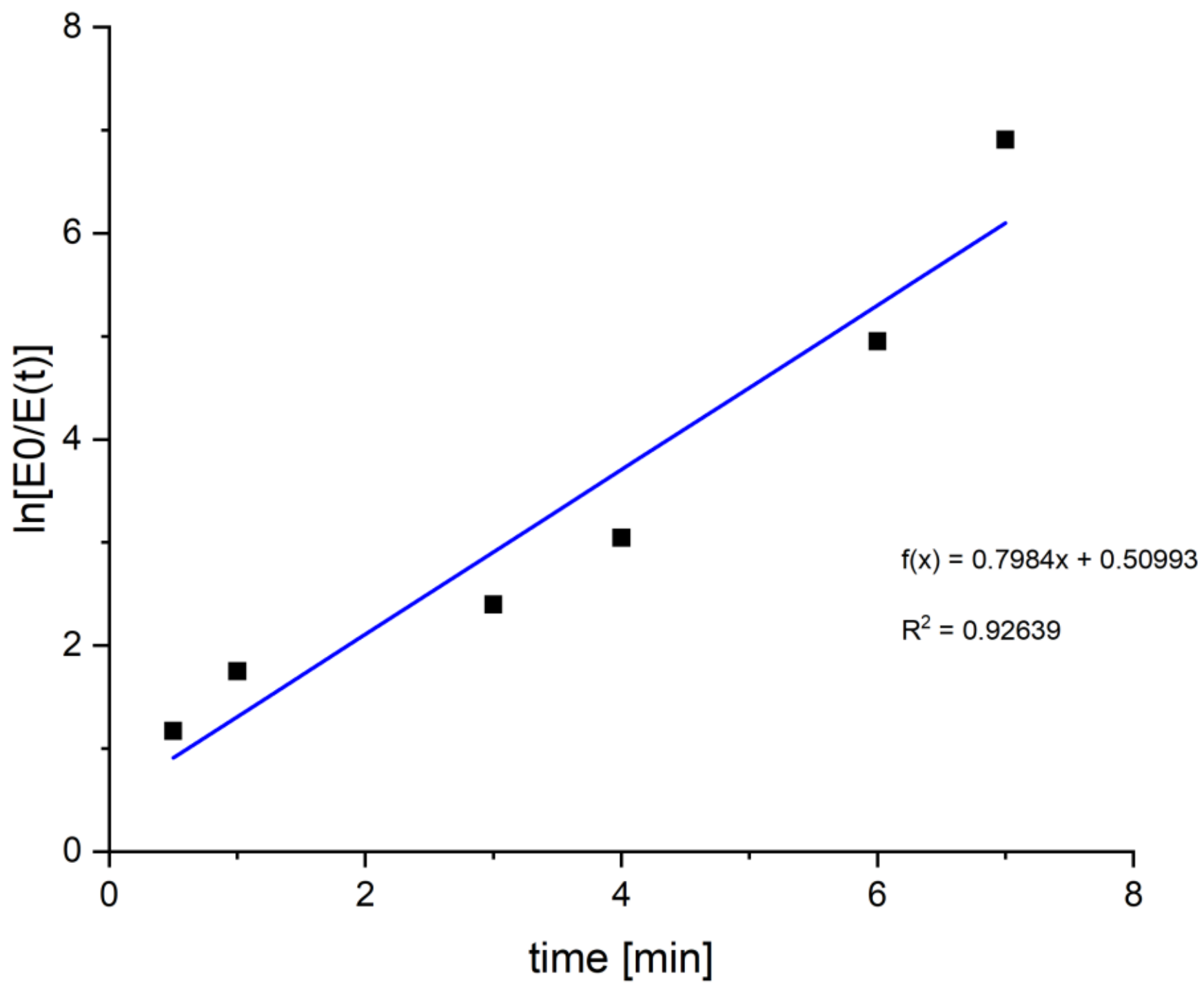

Figure S53. Plot of reaction kinetics (one equivalent *para*-nitrobenzaldehyde, HBPin and 0.01 mol% of **3**).

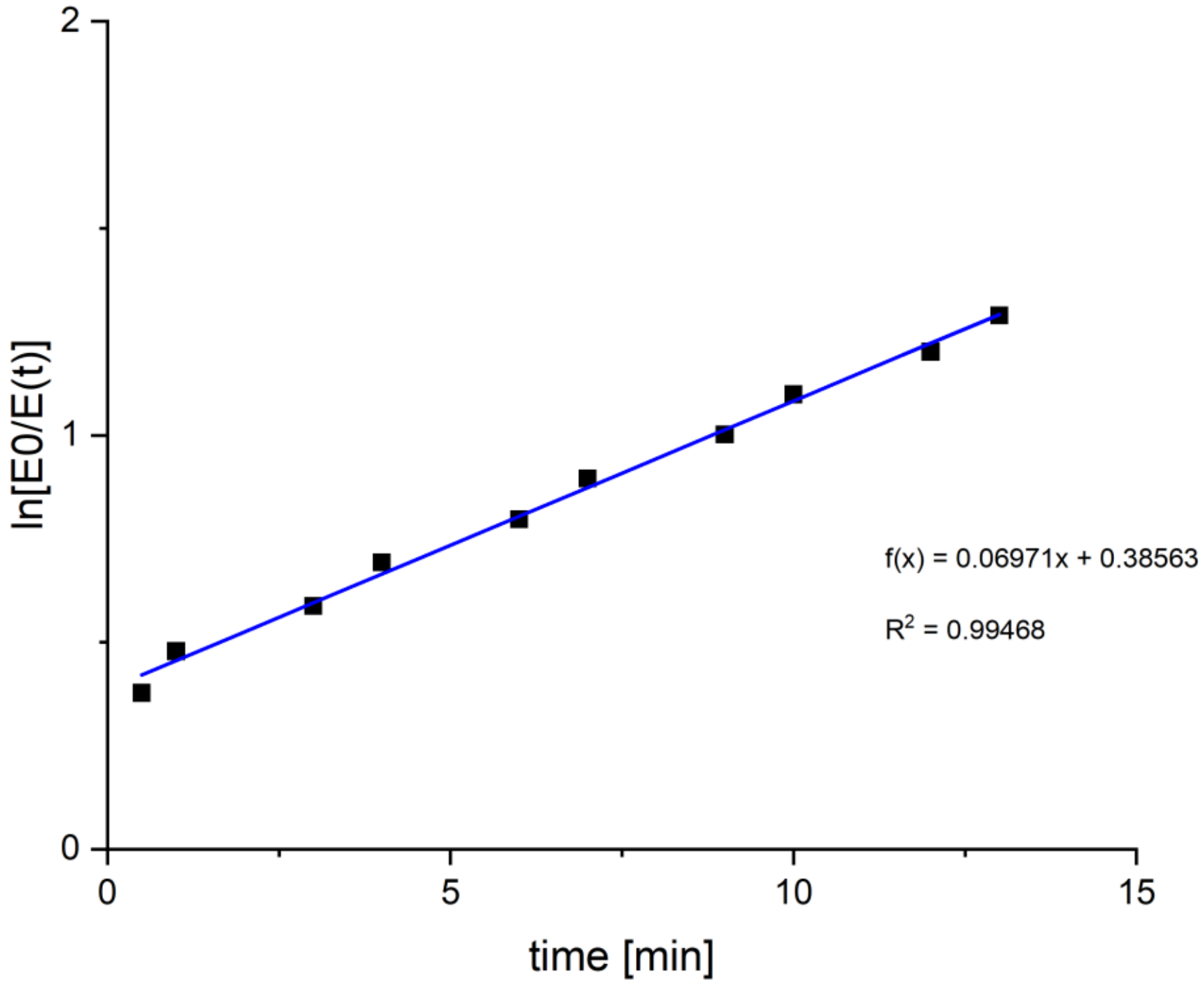

Figure S54. Plot of reaction kinetics (one equivalent *para*-nitrobenzaldehyde, HBPin and 0.01 mol% of **4**).

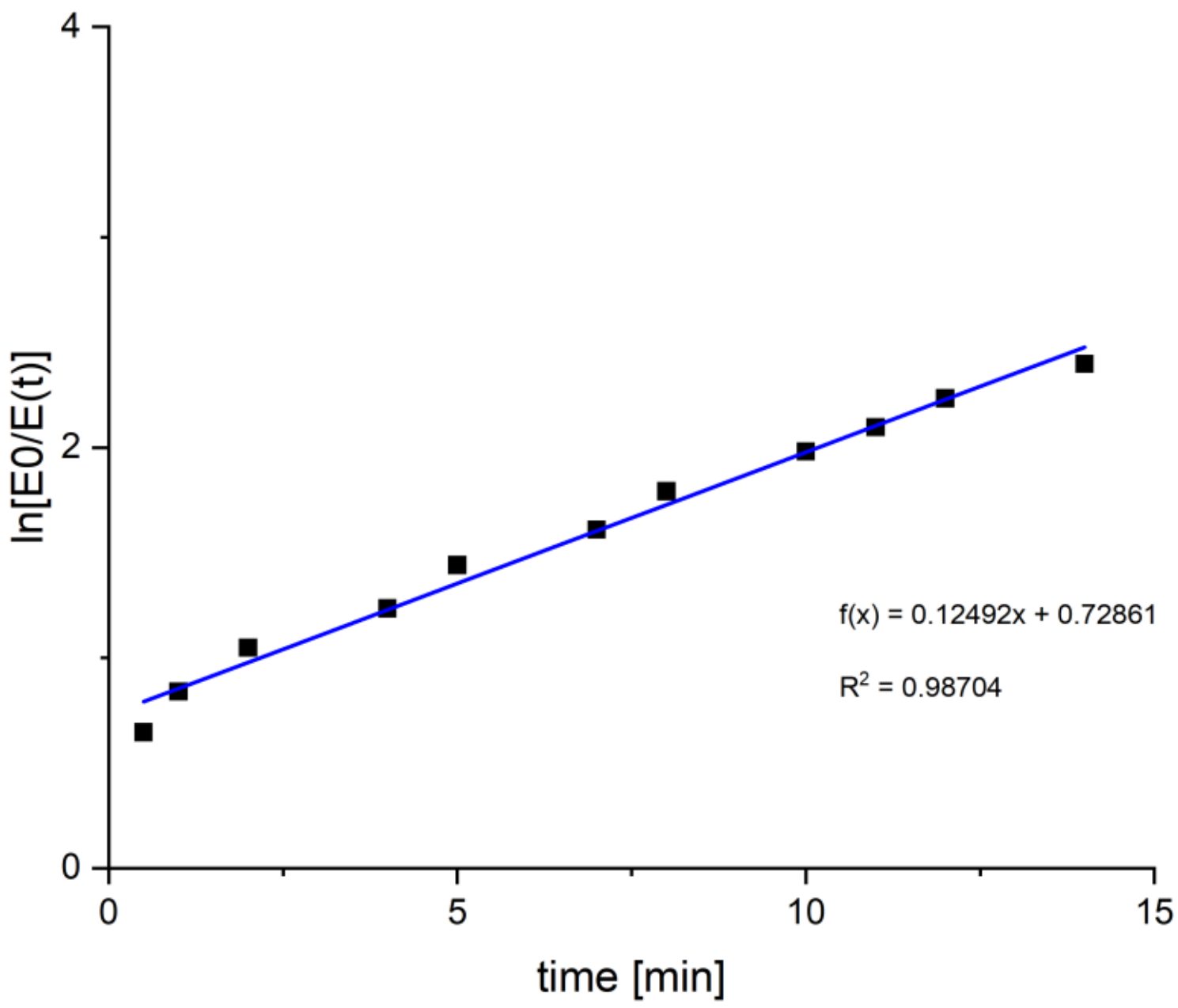

Figure S55. Plot of reaction kinetics (one equivalent *para*-nitrobenzaldehyde, HBPin and 0.01 mol% of **5**).

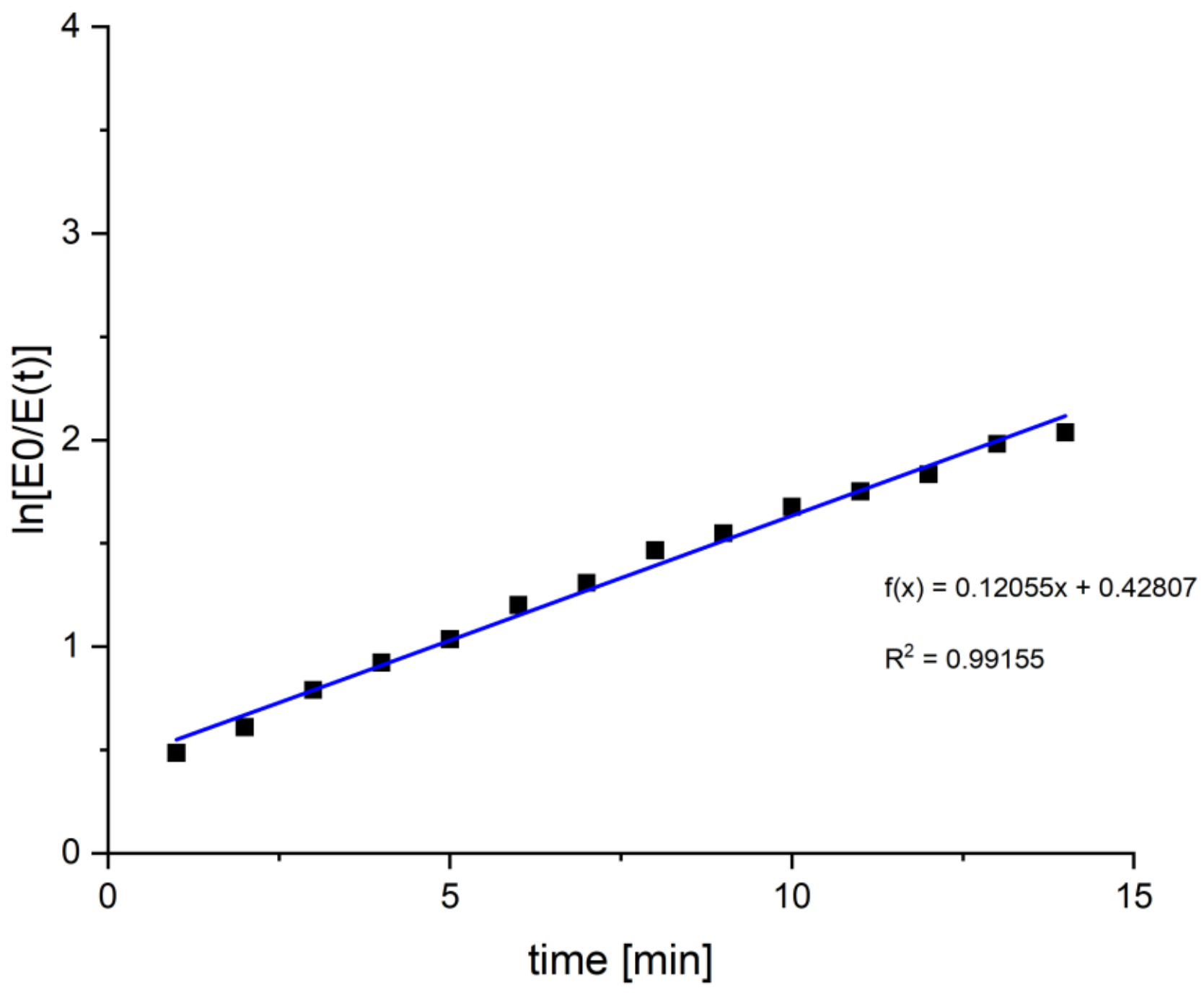

Figure S56. Plot of reaction kinetics (one equivalent *para*-nitrobenzaldehyde, HBPin and 0.01 mol% of **6**).

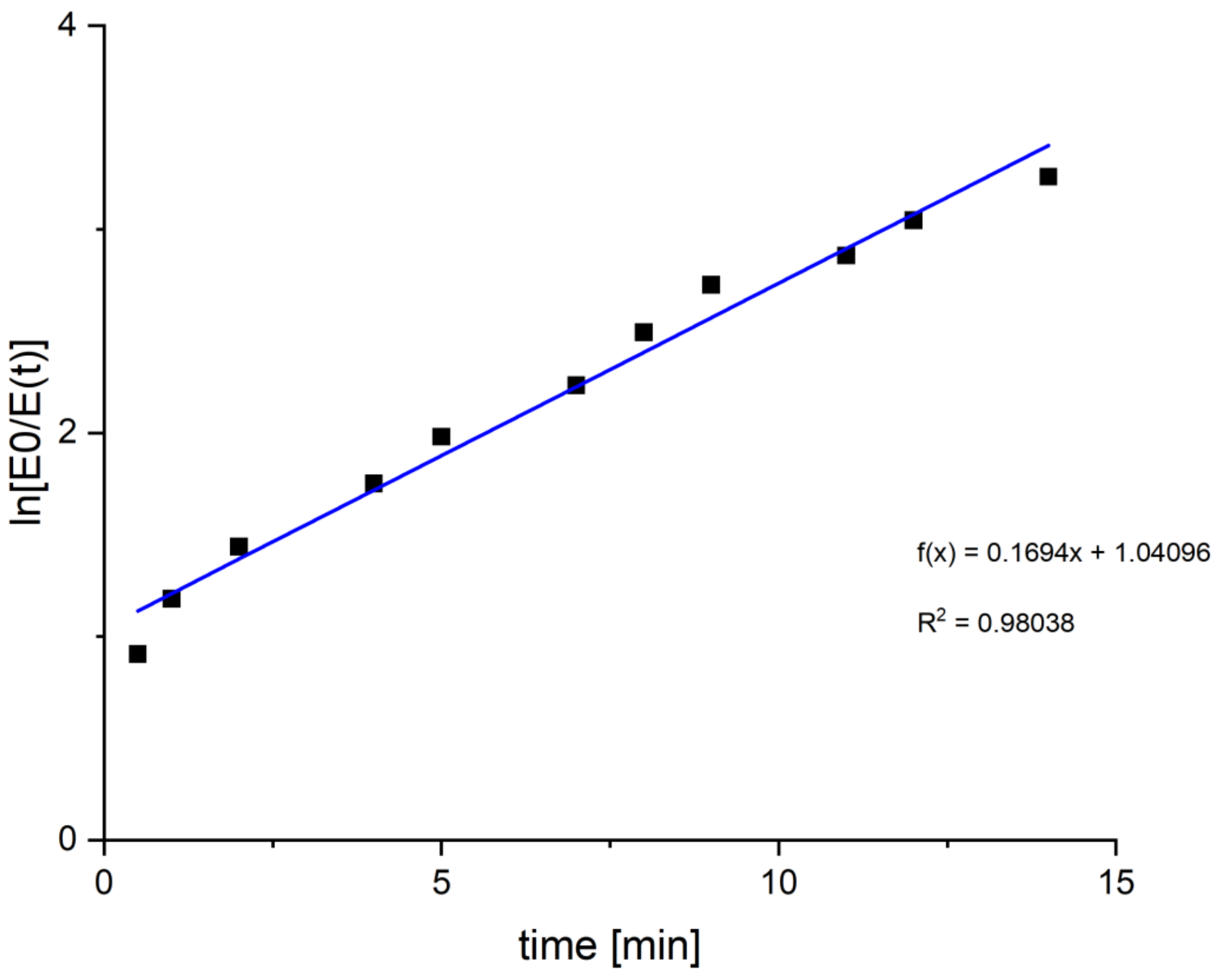

Figure S57. Plot of reaction kinetics (one equivalent *para*-nitrobenzaldehyde, HBPin and 0.01 mol% of **C**).

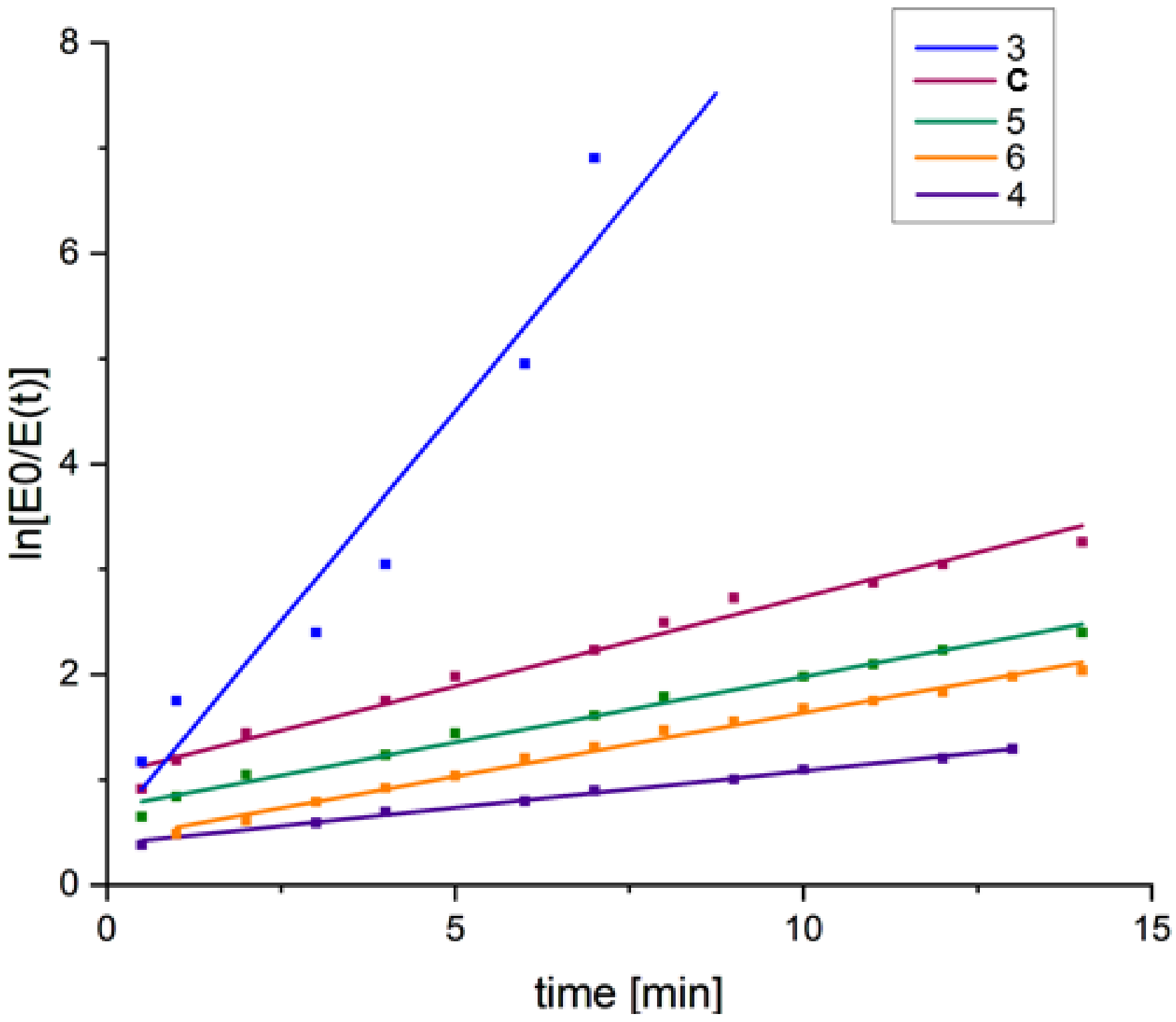

Figure S58. Stacked plot of reaction kinetics (one equivalent *para*-nitrobenzaldehyde, HBPin and 0.01 mol% of each catalyst).

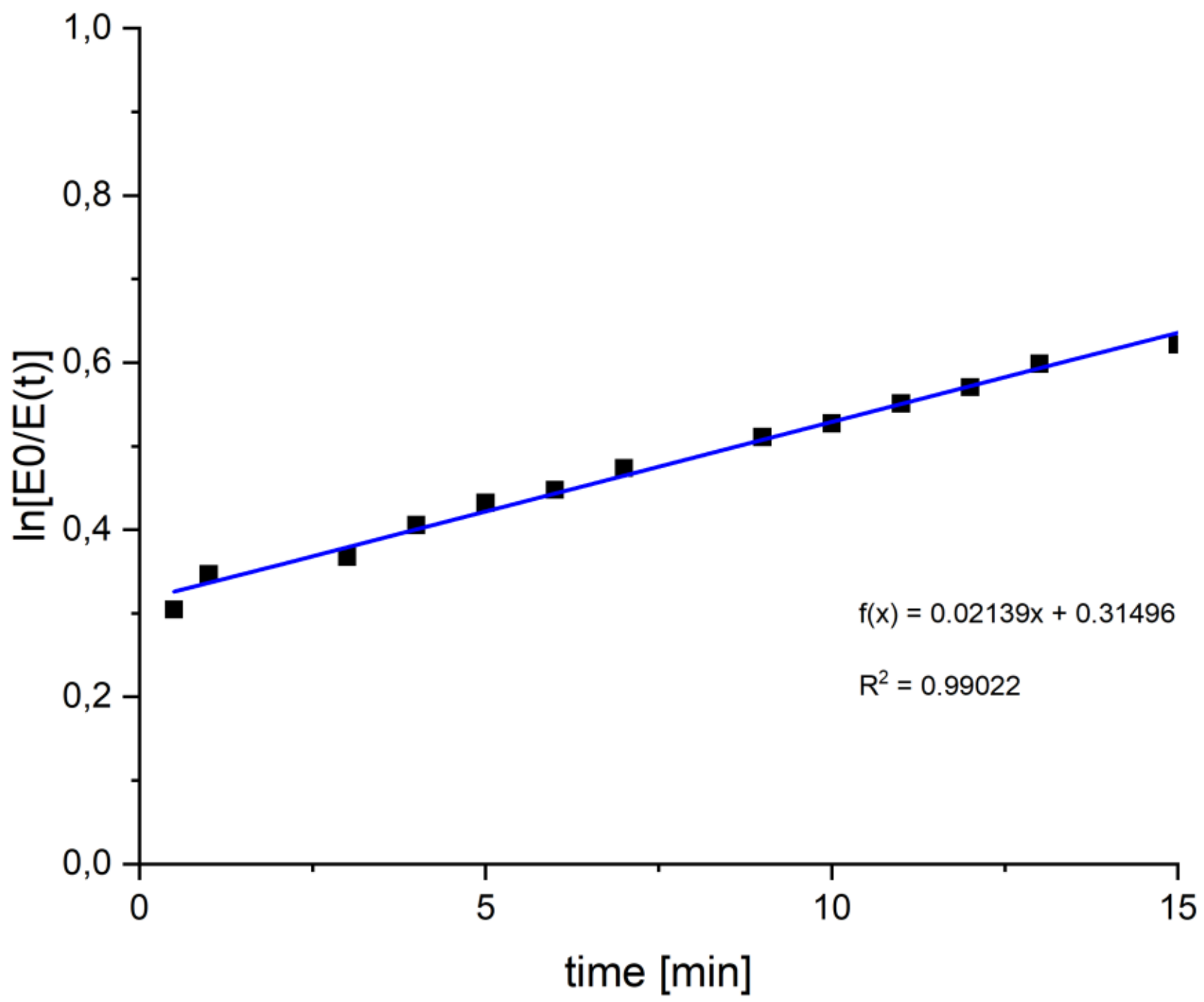

Figure S59. Plot of reaction kinetics (one equivalent *n*-hexanal, HBPin and 0.1 mol% of **3**).

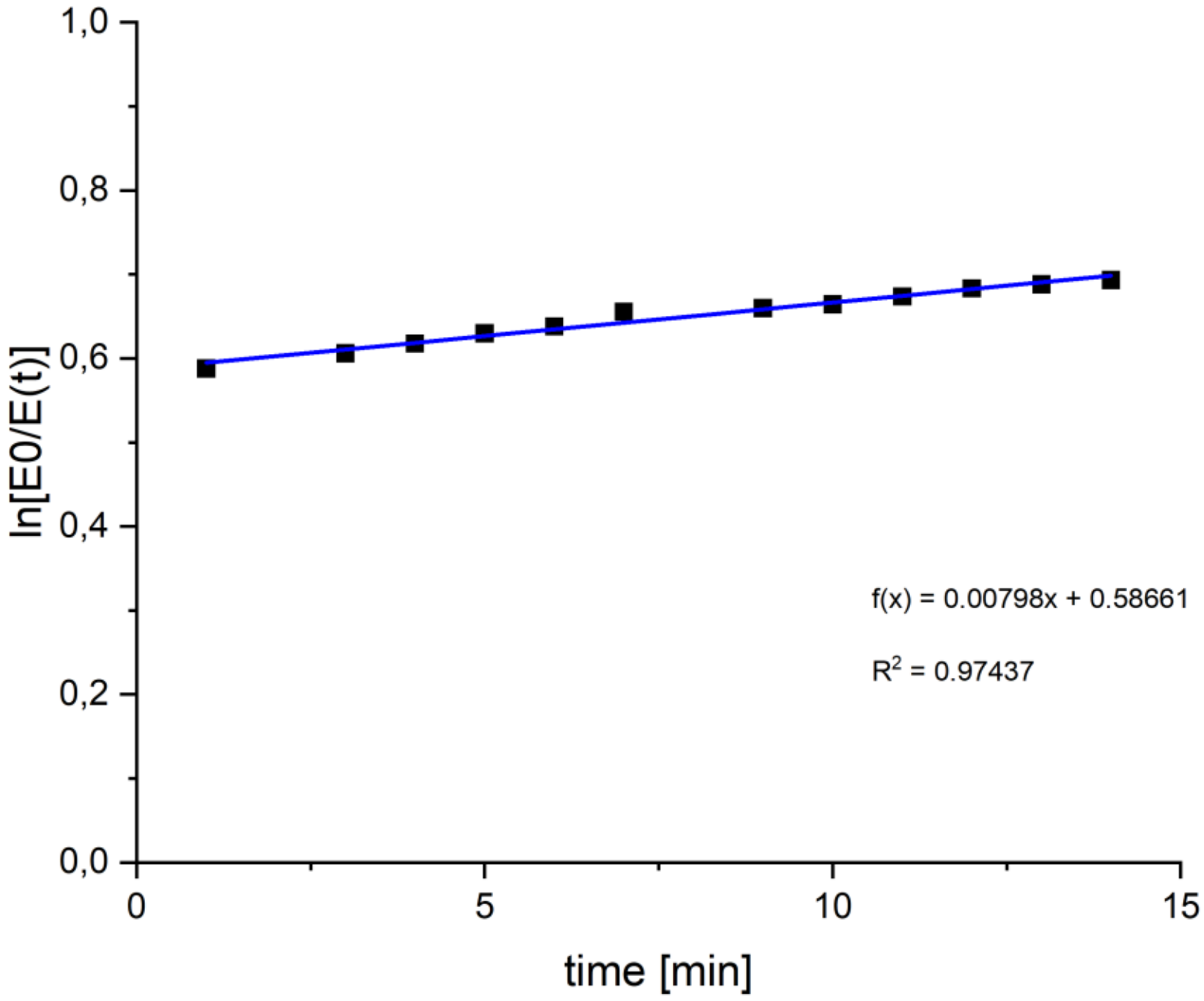

Figure S60. Plot of reaction kinetics (one equivalent *n*-hexanal, HBPin and 0.1 mol% of **4**).

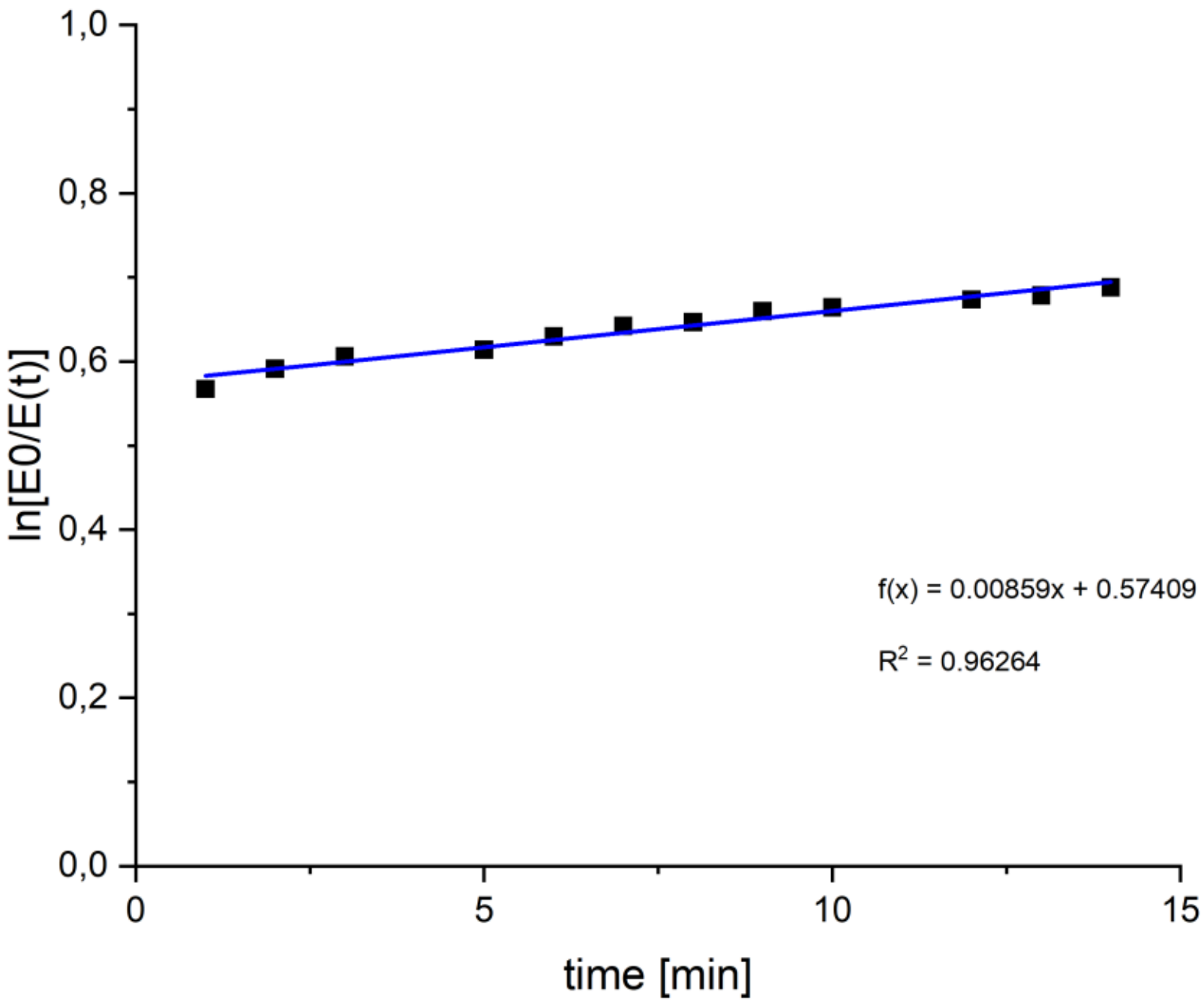

Figure S61. Plot of reaction kinetics (one equivalent *n*-hexanal, HBPin and 0.1 mol% of **5**).

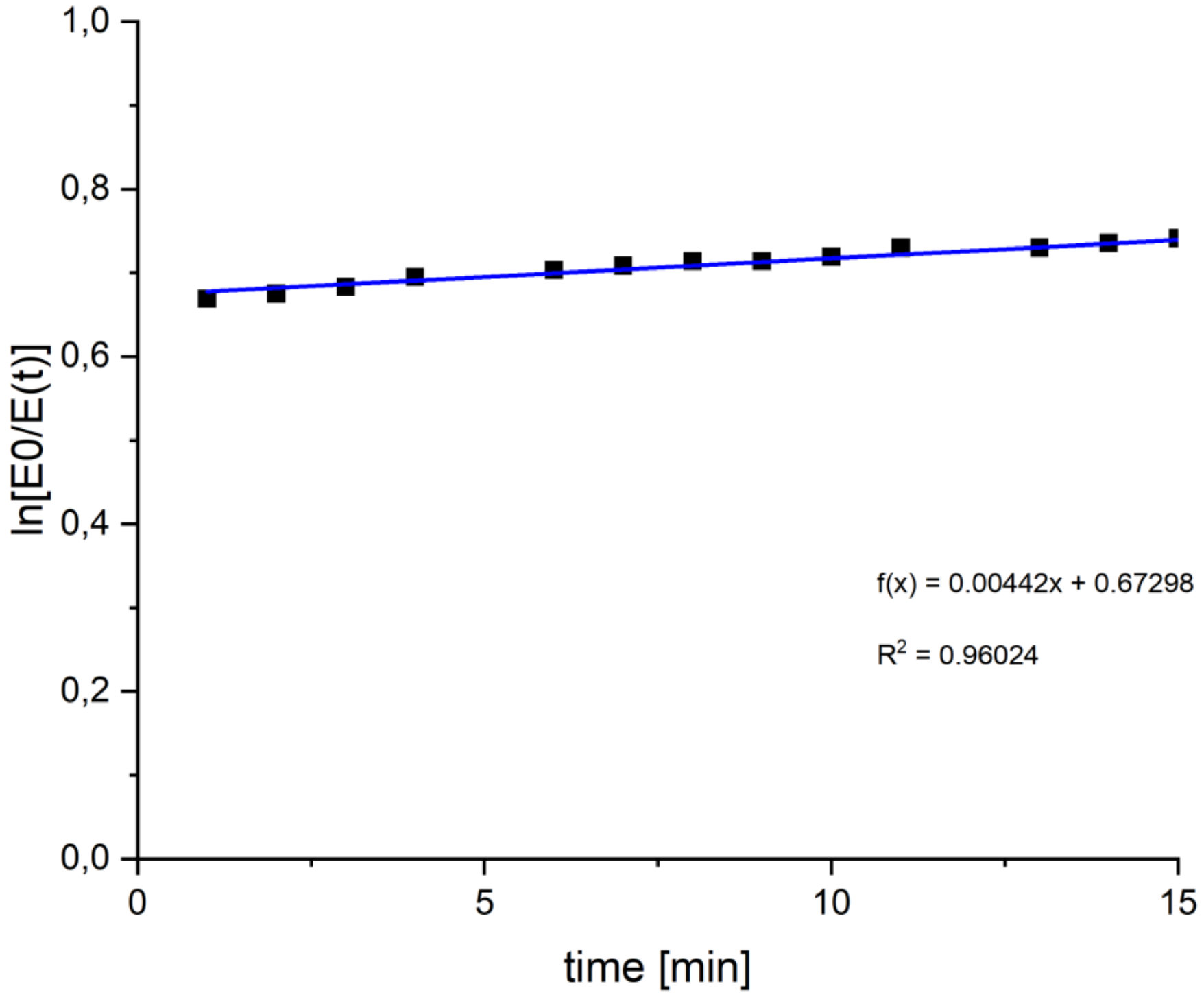

Figure S62. Plot of reaction kinetics (one equivalent *n*-hexanal, HBPin and 0.1 mol% of **6**).

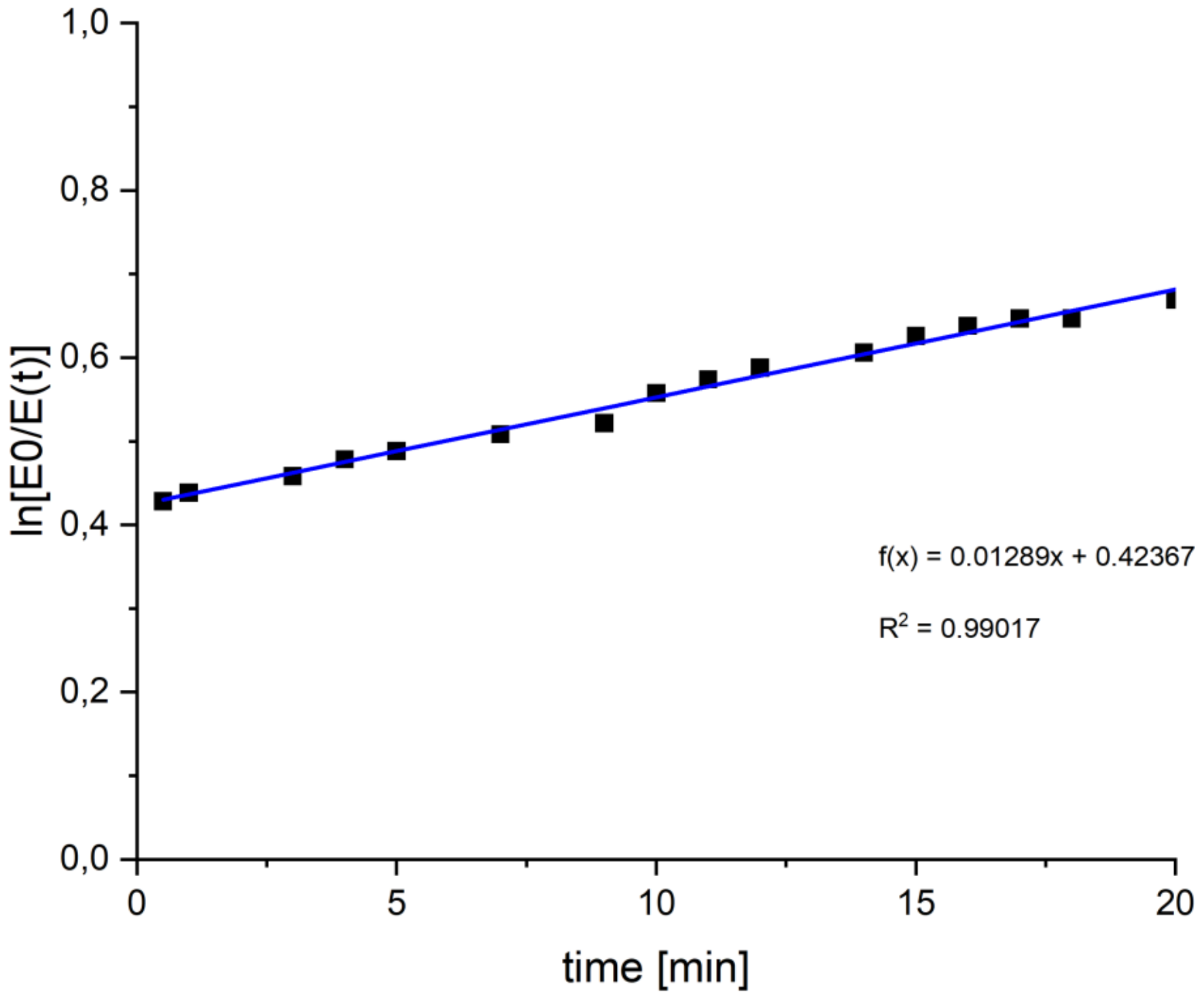

Figure S63. Plot of reaction kinetics (one equivalent *n*-hexanal, HBPin and 0.1 mol% of **C**).

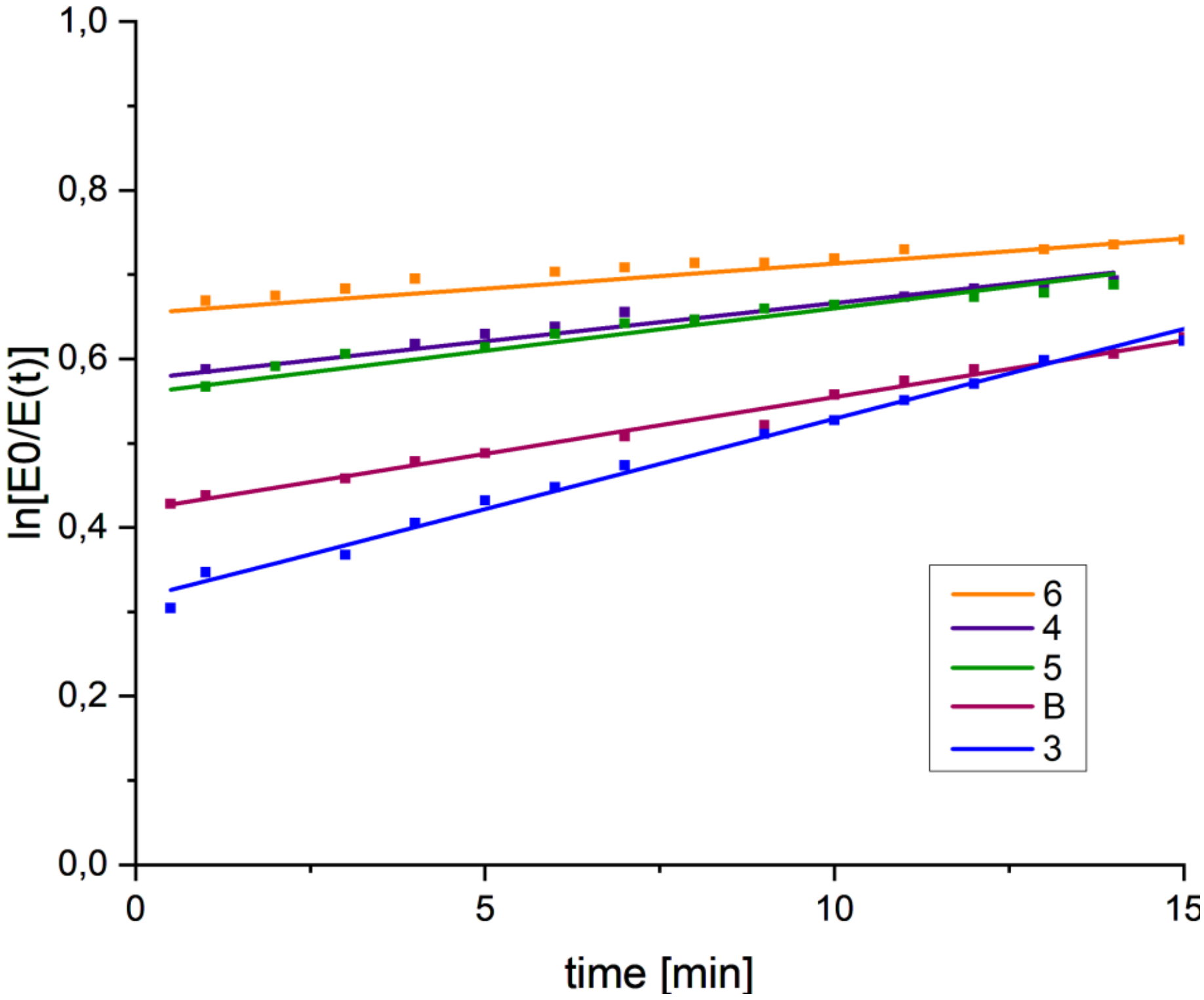

Figure S64. Stacked plot of reaction kinetics (one equivalent *n*-hexanal, HBPin and 0.1 mol% of each catalyst).

To determine the activation parameters E_a and $\Delta G^{\ddagger}$ from the experimental data, temperature-dependent kinetic measurements were conducted using *para*-nitrobenzaldehyde, HBPin, and 0.001 mol% of catalyst **3**. The results obtained from these experiments are summarized in Table S2. Based on the resulting temperature-dependent rate constants k, the activation parameters were determined using Arrhenius (S57) and Eyring (S58) analyses, respectively. The corresponding activation parameters were determined to be E_a = 14.6 kcal/mol and $\Delta G^{\ddagger}$ = 20.1 kcal/mol.

Table S2. Catalytic hydroboration of *para*-nitrobenzaldehyde and HBPin with 0.001 mol% of **3** at different temperatures.

Entry	Temperature (°C)	t(h)	Conversion (%)	Conversion after 15 min (%)	TOF (h^{-1})
**1**	25	1	98	32	98,000
**2**	35	1	>99	47	100,00
**3**	45	0.58	>99	78	172,414
**4**	55	0.42	>99	90	238,095
**5**	65	0.25	>99	>99	400,000

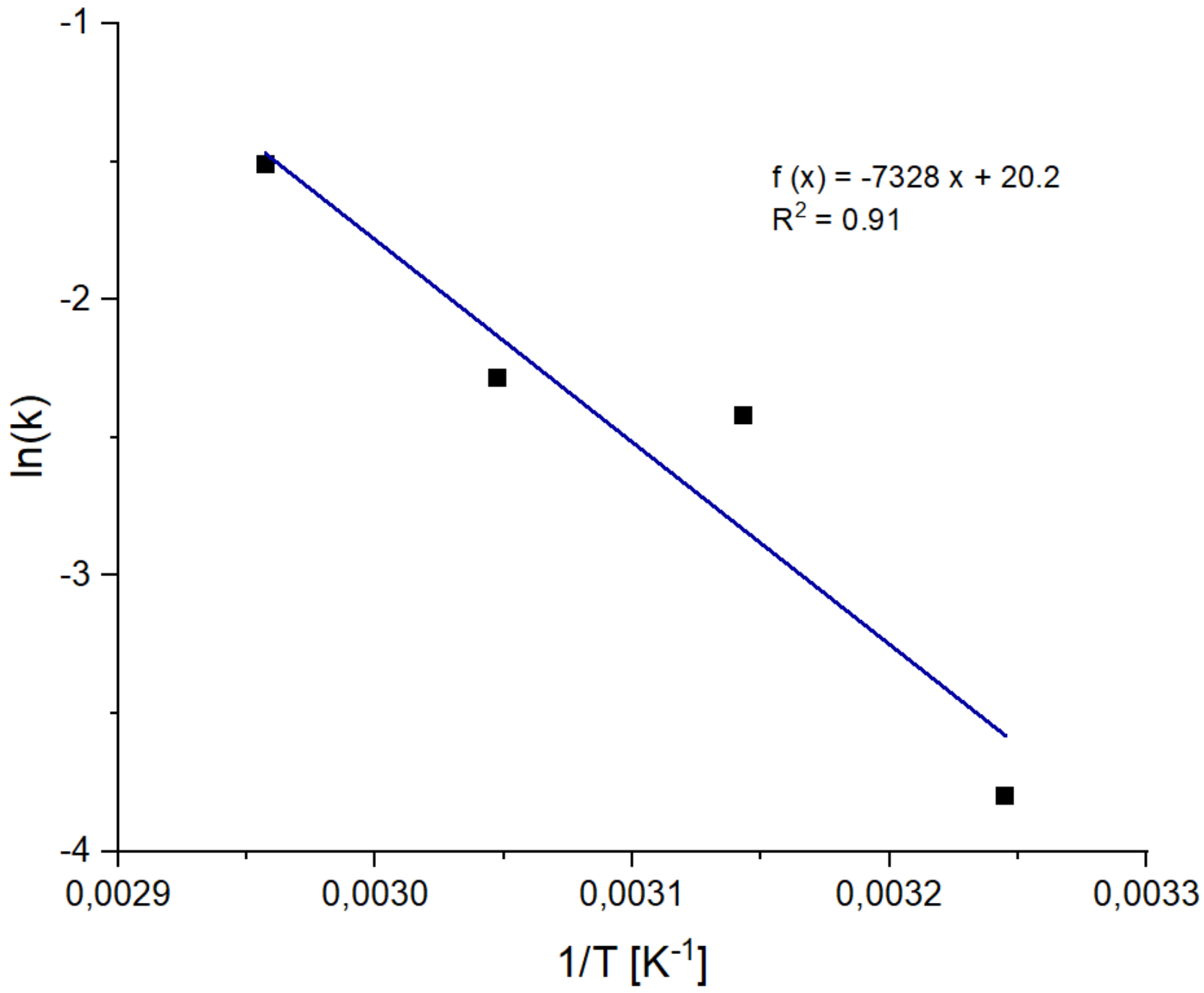

Figure S65. Arrhenius plot of reaction kinetics (one equivalent *para*-nitrobenzaldehyde, HBPin and 0.001 mol% of **3** at different temperatures).

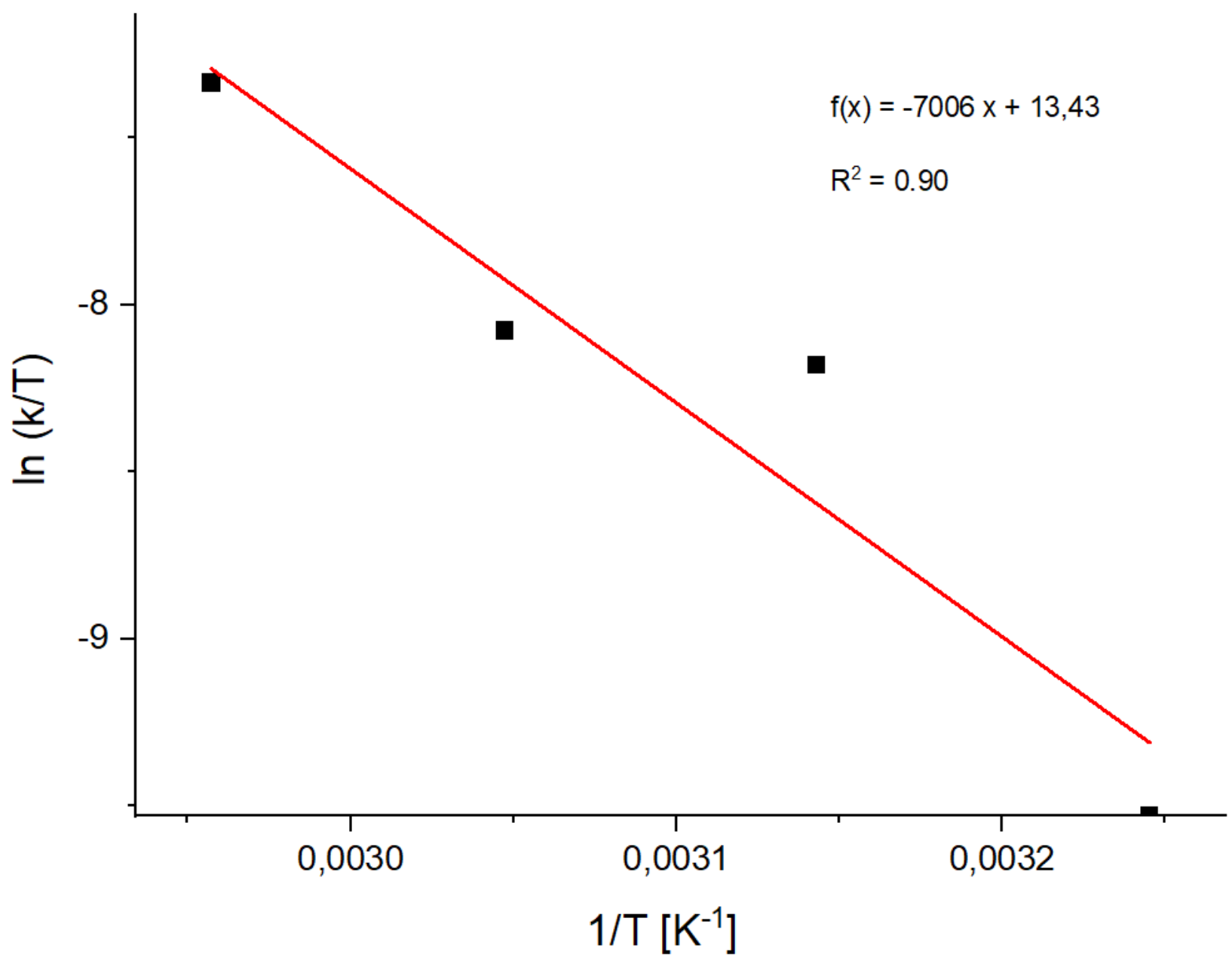

Figure S66. Eyring plot of reaction kinetics (one equivalent *para*-nitrobenzaldehyde, HBPin and 0.001 mol% of **3** at different temperatures).

III. Single Crystal X-ray Diffraction

The crystals were mounted on nylon loops in inert oil. Data of **2**, **3**, and **8** were collected on a Bruker AXS D8 Kappa diffractometer with APEX2 detector (monochromated Mo$_{K\alpha}$ radiation, λ = 0.71073 Å) while those of **4**, **5**, **6**, and **7** were collected on a Bruker AXS D8 Venture diffractometer with Photon II detector (monochromated Cu$_{K\alpha}$ radiation, λ= 1.54178 Å, microfocus source) at 100(2) K. The structures were solved by Direct Methods (SHELXS-2013)[7] and refined anisotropically by full-matrix least-squares on F^2 (SHELXL-2017)[8,9]. Absorption corrections were performed semi-empirically from equivalent reflections on basis of multi-scans and numerical from indexed faces (**4**) (Bruker AXS APEX3). Hydrogen atoms were refined using a riding model or rigid methyl groups.

The structure of **2** contains a mixture of diasteromers resulting in a disorder of Si1 and O1. The solvent molecules are disordered over centers of inversion. Their bond lengths and the angles of the ring were restrained to be equal (SADI) and RIGU restraints were applied to their displacement parameters.

In **3** the ethyl group is highly disordered and could only crudely be modelled by three alternate positions. Its bond lengths and angles were restrained to be equal (SADI) and RIGU and SIMU restraints were applied to its atoms' displacement parameters. Positions in proximity were refined with common displacement parameters (EADP) to avoid correlations. The intensities at resolutions > 1 Å were very weak and a high percentage < $2\sigma(I)$. Consequently, the model should be carefully interpreted and any conclusions drawn from it supported by other means. Quantitative results may be unreliable.

The crystal of **4** was a non-merohedral and the model refined as a 2-component twin against HKLF5 data obtained from PLATON (A. L. Spek, 2018).

In **5** the bromo phenyl group – and correlated an isopropyl group – shows a minor disorder component of approx. 5% occupancy. The corresponding bond lengths and angles were retrained to be equal (SADI) and RIGU and SIMU restraints were applied to the displacement parameters of the bromo phenyl group's atoms. Due to the low occupancy the minor components of the groups were refined with common displacement parameters (EADP) to avoid non-positive definites. The Cl ligand at the Ga1 atom is replaced by a Br ligand with an occupancy of approx. 5%.

An *n*-hexyl group of **6** is disordered. It could be crudely modelled by two alternate positions but the displacement parameters of the atoms at the end of the chain suggest further disorder that cannot be modelled. The corresponding bond lengths were restrained to be equal (SADI) and RIGU restraints were applied to the displacement parameters.

The structure of **7** contains a mixture of diastereomers leading to a disorder of everything but the L^1GaCl moiety. All corresponding bond lengths and most angles of the disordered part were restrained to be equal (SADI). Strongly correlated displacements were constrained to be equal (EADP) and a global RIGU restraint was applied. The displacement parameters of the benzyl group were refined with an additional SIMU restraint. To avoid correlations, the occupancies were constrained to the values of the free variables in the final refinement. Due to the disorder and the necessary vast use of re- and constraints any results beyond the connectivity should not be discussed. In addition, the structure contains a highly disordered difluorobenzene molecule. The final refinement was done with a solvent free dataset from a PLATON/SQUEEZE run. (For details see: A. L. Spek, *Acta Cryst. A46* (1990), 194{201). The molecule was included in the sum formula for completeness.

In **8** a phenyl ring is disordered over two positions. Its bond lengths and angles were restrained to be equal (SADI) and RIGU and SIMU restraints were applied to its displacement parameters. Strongly correlated displacements were refined with equal displacement parameters (EADP). The ring was restrained to planarity (FLAT). The crystal diffracted rather weakly and might have been modulated. Consequently, quantitative results should be carefully assessed.

CCDC-2481719 (**2** (lk_129m)), -2481720 (**3** (lk_061m)), -2481721 (**4** (lk_33m_tw)), -2481722 (**5** (lk_037fm)), -2481728 (**6** (lk_040am)), -2481729 (**7** (lk_122m_sq)), and -2481730 (**8** (lk_125m)) contain the supplementary crystallographic data for this paper. These data can be obtained free of charge from The Cambridge Crystallographic Data Centre via www.ccdc.cam.ac.uk/data_request/cif.

Table S3. Crystal data and structure refinement of **2 – 5**.

**Identification code**	**2 (lk_129m)**	**3 (lk_061m)**	**4 (lk_33m_tw)**	**5 (lk_037fm)**
Empirical formula	$C_{62}H_{88}ClGaN_4OSi$	$C_{47}H_{70}ClGaN_4OSi$	$C_{55.50}H_{74.50}ClGaN_4OSi$	$C_{57}H_{75}Br_{1.03}Cl_{0.97}GaN_4OSi$
M	1038.62	840.33	946.95	1046.90
Crystal size [mm]	0.478 × 0.268 × 0.139	0.172 × 0.109 × 0.103	0.321 × 0.259 × 0.073	0.185×0.130×0.060
T [K]	100(2)	100(2)	100(2)	100(2)
Crystal system	monoclinic	monoclinic	monoclinic	triclinic
Space group	$P2_1/n$	$P2_1/n$	$P2_1/c$	P-1
a [Å]	12.4552(13)	16.386(3)	23.5375(10)	12.5406(8)
b [Å]	39.860(4)	14.769(3)	16.3391(7)	13.9081(8)
c [Å]	12.5293(12)	19.494(4)	27.6367(12)	15.8612(9)
α [°]	90	90	90	88.122(3)
β [°]	114.459(4)	93.003(5)	99.619(3)	86.473(3)
γ [°]	90	90	90	76.507(3)
V [Å^3]	5662.1(10)	4711.1(17)	10479.1(8)	2684.5(3)
Z	4	4	8	2
D_{calc} [g·cm^{-3}]	1.218	1.185	1.200	1.295
μ(MK_α [mm^{-1}])	0.598(Mo)	0.703(Mo)	1.713(Cu)	2.575(Cu)
Transmissions	0.75/0.65	0.75/0.62	0.75/0.49	0.75/0.62
F(000)	2232	1800	4044	1101
Index ranges	$-19 \leq h \leq 19$	$-21 \leq h \leq 21$	$-30 \leq h \leq 29$	$-16 \leq h \leq 16$
	$-61 \leq k \leq 61$	$-19 \leq k \leq 19$	$-20 \leq k \leq 20$	$-15 \leq k \leq 17$
	$-19 \leq l \leq 19$	$-26 \leq l \leq 26$	$-10 \leq l \leq 35$	$-20 \leq l \leq 20$
θ_{max} [°]	33.394	28.385	80.970	80.606
Reflections collected	473045	110167	453993	153831
Independent reflections	21912	11770	22867	11653
R_{int}	0.0493	0.1404	0.0996	0.0623
Refined parameters	731	542	1177	652
R_1 [$I > 2\sigma(I)$]	0.0376	0.0695	0.0601	0.0298
wR_2 [all data]	0.0897	0.1656	0.1556	0.0736
GooF	1.160	1.130	1.149	1.048
$\Delta\rho_{final}$ (max/min) [e·Å^{-3}]	0.791/-0.519	1.641/-0.831	1.456/-0.865	0.408/-0.461

Table S4. Crystal data and structure refinement of **6 – 8**.

**Identification code**	**6 (lk_040am)**	**7 (lk_122m_sq)**	**8 (lk_125m)**
Empirical formula	$C_{57}H_{82}ClGaN_4OSi$	$C_{68}H_{90}ClF_2GaN_4O_2Si$	$C_{68}H_{99}BClGaN_4O_4Si$
M	972.52	1166.69	1180.58
Crystal size [mm]	0.187×0.115×0.055	0.207 × 0.189 × 0.096	0.231 × 0.214 × 0.146
T [K]	100(2)	100(2)	100(2)
Crystal system	monoclinic	monoclinic	monoclinic
Space group	$P2_1/c$	$P2_1/c$	$P2_1/c$
a [Å]	16.5470(6)	13.5895(14)	20.843(4)
b [Å]	27.8294(11)	15.4111(16)	51.604(9)
c [Å]	23.6698(9)	30.176(3)	18.959(3)
α [°]	90	90	90
β [°]	90.3004(19)	100.2978(19)	102.312(3)
γ [°]	90	90	90
V [Å^3]	10899.6(7)	6218.0(11)	19923(6)
Z	8	4	12
D_{calc} [g·cm^{-3}]	1.185	1.246	1.181
μ(MK_α [mm^{-1}])	1.657(Cu)	0.558(Mo)	0.521
Transmissions	0.75/0.68	0.75/0.70	0.75/0.67
F(000)	4176	2488	7608
Index ranges	$-21 \leq h \leq 19$	$-19 \leq h \leq 19$	$-29 \leq h \leq 29$
	$-35 \leq k \leq 35$	$-22 \leq k \leq 22$	$-73 \leq k \leq 73$
	$-29 \leq l \leq 30$	$-43 \leq l \leq 43$	$-27 \leq l \leq 27$
θ_{max} [°]	79.621	30.588	30.607
Reflections collected	549126	173389	531193
Independent reflections	23602	19103	61028
R_{int}	0.0818	0.0772	0.1619
Refined parameters	1252	882	2258
R_1 [$I > 2\sigma(I)$]	0.0301	0.0623	0.0794
wR_2 [all data]	0.0781	0.1293	0.1670
GooF	1.016	1.168	1.094
$\Delta\rho_{final}$ (max/min) [e·Å^{-3}]	0.974/-0.538	0.691/-0.865	0.979/-0.907

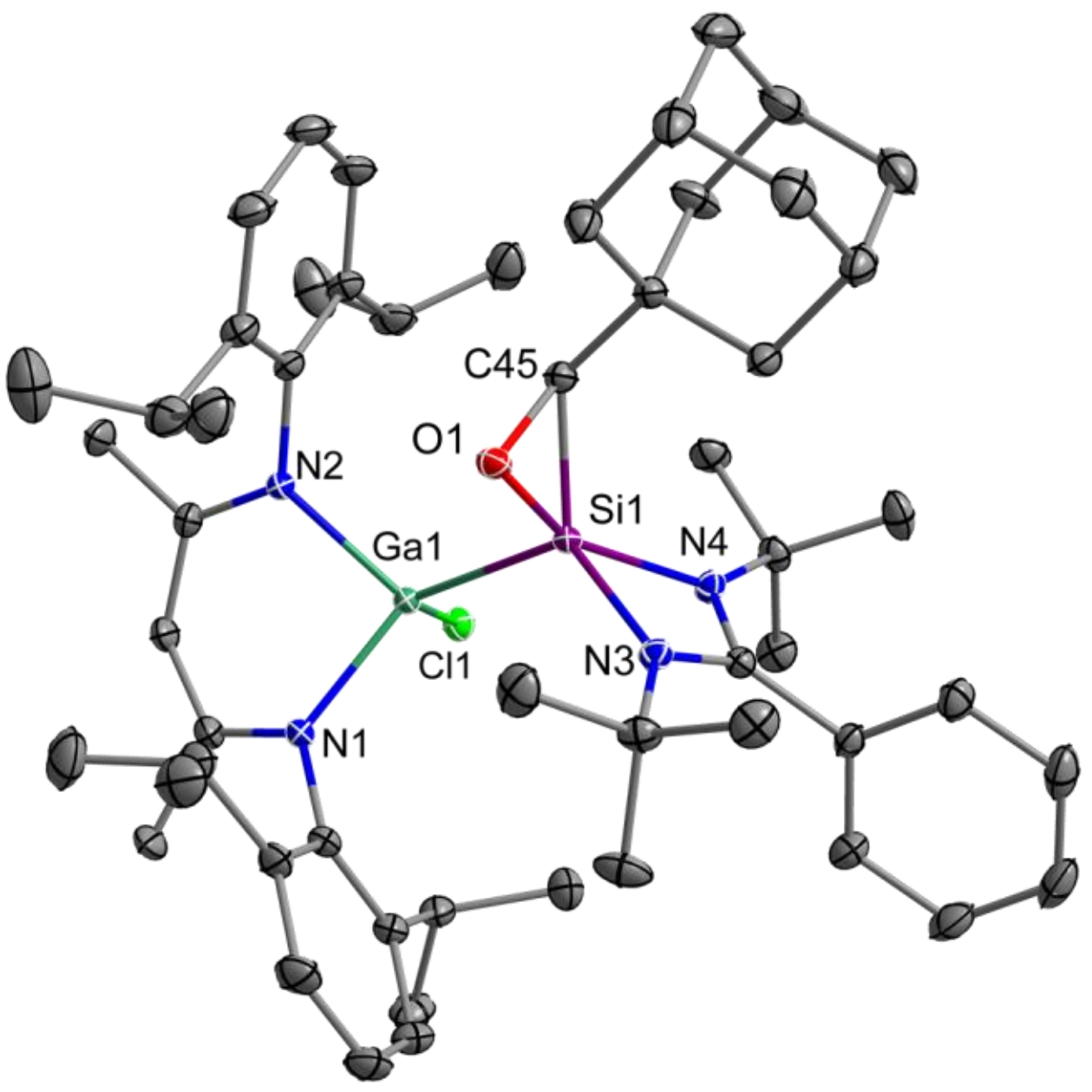

Figure S67. Molecular structure of **2** with thermal ellipsoids at 50% probability level. The hydrogen atoms, solvent molecules and the alternate position of the disordered parts are omitted for clarity.

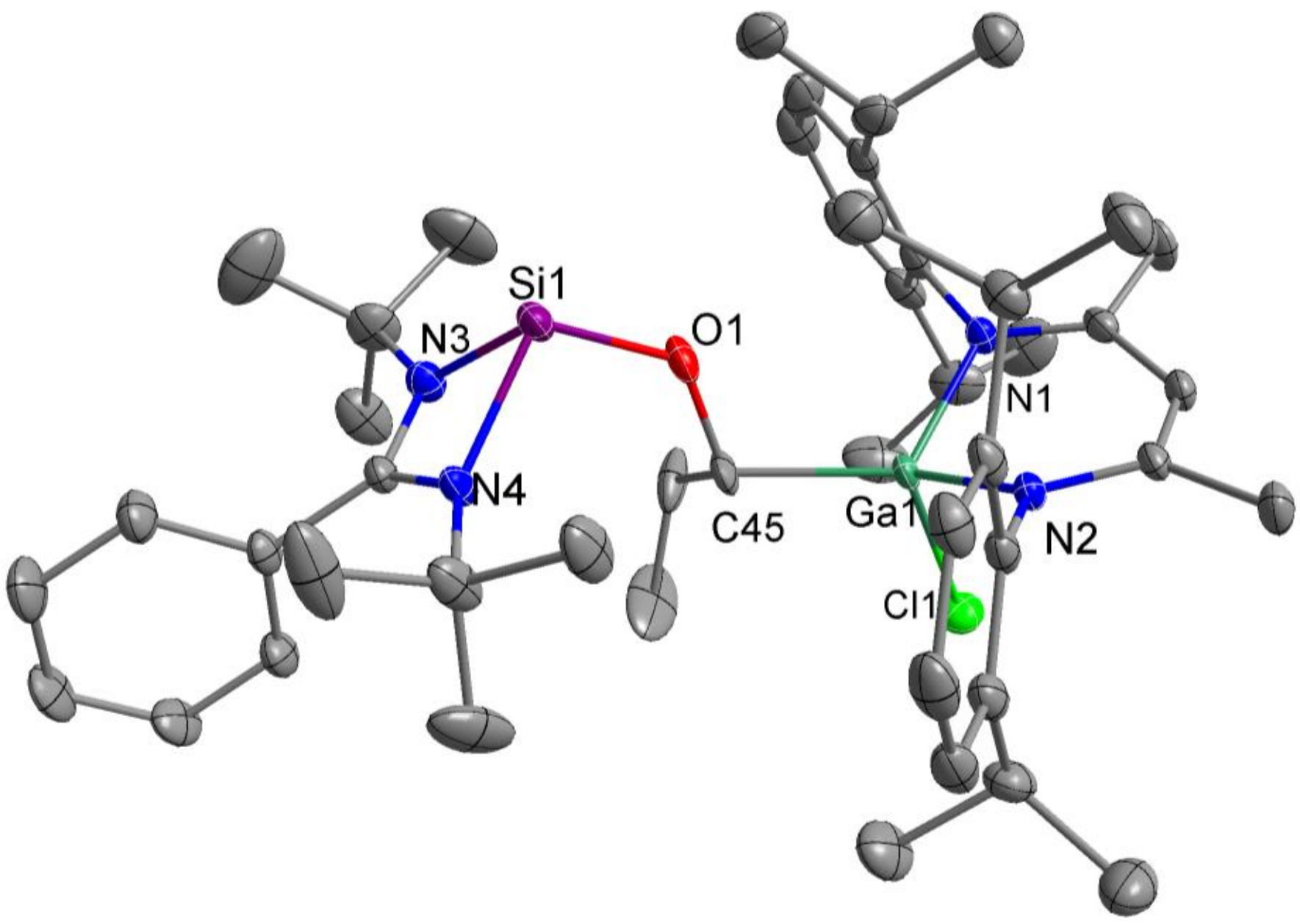

Figure S68. Molecular structure of **3** with thermal ellipsoids at 50% probability level. The hydrogen atoms and the disordered solvent molecule are omitted for clarity.

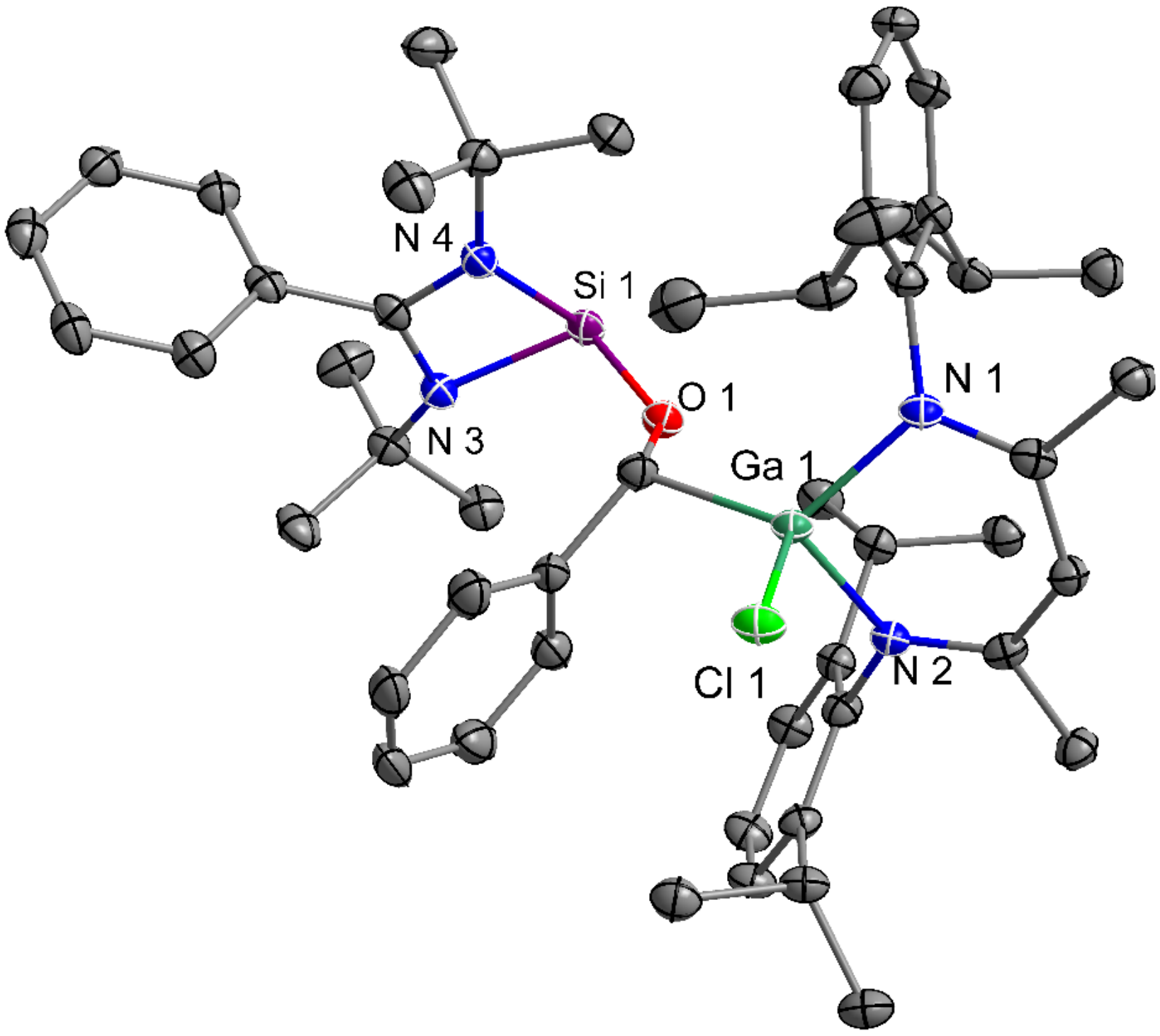

Figure S69. Molecular structure of **4** with thermal ellipsoids at 50% probability level. The hydrogen atoms and the disordered solvent molecule are omitted for clarity.

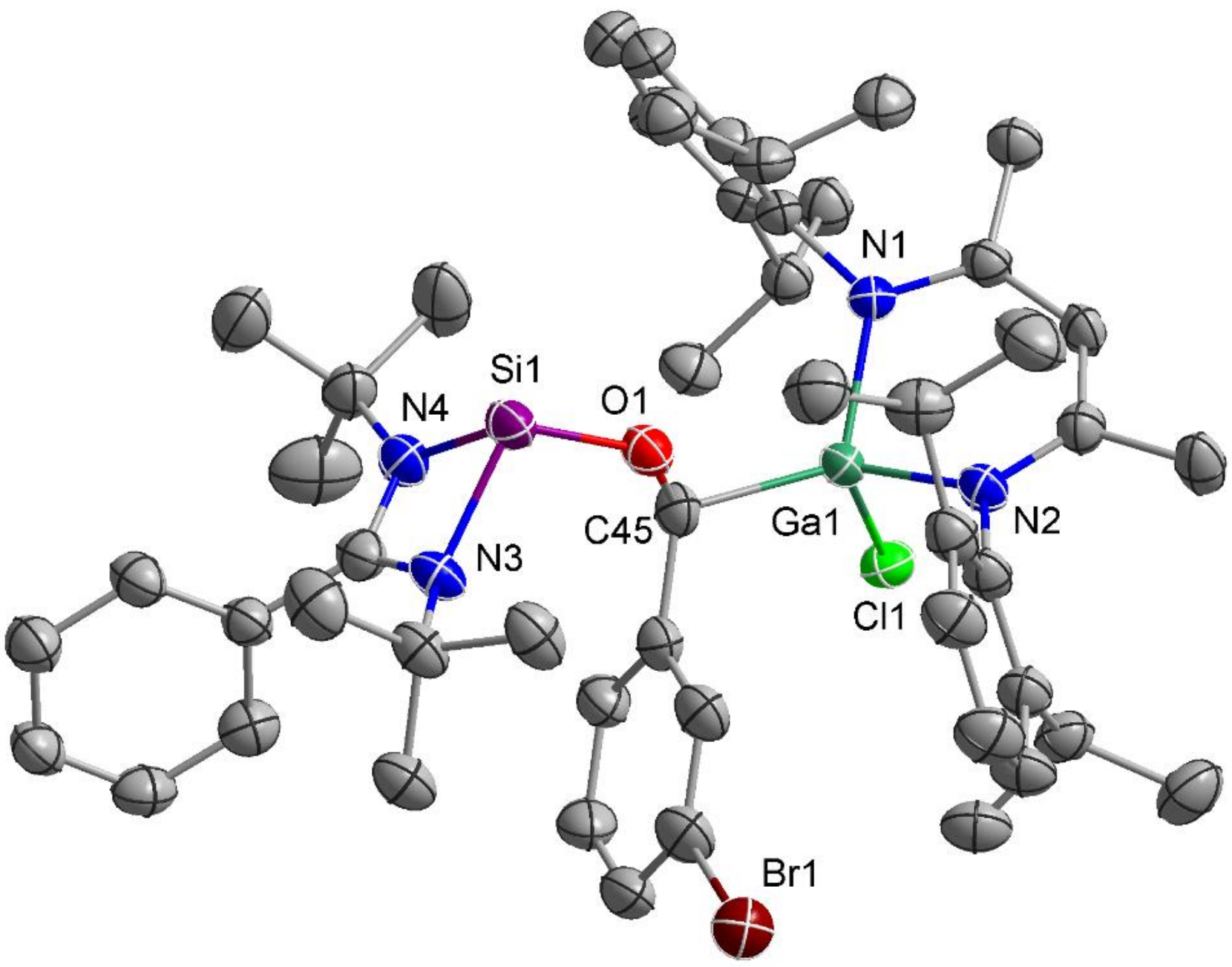

Figure S70. Molecular structure of **5** with thermal ellipsoids at 50% probability level. The hydrogen atoms and the disordered solvent molecule are omitted for clarity.

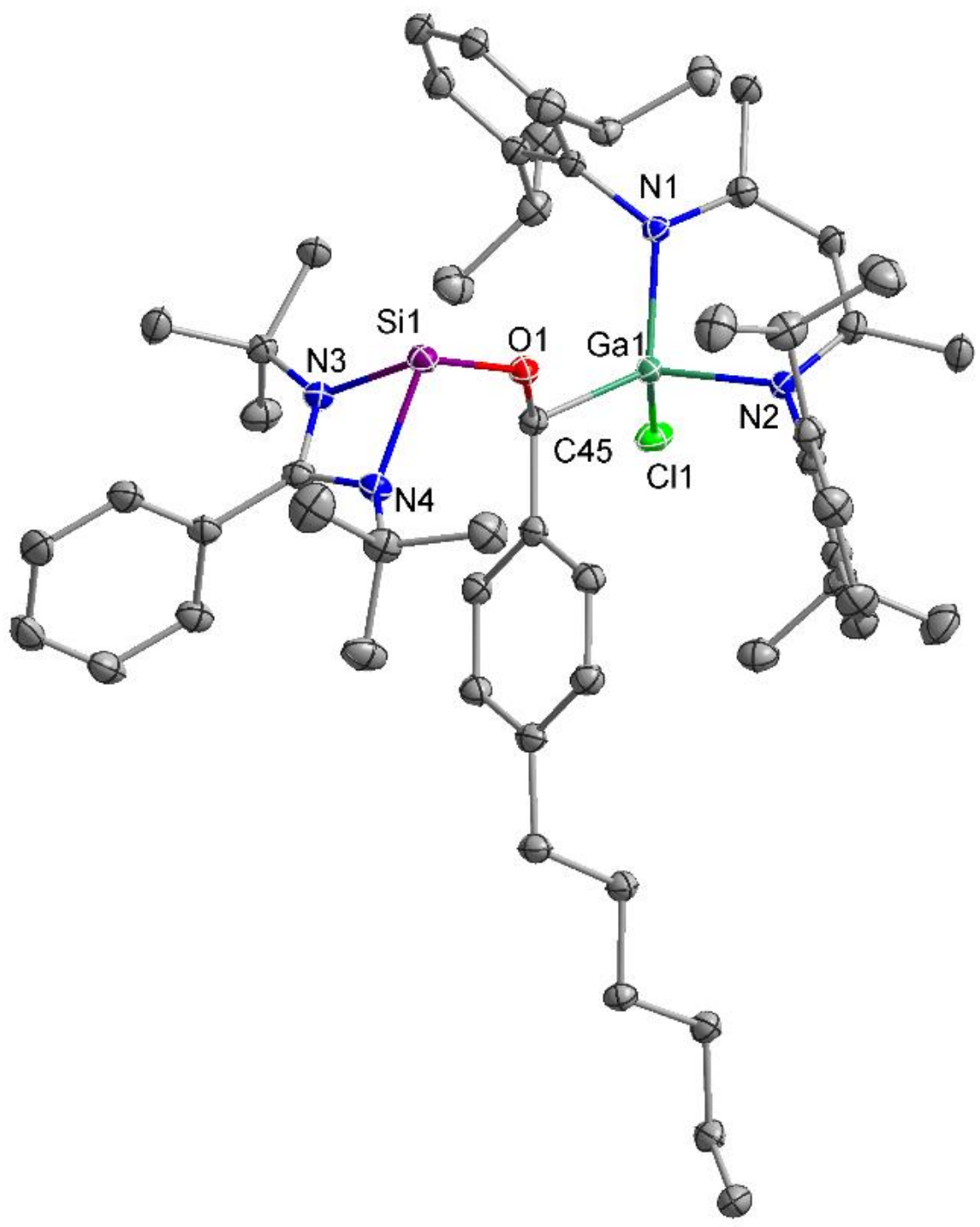

Figure S71. Molecular structure of **6** with thermal ellipsoids at 50% probability level. The hydrogen atoms and the disordered solvent molecule are omitted for clarity.

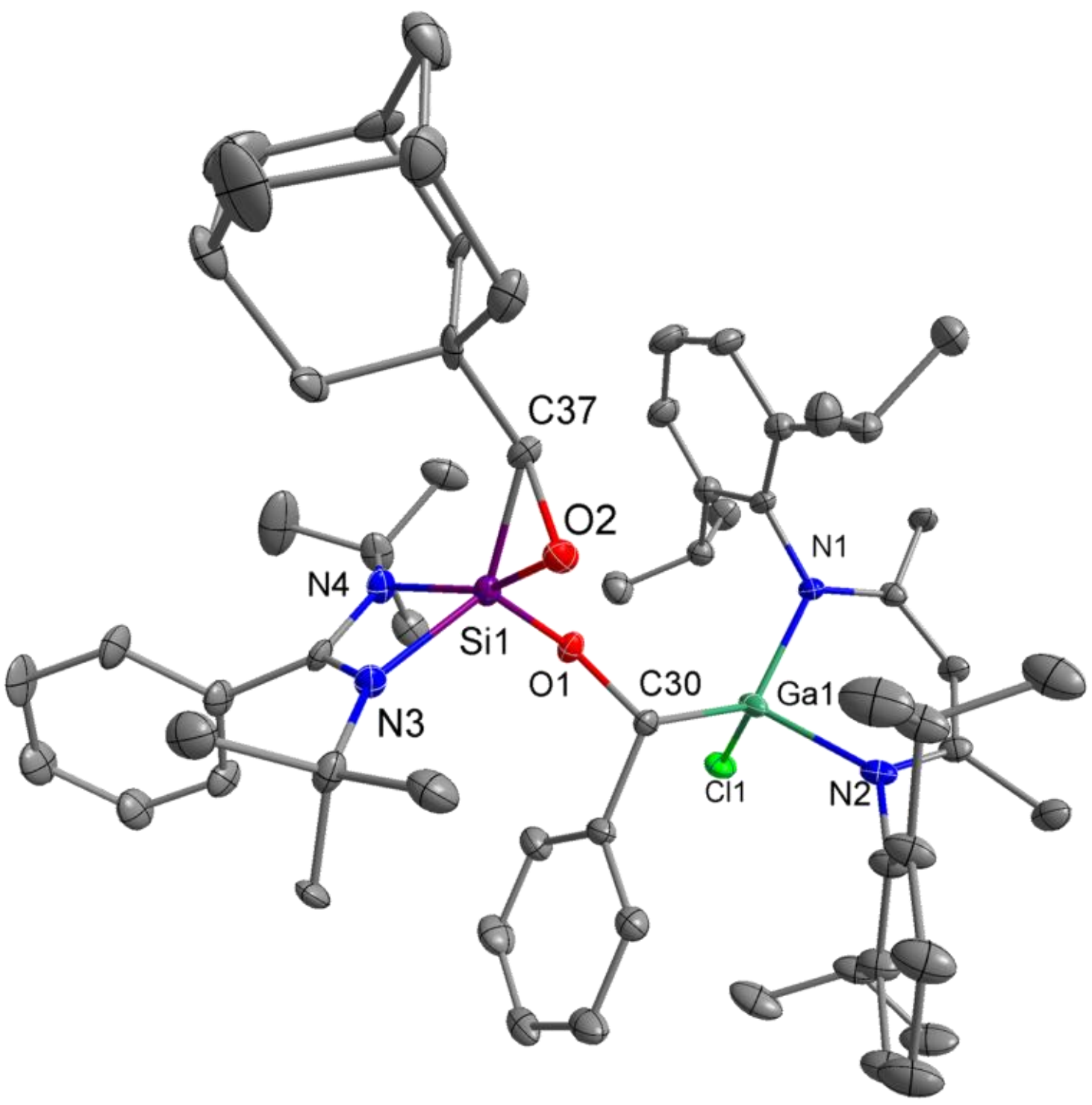

Figure S72. Molecular structure of **7** with thermal ellipsoids at 50% probability level. The hydrogen atoms and the disordered solvent molecule is omitted for clarity.

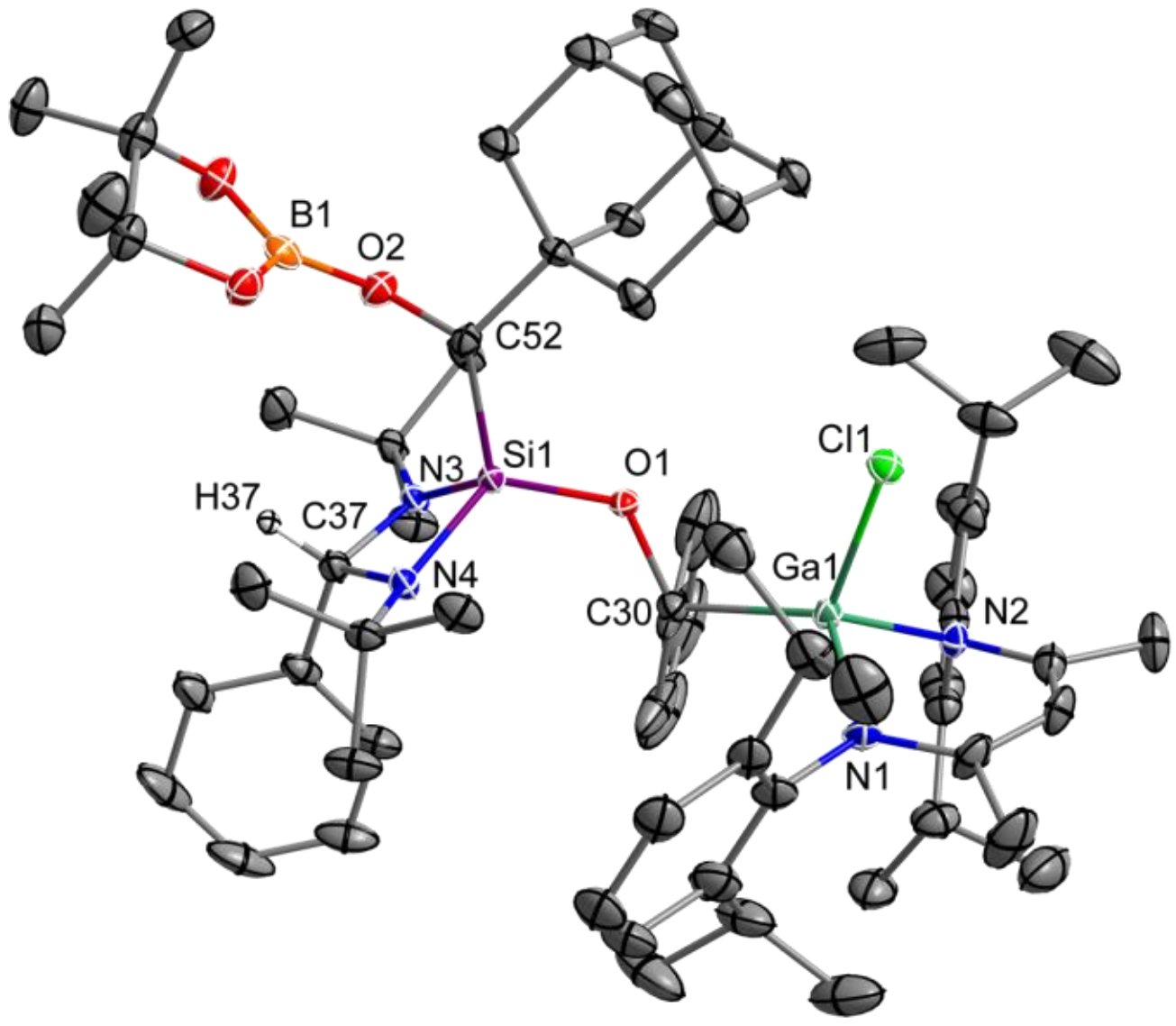

Figure S73. Molecular structure of **8** with thermal ellipsoids at 50% probability level. The hydrogen atoms and the disordered solvent molecules are omitted for clarity.

IV. Quantum chemical calculations

The calculation of the HOMO/LUMO gaps were performed by using the program package Orca 5.0[10]. The geometrical parameters were optimized by means of the density functional methods PBE0[11] with dispersion correction D4[12]. The basis sets def2-TZVPP[13], def2-QZVP[13] (for all atoms E>Ne) and the auxiliary basis set def2/J[14].

All calculations of the mechanistic pathways were performed by using the program packages Gaussian 16[15]. The geometrical parameters of the stationary points were optimized by means of the density functional methods PBE0 with the empirical dispersion D3BJ[16]. The basis sets def2-SVP[13] were employed. For all stationary points no symmetry restriction was applied. Frequency calculations were carried out at each of the structures to verify the nature of the stationary point. It turned out that all transition states have exactly one imaginary frequency, whereas all other structures have none. Furthermore, the energies of the stationary points were calculated using the density functionals PBE0-D3BJ and the basis sets def2-TZVP[13]. To take solvent effects into account, the solvent model SMD[17] (benzene as solvent) was used for the single point calculations. A well-known problem during the calculation of the Gibbs energies is the overestimation of the calculated entropies. The entropies are always computed on the assumption of an ideal gas. This leads to a large deviation for the translation and conformational term of the entropy if the reaction takes place in solution.[18,19] This is particularly dramatic for bi- and trimolecular reactions. Some authors have estimated that the total entropy in solution should be about 50-70% of that in the gas phase.[19-22] In our case, we have therefore used the upper limit, i.e. 70% (instead of 100%) of the calculated entropy. The thus obtained the Gibbs energies G70% were used for comparison with experimental data.

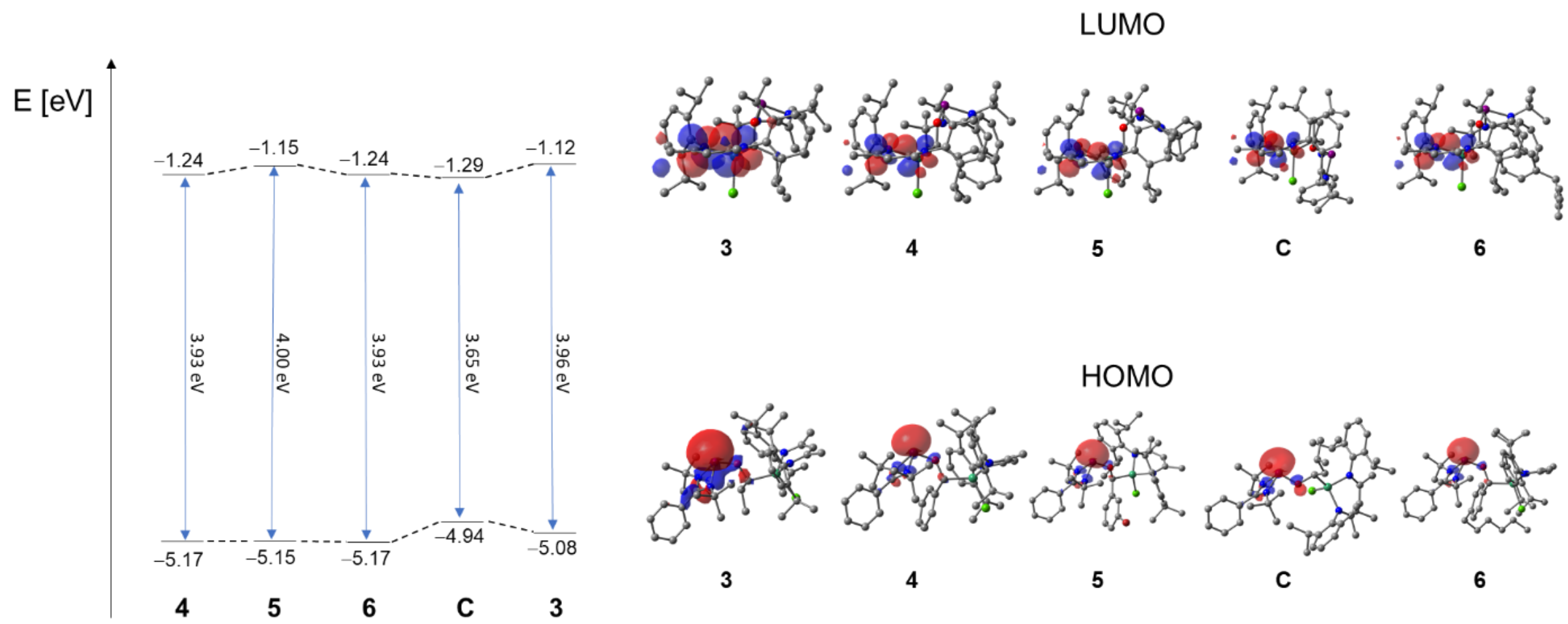

Figure S74. HOMO-LUMO gaps of compounds **3-6** and **C** calculated using PBE0-D4/def2-TZVPP,def2-QZVP(for all atoms E>Ne).

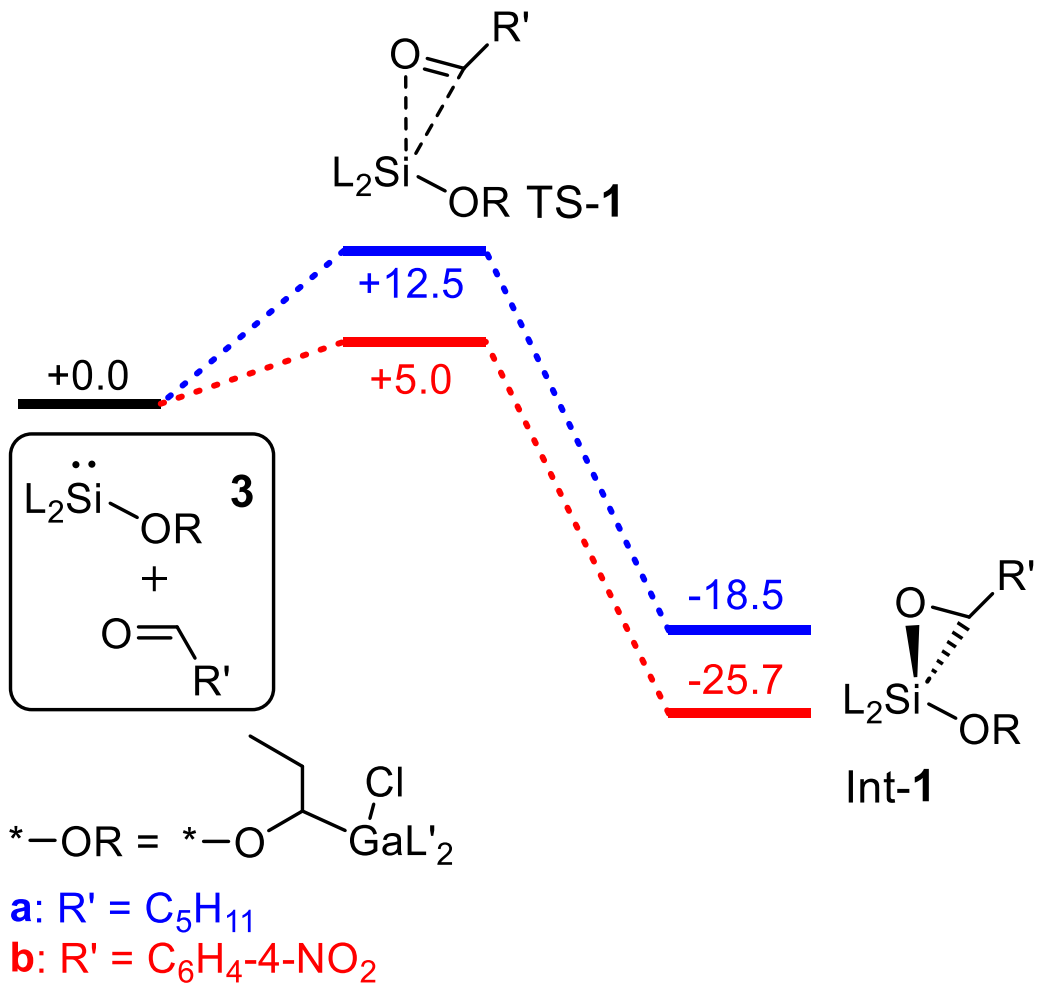

Figure S75. Gibbs energies ($G_{70\%}$) for the reaction of alkoxysilylene **3** with aldehydes to the corresponding oxasiliranes Int-**1** calculated by means of PBE0-D3BJ(SMD,benzene as solvent)/def2-TZVP//PBE0-D3BJ/def2-SVP. Herein $G_{70\%}$ means that 70% calculated gas-phase entropy contributes to solution-phase free energy. The values are given in kcal/mol. L_2 = PhC(N^{t}Bu)$_2$. L'$_2$ = HC[C(Me)NDipp]$_2$, Dipp = 2,6-^{i}Pr$_2$C$_6$H$_3$.

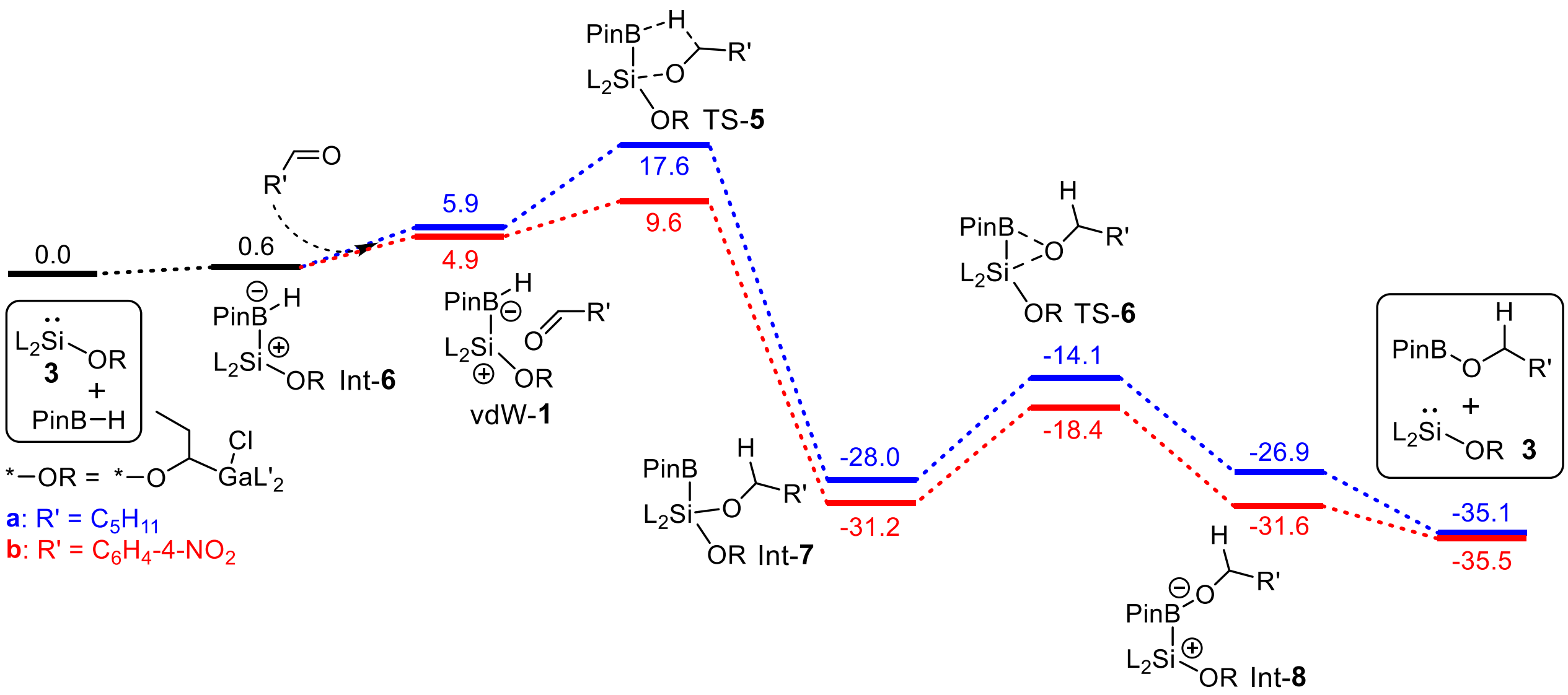

Figure S76. Gibbs energies ($G_{70\%}$) for the hydroboration of *n*-hexanal and *para*-nitrobenzaldehyde with **3** as catalytically active species calculated by means of PBE0-D3BJ(SMD,benzene as solvent)/def2-TZVP//PBE0-D3BJ/def2-SVP. Herein $G_{70\%}$ means that 70% calculated gas-phase entropy contributes to solution-phase free energy. The values are given in kcal/mol. L_2 = PhC(N^{t}Bu)$_2$. L'$_2$ = HC[C(Me)NDipp]$_2$, Dipp = 2,6-^{i}Pr$_2$C$_6$H$_3$.

Table S5. Absolute energies [au] of the calculated compounds by means of different methods.

Compound	E^a	G^a	E^b
PentCHO	-310.498865	-310.362947	-310.846640
NO_2PhCHO	-549.078795	-548.999708	-549.703367
HBPin	-411.105949	-410.948011	-411.561149
**3**	-4797.286449	-4796.298766	-4800.041229
TS-**1a**	-5107.791210	-5106.642254	-5110.886160
Int-**1a**	-5107.842424	-5106.688539	-5110.939724
Int-**2a**	-5518.971655	-5517.631991	-5522.513129
TS-**2a**	-5518.956042	-5517.620154	-5522.500127
Int-**3a**	-5519.011894	-5517.676109	-5522.549595
TS-**3a**	-5829.509608	-5828.011059	-5833.379392
Int-**4a**	-5829.561734	-5828.059316	-5833.430281
TS-**4a**	-5829.482846	-5827.988502	-5833.357766
Int-**5a**	-5829.565134	-5828.066698	-5833.435425
Int-**6**	-5208.415676	-5207.247691	-5211.617456
vdW-**1a**	-5518.938884	-5517.610536	-5522.473644
TS-**5a**	-5518.926859	-5517.592797	-5522.458742
Int-**7a**	-5518.998491	-5517.661147	-5522.535400
TS-**6a**	-5518.978164	-5517.641165	-5522.512201
Int-**8a**	-5518.999884	-5517.664983	-5522.531674
$C_5H_{11}CH_2$OBPin	-721.691495	-721.371159	-722.484064
**3**	-4797.286449	-4796.298766	-4800.041229
TS-**1b**	-5346.380765	-5345.288682	-5349.754722
Int-**1b**	-5346.434975	-5345.336345	-5349.808923
Int-**2b**	-5757.550940	-5756.270981	-5761.371918
TS-**2b**	-5757.543272	-5756.259458	-5761.356971
Int-**3b**	-5757.602591	-5756.321519	-5761.415320
TS-**3b**	-6306.670403	-6305.283447	-6311.089958
Int-**4b**	-6306.719249	-6305.330621	-6311.143792
TS-**4b**	-6306.655496	-6305.277183	-6311.084641
Int-**5b**	-6306.749046	-6305.360408	-6311.166352
Int-**6**	-5208.415676	-5207.247691	-5211.617456
vdW-**1b**	-5757.520111	-5756.248038	-5761.332444
TS-**5b**	-5757.511419	-5756.236806	-5761.325994
Int-**7b**	-5757.580009	-5756.301385	-5761.395604
TS-**6b**	-5757.560369	-5756.282547	-5761.374018
Int-**8b**	-5757.582500	-5756.305492	-5761.394967
4-$NO_2C_6H_4CH_2$OBPin	-960.267771	-960.006366	-961.339906

[a] PBE0-D3BJ/def2-SVP.
[b] PBE0-D3BJ(benzene as solvent)/def2-TZVP//PBE0-D3BJ/def2-SVP.

Cartesian coordinates of the optimized geometry of HBPin at PBE0-D3BJ/def2-SVP level of theory:

C	0.78343000	-0.18432800	0.05278800
C	-0.78345900	-0.18424500	-0.05281800
O	-1.06701400	1.18549000	-0.39617600
O	1.06710600	1.18545400	0.39610400
B	0.00006900	1.93122300	-0.00002800
H	0.00008200	3.13266800	-0.00010600
C	-1.47055400	-0.45411300	1.28063500
H	-2.54036000	-0.22220700	1.17979700
H	-1.36918300	-1.50514600	1.58664400
H	-1.05941600	0.18399400	2.07634000
C	-1.35088800	-1.09010400	-1.12866400
H	-1.08066800	-2.13967400	-0.93796800
H	-2.44780400	-1.01494500	-1.13268700
H	-0.99010700	-0.80833300	-2.12581200
C	1.35074600	-1.09010000	1.12873800
H	1.08043700	-2.13965900	0.93810400
H	2.44766800	-1.01503900	1.13279900
H	0.98993800	-0.80822900	2.12584800
C	1.47059100	-0.45429000	-1.28060700

H	2.54040900	-0.22246200	-1.17972800
H	1.36916800	-1.50533600	-1.58655400
H	1.05955000	0.18378600	-2.07638500

Cartesian coordinates of the optimized geometry of PentCHO at PBE0-D3BJ/def2-SVP level of theory:

O	3.68184000	-0.00545500	-0.42435300
C	2.71387600	0.21883600	0.25046000
H	2.72641300	1.04936000	1.00912000
C	1.41700000	-0.53633600	0.18259100
H	1.32877800	-1.10993800	1.12502100
H	1.48364200	-1.26307800	-0.64171700
C	0.20613100	0.38114500	0.04091300
H	0.29036600	0.95401300	-0.89931700
H	0.21546700	1.13103000	0.85261900
C	-1.11934900	-0.36591400	0.06057900
H	-1.20330000	-0.93456000	1.00483100
H	-1.12341000	-1.12291700	-0.74431600
C	-2.33221700	0.54126300	-0.09323000
H	-2.24194300	1.10808000	-1.03675200
H	-2.32228400	1.29821700	0.71105600
C	-3.65317100	-0.21061300	-0.07234400
H	-3.78692100	-0.75787100	0.87469100
H	-4.50993000	0.47048900	-0.18608100
H	-3.70522200	-0.94946900	-0.88814900

Cartesian coordinates of the optimized geometry of NO_2PhCHO at PBE0-D3BJ/def2-SVP level of theory:

O	3.92419800	-0.61231900	-0.00010000
C	3.17930000	0.33257400	0.00004300
H	3.56975000	1.38198800	0.00020500
C	1.69850800	0.22249400	0.00002600
C	1.09892200	-1.04341900	0.00003200
C	0.91058600	1.37759900	0.00000900
C	-0.28371200	-1.15711100	0.00002300
H	1.74409300	-1.92466700	0.00005200
C	-0.47648400	1.27888000	0.00000300
H	1.38935500	2.36078100	0.00001100
C	-1.04511100	0.01003700	0.00000500
H	-0.79084200	-2.12198000	0.00002800
H	-1.12623200	2.15387000	-0.00001000
N	-2.51440100	-0.10398300	-0.00001400
O	-3.15042500	0.92515200	-0.00004500
O	-2.98344600	-1.21888800	0.00001500

Cartesian coordinates of the optimized geometry of $C_5H_{11}CH_2$OBPin at PBE0-D3BJ/def2-SVP level of theory:

O	0.07347400	-1.43009400	-1.12199700
C	0.81718900	-2.27042600	-0.26141200
H	1.30492000	-3.02307400	-0.90200600
C	1.85869800	-1.49789700	0.52670400
H	2.38145100	-2.20849800	1.19118500
H	1.33679400	-0.78669400	1.19052600
C	2.86594800	-0.75872000	-0.34107800
H	2.32263300	-0.10164000	-1.04127600
H	3.40775500	-1.48567400	-0.97338400
H	0.14016500	-2.79999800	0.42983900
C	-2.58902200	-0.00408000	0.75036300
C	-2.49313700	0.92564000	-0.51241600
B	-0.94961300	-0.70282600	-0.62933600
O	-1.34730400	-0.72184700	0.68637000
O	-1.69554700	0.14012600	-1.40860300
C	-1.72179700	2.21132200	-0.23470200

H	-1.49361500	2.69870300	-1.19321900
H	-2.29909400	2.91222500	0.38512200
H	-0.76891800	1.99845800	0.27239300
C	-3.82105400	1.23865700	-1.17400200
H	-4.49149700	1.76476600	-0.47772200
H	-3.65461600	1.88839100	-2.04530300
H	-4.31971600	0.32645300	-1.52511600
C	-2.67121800	0.72630100	2.07652900
H	-3.56877700	1.36157200	2.11843400
H	-2.73071200	-0.00302400	2.89744300
H	-1.78656300	1.35315700	2.24575600
C	-3.70874300	-1.03213000	0.63915300
H	-3.57789900	-1.78440200	1.43023300
H	-4.70045700	-0.57246700	0.75685300
H	-3.67570300	-1.54860000	-0.33147800
C	3.86574000	0.06355300	0.45782400
H	4.40649300	-0.59487700	1.16273600
H	3.31887200	0.78745100	1.08992400
C	4.87275100	0.81119400	-0.40508000
H	5.41834400	0.08565000	-1.03428200
H	4.32809400	1.46393900	-1.11011100
C	5.86353500	1.63813700	0.39826800
H	6.44669600	1.00530600	1.08679400
H	6.57723900	2.16544900	-0.25288900
H	5.34786000	2.39677000	1.00916400

Cartesian coordinates of the optimized geometry of 4-$NO_2C_6H_4CH_2OBPin$ at PBE0-D3BJ/def2-SVP level of theory:

O	0.97806900	1.84857000	-1.18142000
C	-0.04925600	2.40929500	-0.40973600
H	-0.36731300	3.33717000	-0.91472100
H	0.31396300	2.68674000	0.59444500
C	2.81844800	-0.30325600	0.97233000
C	3.15095600	-0.84938200	-0.46316800
B	1.78010500	0.91635100	-0.60723800
O	1.69570700	0.55765400	0.71069300
O	2.73286400	0.23681200	-1.30812200
C	2.30061400	-2.05710400	-0.84052400
H	2.41526400	-2.24618800	-1.91731400
H	2.60523300	-2.96074500	-0.29379900
H	1.23508900	-1.86982000	-0.63926500
C	4.61858700	-1.13424500	-0.71173700
H	4.99005000	-1.90297700	-0.01758000
H	4.75337900	-1.50627100	-1.73751000
H	5.22926300	-0.22967900	-0.59833800
C	2.40439300	-1.36026500	1.97681700
H	3.21000900	-2.09479300	2.12560100
H	2.19165100	-0.88635100	2.94595100
H	1.49850400	-1.88854400	1.65404500
C	3.92896100	0.57018600	1.54236500
H	3.54648600	1.09554300	2.42919100
H	4.80293700	-0.02530600	1.84216500
H	4.25476100	1.32532900	0.81188100
C	-1.23969600	1.49518300	-0.26617200
C	-2.16602600	1.71156700	0.76066700
C	-1.44582100	0.44111300	-1.16202200
C	-3.28120400	0.89527700	0.89672400
H	-2.00628900	2.52625200	1.47197500
C	-2.55389500	-0.38936100	-1.03581900
H	-0.72488900	0.27324800	-1.96440300
C	-3.45713600	-0.14855700	-0.00669000
H	-4.01361800	1.04024800	1.69080000
H	-2.73599000	-1.21896900	-1.71910400
N	-4.62619700	-1.02325000	0.13549900
O	-4.74575200	-1.92965500	-0.65903700
O	-5.39703600	-0.78295600	1.03856800

Cartesian coordinates of the optimized geometry of **3** at PBE0-D3BJ/def2-SVP level of theory:

Ga	1.39532100	-0.24659300	-0.44764700
Cl	0.48995600	-1.46110500	-2.09741100
Si	-2.11341600	-1.11484100	1.87112900
O	-0.84206400	-0.28914800	1.07789400
N	1.49182800	1.54685200	-1.25316500
N	3.31436600	-0.62283900	-0.66056100
N	-3.17612800	-1.74117700	0.39235400
N	-3.51098500	0.09837500	1.43637100
C	2.33374800	1.71701100	-2.27231600
C	3.38313400	0.83070000	-2.57687800
H	3.94136800	1.05431000	-3.48484000
C	3.89972900	-0.20504000	-1.78162400
C	2.16619200	2.91051500	-3.16937300
H	1.30168500	2.74938500	-3.83183000
H	3.05578500	3.06705500	-3.79055000
H	1.95583800	3.82213300	-2.59421200
C	5.15679700	-0.88000000	-2.25073800
H	5.80203200	-1.17706300	-1.41408700
H	5.72052900	-0.24505000	-2.94477800
H	4.87469100	-1.80426500	-2.78160000
C	0.55321800	2.57102700	-0.91339500
C	-0.68476700	2.65412800	-1.58150300
C	-1.56831600	3.67636500	-1.21890200
H	-2.53067300	3.75300200	-1.73071300
C	-1.24975600	4.58780900	-0.22243700
H	-1.95539000	5.37690100	0.04774000
C	-0.03263000	4.48239300	0.44190100
H	0.20606700	5.19342600	1.23412700
C	0.88570200	3.48079000	0.11692200
C	-1.10670800	1.65871500	-2.64233400
H	-0.24848300	1.00934000	-2.86618400
C	-1.54323800	2.33407800	-3.94175700
H	-2.46510500	2.92213300	-3.80711000
H	-1.74894800	1.57570800	-4.71286000
H	-0.77420200	3.01705100	-4.33439000
C	-2.21106400	0.75388100	-2.09951000
H	-1.90241800	0.25108300	-1.17238900
H	-2.46948400	-0.02010900	-2.83816300
H	-3.11842300	1.34186100	-1.88470800
C	2.21298800	3.38980400	0.84751900
H	2.46264100	2.31964900	0.90636500
C	3.34546200	4.07155300	0.07682800
H	3.11180800	5.13258300	-0.10593600
H	3.53582500	3.59040300	-0.89176800
H	4.28259000	4.02703100	0.65398400
C	2.15028500	3.92567900	2.27452200
H	1.30576200	3.49916200	2.83557800
H	2.05338400	5.02259600	2.29663700
H	3.07491900	3.67336900	2.81361500
C	4.00306400	-1.45649400	0.27156900
C	3.89060100	-2.85946100	0.20466900
C	4.55287200	-3.62615300	1.16955100
H	4.47083100	-4.71518700	1.13032400
C	5.30907900	-3.03194300	2.17044500
H	5.81897500	-3.64888700	2.91411300
C	5.41135800	-1.64585700	2.22517000
H	6.00498200	-1.18366600	3.01625200
C	4.76350800	-0.83538100	1.29026000
C	3.07620300	-3.55548400	-0.86601200
H	2.70566200	-2.79004300	-1.56243300
C	3.91580300	-4.54851700	-1.66902900
H	4.26029000	-5.39042700	-1.04750900
H	4.80857500	-4.07075500	-2.10067600
H	3.32195300	-4.96994200	-2.49480900
C	1.84717500	-4.23449000	-0.26283800

H	2.13370600	-5.04452000	0.42725700
H	1.21528600	-4.66375800	-1.05485600
H	1.22979200	-3.51716600	0.29594800
C	4.90785000	0.67396500	1.35492300
H	4.02562100	1.09710700	0.85082600
C	6.13936300	1.15508300	0.58413000
H	7.05788200	0.71065000	1.00008500
H	6.23229400	2.25052300	0.64903200
H	6.08904300	0.89072700	-0.48030500
C	4.93737500	1.21368900	2.78192800
H	4.07940300	0.86041700	3.37275800
H	4.91333400	2.31373300	2.76950800
H	5.85652500	0.92131700	3.31300700
C	-4.06603300	-0.77073700	0.59488600
C	-5.46637200	-0.72666300	0.09579000
C	-6.51511500	-1.08547200	0.94965800
H	-6.29090600	-1.41657300	1.96611200
C	-7.83291800	-1.03500500	0.50162300
H	-8.64542300	-1.32253400	1.17294400
C	-8.11340500	-0.61928100	-0.79946300
H	-9.14748400	-0.57597600	-1.14926900
C	-7.07107900	-0.26086000	-1.65327300
H	-7.28576800	0.06617700	-2.67323000
C	-5.75165000	-0.31751900	-1.21054300
H	-4.93373200	-0.03124000	-1.87357800
C	-3.29880000	-3.05413700	-0.23913900
C	-4.38710400	-3.89756600	0.43131100
H	-4.20091200	-3.96607300	1.51419500
H	-4.39141900	-4.91736100	0.01632300
H	-5.38653000	-3.46654400	0.27477800
C	-3.57103000	-2.91407500	-1.73851300
H	-4.55597100	-2.46893600	-1.93457500
H	-3.55089200	-3.90604300	-2.21501500
H	-2.79390500	-2.29192900	-2.20499500
C	-1.93822100	-3.72869200	-0.05643000
H	-1.69909400	-3.83987900	1.01337300
H	-1.14970000	-3.12922900	-0.53475500
H	-1.94095300	-4.72956200	-0.51285500
C	-3.93700700	1.38970900	1.96401300
C	-4.85492200	1.19466000	3.17541100
H	-4.36732800	0.54850000	3.92081000
H	-5.80621700	0.72902700	2.87955400
H	-5.08374300	2.16221200	3.64862600
C	-2.65835800	2.11013800	2.40022300
H	-2.89869600	3.10398000	2.80607800
H	-1.96938800	2.22594400	1.55196100
H	-2.13795100	1.53872700	3.18446900
C	-4.63863600	2.23363200	0.89905200
H	-4.82987200	3.24132800	1.29773800
H	-5.60271900	1.80359600	0.59614200
H	-3.99985700	2.33424900	0.00992100
C	0.53448600	-0.51958900	1.31220900
H	0.71213700	-1.57587100	1.60579300
C	1.08274600	0.37892700	2.41277400
H	0.77841400	1.41199400	2.18243500
H	2.18461100	0.35786400	2.36524800
C	0.64251900	0.01497400	3.82257500
H	-0.45464800	0.01080100	3.91777500
H	0.98993500	-0.99365900	4.09702500
H	1.04385500	0.72598000	4.56249700

Cartesian coordinates of the optimized geometry of vdW-**1a** at PBE0-D3BJ/def2-SVP level of theory:

Ga	-2.39883800	-0.45695900	-0.50765400
Cl	-2.04232800	-1.15880300	-2.60190100
Si	1.81808700	0.03904800	-0.23171300
O	0.29723200	-0.49910200	0.17754500

N	-3.36047900	-1.98759700	0.26213100
N	-3.99590400	0.66822500	-0.72079500
N	2.43837900	-0.95657700	-1.68052800
N	2.90953300	-1.32660400	0.38319200
C	-4.56361100	-2.26104200	-0.24097100
C	-5.34243400	-1.32232300	-0.93976500
H	-6.28816300	-1.69342700	-1.33227200
C	-5.12881600	0.06158200	-1.07266300
C	-5.12070400	-3.64876200	-0.09873700
H	-4.70574900	-4.27143100	-0.90848700
H	-6.21362700	-3.65507900	-0.18949600
H	-4.82520700	-4.11715300	0.84865600
C	-6.23966900	0.87887200	-1.66722600
H	-6.38021100	1.82512000	-1.12793400
H	-7.18308300	0.32069100	-1.68078400
H	-5.97517900	1.14437300	-2.70319100
C	-2.71081100	-2.87552200	1.17463500
C	-1.83265300	-3.87260900	0.70581100
C	-1.17308600	-4.67330700	1.64483100
H	-0.48532000	-5.44646900	1.29346800
C	-1.37408700	-4.50414300	3.00750100
H	-0.84706100	-5.13819800	3.72436900
C	-2.24910900	-3.52058200	3.45669800
H	-2.40244100	-3.39038600	4.52935200
C	-2.92710300	-2.69093400	2.56077800
C	-1.57319900	-4.09979700	-0.76890600
H	-2.22831100	-3.42535100	-1.33723000
C	-1.89650900	-5.53224800	-1.19206400
H	-1.22200000	-6.26159800	-0.71546600
H	-1.78397200	-5.64246200	-2.28184600
H	-2.92662900	-5.81433600	-0.92578900
C	-0.13632500	-3.72598800	-1.13078400
H	0.09770700	-2.69553700	-0.82771100
H	0.01846200	-3.80246100	-2.21813800
H	0.57716500	-4.40128300	-0.63015800
C	-3.88607900	-1.63213800	3.07248300
H	-3.89909200	-0.83203400	2.31729900
C	-5.31573800	-2.16660000	3.18385400
H	-5.36033300	-3.02092100	3.87831300
H	-5.71091200	-2.49804400	2.21482500
H	-5.99015700	-1.38362100	3.56502400
C	-3.45246600	-1.01645200	4.39916700
H	-2.41709100	-0.64846600	4.36161900
H	-3.52928100	-1.73534800	5.22976800
H	-4.10692800	-0.16910600	4.65243000
C	-3.90559500	2.09560000	-0.69770900
C	-3.53855300	2.81175200	-1.85541900
C	-3.45828400	4.20584700	-1.77789800
H	-3.17005300	4.77129400	-2.66723900
C	-3.72667100	4.88123800	-0.59547500
H	-3.65651600	5.97071900	-0.55633000
C	-4.06892800	4.16187800	0.54384800
H	-4.26445600	4.69518300	1.47588300
C	-4.15836600	2.76792700	0.51955000
C	-3.18196800	2.12342100	-3.15624400
H	-3.41186800	1.05374200	-3.05177900
C	-3.97758300	2.65945400	-4.34490300
H	-3.73302100	3.71121000	-4.56248100
H	-5.06313500	2.60341100	-4.17024500
H	-3.74889100	2.07580700	-5.24990100
C	-1.67780000	2.22803000	-3.40847500
H	-1.37480600	3.27263200	-3.58545800
H	-1.38662800	1.63008300	-4.28490700
H	-1.10536900	1.85426800	-2.54793300
C	-4.53413800	2.00624600	1.77539600
H	-4.11392300	0.99558900	1.66176100
C	-6.04814900	1.84062600	1.91553900
H	-6.54747300	2.82181000	1.96036400
H	-6.29231900	1.29332600	2.83992400

H	-6.47940700	1.27864700	1.07568200
C	-3.93911700	2.62088400	3.03914900
H	-2.85878400	2.79866200	2.93502100
H	-4.09136200	1.94941400	3.89677800
H	-4.41744300	3.57963600	3.29309500
C	3.19230100	-1.69725700	-0.86377100
C	4.15705300	-2.74133200	-1.28529000
C	5.51899600	-2.44282600	-1.39282400
H	5.86889000	-1.44074700	-1.13369700
C	6.40392500	-3.43019700	-1.82107500
H	7.46754700	-3.19699000	-1.90874900
C	5.94045800	-4.70814400	-2.13146500
H	6.64044100	-5.47848300	-2.46374300
C	4.58212700	-5.00266100	-2.01864600
H	4.21372000	-6.00142200	-2.26336900
C	3.68806800	-4.02032200	-1.60160500
H	2.62088400	-4.23774400	-1.53168000
C	2.60368300	-0.63232800	-3.10246500
C	3.81503500	0.28940600	-3.27018400
H	3.71449200	1.17345900	-2.62130800
H	3.89964100	0.62253200	-4.31622000
H	4.74511700	-0.23240500	-3.00029900
C	2.74598800	-1.88486200	-3.96611100
H	3.70421700	-2.39695500	-3.80987700
H	2.68976800	-1.59432400	-5.02574200
H	1.92880100	-2.59277900	-3.76258200
C	1.32328100	0.09784600	-3.49376000
H	1.18012100	0.98148600	-2.85552400
H	0.44320000	-0.55030900	-3.38025400
H	1.37981000	0.44880900	-4.53437800
C	3.21239500	-1.96680500	1.66770700
C	4.72112200	-2.12845200	1.86422400
H	5.24168200	-1.18677300	1.63920400
H	5.13363900	-2.91118600	1.21337700
H	4.92854000	-2.41535600	2.90614900
C	2.64877500	-1.04217300	2.74400100
H	2.87051100	-1.44348900	3.74304300
H	1.55787800	-0.95685900	2.64251500
H	3.08550300	-0.03543800	2.66722600
C	2.49922700	-3.31879400	1.75118400
H	2.65668700	-3.76754100	2.74389200
H	2.88836000	-4.02098500	0.99966400
H	1.41763100	-3.19498100	1.59425800
C	-0.85216500	0.30823900	0.45816500
H	-0.68568000	1.33235500	0.08006400
C	-1.09211400	0.37984900	1.96147300
H	-0.99292500	-0.64223100	2.36569700
H	-2.14122600	0.66581100	2.13655600
C	-0.19862500	1.35345800	2.71613100
H	0.86879000	1.15185700	2.55382800
H	-0.36678400	2.38345400	2.37095100
H	-0.39025300	1.30558900	3.80020200
C	1.41182800	4.12463200	-0.18364800
C	2.53870000	3.90250700	-1.25556600
B	2.28611500	2.03260900	0.14061500
O	1.04529300	2.79860600	0.13373800
O	3.16504400	2.70638400	-0.81850000
H	2.82786100	1.92017100	1.26275500
C	5.86148900	1.69476100	-0.21808900
O	5.84030300	0.48694400	-0.16981900
H	5.94387300	2.21672300	-1.20134500
C	5.76591500	2.59298000	0.97013400
H	6.59938300	3.31816000	0.91882400
H	4.85059300	3.18075000	0.77734800
C	5.68816600	1.88052300	2.30482900
H	6.64906500	1.38399300	2.52620700
H	4.94620300	1.07237100	2.21570400
C	3.58004100	5.01112800	-1.31686600
H	3.12025400	5.97614200	-1.58195500

H	4.10413700	5.12793700	-0.35915900
H	4.33049700	4.77162800	-2.08605200
C	1.94114400	4.81393600	1.07615100
H	2.17778000	5.87429200	0.90388400
H	1.17019700	4.75251900	1.85834500
H	2.84072800	4.30885300	1.45647400
C	0.18298800	4.86286900	-0.68838700
H	-0.54465800	4.97219500	0.12961800
H	0.44065400	5.86859500	-1.05561500
H	-0.31436100	4.30634100	-1.49260200
C	1.95174100	3.68770400	-2.64819500
H	1.56599700	4.62246600	-3.08223100
H	2.73240700	3.29487100	-3.31584100
H	1.12980700	2.96177700	-2.61412600
C	5.29133000	2.79571200	3.45174900
H	6.02766400	3.61422800	3.55529600
H	4.33098500	3.28165000	3.20097600
C	5.14979800	2.06890600	4.78167100
H	4.41832300	1.24987900	4.66319600
H	6.10759200	1.57907200	5.03291700
C	4.72070100	2.97558700	5.92361400
H	4.62315000	2.42240100	6.87027700
H	5.44925500	3.78652400	6.08584800
H	3.74766800	3.44729800	5.71146300

Cartesian coordinates of the optimized geometry of vdW-**1b** at PBE0-D3BJ/def2-SVP level of theory:

Ga	-2.76558900	-0.17839800	0.37985700
Cl	-2.44989400	0.68676700	2.41424600
Si	1.36451600	0.79260200	-0.00054700
O	-0.26523600	0.80623500	-0.35001600
N	-4.30379000	0.82345200	-0.31452700
N	-3.79732100	-1.80194400	0.82633200
N	1.62593500	1.90956200	1.45368500
N	1.93805400	2.46584400	-0.59365600
C	-5.48638600	0.65012600	0.28244600
C	-5.81196000	-0.48603500	1.03142000
H	-6.81605400	-0.50410300	1.45355500
C	-5.07553600	-1.67932100	1.16802200
C	-6.52631300	1.72804100	0.16770000
H	-6.26236700	2.53951800	0.86544900
H	-7.52281700	1.35585700	0.43349600
H	-6.55434700	2.17028100	-0.83640100
C	-5.81774200	-2.85203900	1.74484400
H	-5.37888500	-3.81034700	1.44095800
H	-6.87454300	-2.82133600	1.45122400
H	-5.77514500	-2.80245600	2.84398200
C	-4.15046900	1.80873600	-1.33713400
C	-3.66126600	3.09239400	-1.03093200
C	-3.47129300	3.99908500	-2.07991300
H	-3.08411900	4.99662000	-1.85768100
C	-3.77036600	3.65732900	-3.39148100
H	-3.61660400	4.37976400	-4.19649000
C	-4.26480100	2.38844200	-3.67821200
H	-4.49280700	2.12432100	-4.71253200
C	-4.45663800	1.44251000	-2.66957700
C	-3.34099900	3.51639400	0.38713200
H	-3.61666700	2.68991900	1.05750200
C	-4.14419300	4.74731600	0.80696600
H	-3.86157000	5.63702600	0.22173600
H	-3.96226800	4.97849700	1.86802800
H	-5.22593300	4.59561600	0.67206200
C	-1.84237500	3.74966100	0.56629000
H	-1.26053000	2.85820700	0.29054300
H	-1.62020600	3.98863200	1.61759300
H	-1.50055900	4.59209700	-0.05793800
C	-4.97611100	0.05610900	-3.00756000

H	-4.61809300	-0.61622300	-2.21264200
C	-6.50525100	-0.00304000	-2.98911100
H	-6.93196000	0.70252400	-3.72001000
H	-6.91773600	0.24226100	-2.00184700
H	-6.85378400	-1.01457100	-3.25060400
C	-4.44943400	-0.47171200	-4.33976600
H	-3.35493200	-0.39303100	-4.40897300
H	-4.88201000	0.07003400	-5.19528200
H	-4.72535800	-1.53028100	-4.46202100
C	-3.12418000	-3.05886000	0.92277000
C	-2.66097200	-3.53716400	2.16667000
C	-2.00691000	-4.77397700	2.19329300
H	-1.64213800	-5.15961000	3.14830800
C	-1.80482100	-5.51414600	1.03643800
H	-1.28858000	-6.47584100	1.08249800
C	-2.25391000	-5.02167600	-0.18421300
H	-2.08555400	-5.60233900	-1.09275400
C	-2.91473000	-3.79461100	-0.26528200
C	-2.83350800	-2.77073900	3.46453200
H	-3.41768200	-1.86366600	3.25102800
C	-3.57702700	-3.59092300	4.52021500
H	-2.97895900	-4.45345100	4.85423500
H	-4.53417600	-3.98311800	4.14643300
H	-3.78341300	-2.97339600	5.40800000
C	-1.48351300	-2.30456500	4.00703800
H	-0.83933300	-3.16001600	4.26613000
H	-1.62387600	-1.69498400	4.91282800
H	-0.95467800	-1.68706200	3.27172500
C	-3.45029700	-3.29169800	-1.59049000
H	-3.47701300	-2.19356500	-1.52198000
C	-4.89234100	-3.75194200	-1.81025200
H	-4.95077800	-4.85163100	-1.83795600
H	-5.28269700	-3.36638600	-2.76532800
H	-5.55901900	-3.39871400	-1.01073700
C	-2.56896800	-3.65442900	-2.78047700
H	-1.52120400	-3.36676200	-2.61233300
H	-2.92113900	-3.13543900	-3.68432000
H	-2.59422200	-4.73370400	-2.99771600
C	2.03921300	2.90161200	0.65659900
C	2.48369000	4.24334500	1.10876700
C	3.83875300	4.53185900	1.29520000
H	4.57924800	3.75723400	1.08549000
C	4.21779200	5.80354200	1.71662200
H	5.27687900	6.02910800	1.86079200
C	3.25608100	6.78729200	1.94635500
H	3.56039400	7.78426700	2.27368400
C	1.90604500	6.49812200	1.75510300
H	1.14851900	7.26524300	1.93065700
C	1.51689700	5.22661900	1.33944200
H	0.46189000	4.99475100	1.18615900
C	1.63736800	1.74900800	2.91186300
C	3.02561000	2.01655300	3.49591800
H	3.78297500	1.39089500	3.00107100
H	3.02789800	1.76860900	4.56780600
H	3.32008000	3.06962000	3.39421900
C	0.59650900	2.66407600	3.55875300
H	0.85028300	3.72423200	3.41710100
H	0.55253600	2.46999000	4.64116000
H	-0.39803100	2.46715000	3.13433000
C	1.26068500	0.28937200	3.15101800
H	1.98193200	-0.38371300	2.66176400
H	0.25444600	0.09299400	2.75178000
H	1.24181600	0.06804800	4.22798500
C	2.13841800	3.07845500	-1.90772800
C	3.00874900	4.33168100	-1.86807000
H	3.98922200	4.11563000	-1.42219800
H	2.53970900	5.15460200	-1.31271500
H	3.16883200	4.67290200	-2.90161700
C	2.84645700	2.03067900	-2.76968100

H	2.93909200	2.38901500	-3.80548300
H	2.29785200	1.07784800	-2.78632900
H	3.85015800	1.82793600	-2.37013700
C	0.75735500	3.41732000	-2.47899400
H	0.84560800	3.79285300	-3.50960400
H	0.27036400	4.19193000	-1.86786900
H	0.10111100	2.53609800	-2.47927700
C	-1.11822600	-0.26620800	-0.74597400
H	-0.60974700	-1.23008500	-0.56467000
C	-1.44181200	-0.16642700	-2.23022900
H	-1.88160300	0.82337100	-2.43203100
H	-2.23841900	-0.89533400	-2.44489400
C	-0.27788700	-0.42061600	-3.17342100
H	0.47903400	0.37174000	-3.09843900
H	0.23486800	-1.36469400	-2.94246300
H	-0.62045500	-0.43923100	-4.22007300
C	2.25990700	-3.08058200	-0.80109600
C	2.44693400	-2.90142100	0.74398600
B	2.61636500	-0.84984200	-0.34734200
O	1.90654600	-1.77428800	-1.20955900
O	3.02044200	-1.60593800	0.83430600
H	3.60058800	-0.27255400	-0.85969700
C	5.25236400	0.82637200	0.34328900
O	5.25591300	1.98139800	-0.02781100
H	4.56690800	0.47133200	1.14453000
C	3.38640200	-3.91159200	1.38348900
H	2.99369400	-4.93495300	1.27982100
H	4.38546700	-3.87934000	0.92993500
H	3.49006500	-3.69539000	2.45761900
C	3.55947600	-3.47613200	-1.50250800
H	3.85083500	-4.51696600	-1.29599900
H	3.41642500	-3.36417300	-2.58718100
H	4.38241900	-2.81164900	-1.20393400
C	1.14799300	-4.03835800	-1.19245500
H	1.08217200	-4.10230000	-2.28925400
H	1.33803200	-5.05131500	-0.80453500
H	0.17597400	-3.70142400	-0.81085600
C	1.11490300	-2.91186400	1.48845600
H	0.63047900	-3.89793900	1.47380400
H	1.28506800	-2.63180600	2.53716000
H	0.41169800	-2.18534100	1.05553200
C	6.19781000	-0.19848300	-0.15488000
C	7.13961900	0.13017300	-1.13758300
C	6.15798400	-1.48571100	0.39079200
C	8.03896500	-0.82728100	-1.58314800
H	7.14518600	1.14615600	-1.53776100
C	7.05490700	-2.45274900	-0.04677000
H	5.38028800	-1.72295100	1.12065500
C	7.97983200	-2.10407500	-1.02585100
H	8.78608300	-0.61688800	-2.34822500
H	7.04979200	-3.47089900	0.34252200
N	8.92983200	-3.12333800	-1.49472600
O	8.86255500	-4.22281900	-0.99169100
O	9.71911100	-2.79756100	-2.35361500

Cartesian coordinates of the optimized geometry of Int-**1a** at PBE0-D3BJ/def2-SVP level of theory:

Ga	-1.04742600	-0.82535800	-0.75160900
Cl	0.12254700	-0.92170500	-2.65578500
Si	1.94296100	1.66774600	0.38058800
O	0.95301600	0.41953300	0.90524800
N	-1.43726500	-2.72960900	-0.44193800
N	-2.87530900	-0.38425400	-1.36088900
N	3.25584100	1.26907800	-0.85768400
N	3.48335800	0.91587800	1.25100500
C	-2.20377100	-3.32676900	-1.36029500
C	-3.07866100	-2.63246600	-2.20704600

H	-3.61224600	-3.23827700	-2.93903300
C	-3.49686100	-1.28981900	-2.10867300
C	-2.11696400	-4.81804200	-1.52129900
H	-1.15776900	-5.06899300	-2.00202300
H	-2.93179100	-5.20105100	-2.14679100
H	-2.12610800	-5.33461200	-0.55232700
C	-4.70726600	-0.91629600	-2.91888100
H	-4.49499000	-1.12238000	-3.97909400
H	-4.97204900	0.14191000	-2.81270400
H	-5.56953000	-1.53649400	-2.63688700
C	-0.77520700	-3.50869200	0.55652200
C	0.52950800	-3.99404300	0.33615500
C	1.14603200	-4.74225300	1.34432900
H	2.15952400	-5.11767100	1.18364200
C	0.49487600	-5.01724600	2.53805200
H	0.98964000	-5.60746100	3.31299100
C	-0.79029600	-4.52916300	2.74617200
H	-1.29306300	-4.74019200	3.69122800
C	-1.44487500	-3.76539500	1.77621800
C	1.29275000	-3.70398600	-0.93782900
H	0.60042700	-3.24203400	-1.65476500
C	1.85777600	-4.96554700	-1.58748200
H	2.63637000	-5.43733700	-0.96699800
H	2.31600100	-4.71931300	-2.55779300
H	1.07495600	-5.71940700	-1.76334100
C	2.39576900	-2.68206400	-0.66786800
H	1.99606300	-1.75528800	-0.22931000
H	2.90966300	-2.41591800	-1.60356700
H	3.13758100	-3.09460100	0.03587600
C	-2.84220200	-3.23554600	2.04521900
H	-2.94698500	-2.31352700	1.45369600
C	-3.93110700	-4.19777200	1.56609300
H	-3.83620000	-5.17812800	2.06013600
H	-3.89187100	-4.35794900	0.48033300
H	-4.92904400	-3.79529100	1.80284300
C	-3.05785300	-2.88083100	3.51400400
H	-2.26443200	-2.21949900	3.89216300
H	-3.08432700	-3.77763400	4.15228800
H	-4.02015500	-2.36777900	3.64635400
C	-3.47019800	0.87423700	-1.03742400
C	-2.94592100	2.06591900	-1.59817700
C	-3.50535500	3.28232400	-1.19940200
H	-3.10744900	4.20936400	-1.61158700
C	-4.55485700	3.34073700	-0.28883400
H	-4.97378000	4.30605600	0.00476500
C	-5.06066000	2.16690800	0.24712600
H	-5.88362400	2.21384700	0.96430700
C	-4.52997500	0.92041500	-0.10326900
C	-1.82826100	2.04126400	-2.62557800
H	-1.04335500	1.37004700	-2.24239900
C	-2.29535500	1.46697800	-3.96414800
H	-3.12086100	2.06693400	-4.38055900
H	-2.62715800	0.42579900	-3.87797500
H	-1.46417400	1.47796600	-4.68485000
C	-1.16201000	3.39308900	-2.84310900
H	-1.85326400	4.12837600	-3.28619500
H	-0.32422900	3.27330600	-3.54444800
H	-0.74463100	3.78494400	-1.90603000
C	-5.12750100	-0.31723400	0.54275900
H	-4.52385800	-1.18643200	0.24011700
C	-6.56713700	-0.55579400	0.08031800
H	-7.22959400	0.25119500	0.43175800
H	-6.95307100	-1.50392600	0.48699000
H	-6.65064100	-0.59405100	-1.01357300
C	-5.08811800	-0.23238800	2.06835700
H	-4.06848000	-0.07341000	2.44562700
H	-5.47946600	-1.15991500	2.51294000
H	-5.71146000	0.59508500	2.44020400
C	4.09744500	0.81815200	0.09332000

C	5.48369100	0.32186500	-0.10261900
C	6.56487800	1.19755300	0.03785200
H	6.38075600	2.25191300	0.25402400
C	7.86792800	0.72346300	-0.08391400
H	8.70763800	1.41317600	0.02718000
C	8.10037000	-0.62739800	-0.34057200
H	9.12361200	-0.99861200	-0.43310200
C	7.02427400	-1.50259900	-0.47766200
H	7.20007000	-2.56184900	-0.67820100
C	5.71845700	-1.03152900	-0.35999500
H	4.87446400	-1.71364600	-0.46786100
C	3.53062200	1.54427100	-2.27993000
C	4.64653200	2.58440500	-2.42069300
H	4.40638000	3.48424800	-1.83379000
H	4.74767700	2.88268000	-3.47505400
H	5.61966800	2.19561000	-2.09191500
C	3.89992200	0.25147900	-3.01207600
H	4.83301600	-0.18849900	-2.63511900
H	4.03856900	0.46103000	-4.08344500
H	3.08544200	-0.48008600	-2.90982300
C	2.26090300	2.10644000	-2.91248000
H	1.95314000	3.04339900	-2.43432400
H	1.43105600	1.39354000	-2.81822000
H	2.44568100	2.28326600	-3.98250100
C	3.77716600	0.39986600	2.58456800
C	5.24920700	0.53608900	2.97718500
H	5.59943900	1.57052800	2.84360400
H	5.90336100	-0.13033900	2.40008000
H	5.36004700	0.27412400	4.04021000
C	2.93131000	1.23561100	3.54645100
H	3.05487500	0.87685900	4.57860900
H	1.87021200	1.16070300	3.27401100
H	3.22311000	2.29535900	3.50241100
C	3.34382600	-1.06812000	2.65718900
H	3.45914400	-1.45537700	3.68124400
H	3.96033100	-1.68643200	1.98773200
H	2.29329200	-1.16543400	2.35066500
C	-0.45331500	0.34737500	0.73370800
H	-0.84358900	1.34821500	0.47173600
C	-1.11868400	-0.13344100	2.01591200
H	-0.64670300	-1.08230600	2.32251100
H	-2.16391300	-0.38392900	1.76983900
C	-1.12553300	0.84627500	3.17800800
H	-0.10695000	1.11903100	3.48722300
H	-1.65490500	1.77384700	2.90962300
H	-1.63087800	0.41512700	4.05676200
C	1.56697900	3.42340600	0.70796600
O	0.86109100	2.74792800	-0.39821300
H	2.23104900	4.22103000	0.32605100
C	0.62359900	3.94251100	1.76734900
H	0.10629100	3.09980400	2.25205000
H	1.20746000	4.43944100	2.56279500
C	-0.42247400	4.89177100	1.19328100
H	0.06190900	5.83355500	0.87662900
H	-0.82594700	4.43034700	0.27647400
C	-1.57507800	5.19038800	2.13891700
H	-1.19054200	5.59383100	3.09426700
H	-2.07845300	4.24010300	2.39657400
C	-2.60269200	6.15219900	1.55914700
H	-2.91586300	5.77890100	0.56860500
H	-2.12226000	7.12870500	1.37066900
C	-3.82551200	6.34018900	2.44204300
H	-4.54488700	7.04816900	2.00188900
H	-4.35066700	5.38342400	2.59771700
H	-3.54722000	6.72512100	3.43661000

Cartesian coordinates of the optimized geometry of Int-**1b** at PBE0-D3BJ/def2-SVP level of theory:

Ga	0.89259400	-1.24534400	0.69055300
Cl	-0.45533200	-1.88113400	2.35886900
Si	-1.98271100	1.56497700	0.25669800
O	-1.09939800	0.41456500	-0.55711800
N	1.41932400	-2.97688300	-0.06686500
N	2.61546500	-0.90146000	1.58421700
N	-3.42207400	0.91309300	1.21039800
N	-3.52054400	1.37629300	-0.88736300
C	2.13844300	-3.77146300	0.73247200
C	2.93593000	-3.28947900	1.77849100
H	3.47115900	-4.04444100	2.35255700
C	3.28230300	-1.95086400	2.05928700
C	2.07426700	-5.25780100	0.52651100
H	1.11387500	-5.61806500	0.93165700
H	2.88708100	-5.77518100	1.04978200
H	2.08952100	-5.52888400	-0.53689700
C	4.50388300	-1.73688900	2.90801700
H	4.70516400	-2.62481400	3.52016600
H	4.40949900	-0.85506200	3.55356800
H	5.37826900	-1.56837300	2.26134600
C	0.94119900	-3.45489300	-1.32488100
C	-0.38234700	-3.91591000	-1.46475100
C	-0.81654900	-4.32865300	-2.72904300
H	-1.84357000	-4.68227400	-2.84895900
C	0.03229200	-4.30472100	-3.82677600
H	-0.32315000	-4.63584200	-4.80538300
C	1.33847300	-3.85145200	-3.67503500
H	2.00004100	-3.82679600	-4.54302400
C	1.81448000	-3.41186100	-2.43806800
C	-1.34375600	-3.96606800	-0.29713500
H	-0.77879600	-3.73265300	0.61566700
C	-1.95744700	-5.35257300	-0.11166800
H	-2.60984100	-5.63217300	-0.95438600
H	-2.57088700	-5.37752400	0.80233600
H	-1.18209100	-6.12888100	-0.02191800
C	-2.42373600	-2.89307600	-0.43564400
H	-1.99203300	-1.88429000	-0.52122400
H	-3.07989400	-2.90068500	0.44831300
H	-3.04305600	-3.07286500	-1.33056600
C	3.23641600	-2.89594100	-2.31274600
H	3.25302000	-2.23857800	-1.43024600
C	4.24135200	-4.02124600	-2.05702200
H	4.22771800	-4.75202900	-2.88169800
H	4.03273700	-4.56093000	-1.12420600
H	5.26197600	-3.61390700	-1.98232100
C	3.67448500	-2.07069800	-3.52012100
H	2.95615700	-1.27081900	-3.75198900
H	3.79071100	-2.69246600	-4.42128400
H	4.65328700	-1.60707300	-3.32569900
C	3.14144100	0.41417100	1.80013100
C	2.65108200	1.19744900	2.87236100
C	3.26780400	2.42685700	3.12130400
H	2.90976600	3.04393100	3.94558900
C	4.32218600	2.88620300	2.33895900
H	4.79341400	3.84610600	2.56239400
C	4.74263200	2.13809000	1.24933500
H	5.53384200	2.52393500	0.60166800
C	4.15082400	0.90689400	0.94676300
C	1.48295800	0.74120700	3.72853900
H	0.76810100	0.24864200	3.05315600
C	1.88121600	-0.29591300	4.77964000
H	2.66105600	0.09950900	5.45100000
H	2.24506000	-1.22863200	4.33011100
H	1.00557800	-0.55983100	5.39163800
C	0.73461700	1.89659200	4.38299500
H	1.34246400	2.40899400	5.14592700
H	-0.15863700	1.50892100	4.89311000

H	0.39690300	2.62603300	3.63327300
C	4.56991600	0.19458500	-0.32446200
H	3.95202400	-0.71141400	-0.41054000
C	6.03380000	-0.24258900	-0.31413800
H	6.70934900	0.62462600	-0.24691000
H	6.28065000	-0.78250800	-1.24181800
H	6.26181200	-0.90858100	0.53059700
C	4.28014300	1.08049400	-1.53842600
H	3.23223600	1.40843500	-1.55287800
H	4.48057200	0.53949000	-2.47466800
H	4.90178200	1.98902500	-1.53994400
C	-4.20576300	0.87483100	0.11675000
C	-5.58613300	0.33661200	0.03206500
C	-6.69153300	1.17811100	0.18494100
H	-6.54025300	2.23631800	0.40746100
C	-7.97899800	0.66882400	0.04083400
H	-8.83952100	1.33077700	0.16046500
C	-8.16928500	-0.67942100	-0.26033300
H	-9.18042800	-1.07639000	-0.37549400
C	-7.06744100	-1.51925700	-0.41478400
H	-7.21089100	-2.57596600	-0.65088400
C	-5.77712300	-1.01505700	-0.26932700
H	-4.91170400	-1.66879900	-0.39038000
C	-3.77165400	0.60684200	2.61075500
C	-4.94400300	1.47645500	3.07451400
H	-4.73018600	2.54061000	2.89071700
H	-5.09715900	1.34307600	4.15577700
H	-5.88393500	1.21078200	2.57312300
C	-4.10704000	-0.87901000	2.76412800
H	-5.00659800	-1.15931500	2.19940000
H	-4.29372300	-1.10792600	3.82415300
H	-3.25872300	-1.49182900	2.42713600
C	-2.56126600	0.92571900	3.48176100
H	-2.28705300	1.98529900	3.41816500
H	-1.69120300	0.33127500	3.17576800
H	-2.79961500	0.67458300	4.52577600
C	-3.75643800	1.41121700	-2.32984200
C	-5.22026500	1.65156600	-2.70269100
H	-5.62135900	2.53815800	-2.18941900
H	-5.86413100	0.79344700	-2.47171500
H	-5.28085200	1.83146700	-3.78646800
C	-2.92922900	2.58515900	-2.85497000
H	-2.97106600	2.62553200	-3.95303800
H	-1.87839000	2.49528000	-2.55060700
H	-3.31608800	3.53449000	-2.45486800
C	-3.26943200	0.09627900	-2.94765700
H	-3.34185200	0.13582400	-4.04514900
H	-3.88496400	-0.74472300	-2.59441200
H	-2.22877600	-0.09906800	-2.65829000
C	0.30454600	0.20011000	-0.53322800
H	0.80764400	1.10501800	-0.15310200
C	0.80413700	-0.06701500	-1.94605500
H	0.27369300	-0.93978000	-2.36171900
H	1.86009600	-0.37443700	-1.87095100
C	0.69403800	1.11104100	-2.90153800
H	-0.35469600	1.36093100	-3.10720700
H	1.17274800	2.01334400	-2.48966300
H	1.17229600	0.88486600	-3.86721100
C	-1.31066800	3.27686200	0.43967100
O	-0.95744800	2.27447900	1.43664000
H	-1.97503400	4.06742100	0.83278000
C	-0.15455600	3.83331400	-0.29560000
C	-0.32041200	4.65026900	-1.42837500
C	1.15003100	3.54280400	0.13751900
C	0.77591000	5.11276600	-2.14181300
H	-1.32739900	4.90819000	-1.76341500
C	2.25426000	4.01395300	-0.55609100
H	1.28152300	2.93730800	1.03552700
C	2.05687900	4.77399100	-1.70530100

H	0.66600400	5.72446800	-3.03733600
H	3.26688800	3.79400600	-0.22259000
N	3.21632800	5.22179800	-2.46693700
O	3.01967400	5.92379300	-3.43695200
O	4.31458700	4.86273500	-2.08967300

Cartesian coordinates of the optimized geometry of Int-**2a** at PBE0-D3BJ/def2-SVP level of theory:

Ga	-1.91079800	-0.96656700	-0.44123000
Cl	-1.52413000	-1.85375700	-2.45686000
Si	2.19768700	0.11206900	0.12323400
O	0.66845700	-0.38551200	0.49501900
N	-2.40570000	-2.57530800	0.59651000
N	-3.76478200	-0.32033500	-0.62844700
N	2.88427400	-0.43535600	-1.47143000
N	3.13626500	-1.50221100	0.38152100
C	-3.55154900	-3.18344200	0.27979900
C	-4.57997000	-2.58116300	-0.46040800
H	-5.43084200	-3.21974200	-0.69250400
C	-4.72381300	-1.22719300	-0.80392000
C	-3.77440300	-4.60616300	0.71097600
H	-3.19563200	-5.27694600	0.05689700
H	-4.83325700	-4.87940900	0.63444300
H	-3.42305400	-4.78591900	1.73513200
C	-6.02991200	-0.81622700	-1.42185600
H	-5.91052000	-0.81157500	-2.51737500
H	-6.32659800	0.19846700	-1.12825800
H	-6.82971600	-1.52258400	-1.16904100
C	-1.50070800	-3.22162500	1.49531400
C	-0.57571900	-4.17109000	1.01368100
C	0.26318700	-4.80616800	1.93429600
H	0.97319000	-5.55618600	1.57772100
C	0.20679400	-4.50931900	3.29009700
H	0.87461100	-5.01472200	3.99170300
C	-0.70342100	-3.56451300	3.74940100
H	-0.74666600	-3.33488500	4.81580000
C	-1.57351900	-2.91176900	2.87156700
C	-0.45724400	-4.50648900	-0.45922200
H	-1.29827300	-4.03374800	-0.98473800
C	-0.51330700	-6.00844300	-0.73209000
H	0.36235500	-6.53423900	-0.31913300
H	-0.52552300	-6.19642300	-1.81672400
H	-1.41050500	-6.47464000	-0.29704700
C	0.81702500	-3.89734800	-1.04080200
H	0.88675500	-2.82421900	-0.81661600
H	0.83963100	-4.01900900	-2.13453200
H	1.70355700	-4.39158700	-0.61317200
C	-2.60556000	-1.93858800	3.40859200
H	-2.88317100	-1.27992400	2.57247800
C	-3.88533900	-2.65471400	3.84500000
H	-3.67370700	-3.39231300	4.63570400
H	-4.36632800	-3.18157800	3.00948700
H	-4.61389300	-1.93050800	4.24268400
C	-2.06741800	-1.06517200	4.53887900
H	-1.10851800	-0.59718100	4.27224100
H	-1.91684000	-1.64121800	5.46519100
H	-2.78281900	-0.26208800	4.77175900
C	-4.05143300	1.07170900	-0.78277100
C	-3.94160700	1.68911300	-2.04485500
C	-4.20732100	3.05982900	-2.13536800
H	-4.11949800	3.55539700	-3.10501000
C	-4.57717200	3.79828100	-1.01988000
H	-4.77536100	4.86866900	-1.11181600
C	-4.69045800	3.17273500	0.21756500
H	-4.98026000	3.76109800	1.08979100
C	-4.43265200	1.80795600	0.36366300
C	-3.55911500	0.91819400	-3.29260900

H	-3.49378200	-0.14557600	-3.02444800
C	-4.60694700	1.06135400	-4.39659800
H	-4.66143100	2.09498600	-4.77342000
H	-5.61388600	0.78526800	-4.04843900
H	-4.35134800	0.41444700	-5.25006000
C	-2.17869000	1.32778100	-3.80259500
H	-2.18532100	2.35827400	-4.18931600
H	-1.85696500	0.65750300	-4.61369100
H	-1.41929600	1.27506200	-3.01067700
C	-4.59655300	1.13237800	1.71433200
H	-3.90108900	0.27968400	1.72627300
C	-6.00466300	0.56174100	1.89857500
H	-6.76272800	1.35730900	1.81925600
H	-6.10269400	0.09761900	2.89278900
H	-6.24150900	-0.20798500	1.15252000
C	-4.24342100	2.03969600	2.88951200
H	-3.26271500	2.51989600	2.76037900
H	-4.21640200	1.45543000	3.82181800
H	-4.99244300	2.83429700	3.03210500
C	3.55046600	-1.43641100	-0.87700100
C	4.64086300	-2.22775900	-1.49421000
C	5.88155900	-1.61324200	-1.69398700
H	6.02498400	-0.57430100	-1.38917300
C	6.92532000	-2.32561000	-2.27893000
H	7.89340300	-1.84272400	-2.42909200
C	6.73131000	-3.64753600	-2.67744200
H	7.54813400	-4.20327500	-3.14355500
C	5.49300500	-4.25846200	-2.48309900
H	5.33570900	-5.29174900	-2.80011300
C	4.44984200	-3.55460200	-1.88757200
H	3.47624600	-4.02475100	-1.74540800
C	2.77793700	-0.01326000	-2.88043000
C	3.73798800	1.15248800	-3.12209200
H	3.51488000	1.98965700	-2.44478900
H	3.63247400	1.51870600	-4.15425600
H	4.78334600	0.83894000	-2.97720100
C	3.07091300	-1.16325500	-3.84349800
H	4.12588000	-1.46601900	-3.84885700
H	2.81456800	-0.83350200	-4.86052100
H	2.44908900	-2.04080600	-3.60971600
C	1.33435600	0.43817800	-3.09402300
H	1.08739000	1.31001300	-2.47150200
H	0.63482200	-0.38576900	-2.89376300
H	1.19923000	0.75205000	-4.13977500
C	3.73122800	-2.11705300	1.57242400
C	5.12174000	-1.53450600	1.84208100
H	5.07793600	-0.43790700	1.91117200
H	5.82944900	-1.80680000	1.04565200
H	5.51867000	-1.92224700	2.79249400
C	2.78500700	-1.76980100	2.72356900
H	3.13774600	-2.22692700	3.65922800
H	1.76940800	-2.13378200	2.51339900
H	2.73737300	-0.68075000	2.87721100
C	3.81874200	-3.63679600	1.44111500
H	4.16836400	-4.06712600	2.39146100
H	4.52360300	-3.94075800	0.65598900
H	2.82930900	-4.05722900	1.21988100
C	-0.56206600	0.31133000	0.28856100
H	-0.39821300	1.12618000	-0.43774000
C	-1.11351900	0.90910800	1.57647100
H	-1.56778300	0.12178700	2.19443900
H	-1.93920600	1.56900900	1.26802100
C	-0.13440000	1.68256100	2.43863000
H	0.62694700	1.00316900	2.84974800
H	0.37661900	2.47389200	1.87374000
H	-0.65522200	2.14268300	3.29318400
C	3.16601800	1.52822900	0.76034500
O	1.98135400	1.94540700	-0.01292600
H	4.06510800	1.72879800	0.14882000

C	3.34382500	2.07725100	2.15247300
H	2.44045900	1.90386300	2.75325900
H	4.13750100	1.46531300	2.62136700
C	3.73541000	3.54738100	2.23036100
H	4.65409300	3.71147600	1.63889800
H	2.95146500	4.15494800	1.75181500
H	3.16151900	3.57753000	-0.88458200
C	0.45789700	4.60761600	-1.85700700
C	0.25639900	4.94565800	-0.33807600
O	1.38424800	4.33917100	0.25999700
O	1.07431000	3.33637600	-1.79380100
C	0.26606900	6.43297900	-0.01590600
H	-0.53658600	6.96590300	-0.55005900
H	0.11007600	6.57663100	1.06422400
H	1.23018400	6.88780500	-0.27758400
C	-1.02125700	4.31813100	0.21601600
H	-1.09365500	3.26022700	-0.06918300
H	-1.00224100	4.37771900	1.31422100
H	-1.92670700	4.83033000	-0.14107800
C	1.41701000	5.58273600	-2.54392100
H	1.69410900	5.16455000	-3.52306500
H	0.96216800	6.57193500	-2.70267900
H	2.33822000	5.70675600	-1.95725500
C	-0.82747200	4.51302300	-2.65719100
H	-1.36752800	5.47274500	-2.64917300
H	-0.59540800	4.25925800	-3.70257900
H	-1.49014000	3.73650600	-2.25830000
B	1.95745100	3.37588300	-0.65553400
C	3.95155400	4.02336100	3.65868800
H	4.73979600	3.41799000	4.14605200
H	3.03192600	3.84035800	4.24540200
C	4.31685700	5.49812100	3.75458500
H	3.52854600	6.09278500	3.26096300
H	5.23529900	5.68103100	3.16890100
C	4.51367700	5.98470700	5.18122000
H	5.31949100	5.42772700	5.68695800
H	4.77636400	7.05324000	5.21635600
H	3.59779100	5.84826700	5.77915800

Cartesian coordinates of the optimized geometry of Int-**2b** at PBE0-D3BJ/def2-SVP level of theory:

Ga	-2.18422200	-0.89297800	-0.16049600
Cl	-2.18703600	-2.03915200	-2.07814300
Si	2.06292600	-0.06994900	-0.42552500
O	0.58143400	-0.41705600	0.20045200
N	-2.65362300	-2.30516100	1.13852600
N	-3.97957600	-0.07422100	-0.16546500
N	2.46374700	-0.84748500	-2.02293200
N	2.91688300	-1.72452300	-0.10817600
C	-3.88786400	-2.81283900	1.08445000
C	-4.96414000	-2.18871600	0.43793000
H	-5.89893600	-2.74722800	0.43135900
C	-5.03535800	-0.87881500	-0.06197500
C	-4.16988600	-4.14224600	1.72714500
H	-3.82080600	-4.94510000	1.05897400
H	-5.24506800	-4.27845200	1.89242700
H	-3.63456700	-4.26578000	2.67696300
C	-6.38541700	-0.40200400	-0.51732900
H	-6.48364100	-0.61451800	-1.59413800
H	-6.51067100	0.68007200	-0.38840200
H	-7.19047600	-0.93208400	0.00597200
C	-1.67524100	-2.94066000	1.96492600
C	-0.94059400	-4.04278800	1.47945400
C	-0.02230100	-4.65543700	2.33736500
H	0.53828800	-5.52403400	1.98380500
C	0.18755100	-4.18610600	3.62795900
H	0.91622300	-4.67510000	4.27878600

C	-0.53868400	-3.09452900	4.08996200
H	-0.37641000	-2.73408600	5.10747900
C	-1.48684700	-2.46197200	3.28081800
C	-1.11363700	-4.57074600	0.06880800
H	-1.97381200	-4.05684100	-0.38155300
C	-1.38537400	-6.07376900	0.03023500
H	-0.51698400	-6.65609300	0.37736300
H	-1.60284000	-6.39410600	-1.00033000
H	-2.24162800	-6.35606200	0.66121100
C	0.09983000	-4.22287900	-0.79328800
H	0.35353800	-3.15575500	-0.71986700
H	-0.10236500	-4.45038200	-1.85093100
H	0.97610100	-4.80795400	-0.47235700
C	-2.33279500	-1.33299700	3.83979700
H	-2.67757800	-0.73892000	2.98027600
C	-3.58878600	-1.86209800	4.53608100
H	-3.32370800	-2.53288400	5.36893000
H	-4.23861000	-2.41769500	3.84646000
H	-4.17913100	-1.02785600	4.94698300
C	-1.55587400	-0.40850600	4.77363300
H	-0.60718500	-0.07575500	4.32746800
H	-1.32411400	-0.89672400	5.73296100
H	-2.15345400	0.48629100	5.00461600
C	-4.15309600	1.30956100	-0.47991900
C	-4.21251500	1.73480700	-1.82261100
C	-4.34615600	3.10440500	-2.07592700
H	-4.38596500	3.45231600	-3.11081900
C	-4.43221500	4.02460000	-1.04024500
H	-4.53369800	5.08989900	-1.26019100
C	-4.39059400	3.58727600	0.27999900
H	-4.46429400	4.31733600	1.08775200
C	-4.25287600	2.23206100	0.58836500
C	-4.16640900	0.76130900	-2.98446000
H	-4.15250100	-0.25660500	-2.57020400
C	-5.39886200	0.89078500	-3.88060800
H	-5.42146500	1.86179800	-4.40013700
H	-6.33580800	0.80207500	-3.31055600
H	-5.39256500	0.10600000	-4.65282300
C	-2.88831900	0.91715200	-3.80502700
H	-2.86739300	1.88234900	-4.33356100
H	-2.81582100	0.11408700	-4.55374600
H	-1.99191000	0.85869000	-3.17353100
C	-4.26346800	1.76463200	2.03429600
H	-3.66090000	0.84431300	2.07455700
C	-5.67360700	1.39247300	2.49899800
H	-6.35407700	2.25457100	2.41278400
H	-5.65767700	1.07685200	3.55421200
H	-6.09853800	0.56565200	1.91541300
C	-3.64701800	2.77148600	3.00162200
H	-2.65962900	3.12001900	2.66556600
H	-3.52507700	2.31350600	3.99490100
H	-4.28904400	3.65627100	3.13414800
C	3.10780100	-1.85478300	-1.41313800
C	3.97865400	-2.84678100	-2.08482600
C	5.21375900	-2.41577600	-2.58079100
H	5.50961200	-1.37128800	-2.46050900
C	6.05889700	-3.31769400	-3.22135800
H	7.02470600	-2.97862100	-3.60221800
C	5.66878200	-4.64690800	-3.38153900
H	6.32904400	-5.35226100	-3.89121800
C	4.43387000	-5.07398500	-2.89509900
H	4.12157900	-6.11209100	-3.02788300
C	3.59018600	-4.17918000	-2.24222100
H	2.61550300	-4.50486600	-1.87726000
C	2.10339700	-0.62616800	-3.43883700
C	3.05900300	0.40548100	-4.03750300
H	3.02001800	1.35046500	-3.47851200
H	2.77521100	0.62111100	-5.07834500
H	4.09297400	0.02677200	-4.03732800

C	2.13903300	-1.92016700	-4.25166600
H	3.15339800	-2.30823000	-4.40883000
H	1.70704600	-1.71050100	-5.24064700
H	1.52563400	-2.70268100	-3.77998900
C	0.66772000	-0.10458700	-3.44510500
H	0.58752200	0.85541800	-2.91704100
H	-0.01683200	-0.84573200	-3.00895200
H	0.35003000	0.08290400	-4.48149400
C	3.58340900	-2.35032100	1.03971400
C	5.10143800	-2.16045300	0.96509700
H	5.36463300	-1.11346500	0.76225500
H	5.54051800	-2.77647400	0.16767400
H	5.56907900	-2.45608200	1.91605300
C	2.99721200	-1.66354500	2.27524700
H	3.43354100	-2.08775000	3.19073100
H	1.90751700	-1.80985600	2.30735900
H	3.20263800	-0.58337800	2.27333800
C	3.26697600	-3.84240300	1.12588200
H	3.69924000	-4.25416000	2.05022600
H	3.69151900	-4.39997900	0.28075700
H	2.18270100	-3.99843500	1.15832600
C	-0.63430400	0.32906300	0.17257900
H	-0.58598400	1.06486800	-0.64882600
C	-0.87282400	1.06182500	1.48436300
H	-1.19980100	0.35012400	2.25470900
H	-1.72419600	1.73493600	1.30223200
C	0.29572600	1.85888900	2.02546800
H	1.14330700	1.20588400	2.28215700
H	0.64863400	2.59215900	1.29281900
H	0.01181500	2.39626900	2.94359400
C	3.20471600	1.34581400	-0.10814600
O	1.97807300	1.76471700	-0.77145500
H	4.03188300	1.33342700	-0.84354000
H	3.22323900	3.04909000	-2.06835900
C	0.41243200	4.16540100	-2.91464500
C	0.45503900	4.76610300	-1.47033300
O	1.67942200	4.25173300	-0.97845300
O	1.02966700	2.90445500	-2.72607200
C	0.50258700	6.28484500	-1.41644300
H	-0.38197700	6.72927600	-1.89917900
H	0.52060200	6.61838600	-0.36781400
H	1.40635500	6.67041200	-1.90532400
C	-0.69646200	4.26083500	-0.60416400
H	-0.78679200	3.16844900	-0.66928400
H	-0.49924700	4.52534600	0.44535100
H	-1.66147200	4.70154100	-0.89177200
C	1.25740800	4.97202300	-3.90146600
H	1.37520300	4.38139100	-4.82196600
H	0.79066800	5.93369500	-4.16164900
H	2.25926100	5.16471300	-3.49159500
C	-0.98352300	3.96979800	-3.47317300
H	-1.51081500	4.93263900	-3.55965700
H	-0.92318000	3.52066400	-4.47586700
H	-1.57786300	3.30327500	-2.83796800
B	2.03607600	3.07061400	-1.71971400
C	3.64096500	2.07427700	1.11671400
C	3.12085000	3.32518600	1.47628200
C	4.66142600	1.51944200	1.90639200
C	3.56862600	3.97351900	2.62102400
H	2.38631500	3.80537100	0.82501500
C	5.11812900	2.15701000	3.05086500
H	5.10996400	0.56780100	1.62199900
C	4.55131900	3.37755500	3.40402400
H	3.17250500	4.94587300	2.91414700
H	5.90133900	1.73028500	3.67703800
N	5.01352300	4.05192700	4.61708500
O	5.88223600	3.50757900	5.26602900
O	4.49915700	5.11170400	4.90244300

Cartesian coordinates of the optimized geometry of Int-**3a** at PBE0-D3BJ/def2-SVP level of theory:

Ga	2.40994000	-0.18585000	-0.35096600
Cl	1.75182500	0.05493200	-2.48333800
Si	-1.64455400	0.21002600	0.06106200
O	-0.10210700	0.42653600	0.74782600
N	3.71677100	1.28235700	-0.20437200
N	3.75989700	-1.61588400	-0.44786800
N	-2.23298000	1.15006600	-1.45184000
N	-2.03738600	2.13248900	0.46118300
C	4.92449400	1.13210400	-0.75026700
C	5.42295500	-0.08178700	-1.25364500
H	6.38995500	-0.02274400	-1.75072300
C	4.91976500	-1.37558600	-1.05237200
C	5.84418900	2.31846500	-0.83468000
H	6.86360900	2.00329000	-1.08528600
H	5.85827200	2.87765000	0.11114600
H	5.49549100	3.01889000	-1.60665700
C	5.74214000	-2.52586700	-1.55902300
H	5.67689000	-3.40102700	-0.89980100
H	6.79283800	-2.24110100	-1.69190200
H	5.34284400	-2.83154200	-2.53982000
C	3.35422300	2.55431300	0.33642700
C	2.97395700	3.61466300	-0.51356100
C	2.72325800	4.86408000	0.06270500
H	2.43644900	5.69858200	-0.58156300
C	2.81451200	5.06098400	1.43382900
H	2.61515200	6.04640600	1.86145600
C	3.13148900	3.99155000	2.26388200
H	3.17281400	4.14385500	3.34371600
C	3.40269000	2.72661600	1.73701800
C	2.77407100	3.43672100	-2.00696900
H	3.14030000	2.43874100	-2.28816900
C	3.51843500	4.47973300	-2.84028200
H	3.10984900	5.49115500	-2.68707900
H	3.42158700	4.24815600	-3.91204200
H	4.59171700	4.52265200	-2.60062900
C	1.28172900	3.46452800	-2.33924400
H	0.73066200	2.69712500	-1.77850600
H	1.12200100	3.27168600	-3.41116000
H	0.85551100	4.45213000	-2.09828300
C	3.78042000	1.57399300	2.64641200
H	3.42445900	0.65543400	2.15380200
C	5.29767100	1.44545700	2.79224700
H	5.73000200	2.37168100	3.20327600
H	5.78592900	1.23801500	1.82969400
H	5.55246000	0.62089300	3.47623100
C	3.10596800	1.64191500	4.01245400
H	2.02119700	1.80049900	3.91877200
H	3.51676200	2.45218200	4.63532600
H	3.26620900	0.70063200	4.55947200
C	3.42984300	-2.92216600	0.02172600
C	2.70586700	-3.81758400	-0.78832100
C	2.35981700	-5.06297100	-0.25167000
H	1.79271000	-5.76725300	-0.86544100
C	2.72133900	-5.41791100	1.04045900
H	2.43900800	-6.39439200	1.44085000
C	3.43836700	-4.52275000	1.82888300
H	3.71128300	-4.80597000	2.84703800
C	3.79955300	-3.26398400	1.34478800
C	2.30201500	-3.47914400	-2.20950100
H	2.69903800	-2.48169200	-2.44673600
C	2.89002600	-4.47286400	-3.21233500
H	2.46886100	-5.48192900	-3.07691400
H	3.98283000	-4.55754600	-3.11364900
H	2.66352100	-4.15632400	-4.24231400
C	0.78345300	-3.39911300	-2.35356800

H	0.31363700	-4.37419800	-2.15184100
H	0.51019100	-3.09360100	-3.37511600
H	0.34712000	-2.66174900	-1.66487500
C	4.57795700	-2.29243800	2.21621300
H	4.30107100	-1.27974300	1.88241800
C	6.08866900	-2.43440800	2.01551900
H	6.41995900	-3.45517400	2.26515900
H	6.63112900	-1.73204700	2.66766600
H	6.39192000	-2.22343000	0.98199400
C	4.23879900	-2.40248500	3.70022100
H	3.15528800	-2.35471300	3.87993800
H	4.71184600	-1.57835700	4.25589300
H	4.61580300	-3.33974200	4.13872300
C	-2.43982800	2.29497600	-0.78153200
C	-3.09655100	3.51553900	-1.32314400
C	-4.46547100	3.69326800	-1.09477200
H	-5.02007000	2.93223300	-0.54129700
C	-5.11147000	4.83707100	-1.55566800
H	-6.18064600	4.96635500	-1.37285500
C	-4.39405200	5.81686500	-2.24099400
H	-4.89977900	6.71686900	-2.59835400
C	-3.02988500	5.64373800	-2.46950100
H	-2.46274700	6.40630300	-3.00816400
C	-2.38291600	4.49601300	-2.01705200
H	-1.31888900	4.35376300	-2.20829100
C	-2.67105600	0.76042300	-2.80315600
C	-4.19816600	0.65350500	-2.86957500
H	-4.55978100	-0.08007100	-2.13375100
H	-4.50773000	0.31111100	-3.86946400
H	-4.68124000	1.62373000	-2.68618700
C	-2.15129200	1.74936000	-3.85082900
H	-2.63253800	2.73248700	-3.77671100
H	-2.35538000	1.35565200	-4.85786400
H	-1.06319400	1.87236800	-3.74491200
C	-2.06841800	-0.60916500	-3.12085100
H	-2.46413200	-1.38013000	-2.44868100
H	-0.97297000	-0.58834700	-3.03067100
H	-2.33290900	-0.88075500	-4.15410900
C	-1.67800400	3.15829900	1.45164400
C	-2.83219800	4.11942600	1.75458000
H	-3.74450900	3.56569000	2.02509200
H	-3.06598800	4.77848000	0.90857900
H	-2.55879900	4.75724200	2.60889600
C	-1.27052800	2.46079900	2.75166000
H	-0.88476500	3.21086900	3.45791100
H	-0.48538400	1.72173100	2.54860500
H	-2.12026600	1.95587200	3.22710600
C	-0.46028800	3.93232300	0.94284400
H	-0.11982800	4.65673300	1.69713800
H	-0.69013600	4.48854400	0.02372800
H	0.36627500	3.23817600	0.73779400
C	0.84753400	-0.61839200	0.79865700
H	0.43865600	-1.50282600	0.26604400
C	1.10719200	-1.05981900	2.22982200
H	1.61021700	-0.26075700	2.79699200
H	1.80986900	-1.90638600	2.18649500
C	-0.15306000	-1.48545600	2.96885300
H	-0.82657800	-0.63118200	3.12746100
H	-0.71868300	-2.24280400	2.40447000
H	0.08917800	-1.90453800	3.95802300
C	-3.23000100	-0.46329300	0.95562600
O	-3.08263500	-1.87444000	1.13238800
H	-4.00765700	-0.29138100	0.18494900
C	-3.74829000	0.12057700	2.25399100
H	-3.01114100	-0.05009400	3.05650000
H	-3.83227600	1.21047200	2.12608400
C	-5.09394700	-0.44843500	2.68252300
H	-5.82853700	-0.29653600	1.86949300
H	-5.00006100	-1.54055700	2.80565500

H	-1.30957600	-1.10017300	-0.61498100
C	-3.85248400	-4.65463000	-0.91509800
C	-4.81542200	-3.54775400	-1.47356300
O	-4.24090400	-2.35367600	-0.92548100
O	-3.43075400	-4.09046500	0.33176500
C	-4.85290300	-3.43751900	-2.98554100
H	-5.21374800	-4.37506400	-3.43492800
H	-5.53928100	-2.63062500	-3.28155700
H	-3.86366200	-3.20527300	-3.39863500
C	-6.22872100	-3.66123500	-0.91316100
H	-6.21085800	-3.76551800	0.18182900
H	-6.78100200	-2.74256100	-1.15830900
H	-6.77180500	-4.51756400	-1.33811900
C	-2.60607500	-4.82795700	-1.77468800
H	-1.87723000	-5.43177200	-1.21533900
H	-2.82565500	-5.33428400	-2.72567500
H	-2.13855400	-3.85666800	-1.99131800
C	-4.50981500	-5.99624300	-0.65520400
H	-4.92698200	-6.41540500	-1.58340500
H	-3.76210900	-6.70274200	-0.26665900
H	-5.31225400	-5.91291800	0.08879900
B	-3.55352800	-2.72933300	0.21134100
C	-5.62543800	0.16431700	3.96918000
H	-5.71220000	1.26030300	3.84707200
H	-4.88450400	0.01495000	4.77599500
C	-6.96871000	-0.39959900	4.41104100
H	-7.70670700	-0.25007900	3.60267900
H	-6.87734900	-1.49378400	4.53027400
C	-7.49052000	0.21451400	5.70008600
H	-7.62360000	1.30387200	5.59821700
H	-8.46173100	-0.21287200	5.99304600
H	-6.78894700	0.04797200	6.53350500

Cartesian coordinates of the optimized geometry of Int-**3b** at PBE0-D3BJ/def2-SVP level of theory:

Ga	-2.21454700	-0.85497800	-0.40481100
Cl	-1.40426300	-1.44990300	-2.40864800
Si	1.54956700	0.29147700	-0.41573400
O	0.37218100	-0.33293000	0.63893600
N	-2.99201100	-2.55463700	0.24047600
N	-3.95085900	-0.05527200	-0.89860300
N	2.56511400	-0.55215100	-1.74464400
N	2.84258600	-1.02524800	0.33560800
C	-4.06268900	-3.03504800	-0.38579200
C	-4.89034200	-2.25910300	-1.21542000
H	-5.70628000	-2.79492300	-1.69809600
C	-4.90284000	-0.86545100	-1.36941000
C	-4.42918200	-4.48532900	-0.24021800
H	-5.51139600	-4.63189900	-0.34451800
H	-4.08955800	-4.90721000	0.71360500
H	-3.93437800	-5.05226900	-1.04515600
C	-6.06868900	-0.27192400	-2.10909700
H	-6.52422600	0.55538800	-1.54775100
H	-6.83153400	-1.03089400	-2.31701200
H	-5.72354200	0.15349000	-3.06325800
C	-2.29484700	-3.33566100	1.21408500
C	-1.28749500	-4.23670100	0.81720300
C	-0.58685700	-4.92923300	1.81080200
H	0.20292400	-5.62541800	1.51800700
C	-0.87412600	-4.74759700	3.15619000
H	-0.31031200	-5.29233400	3.91698900
C	-1.88739600	-3.87177700	3.53252100
H	-2.11477100	-3.74077700	4.59166600
C	-2.61554700	-3.15582300	2.57990400
C	-0.94751800	-4.48649400	-0.63808400
H	-1.63291000	-3.89083100	-1.25693000
C	-1.11616600	-5.95864500	-1.01580600

H	-0.38607300	-6.59630600	-0.49233500
H	-0.95853300	-6.09669600	-2.09683200
H	-2.11859500	-6.33641300	-0.76584600
C	0.46391900	-4.00757200	-0.96275500
H	0.59914900	-2.94903500	-0.69984500
H	0.66532800	-4.11700300	-2.03905300
H	1.21074500	-4.59737000	-0.40836500
C	-3.75169900	-2.24340500	3.00346900
H	-3.79589800	-1.43185000	2.26186600
C	-5.09865200	-2.96879500	2.95478100
H	-5.09396500	-3.84504300	3.62255800
H	-5.34632700	-3.31569500	1.94322200
H	-5.90887100	-2.29796600	3.28114200
C	-3.54852600	-1.61164400	4.37659800
H	-2.56863000	-1.12115100	4.46366200
H	-3.63151800	-2.35473000	5.18518000
H	-4.32528600	-0.85430900	4.56064500
C	-4.18917400	1.35460100	-0.91345600
C	-3.88154200	2.12768700	-2.05213300
C	-4.23800600	3.48092600	-2.05025200
H	-4.01623400	4.08883400	-2.93064500
C	-4.87834800	4.05966800	-0.96256700
H	-5.16412600	5.11401400	-0.99065400
C	-5.13627700	3.29587300	0.17020500
H	-5.61561200	3.76004300	1.03460600
C	-4.78708200	1.94464800	0.22321000
C	-3.17443700	1.54909800	-3.26043700
H	-3.10714000	0.46002600	-3.12579400
C	-3.92174300	1.81694400	-4.56646800
H	-3.94016800	2.88971600	-4.81600000
H	-4.96593200	1.47074100	-4.52713400
H	-3.42529200	1.29621700	-5.39951300
C	-1.74290400	2.07822000	-3.34244000
H	-1.73052100	3.16787500	-3.50519000
H	-1.20888400	1.60594400	-4.18020300
H	-1.17344400	1.86844600	-2.42515700
C	-5.07852200	1.13202800	1.46974200
H	-4.42412900	0.24877300	1.42583600
C	-6.51800700	0.61665700	1.49576100
H	-7.23577300	1.45195500	1.46050400
H	-6.70738400	0.04665700	2.41894200
H	-6.72926800	-0.04937100	0.64743200
C	-4.74801100	1.89175200	2.75188400
H	-3.72810800	2.30275300	2.72710500
H	-4.82121300	1.22140900	3.62127100
H	-5.44510700	2.72588700	2.92744700
C	3.26963600	-1.30382900	-0.87262400
C	4.34824300	-2.26603300	-1.23310700
C	5.68343300	-1.84907200	-1.26812300
H	5.92911300	-0.81571300	-1.02473900
C	6.69340200	-2.74946300	-1.59309200
H	7.73158200	-2.41045200	-1.61188300
C	6.38149000	-4.07685800	-1.88550300
H	7.17495400	-4.78351500	-2.13933900
C	5.05420400	-4.49823000	-1.84854200
H	4.80069600	-5.53675300	-2.07303300
C	4.04078600	-3.59806100	-1.52399200
H	3.00341600	-3.93081200	-1.49472600
C	2.60759500	-0.53465500	-3.21666600
C	4.01244600	-0.22564000	-3.74372500
H	4.36820500	0.74856100	-3.37514300
H	3.98638100	-0.17269000	-4.84241800
H	4.74382600	-0.99502500	-3.46588800
C	2.11780200	-1.86946400	-3.78705400
H	2.80897100	-2.68965200	-3.55080500
H	2.04190800	-1.80340100	-4.88319300
H	1.12237500	-2.10623300	-3.38581300
C	1.66280300	0.56412100	-3.70032300
H	1.93079800	1.54445700	-3.27784300

H	0.62586700	0.34044300	-3.42265300
H	1.72543000	0.63489600	-4.79636200
C	3.23169100	-1.59674900	1.63272000
C	4.74539300	-1.53823800	1.86116300
H	5.12888500	-0.52377800	1.68259200
H	5.29273400	-2.23940500	1.21870200
H	4.96603000	-1.80480800	2.90561000
C	2.53956800	-0.77869400	2.72119400
H	2.81982400	-1.16901600	3.71034700
H	1.45233900	-0.83444800	2.60088600
H	2.83895200	0.27625300	2.67162100
C	2.73419700	-3.04084600	1.73639400
H	2.96250400	-3.44490200	2.73460700
H	3.21859900	-3.68938200	0.99260400
H	1.64589200	-3.07854100	1.59095100
C	-0.94506000	0.17872600	0.73119000
H	-0.98470100	1.21394800	0.33582800
C	-1.37251400	0.20094700	2.19223000
H	-1.22988400	-0.81445000	2.59705600
H	-2.45611400	0.39598500	2.23622000
C	-0.65150500	1.21623500	3.06434200
H	0.43629500	1.07868200	3.02203300
H	-0.85188700	2.24678500	2.73674300
H	-0.96890200	1.12782200	4.11572700
C	2.45992200	1.94914100	0.09736700
O	1.64898700	2.56940300	1.08361200
H	2.40428100	2.53028500	-0.84053700
H	0.55664800	0.98708200	-1.30899100
C	-0.67178500	5.24011100	1.06362800
C	-0.01485800	5.35836900	-0.36013400
O	0.64565800	4.08965700	-0.49390300
O	0.22859700	4.35447200	1.74430300
C	-1.00135100	5.52489700	-1.49834600
H	-1.57425900	6.45747500	-1.38320600
H	-0.45847100	5.57169600	-2.45363100
H	-1.70325800	4.68362600	-1.54637200
C	1.06568900	6.43089700	-0.42559000
H	1.77637700	6.32918000	0.40796600
H	1.62474200	6.31213800	-1.36484200
H	0.63987000	7.44407200	-0.40037500
C	-2.03403800	4.56291300	1.02538100
H	-2.34161500	4.32344700	2.05333700
H	-2.80023600	5.21030200	0.57962400
H	-2.00902200	3.62481500	0.45119000
C	-0.75439500	6.54386100	1.83381700
H	-1.36763200	7.27978400	1.29222500
H	-1.22208300	6.36306300	2.81241700
H	0.24046400	6.97210200	2.00985900
B	0.86972100	3.62910100	0.77745700
C	3.88271200	1.93584100	0.53535800
C	4.89279400	1.63296400	-0.39205800
C	4.25292500	2.25184800	1.85054300
C	6.22771300	1.61719400	-0.01897000
H	4.61631500	1.41000000	-1.42392800
C	5.58672200	2.23503600	2.24081700
H	3.47409600	2.52387200	2.56358900
C	6.56025000	1.90783600	1.30276100
H	7.02610000	1.39101000	-0.72629300
H	5.89132200	2.47107500	3.26049100
N	7.96423800	1.87298900	1.70639400
O	8.22626000	2.14932700	2.85720500
O	8.78247700	1.56508700	0.86478000

Cartesian coordinates of the optimized geometry of Int-**4a** at PBE0-D3BJ/def2-SVP level of theory:

Ga	2.42224700	-0.31549500	-0.51621900
Cl	2.52130200	1.01922900	-2.31120300

Si	-1.28124500	1.64790600	0.09591600
O	0.14025700	0.95456400	0.53669700
N	4.04976900	0.23848000	0.46864300
N	3.20938800	-1.98583500	-1.20353300
N	-1.22788500	2.93375600	-1.19810900
N	-0.97957800	3.35145600	0.89876600
C	5.22315700	-0.01028600	-0.11066300
C	5.39845500	-0.96353300	-1.12699600
H	6.40215700	-1.02750300	-1.54448500
C	4.49645200	-1.95088100	-1.55618300
C	6.43804800	0.77404900	0.29876500
H	7.35834000	0.20486600	0.11917300
H	6.39657100	1.08686900	1.34943600
H	6.48223500	1.69009800	-0.31257900
C	5.03385500	-3.01955800	-2.46542300
H	4.77935400	-4.02430100	-2.10098200
H	6.12157900	-2.93719800	-2.57220700
H	4.57011700	-2.92457800	-3.45914500
C	3.96498800	1.06064700	1.63470100
C	3.81947500	2.45671200	1.51629800
C	3.66768100	3.21384500	2.68319900
H	3.54645300	4.29709400	2.60248600
C	3.67152500	2.61710000	3.93611100
H	3.54729500	3.22439100	4.83578100
C	3.84072700	1.24033700	4.04104200
H	3.85179900	0.77662200	5.02878000
C	3.99133700	0.44027300	2.90612500
C	3.82344600	3.15954200	0.17539900
H	4.01233400	2.40821700	-0.60366900
C	4.91939500	4.22135400	0.08823300
H	4.73806300	5.05177600	0.78938800
H	4.95741700	4.64847700	-0.92600400
H	5.91176400	3.80701800	0.32080500
C	2.45428000	3.76158600	-0.12197600
H	1.66133800	3.00479500	-0.04842800
H	2.43475900	4.18204200	-1.13886000
H	2.22238100	4.56916800	0.59110100
C	4.21375000	-1.05402900	3.04496500
H	3.80028500	-1.51802400	2.13742600
C	5.70528300	-1.39494900	3.08553400
H	6.19695100	-0.89471200	3.93525600
H	6.22455400	-1.09074400	2.16741700
H	5.84879400	-2.48058400	3.20155500
C	3.50361500	-1.66505600	4.24856700
H	2.43476800	-1.40807700	4.26921800
H	3.95208200	-1.33767700	5.19962000
H	3.58919200	-2.76144000	4.21875600
C	2.45155500	-3.16349200	-1.50539400
C	1.82570600	-3.31466300	-2.75913600
C	1.18900200	-4.52787000	-3.04034600
H	0.69982000	-4.65766800	-4.00820300
C	1.15114700	-5.55812700	-2.11036400
H	0.64825800	-6.49764200	-2.35097800
C	1.73028200	-5.37681600	-0.85969200
H	1.67326700	-6.17759900	-0.11997400
C	2.38245200	-4.18540200	-0.53255600
C	1.76268400	-2.19093800	-3.77080100
H	2.45586300	-1.40049900	-3.44821900
C	2.17545900	-2.62448800	-5.17578100
H	1.46722800	-3.34884400	-5.60800300
H	3.17406600	-3.08881900	-5.18942900
H	2.19920900	-1.75276200	-5.84746700
C	0.35793600	-1.58725800	-3.77123200
H	-0.39723900	-2.31662800	-4.10277700
H	0.31117100	-0.71678900	-4.44170600
H	0.05146300	-1.25557400	-2.76998100
C	3.02474800	-4.01908100	0.83070500
H	3.10636200	-2.93596800	1.00501800
C	4.44655700	-4.58143700	0.86733500

H	4.45076500	-5.65342700	0.61269700
H	4.87975400	-4.47131400	1.87409100
H	5.11149500	-4.06153500	0.16351300
C	2.17904400	-4.60997800	1.95529700
H	1.13813800	-4.25831300	1.90739000
H	2.59173300	-4.32359700	2.93387100
H	2.16187600	-5.71038500	1.92194400
C	-1.14289300	3.91679200	-0.28005300
C	-1.32833500	5.36733400	-0.53680300
C	-2.63002300	5.87759500	-0.56644000
H	-3.47838600	5.20134600	-0.43989500
C	-2.84136200	7.24108000	-0.75900300
H	-3.86050500	7.63337700	-0.77927100
C	-1.75594600	8.09878400	-0.92824400
H	-1.92209700	9.16756200	-1.08115600
C	-0.45769800	7.59003200	-0.90351300
H	0.39579100	8.25833300	-1.03710600
C	-0.24128500	6.22929700	-0.70574600
H	0.77298300	5.83015000	-0.67759700
C	-1.22742400	3.01777300	-2.67121800
C	-2.55490100	3.59400700	-3.16826100
H	-3.40159400	3.02768000	-2.75725700
H	-2.60371700	3.52879100	-4.26550500
H	-2.66727700	4.65166500	-2.89053900
C	-0.05774500	3.87214500	-3.16528800
H	-0.15802100	4.92812600	-2.88211200
H	-0.02015400	3.82643300	-4.26374100
H	0.89139900	3.47373000	-2.77935800
C	-1.02587600	1.60136600	-3.19648600
H	-1.80280700	0.90447300	-2.86330300
H	-0.04925900	1.21864900	-2.87030000
H	-1.02671900	1.61671700	-4.29630900
C	-1.06108900	3.86830300	2.26506100
C	-2.53241600	4.02464100	2.66495900
H	-3.07172600	3.07328600	2.55059800
H	-3.02883800	4.78419700	2.04271500
H	-2.61293200	4.34146100	3.71592200
C	-0.38667400	2.82064200	3.15215800
H	-0.41640600	3.13879400	4.20437700
H	0.66060100	2.66972700	2.85398700
H	-0.89857000	1.85113600	3.07377000
C	-0.33184400	5.20081000	2.43813200
H	-0.29878100	5.45278900	3.50838900
H	-0.83273100	6.02603700	1.91611100
H	0.70291500	5.12857000	2.07430900
C	0.68654400	-0.36322800	0.44808000
H	-0.01288200	-1.01036700	-0.10708400
C	0.88481200	-0.94140000	1.84174400
H	1.53481300	-0.26722800	2.42267400
H	1.44543400	-1.88339400	1.72525800
C	-0.38823100	-1.22412800	2.62082200
H	-0.91200200	-0.29444100	2.87875300
H	-1.07908200	-1.84157100	2.02976800
H	-0.16765900	-1.74765500	3.56464200
C	-2.97432800	1.05040500	0.47247300
O	-2.29980300	0.29924100	-0.60678000
H	-3.81012300	1.63037600	0.04552700
C	-3.39487600	0.26285800	1.69953400
H	-3.03754300	-0.76846300	1.57101200
H	-2.88461700	0.65124500	2.59906400
C	-4.89394200	0.24825200	1.95179000
H	-5.25585000	1.27804400	2.13352700
H	-5.38549100	-0.10961100	1.03574400
H	-0.60849200	-3.44091200	-1.42070000
C	-4.18464100	-1.03275800	-2.95541100
C	-4.98192800	-1.58871900	-1.71635700
O	-4.10909900	-1.32335100	-0.64029700
O	-2.83865900	-1.17501800	-2.55500300
C	-6.31693200	-0.89786700	-1.46717000

H	-6.99321600	-1.01072500	-2.32882700
H	-6.80757600	-1.35114000	-0.59224700
H	-6.18667900	0.17228500	-1.26127000
C	-5.23023500	-3.09468800	-1.79894900
H	-4.30706400	-3.64245400	-2.02939600
H	-5.59083900	-3.44390400	-0.82046300
H	-5.98426400	-3.34790100	-2.55915500
C	-4.48733400	0.44378700	-3.20719000
H	-3.79685700	0.82580200	-3.97174700
H	-5.51529600	0.60209600	-3.56491700
H	-4.33955900	1.03118000	-2.28991200
C	-4.39412900	-1.81057200	-4.24564300
H	-5.45064200	-1.80137600	-4.55567700
H	-3.79701900	-1.35314900	-5.04879900
H	-4.06558100	-2.85218500	-4.13722200
B	-2.75822800	-1.14454300	-1.12260800
C	-1.58699500	-3.30899100	-0.92844200
H	-2.34624600	-3.60658600	-1.67347300
O	-1.74057800	-1.97187200	-0.55658100
C	-1.65263500	-4.21539900	0.28962500
H	-1.37602200	-5.24190000	-0.00893800
H	-0.86771100	-3.88478200	0.99262200
C	-3.00075000	-4.20505300	0.99382800
H	-3.36649000	-3.16536000	1.04089300
H	-3.73999300	-4.74548000	0.37637100
C	-5.28141700	-0.64555700	3.11762300
H	-4.78070000	-0.29944700	4.04129400
H	-4.88518400	-1.65864900	2.92588100
C	-6.78117500	-0.73724700	3.35820400
H	-7.27152000	-1.07659300	2.42847800
H	-7.18274200	0.27310600	3.55477200
C	-7.15101800	-1.66891900	4.50150200
H	-8.24027700	-1.72222300	4.65139300
H	-6.79037500	-2.69301700	4.31178300
H	-6.70015600	-1.33539200	5.45036000
C	-2.97073300	-4.80536700	2.39076400
H	-2.56153100	-5.83249100	2.35095500
H	-2.26367100	-4.22843800	3.01604100
C	-4.33292700	-4.83045800	3.06964600
H	-4.74522000	-3.80638200	3.07391900
H	-5.03163600	-5.42749600	2.45705700
C	-4.30227600	-5.37154100	4.48947800
H	-5.30256300	-5.36828500	4.94986800
H	-3.92713900	-6.40768200	4.51605700
H	-3.64006300	-4.76740700	5.13092000

Cartesian coordinates of the optimized geometry of Int-**4b** at PBE0-D3BJ/def2-SVP level of theory:

Ga	2.17361200	-1.57349600	-0.60514800
Cl	3.41930200	-0.70009200	-2.23901000
Si	0.14956100	2.10996300	0.16862900
O	0.76360800	0.61903000	0.48823900
N	3.53342000	-1.96198000	0.77086100
N	1.98064000	-3.44632400	-1.17656400
N	1.15479300	3.26301900	-0.81212500
N	1.20000700	3.15036600	1.33267600
C	4.43566500	-2.90186200	0.47120200
C	4.27173500	-3.84762300	-0.55306800
H	5.12446200	-4.49956000	-0.73512300
C	3.09894700	-4.16713300	-1.25616900
C	5.71658900	-2.96287400	1.25459200
H	6.27919900	-3.87325300	1.01852100
H	5.53218100	-2.92020200	2.33645200
H	6.34129700	-2.08979000	1.01094200
C	3.14178800	-5.36598000	-2.15914600
H	2.19383300	-5.91826200	-2.16166400
H	3.96226700	-6.04201900	-1.89095400

H	3.30856500	-5.01284300	-3.19020100
C	3.73691200	-1.16699500	1.94295100
C	4.55144600	-0.01716800	1.88677200
C	4.78538700	0.69167100	3.06900600
H	5.42435400	1.57752700	3.04117500
C	4.23244400	0.28558300	4.27710500
H	4.43306300	0.84962100	5.19100200
C	3.41272800	-0.83594100	4.31383800
H	2.96933100	-1.14685000	5.26187100
C	3.14681000	-1.57685200	3.15846400
C	5.14586200	0.48109700	0.58613300
H	5.02919700	-0.30801800	-0.16976100
C	6.63248400	0.81277400	0.69167800
H	6.81853400	1.67680300	1.34935300
H	7.03475700	1.06586400	-0.30112400
H	7.21579300	-0.03297500	1.08707500
C	4.35020500	1.68493800	0.08778800
H	3.28272400	1.44761200	-0.01253400
H	4.71777700	2.01751800	-0.89479900
H	4.44188200	2.51789100	0.80270500
C	2.27184000	-2.81172700	3.23788000
H	1.91849500	-3.01039300	2.21575800
C	3.06147100	-4.04548200	3.67764700
H	3.51591300	-3.89082500	4.66935100
H	3.86568800	-4.29071800	2.96970200
H	2.39875100	-4.92281500	3.74264400
C	1.04756100	-2.60320000	4.12582800
H	0.50769200	-1.68221300	3.86157300
H	1.31906900	-2.54184600	5.19108900
H	0.34902700	-3.44592400	4.01873700
C	0.74930200	-3.97758700	-1.67170800
C	0.32686900	-3.69808200	-2.98609300
C	-0.89322600	-4.23036700	-3.41802600
H	-1.23826700	-4.01369500	-4.43179000
C	-1.65967400	-5.04056900	-2.59106100
H	-2.60217300	-5.45900700	-2.95212300
C	-1.22525700	-5.31388100	-1.29714400
H	-1.83392000	-5.94806400	-0.64927100
C	-0.03161500	-4.77965600	-0.80646500
C	1.15689500	-2.86218700	-3.93753600
H	2.10813100	-2.62922400	-3.43926600
C	1.48430300	-3.61993400	-5.22430100
H	0.58007700	-3.82086500	-5.82055700
H	1.96612100	-4.58817200	-5.01939600
H	2.16644100	-3.02576300	-5.85173900
C	0.47447100	-1.53197600	-4.24582900
H	-0.45305400	-1.68509300	-4.81916200
H	1.14139000	-0.88692000	-4.83795100
H	0.19493800	-0.97818900	-3.33983200
C	0.41569200	-5.08414400	0.61215100
H	1.10950300	-4.28049000	0.90080700
C	1.19133000	-6.40072100	0.69415200
H	0.56858300	-7.24337600	0.35341100
H	1.49497500	-6.60347000	1.73314900
H	2.10218400	-6.38467400	0.08113200
C	-0.73679700	-5.08857100	1.61312900
H	-1.33360900	-4.16697700	1.56091900
H	-0.34648800	-5.18529800	2.63744300
H	-1.41749800	-5.93930600	1.45413300
C	1.58716200	3.88091500	0.29811600
C	2.23122300	5.21649300	0.34768600
C	1.39415400	6.33695100	0.34591700
H	0.31046500	6.20364800	0.30930000
C	1.94258800	7.61705500	0.38687900
H	1.28406200	8.48832300	0.38545100
C	3.32569000	7.78232300	0.42637900
H	3.75581400	8.78592700	0.45590500
C	4.16006900	6.66449800	0.42632800
H	5.24459400	6.79033800	0.45386200

C	3.61754000	5.38381800	0.38824300
H	4.26951500	4.51018100	0.37983600
C	1.29376300	3.57550300	-2.24358800
C	0.08853900	4.40535500	-2.69159200
H	-0.85432400	3.85864600	-2.53866000
H	0.17296300	4.64621400	-3.76224500
H	0.04713000	5.35732000	-2.13969000
C	2.58834800	4.32890500	-2.54489400
H	2.58122500	5.36065100	-2.17100300
H	2.71519500	4.37152000	-3.63619600
H	3.45948000	3.80395500	-2.12631000
C	1.33821700	2.22891500	-2.96361300
H	0.40840900	1.66113800	-2.81946000
H	2.18498500	1.62502600	-2.60905500
H	1.46172000	2.38591900	-4.04436100
C	1.27144500	3.37432400	2.77857900
C	0.16264500	4.33999700	3.20761700
H	-0.82512600	3.97374500	2.89207300
H	0.32006600	5.33626200	2.76831800
H	0.15351600	4.45058600	4.30246100
C	1.06769300	2.00404600	3.42428200
H	1.10115600	2.08956400	4.51959800
H	1.84480900	1.29960300	3.09930200
H	0.09509100	1.57479800	3.14680100
C	2.63422500	3.91547300	3.21023700
H	2.68937100	3.91067600	4.30872500
H	2.80186900	4.94578100	2.87206300
H	3.44231300	3.27493500	2.82952500
C	0.48013300	-0.64612800	-0.11574500
H	-0.11341900	-0.47636100	-1.02902800
C	-0.30862800	-1.55630900	0.82250600
H	0.36373000	-2.07650100	1.52030300
H	-0.75227000	-2.34506600	0.19308900
C	-1.38453000	-0.86271200	1.63040500
H	-0.92861600	-0.22070100	2.39677200
H	-2.02987100	-0.23874000	0.99970700
H	-2.02854600	-1.59069900	2.14558200
C	-1.58785400	2.72049500	0.08881000
O	-1.27748800	1.71151300	-0.94668600
H	-1.64718700	3.70093800	-0.41180200
H	-2.21055900	-1.27159700	-1.50599000
C	-2.40419300	1.25220300	-4.30988500
C	-3.44054000	2.32081500	-3.74267500
O	-2.96651600	2.56018600	-2.43180400
O	-1.80179500	0.70365600	-3.14426200
C	-3.45786700	3.63950100	-4.50196400
H	-3.76852700	3.49507200	-5.54765800
H	-4.17756900	4.32168400	-4.02540700
H	-2.47507900	4.12706800	-4.49222300
C	-4.87309200	1.78656900	-3.65822500
H	-4.92115500	0.86133900	-3.07084100
H	-5.49209600	2.53448100	-3.14045000
H	-5.31020300	1.60969500	-4.65160600
C	-1.31333100	1.87736800	-5.17225800
H	-0.54134900	1.12020100	-5.37149500
H	-1.70943700	2.22930700	-6.13609200
H	-0.83498600	2.72082100	-4.66365200
C	-3.06158700	0.13165500	-5.10822300
H	-3.58780700	0.52492700	-5.99135100
H	-2.28950300	-0.56876400	-5.45835600
H	-3.77851400	-0.43440700	-4.50024100
B	-2.38263700	1.31890900	-1.99074900
C	-3.24871700	-0.89679600	-1.41824400
H	-3.74239700	-1.22739600	-2.35760900
O	-3.30893400	0.47490000	-1.26698600
C	-2.81977100	2.53321400	0.91889500
C	-2.78801900	2.02432800	2.22083200
C	-4.05329400	2.94074400	0.38487300
C	-3.95300700	1.87361800	2.96587700

H	-1.83808700	1.72879400	2.66291200
C	-5.22400600	2.81731000	1.11950600
H	-4.07754500	3.31193000	-0.64145400
C	-5.15657500	2.27101300	2.39799600
H	-3.94707600	1.45221000	3.97086500
H	-6.19162600	3.11917700	0.71874900
C	-3.91311700	-1.60462800	-0.26967800
C	-4.68425200	-0.90051900	0.65972400
C	-3.72869700	-2.98259900	-0.10308200
C	-5.23771500	-1.54768200	1.75960100
H	-4.80468100	0.17388600	0.52074400
C	-4.27897500	-3.64627800	0.98413500
H	-3.11898100	-3.53479300	-0.82217700
C	-5.02131100	-2.91460100	1.90887600
H	-5.81463100	-1.01445100	2.51682200
H	-4.13485700	-4.71437100	1.14627000
N	-5.57476300	-3.60580000	3.07497100
O	-6.22490300	-2.95221900	3.86045900
O	-5.34476400	-4.79257400	3.18497500
N	-6.39540000	2.09872500	3.16408900
O	-7.39383400	2.63671700	2.74074900
O	-6.33955100	1.42472300	4.17051500

Cartesian coordinates of the optimized geometry of Int-**5a** at PBE0-D3BJ/def2-SVP level of theory:

Ga	2.96354900	0.44452100	-0.49971000
Cl	2.38786900	1.62113700	-2.30710200
Si	-1.21730800	0.63318300	-0.07884000
O	0.35523200	0.63946400	0.39536600
N	4.18340300	1.63497400	0.45750300
N	4.34010900	-0.77546600	-1.16444600
N	-1.64961000	2.00997300	-1.19926900
N	-2.06581400	1.88820400	0.91252100
C	5.36816000	1.86770700	-0.10610600
C	5.93595500	1.03534900	-1.08749200
H	6.89007800	1.37032300	-1.49183200
C	5.51576300	-0.24102000	-1.50054300
C	6.14297000	3.09286100	0.28534000
H	5.81197800	3.92808800	-0.35354300
H	7.21965400	2.95384600	0.12924600
H	5.95373100	3.38672500	1.32533800
C	6.44704600	-1.02739800	-2.37706900
H	6.03177200	-1.07913100	-3.39559100
H	6.54722500	-2.06458600	-2.02916200
H	7.43793700	-0.56149700	-2.42500200
C	3.74208300	2.34903400	1.61670900
C	3.00143700	3.54024300	1.48841600
C	2.53167000	4.15940400	2.65229700
H	1.95124500	5.08114700	2.56595400
C	2.78687000	3.62745800	3.90791500
H	2.40706700	4.12448200	4.80351800
C	3.53018300	2.45723400	4.02082400
H	3.73083500	2.04503400	5.01104000
C	4.01811100	1.79789800	2.89057700
C	2.69751700	4.17518800	0.14750000
H	3.16783200	3.56383900	-0.63552000
C	3.26940600	5.58980900	0.04980100
H	2.77951400	6.27523900	0.75959700
H	3.11393900	5.99575900	-0.96177200
H	4.34844800	5.61174700	0.26424700
C	1.19583900	4.17070100	-0.12883400
H	0.78803800	3.15142100	-0.09215500
H	0.98834200	4.57983100	-1.12860600
H	0.65706600	4.78362600	0.61186200
C	4.85099500	0.53873200	3.03995000
H	4.69154700	-0.05444500	2.12685900
C	6.34605000	0.85893000	3.11041100

H	6.56422000	1.51014800	3.97185200
H	6.70464600	1.36613800	2.20541300
H	6.93334000	-0.06544000	3.22617200
C	4.44398500	-0.31888300	4.23398200
H	3.36713300	-0.54110000	4.23382000
H	4.69021800	0.16737800	5.19059100
H	4.99038100	-1.27354200	4.21559400
C	4.06683400	-2.15253600	-1.44481500
C	3.52031500	-2.54198200	-2.68478600
C	3.31194800	-3.90524100	-2.92088400
H	2.88994200	-4.22013800	-3.87824700
C	3.62698500	-4.86150800	-1.96577700
H	3.46186500	-5.92088500	-2.17295400
C	4.12789400	-4.46088200	-0.73237700
H	4.34837600	-5.21403200	0.02614400
C	4.34550300	-3.11151400	-0.44421700
C	3.10832300	-1.54005100	-3.74270000
H	3.44873800	-0.54464500	-3.42375800
C	3.72705000	-1.83186800	-5.10867700
H	3.36098400	-2.78070600	-5.53137500
H	4.82476500	-1.89633400	-5.05728100
H	3.46812200	-1.03400400	-5.82136000
C	1.58316200	-1.48052200	-3.83531600
H	1.16690000	-2.43628400	-4.19229800
H	1.26515100	-0.68605500	-4.52648100
H	1.13342600	-1.26651400	-2.85514300
C	4.87838000	-2.70077500	0.91409500
H	4.54834900	-1.66336400	1.07496100
C	6.40701500	-2.68863800	0.95679400
H	6.81353400	-3.68485900	0.72011500
H	6.76286600	-2.40757600	1.96058100
H	6.83206600	-1.96929500	0.24315700
C	4.31147400	-3.55103300	2.04786900
H	3.21472300	-3.62073200	1.99514700
H	4.57767600	-3.11486800	3.02156100
H	4.71439400	-4.57548500	2.03389300
C	-2.29316200	2.60699000	-0.18745800
C	-3.13597400	3.81665000	-0.28262900
C	-4.52844300	3.66791700	-0.27990000
H	-4.96864100	2.66256300	-0.21914400
C	-5.32582600	4.80343100	-0.39422900
H	-6.41278800	4.69309100	-0.40436300
C	-4.74915600	6.06997700	-0.49681300
H	-5.38456400	6.95491300	-0.58186500
C	-3.36206400	6.20882000	-0.49352800
H	-2.90732400	7.19877100	-0.57271200
C	-2.55027600	5.08053900	-0.39358100
H	-1.46315800	5.17965200	-0.39419900
C	-1.83002200	2.16730800	-2.65427400
C	-3.30674800	2.30687600	-3.02985900
H	-3.90723100	1.56605800	-2.48140400
H	-3.42261500	2.13463500	-4.11041800
H	-3.69567400	3.30983900	-2.81056700
C	-1.01674800	3.36562500	-3.14283300
H	-1.37468500	4.29954500	-2.68666400
H	-1.12111200	3.46353100	-4.23396300
H	0.04809100	3.23167600	-2.90448400
C	-1.27706600	0.88869700	-3.27496200
H	-1.91264400	0.03590500	-3.00037000
H	-0.24126200	0.71499700	-2.94543900
H	-1.26918300	0.97156700	-4.37110300
C	-2.28335400	2.19001500	2.33645300
C	-3.75936500	2.44589300	2.64058400
H	-4.36706800	1.62273500	2.23579400
H	-4.10451700	3.40099900	2.22438000
H	-3.89554200	2.48950100	3.73193000
C	-1.82960000	0.94632500	3.09491700
H	-1.97024300	1.09131600	4.17525100
H	-0.76340800	0.74848400	2.91309200

H	-2.43546100	0.08534000	2.77448700
C	-1.41490100	3.38615700	2.73130200
H	-1.52071600	3.58705500	3.80810900
H	-1.71978100	4.29357600	2.18879000
H	-0.35387500	3.18601700	2.51831000
C	1.39290200	-0.33786900	0.41648100
H	1.09457500	-1.21422000	-0.18723300
C	1.65474000	-0.79065400	1.84558500
H	1.73195200	0.11465500	2.47080700
H	2.64627600	-1.26769200	1.88781300
C	0.62495100	-1.75597600	2.41597800
H	-0.40141100	-1.37668400	2.30846200
H	0.66807700	-2.72984700	1.90541900
H	0.79677200	-1.93343600	3.48883100
C	-2.24383800	-0.90943400	-0.33279100
O	-3.37793400	-0.21105500	-0.74501200
H	-2.39038300	-1.29772400	0.69569300
B	-4.66531400	-0.43703200	-0.01443200
O	-5.50796800	0.70060300	-0.40192900
O	-5.38371000	-1.61768600	-0.46619800
C	-6.73273100	0.22200900	-0.90364400
C	-6.35387900	-1.21979900	-1.40314400
C	-7.75322300	0.18919300	0.23582000
H	-7.79972100	1.18977500	0.69163900
H	-8.76270600	-0.08455200	-0.10740100
H	-7.44229100	-0.51928600	1.01633300
C	-7.22976800	1.15633800	-1.99815600
H	-8.13299300	0.75838900	-2.48696300
H	-7.48664700	2.13613700	-1.56508000
H	-6.45824900	1.31912800	-2.76219100
C	-7.50373600	-2.21544600	-1.38287900
H	-8.33379100	-1.88712900	-2.02821500
H	-7.15129100	-3.19139900	-1.74992100
H	-7.88311000	-2.35973300	-0.36263400
C	-5.72370100	-1.19695200	-2.79864800
H	-4.90624100	-0.46461300	-2.84156000
H	-5.29358700	-2.18988700	-2.99972800
H	-6.45591100	-0.97059600	-3.58879500
C	-4.89002900	-1.50840800	2.21539100
O	-4.34379500	-0.51737900	1.40695800
C	-1.90878700	-2.09261100	-1.23618300
H	-2.89162300	-2.53972300	-1.46262500
H	-1.49503500	-1.76218100	-2.20270700
C	-1.00548200	-3.14412700	-0.61711700
H	-0.03237600	-2.70184200	-0.34629400
H	-1.44950900	-3.48998400	0.33332500
C	-0.75224500	-4.33875500	-1.52234900
H	-0.29190800	-3.99083600	-2.46541700
H	-1.71823300	-4.79279500	-1.80852000
C	0.14234900	-5.39417700	-0.88865600
H	-0.31529700	-5.73555300	0.05701700
H	1.10396000	-4.92815500	-0.60986900
C	0.40016800	-6.58815900	-1.79246300
H	0.87367400	-6.27493200	-2.73724300
H	1.06307600	-7.32695800	-1.31582200
H	-0.53829200	-7.10351300	-2.05313000
C	-4.08988200	-2.80276700	2.16689900
H	-4.06454700	-3.13831700	1.11667500
H	-3.04520600	-2.58764900	2.46139400
C	-4.64248600	-3.91251900	3.04489000
H	-4.70186200	-3.56708300	4.09384600
H	-5.68233300	-4.13319300	2.74264500
C	-3.81931100	-5.19120700	2.98235100
H	-2.77859600	-4.96922100	3.28434300
H	-3.75588600	-5.52859200	1.93134600
C	-4.36037700	-6.32430000	3.84264000
H	-4.42487200	-5.98355100	4.89149900
H	-5.39892000	-6.54440800	3.53790100
C	-3.52567400	-7.59277100	3.76721400

H	-2.49084900	-7.40988500	4.09999100
H	-3.94043300	-8.39454500	4.39729900
H	-3.47358200	-7.97455400	2.73459900
H	-4.91433200	-1.13223100	3.25734800
H	-5.93421900	-1.73402600	1.92937800

Cartesian coordinates of the optimized geometry of Int-**5b** at PBE0-D3BJ/def2-SVP level of theory:

Ga	-2.89846100	0.74768600	0.64793300
Cl	-2.20421600	1.49112100	2.63227300
Si	1.31489900	0.78568400	0.00220900
O	-0.27308500	0.95273500	-0.29110000
N	-4.14410000	2.12655900	0.05927100
N	-4.24904200	-0.58333200	1.11359600
N	1.91577000	1.76908200	1.40478600
N	2.23893400	2.18020900	-0.68618100
C	-5.27915700	2.24422900	0.75259200
C	-5.81087700	1.21138300	1.53927100
H	-6.73194900	1.44995800	2.06920200
C	-5.40121900	-0.13573400	1.60978200
C	-6.01615100	3.55215400	0.73118100
H	-5.57672900	4.20382700	1.50467700
H	-7.08023700	3.42123800	0.96214600
H	-5.90667500	4.07267700	-0.22856700
C	-6.32598000	-1.10146900	2.29123000
H	-5.90563800	-1.38513300	3.26821000
H	-6.42771200	-2.03096300	1.71447300
H	-7.31578800	-0.65853400	2.45047000
C	-3.78257600	3.05054000	-0.96910200
C	-2.92605100	4.13479800	-0.69334700
C	-2.52956900	4.95645500	-1.75469100
H	-1.86106100	5.79760400	-1.55450700
C	-2.97442100	4.72848800	-3.04951700
H	-2.65433100	5.38208800	-3.86432800
C	-3.83320800	3.66419700	-3.30487000
H	-4.18136800	3.49105000	-4.32472900
C	-4.24780400	2.80665600	-2.28362500
C	-2.43639300	4.44938100	0.70520400
H	-2.87535900	3.71238100	1.39198300
C	-2.88993100	5.83722200	1.15842900
H	-2.43152500	6.63368700	0.55067200
H	-2.60031400	6.01140500	2.20636500
H	-3.98164500	5.95287500	1.08296800
C	-0.91925000	4.30306100	0.80985700
H	-0.59558000	3.29792500	0.50598200
H	-0.59103600	4.46282200	1.84811100
H	-0.40313400	5.03871200	0.17146900
C	-5.18744000	1.65441200	-2.58950100
H	-5.01449200	0.89000700	-1.81636200
C	-6.65448400	2.07734500	-2.48132600
H	-6.87970800	2.88734800	-3.19345300
H	-6.91049600	2.43314400	-1.47515500
H	-7.31732200	1.22864400	-2.71115900
C	-4.93073100	1.01447100	-3.95125700
H	-3.87448100	0.73985500	-4.08634000
H	-5.21349700	1.68396800	-4.77829400
H	-5.53721200	0.10293200	-4.05997900
C	-3.96828600	-1.98907000	1.07853800
C	-3.44252300	-2.64782700	2.20961000
C	-3.23321200	-4.02868400	2.13393600
H	-2.82841100	-4.55704300	3.00058500
C	-3.50810700	-4.74111800	0.97457400
H	-3.31625500	-5.81468500	0.93874400
C	-3.97716900	-4.07016200	-0.14896900
H	-4.15567900	-4.62874300	-1.06907200
C	-4.21301700	-2.69296400	-0.12226100
C	-3.03416000	-1.91310800	3.46998700

H	-3.35087200	-0.86422900	3.37568800
C	-3.67532100	-2.49281900	4.72990900
H	-3.33115900	-3.52034400	4.92500600
H	-4.77355000	-2.52155400	4.66104500
H	-3.40740800	-1.88491800	5.60765600
C	-1.50911700	-1.91286200	3.59306200
H	-1.12194000	-2.92847500	3.77179700
H	-1.18744500	-1.27272000	4.42818700
H	-1.03504700	-1.53254300	2.67705100
C	-4.73422900	-1.98425700	-1.35678800
H	-4.34176600	-0.95636500	-1.31366000
C	-6.25993100	-1.87154900	-1.35052800
H	-6.72320800	-2.86939200	-1.29637400
H	-6.61545600	-1.38440100	-2.27222400
H	-6.62718000	-1.27797000	-0.50180000
C	-4.24679600	-2.61538300	-2.65803100
H	-3.16534500	-2.81448000	-2.65022900
H	-4.46535900	-1.95199000	-3.50760000
H	-4.75262600	-3.57210800	-2.86059000
C	2.57613100	2.55963800	0.54932600
C	3.52085100	3.63774200	0.90651000
C	4.89436400	3.37195900	0.87187500
H	5.24452700	2.37494600	0.57141800
C	5.78375700	4.38510800	1.22119100
H	6.85732300	4.18322800	1.20369100
C	5.31430300	5.64679400	1.58719700
H	6.02063000	6.43592500	1.85591000
C	3.94416600	5.90435800	1.61020300
H	3.57424000	6.89203200	1.89391800
C	3.04111100	4.89793600	1.27467600
H	1.96586300	5.08858300	1.29549500
C	2.09691100	1.56196600	2.85336000
C	3.56152300	1.26751200	3.18061100
H	3.95521800	0.51567500	2.48175600
H	3.63946100	0.88689200	4.20976900
H	4.18572900	2.16829500	3.10813200
C	1.58282300	2.77877100	3.62214900
H	2.17234200	3.67853200	3.39948100
H	1.65957200	2.58675000	4.70260300
H	0.52641800	2.96511800	3.37995700
C	1.23376900	0.35550000	3.20214600
H	1.58656400	-0.54657400	2.68381600
H	0.18269000	0.54157600	2.94037700
H	1.28151200	0.15468300	4.28165000
C	2.50501100	2.78750100	-2.00102600
C	4.00393800	2.94262700	-2.25866200
H	4.52194900	1.99033300	-2.07239800
H	4.44704700	3.72867600	-1.63387600
H	4.15518000	3.22977900	-3.31027200
C	1.91912300	1.82016100	-3.02448800
H	2.07367600	2.20827700	-4.04107600
H	0.83720600	1.69867800	-2.87003600
H	2.42520400	0.84659100	-2.94301000
C	1.78275000	4.13318000	-2.08804500
H	1.92810300	4.57030700	-3.08723100
H	2.17855200	4.84553700	-1.34948300
H	0.70257800	4.00957900	-1.91728400
C	-1.43886100	0.17314200	-0.56790900
H	-1.24146600	-0.88111700	-0.31360300
C	-1.77830000	0.28074900	-2.04681100
H	-1.74279800	1.34752600	-2.32297000
H	-2.82467000	-0.02565000	-2.19352800
C	-0.89439200	-0.55023100	-2.96389900
H	0.17500800	-0.38999000	-2.76638900
H	-1.09529900	-1.62661800	-2.85486200
H	-1.06611600	-0.29963600	-4.02223800
C	2.25510800	-0.83601800	-0.22331400
O	3.48974400	-0.47376100	0.27052900
H	2.28027600	-0.98936800	-1.32170700

C	1.66045800	-2.06396900	0.40952500
C	2.24744500	-2.60998700	1.55557200
C	0.52905300	-2.68446600	-0.13755300
C	1.67381900	-3.70789200	2.18614700
H	3.16581000	-2.15737600	1.93034900
C	-0.03654800	-3.80369300	0.45648100
H	0.10347900	-2.30004400	-1.06542800
C	0.53540100	-4.28378500	1.63199800
H	2.09317900	-4.12831600	3.10051000
H	-0.90541400	-4.30694000	0.03209100
N	-0.08586400	-5.41348000	2.31354300
O	0.15323000	-5.55386300	3.49631900
O	-0.81427200	-6.13344500	1.66579300
B	4.72243200	-0.56868400	-0.57327800
O	5.62122900	0.45396300	-0.04215700
O	5.43878500	-1.82511300	-0.42425700
C	6.89700800	-0.11254700	0.14770800
C	6.55316000	-1.62062700	0.41458700
C	7.72046300	0.07605300	-1.12758500
H	7.72119400	1.14530700	-1.38691800
H	8.76345100	-0.25426600	-1.00814500
H	7.27355400	-0.47119600	-1.96922500
C	7.59225300	0.58741400	1.30666700
H	8.53643900	0.08583000	1.57001700
H	7.83298800	1.62525900	1.02712500
H	6.94845000	0.61774200	2.19574500
C	7.65390900	-2.59746500	0.03306300
H	8.57939900	-2.39878400	0.59574900
H	7.33282500	-3.62534400	0.26042300
H	7.87656500	-2.54530300	-1.04092600
C	6.13319000	-1.86292700	1.86556600
H	5.37961800	-1.12631300	2.17631100
H	5.68268800	-2.86434200	1.93425300
H	6.98245600	-1.81660200	2.56361300
H	5.60255100	-1.76311500	-2.75721800
C	4.65993700	-1.23719400	-2.98322700
O	4.31787300	-0.34824300	-1.96840500
H	4.79512500	-0.67255700	-3.92317700
C	3.55120600	-2.24928200	-3.16204200
C	2.51644200	-2.01847000	-4.07695000
C	3.46313100	-3.34949400	-2.29616900
C	1.37879100	-2.81685900	-4.08625800
H	2.58916400	-1.17434000	-4.76777400
C	2.34136200	-4.16811200	-2.30292000
H	4.26266200	-3.50282000	-1.56702800
C	1.29804500	-3.86694700	-3.17640800
H	0.54326800	-2.62767500	-4.75966700
H	2.23279100	-5.00328900	-1.61091000
N	0.04694700	-4.61266000	-3.06977200
O	0.03245100	-5.60206100	-2.37165500
O	-0.92109000	-4.17462700	-3.66400000

Cartesian coordinates of the optimized geometry of Int-**6** at PBE0-D3BJ/def2-SVP level of theory:

Ga	-1.81454900	-0.69431200	-0.52666600
Cl	-0.95990400	-0.74543700	-2.59676100
Si	2.07308200	0.75907700	0.52483600
O	0.67902500	-0.17501200	0.58005100
N	-2.41488400	-2.55055400	-0.27478800
N	-3.62375600	0.02995600	-0.88531300
N	3.19364600	0.52809800	-0.93268800
N	3.13911200	-1.03864400	0.55170600
C	-3.47301800	-2.99123000	-0.95248500
C	-4.41121200	-2.14734500	-1.56651800
H	-5.21375100	-2.64942000	-2.10530100
C	-4.53435400	-0.75174000	-1.45959700
C	-3.69422500	-4.47071200	-1.09828300

H	-3.08878600	-4.83751800	-1.94226100
H	-4.74634600	-4.69458500	-1.31197000
H	-3.37271700	-5.02577600	-0.20784800
C	-5.77362800	-0.14295100	-2.05472900
H	-6.07714000	0.77340300	-1.53318300
H	-6.60342800	-0.86037700	-2.05130800
H	-5.56874000	0.13073600	-3.10175100
C	-1.60695600	-3.45092000	0.48480300
C	-0.55171700	-4.15705500	-0.12688300
C	0.22185400	-5.00819900	0.66944400
H	1.04229100	-5.56591300	0.21154100
C	-0.02834400	-5.15230600	2.02722000
H	0.59498300	-5.81452100	2.63256300
C	-1.07123500	-4.44525800	2.61745200
H	-1.25895800	-4.56132900	3.68613900
C	-1.87892500	-3.58883100	1.86576200
C	-0.23064900	-4.01405800	-1.60159600
H	-0.99546800	-3.36530800	-2.05043100
C	-0.25475800	-5.35698900	-2.33106300
H	0.54619400	-6.02519000	-1.97623600
H	-0.10277700	-5.20732200	-3.41134000
H	-1.20858500	-5.88771400	-2.19150500
C	1.11143900	-3.31176600	-1.80978000
H	1.18571600	-2.39345400	-1.20943800
H	1.23664100	-3.03530000	-2.86775300
H	1.94288600	-3.97841200	-1.53000300
C	-3.04150800	-2.85281900	2.50848200
H	-3.14283700	-1.89757400	1.97072200
C	-4.36161400	-3.60661200	2.32916200
H	-4.30134500	-4.61178200	2.77628700
H	-4.63549200	-3.72001900	1.27227800
H	-5.18238700	-3.06385200	2.82443700
C	-2.82150300	-2.53526700	3.98406500
H	-1.85174200	-2.04911900	4.16060900
H	-2.86802700	-3.44164500	4.60833100
H	-3.61098100	-1.85791200	4.34404000
C	-3.87650400	1.42100400	-0.68321800
C	-3.65795300	2.35420400	-1.71845100
C	-3.90929700	3.70527100	-1.45422100
H	-3.75251100	4.43865500	-2.24916900
C	-4.35136600	4.13072600	-0.20852500
H	-4.53904200	5.19138500	-0.02482000
C	-4.55528700	3.19986400	0.80497000
H	-4.90355600	3.53826300	1.78246000
C	-4.32833300	1.83865500	0.58972000
C	-3.16198200	1.95169200	-3.09373400
H	-3.08227500	0.85562900	-3.11696500
C	-4.12059600	2.39041100	-4.20132600
H	-4.16484100	3.48788200	-4.28710400
H	-5.14705100	2.03481900	-4.02750200
H	-3.78394600	1.99948600	-5.17400900
C	-1.75821600	2.49662100	-3.35395400
H	-1.76582100	3.59538500	-3.43584800
H	-1.35761700	2.08709900	-4.29350400
H	-1.06606100	2.21451700	-2.54987600
C	-4.61296000	0.82764800	1.68365300
H	-3.93689500	-0.02345200	1.50853600
C	-6.03884500	0.28147000	1.58525000
H	-6.77656300	1.09340700	1.68768700
H	-6.22623800	-0.45189100	2.38556800
H	-6.22065600	-0.22220400	0.62626100
C	-4.34031500	1.36152900	3.08631400
H	-3.34679200	1.82714600	3.16092700
H	-4.38526300	0.54091800	3.81842100
H	-5.08998700	2.10779200	3.39315700
C	3.76684000	-0.63069600	-0.50905100
C	4.98348300	-1.23208700	-1.11653700
C	6.22209600	-0.61409200	-0.91576000
H	6.27616400	0.29557500	-0.31354700

C	7.37631200	-1.15666700	-1.47664500
H	8.34037700	-0.67068200	-1.30987600
C	7.29950900	-2.31319500	-2.25098400
H	8.20347200	-2.73509000	-2.69645900
C	6.06523300	-2.92993400	-2.45618400
H	5.99920400	-3.83387000	-3.06606200
C	4.91241800	-2.39631800	-1.88762100
H	3.94376100	-2.86943000	-2.05603500
C	3.31705500	1.21840400	-2.22936400
C	3.72464200	0.27388800	-3.36381700
H	4.76506200	-0.06425900	-3.28914200
H	3.61881400	0.80838600	-4.31947400
H	3.06554200	-0.60692000	-3.39655600
C	1.94081300	1.77892700	-2.60236500
H	1.18296500	0.98194500	-2.62330800
H	1.98586600	2.24632300	-3.59749100
H	1.61394500	2.55277300	-1.89708400
C	4.33191400	2.35866400	-2.10787100
H	5.33291500	1.96287400	-1.87930000
H	4.03176500	3.05036500	-1.30790200
H	4.39825300	2.92214500	-3.05215300
C	3.48580200	-2.05149700	1.55059300
C	4.77637700	-1.67732700	2.28466600
H	4.66851700	-0.70105100	2.77163300
H	5.63301700	-1.64039400	1.59535200
H	4.99988300	-2.42643400	3.06001400
C	2.32199000	-2.08935200	2.54347800
H	2.51751200	-2.83768300	3.32635700
H	1.38857100	-2.35226900	2.02721900
H	2.19252100	-1.10513500	3.01224900
C	3.64408700	-3.44775300	0.94348400
H	3.74116000	-4.18614700	1.75395900
H	4.53732300	-3.52805000	0.31056800
H	2.75848200	-3.70927700	0.35266400
C	-0.66163600	0.22988600	0.79866900
H	-0.78891600	1.30223300	0.57258500
C	-1.02535600	-0.00174900	2.25859800
H	-0.74085900	-1.03414900	2.51648900
H	-2.11689500	0.05667700	2.37807000
C	-0.36863800	0.97628600	3.22246200
H	0.72768400	0.95087400	3.13668700
H	-0.68913700	2.00845600	3.00840300
H	-0.63230400	0.74540600	4.26707000
C	0.15584100	4.61722600	0.34672700
C	1.66452600	4.97850500	0.56802500
B	1.43226400	2.70173900	0.35183200
O	0.15606200	3.18871900	0.50962200
O	2.32960900	3.74656000	0.24213300
H	3.92995200	2.80723800	1.72498800
C	4.02548700	1.80276700	2.17785800
O	2.83722300	1.07834900	2.02775800
H	4.85769200	1.29571800	1.64726900
C	4.38529600	1.92520400	3.64883900
H	4.56192000	0.91482400	4.05530200
H	5.34880100	2.45938800	3.72087300
C	3.32892400	2.62659500	4.48535500
H	2.35708200	2.12185000	4.38260200
H	3.59925100	2.63197100	5.55221200
H	3.19782200	3.67273500	4.16941800
C	-0.80918000	5.21006600	1.35780500
H	-0.56602600	4.89760100	2.38120300
H	-0.80202900	6.30979100	1.31220500
H	-1.82692300	4.86128300	1.12956400
C	-0.33510600	4.91017400	-1.06333400
H	0.32954200	4.47272400	-1.82144000
H	-1.32856300	4.45963800	-1.18535200
H	-0.41501800	5.99122000	-1.24813100
C	2.19910400	6.07323600	-0.33651400
H	1.66338700	7.01970900	-0.16799000

H	3.26431000	6.24060300	-0.12023800
H	2.10844800	5.80096600	-1.39564500
C	1.99103800	5.29345300	2.02197900
H	1.62134300	4.50260800	2.68918700
H	3.08270000	5.35231500	2.13962000
H	1.55710300	6.25314200	2.33782100

Cartesian coordinates of the optimized geometry of Int-**7a** at PBE0-D3BJ/def2-SVP level of theory:

Ga	-2.10047600	-0.64707100	-0.44692100
Cl	-1.50605400	-1.32809800	-2.49682900
Si	2.05399100	0.21252500	-0.03967400
O	0.53237200	-0.36655900	0.36087100
N	-2.94184500	-2.26526500	0.29201300
N	-3.80400900	0.27671700	-0.83008500
N	3.00407200	-0.65685300	-1.36367500
N	2.78745400	-1.68220300	0.52445300
C	-4.10507900	-2.66701400	-0.21779000
C	-4.95032800	-1.84118200	-0.97690500
H	-5.85278300	-2.31576800	-1.35921600
C	-4.85986100	-0.45420500	-1.18188500
C	-4.55631700	-4.08295000	0.00521000
H	-4.01559300	-4.74391000	-0.69042200
H	-5.63109000	-4.19092800	-0.18288700
H	-4.32342200	-4.43237300	1.01947800
C	-6.02897700	0.21209300	-1.85087100
H	-6.24086200	1.19859200	-1.41822600
H	-6.92718100	-0.41414400	-1.79422000
H	-5.78912600	0.37598600	-2.91320100
C	-2.22984100	-3.09792800	1.20945300
C	-1.35013600	-4.09340000	0.73872500
C	-0.67989700	-4.88229300	1.67960000
H	0.00385300	-5.66010900	1.33134600
C	-0.86083000	-4.69157400	3.04257000
H	-0.32170800	-5.31425100	3.76029000
C	-1.72106400	-3.69515700	3.49225600
H	-1.85021400	-3.54386800	4.56522000
C	-2.41865400	-2.88370800	2.59429300
C	-1.09380100	-4.31213000	-0.73872300
H	-1.78790600	-3.67195000	-1.30051800
C	-1.33194700	-5.75959800	-1.16622000
H	-0.60916300	-6.44711800	-0.69842800
H	-1.21849300	-5.85751800	-2.25701000
H	-2.33956100	-6.11120300	-0.89653400
C	0.31758500	-3.85997200	-1.11420800
H	0.53634600	-2.85079300	-0.73618400
H	0.43728400	-3.84717300	-2.20831500
H	1.06490800	-4.55223100	-0.69423400
C	-3.37822200	-1.81944900	3.09604600
H	-3.35951000	-1.00768100	2.35309000
C	-4.81863700	-2.33279900	3.16031500
H	-4.89178500	-3.21064000	3.82212900
H	-5.20226200	-2.61774700	2.17185800
H	-5.48614000	-1.55220500	3.55837200
C	-2.97426400	-1.22548100	4.44171000
H	-1.92412400	-0.90032100	4.44821100
H	-3.11436300	-1.94356800	5.26509100
H	-3.60183100	-0.35067000	4.67014800
C	-3.85604700	1.70229300	-0.90509000
C	-3.56326700	2.37328600	-2.11066000
C	-3.64657800	3.76934000	-2.12771100
H	-3.42726100	4.30169300	-3.05644500
C	-3.99109300	4.48884000	-0.99104000
H	-4.04957200	5.57940500	-1.02725300
C	-4.25372300	3.81520900	0.19721600
H	-4.51628000	4.38503200	1.09048700
C	-4.19300300	2.42116700	0.26447000

C	-3.14124200	1.64033600	-3.36802800
H	-3.22407200	0.56168000	-3.17464100
C	-4.02650900	1.97841000	-4.56705900
H	-3.92207700	3.03353800	-4.86647100
H	-5.09265100	1.80151100	-4.35870900
H	-3.74331300	1.36243400	-5.43452000
C	-1.67110500	1.91596500	-3.68794400
H	-1.53110800	2.95882400	-4.01640600
H	-1.32417300	1.25320700	-4.49483400
H	-1.02796600	1.74721500	-2.81327600
C	-4.52218400	1.69935800	1.55724200
H	-3.97604200	0.74395200	1.52992500
C	-6.00950600	1.35294500	1.65063300
H	-6.62737700	2.26402700	1.60349800
H	-6.22728800	0.84362200	2.60266900
H	-6.32755400	0.68437800	0.83919200
C	-4.07180000	2.46076800	2.80048000
H	-3.02259600	2.78258700	2.72533600
H	-4.16697700	1.82122700	3.69103000
H	-4.68872700	3.35534500	2.97949300
C	3.41200500	-1.71689100	-0.61134600
C	4.49949200	-2.64891100	-1.01268000
C	5.82171400	-2.19330700	-0.98574800
H	6.02860100	-1.16788200	-0.67113500
C	6.86405800	-3.04047600	-1.35645200
H	7.89413900	-2.67782200	-1.32710600
C	6.59185600	-4.34429400	-1.76716200
H	7.40819500	-5.00684100	-2.06414500
C	5.27391400	-4.80067700	-1.79839900
H	5.05510400	-5.82052500	-2.12312600
C	4.23222300	-3.95998400	-1.41815400
H	3.19985200	-4.31144500	-1.45053100
C	3.16415500	-0.40564900	-2.80702500
C	4.32961500	0.56206000	-3.03202000
H	4.16826200	1.49207200	-2.46943900
H	4.42942500	0.80978900	-4.10077400
H	5.27551100	0.10846200	-2.69950300
C	3.39652200	-1.68779500	-3.61090400
H	4.38801900	-2.12576200	-3.44419400
H	3.31640800	-1.44742500	-4.68148200
H	2.63122200	-2.44342100	-3.37813500
C	1.85755500	0.20335700	-3.32498700
H	1.62730500	1.15830900	-2.83604900
H	1.00792800	-0.47208100	-3.14853400
H	1.93663200	0.39992600	-4.40478500
C	3.01291800	-2.39960000	1.77914800
C	4.32482200	-1.95411500	2.43284100
H	4.29391800	-0.87862300	2.64665500
H	5.18697300	-2.16832900	1.78318500
H	4.47479400	-2.49283600	3.38131000
C	1.84285500	-2.03481800	2.69482600
H	1.94571100	-2.54758600	3.66314000
H	0.88858300	-2.32967800	2.23804000
H	1.82055600	-0.95094100	2.86695300
C	3.03273600	-3.92028000	1.60073900
H	3.02608300	-4.40044700	2.59105700
H	3.92776800	-4.26628600	1.06807600
H	2.13985500	-4.25588100	1.05814700
C	-0.68753100	0.33353700	0.53751400
H	-0.64266500	1.32785100	0.06126200
C	-0.95575200	0.51961400	2.02430300
H	-0.86453200	-0.46344600	2.51237500
H	-1.99882800	0.84055200	2.16555500
C	-0.03046000	1.52136300	2.70018800
H	1.02644500	1.24686100	2.56738200
H	-0.16405300	2.52798600	2.27287700
H	-0.23074500	1.58524800	3.78171200
C	0.87683100	4.21153400	-1.01179000
C	2.31876600	4.16978900	-1.61321300

B	1.74336200	2.09993100	-0.79515200
O	0.56597500	2.81131900	-0.88604000
O	2.81172900	2.91121500	-1.12318900
H	4.38547600	1.95063100	0.25311500
C	4.29519900	1.29930600	1.14127000
O	2.99253400	0.79885800	1.26392500
H	5.01389500	0.46559700	1.00971400
C	4.65768100	2.08741100	2.38619900
H	4.59588100	1.41926600	3.26368700
H	5.71169200	2.40804000	2.30886200
C	3.75416800	3.29103000	2.59663100
H	2.70882600	2.94123100	2.59399500
H	3.85085300	3.96349700	1.72582500
C	3.24086500	5.27661200	-1.13782600
H	2.85204300	6.26301000	-1.43303400
H	3.36366100	5.25959200	-0.04766400
H	4.23401900	5.15093100	-1.59314300
C	0.82612700	4.80001200	0.39359300
H	1.00081100	5.88560600	0.38842400
H	-0.17205800	4.61337500	0.81522900
H	1.56703600	4.32630500	1.05150300
C	-0.16184800	4.88215000	-1.88724900
H	-1.14719900	4.81070000	-1.40642500
H	0.08101500	5.94661100	-2.02748200
H	-0.23585400	4.40392900	-2.87043800
C	2.32804100	4.09847900	-3.13584300
H	2.03599500	5.05726300	-3.58785300
H	3.34404000	3.85051100	-3.47539400
H	1.64828800	3.31782200	-3.50560800
C	4.02298400	4.07226700	3.87179400
H	3.92772500	3.39684800	4.74180600
H	5.07132800	4.42424200	3.88149300
C	3.08931400	5.26136600	4.05424800
H	3.18204900	5.92994900	3.17928300
H	2.04497900	4.90149900	4.04119100
C	3.34485700	6.04891100	5.32891900
H	2.65336700	6.89946000	5.42974000
H	3.22257400	5.41356700	6.22104300
H	4.37113800	6.45002200	5.35180100

Cartesian coordinates of the optimized geometry of Int-**7b** at PBE0-D3BJ/def2-SVP level of theory:

Ga	2.53551000	-0.16770700	-0.33031700
Cl	2.44886600	0.84494600	-2.32733300
Si	-1.60239900	0.79479300	-0.21518700
O	-0.03887400	0.62181200	0.35936100
N	3.91701100	0.87840400	0.59661800
N	3.70623200	-1.70513900	-0.71837800
N	-1.96669900	2.07076900	-1.50394900
N	-1.53173000	2.74074300	0.49977900
C	5.17467600	0.77900100	0.16786700
C	5.64118300	-0.26836100	-0.64449700
H	6.68151000	-0.19351500	-0.95764700
C	4.98982500	-1.46713200	-0.98078700
C	6.16628500	1.84239800	0.54648600
H	6.01394100	2.72034800	-0.10091600
H	7.19596200	1.49054000	0.41201700
H	6.02612600	2.18301300	1.58064600
C	5.80251800	-2.51142700	-1.69223800
H	5.53873700	-3.52709300	-1.37009400
H	6.87691700	-2.34689600	-1.54776400
H	5.58717600	-2.45318200	-2.77120300
C	3.55299100	1.86790200	1.56165600
C	3.21391100	3.17403200	1.15469100
C	2.85841400	4.10306900	2.13835700
H	2.59506500	5.12039100	1.83892300
C	2.82800300	3.75662600	3.48201100

H	2.54153800	4.49593300	4.23357000
C	3.15199200	2.45997400	3.86761200
H	3.11622900	2.19183600	4.92468300
C	3.51813900	1.49571900	2.92548900
C	3.19466200	3.58894300	-0.30250000
H	3.59272000	2.75583000	-0.89804000
C	4.05791900	4.82055600	-0.57202800
H	3.66040900	5.71547300	-0.06708700
H	4.08491400	5.03766700	-1.65108700
H	5.09410300	4.68368700	-0.22703100
C	1.75929800	3.81788200	-0.77575200
H	1.10704900	2.97044000	-0.52021400
H	1.73215800	3.95213900	-1.86792700
H	1.34247000	4.72361700	-0.30644800
C	3.89454300	0.09134000	3.36185800
H	3.58446600	-0.58086400	2.54754500
C	5.40753600	-0.07224000	3.52389200
H	5.80148500	0.64268300	4.26383200
H	5.94516200	0.08278200	2.57924700
H	5.64739600	-1.08852700	3.87443200
C	3.18013700	-0.35943800	4.63185500
H	2.09402500	-0.19958800	4.57082600
H	3.55328300	0.17100700	5.52207600
H	3.35553300	-1.43232700	4.80182100
C	3.15555600	-3.01246400	-0.88896500
C	2.63529900	-3.42026000	-2.13448700
C	2.10459100	-4.70985700	-2.24099500
H	1.70054900	-5.03979400	-3.20121900
C	2.07619300	-5.57302800	-1.15403500
H	1.65709600	-6.57658500	-1.25960900
C	2.57759600	-5.15184300	0.07305100
H	2.54643600	-5.83153600	0.92654000
C	3.12148800	-3.87488700	0.23104300
C	2.61450300	-2.50804800	-3.34396200
H	3.14222600	-1.58142000	-3.07848300
C	3.32042800	-3.12579700	-4.55040200
H	2.79178000	-4.01937200	-4.91898500
H	4.35135400	-3.42953900	-4.31343000
H	3.36100500	-2.40288800	-5.37980200
C	1.18149800	-2.10974100	-3.69901300
H	0.61706000	-2.97449900	-4.08516900
H	1.17953800	-1.32787500	-4.47312400
H	0.64416900	-1.71751000	-2.82449000
C	3.69394600	-3.44469000	1.56855600
H	3.60781100	-2.34779700	1.60812400
C	5.18377200	-3.77505600	1.68114900
H	5.35329000	-4.85803100	1.56975900
H	5.57258000	-3.46989600	2.66539300
H	5.77957900	-3.25799000	0.91710800
C	2.93194200	-4.01840800	2.75968200
H	1.84781700	-3.85435800	2.67084100
H	3.27259000	-3.54212700	3.69139200
H	3.10167700	-5.10060200	2.87228200
C	-1.95920600	3.13866200	-0.66015700
C	-2.50286300	4.47820200	-1.00764400
C	-3.88979600	4.63347500	-1.09868400
H	-4.53894900	3.77466700	-0.91330800
C	-4.43795100	5.87368900	-1.41990000
H	-5.52255400	5.98752100	-1.48329100
C	-3.60380000	6.96345800	-1.66253900
H	-4.03260000	7.93461400	-1.92029600
C	-2.21972800	6.81081800	-1.57560500
H	-1.56187400	7.66134300	-1.76826000
C	-1.67033000	5.57600700	-1.24482800
H	-0.58828300	5.45166500	-1.18481300
C	-2.05887400	2.03822300	-2.97581600
C	-3.50259900	1.75083500	-3.39764100
H	-3.84020100	0.79662900	-2.96917100
H	-3.57942400	1.68633600	-4.49449900

H	-4.17314000	2.55498800	-3.05908400
C	-1.57983000	3.34085000	-3.62227500
H	-2.27388800	4.17613300	-3.46999100
H	-1.48817800	3.18058900	-4.70667700
H	-0.58845600	3.62758600	-3.24028900
C	-1.13363700	0.92783700	-3.48023200
H	-1.45586000	-0.05737800	-3.12391900
H	-0.09757800	1.09405200	-3.15138500
H	-1.15320200	0.89725300	-4.57995700
C	-1.57061600	3.37706100	1.81751100
C	-3.01173800	3.45176700	2.33282400
H	-3.43726400	2.44247200	2.40337800
H	-3.63967400	4.06417800	1.66785100
H	-3.03297900	3.91041800	3.33340900
C	-0.74271500	2.49111600	2.75020900
H	-0.72938700	2.92084700	3.76306000
H	0.29072200	2.40570600	2.38807300
H	-1.17119200	1.48185500	2.80140600
C	-0.95803100	4.78017400	1.81884300
H	-0.86054600	5.12936200	2.85787000
H	-1.57709900	5.50824300	1.27955600
H	0.04570700	4.76277000	1.37437300
C	0.76814500	-0.53801200	0.48023900
H	0.34854200	-1.37159500	-0.10797700
C	0.82419000	-0.96392100	1.93998200
H	1.13702000	-0.09450400	2.53944300
H	1.60941400	-1.72563200	2.06153100
C	-0.49167000	-1.51109000	2.47145600
H	-1.30228500	-0.78015100	2.33766700
H	-0.78421500	-2.42432200	1.93117600
H	-0.42412700	-1.75248700	3.54410900
C	-2.32272300	-3.26687100	-1.29957800
C	-3.34599600	-2.53995300	-2.23197500
B	-2.06613900	-0.99717800	-1.10637400
O	-1.41494800	-2.19955900	-0.96124900
O	-3.26763500	-1.18111200	-1.76052300
H	-4.34538600	0.47603300	-0.53737600
C	-4.21889300	0.57741700	0.55525400
O	-2.88154700	0.58350300	0.93134100
H	-4.69653500	1.53991400	0.83760400
C	-4.77942100	-3.01784500	-2.10149200
H	-4.86318300	-4.08188000	-2.36959200
H	-5.15875000	-2.87940200	-1.08139600
H	-5.42333600	-2.44388800	-2.78407900
C	-2.94408400	-3.74945500	0.00490400
H	-3.61822500	-4.60264900	-0.15662400
H	-2.14066600	-4.06962100	0.68352400
H	-3.50557200	-2.94641500	0.50115200
C	-1.54115200	-4.38981700	-1.95103100
H	-0.81804700	-4.79872400	-1.23135900
H	-2.21530400	-5.20091100	-2.26572000
H	-0.97531900	-4.03926000	-2.82172900
C	-2.92001900	-2.53408400	-3.69482200
H	-3.03205100	-3.52772800	-4.15199000
H	-3.55194600	-1.82454400	-4.24830600
H	-1.87417000	-2.21523300	-3.80679200
C	-4.98820300	-0.53428500	1.21521500
C	-4.45614900	-1.21414000	2.31474600
C	-6.24874600	-0.90358600	0.72778500
C	-5.16216600	-2.24843900	2.91941800
H	-3.46626700	-0.93130300	2.67362000
C	-6.96616200	-1.93542700	1.31761900
H	-6.66478200	-0.38486200	-0.14010300
C	-6.40772000	-2.59737800	2.40875100
H	-4.76498000	-2.79762200	3.77310000
H	-7.94289400	-2.24909500	0.94936100
N	-7.15056400	-3.69829900	3.02778500
O	-6.63931400	-4.25842700	3.97290300
O	-8.23032500	-3.98141700	2.55499300

Cartesian coordinates of the optimized geometry of Int-**8a** at PBE0-D3BJ/def2-SVP level of theory:

Ga	-1.86163600	-1.08850200	-0.54666500
Cl	-1.02189600	-2.12146700	-2.34345100
Si	2.14639200	0.27696400	-0.09460400
O	0.70150000	-0.48027300	0.24326900
N	-2.67920000	-2.55293900	0.46634300
N	-3.55720200	-0.38974300	-1.26985700
N	3.03995100	-0.80150900	-1.33577900
N	3.39429100	-0.79906000	0.78297500
C	-3.74082700	-3.16287500	-0.06320600
C	-4.56736400	-2.57034400	-1.02757300
H	-5.40304900	-3.17861400	-1.37183900
C	-4.55440200	-1.24016300	-1.49212200
C	-4.06701800	-4.56554900	0.36405600
H	-3.43536100	-5.25638200	-0.21855300
H	-5.11678800	-4.81467000	0.16733400
H	-3.83802700	-4.73853200	1.42327400
C	-5.74811400	-0.79867800	-2.29091900
H	-5.92981300	0.27936900	-2.19502600
H	-6.64564300	-1.35221900	-1.98861500
H	-5.56721400	-1.00598900	-3.35716700
C	-2.06796600	-3.03066300	1.66539800
C	-0.94261700	-3.87595500	1.61278000
C	-0.33502400	-4.24852400	2.81712900
H	0.54353700	-4.89816700	2.79044100
C	-0.82870700	-3.81378600	4.03948200
H	-0.34007200	-4.11599800	4.96878300
C	-1.94812400	-2.98803600	4.07688600
H	-2.32803200	-2.64547000	5.04114300
C	-2.58184900	-2.57543600	2.90319600
C	-0.38155100	-4.39731800	0.30582600
H	-1.01251200	-4.01385300	-0.50825700
C	-0.42061300	-5.92445800	0.24726100
H	0.24399700	-6.37684100	1.00085100
H	-0.08921900	-6.27802400	-0.74160400
H	-1.43404000	-6.31462800	0.42635900
C	1.03043800	-3.86935500	0.05494000
H	1.05960300	-2.77178800	0.10661200
H	1.37433600	-4.17203800	-0.94659700
H	1.74061400	-4.27206000	0.79647200
C	-3.79176800	-1.65968500	2.96664200
H	-3.81930200	-1.10865900	2.01364000
C	-5.10022100	-2.44724800	3.06638000
H	-5.10960200	-3.07397800	3.97261800
H	-5.25662700	-3.10355600	2.20070100
H	-5.95923900	-1.75999400	3.11991500
C	-3.71299300	-0.63765400	4.09803200
H	-2.75899200	-0.09116600	4.09338100
H	-3.82706700	-1.11073600	5.08584200
H	-4.52823000	0.09562800	4.00120200
C	-3.63043900	0.97519700	-1.68763400
C	-3.37571000	1.33784100	-3.02702300
C	-3.44579500	2.69306800	-3.36891200
H	-3.25116800	2.98881400	-4.40259900
C	-3.73772700	3.66642000	-2.42421700
H	-3.77671300	4.71922600	-2.71294800
C	-3.96484300	3.29664000	-1.10280300
H	-4.18302300	4.06571200	-0.36004200
C	-3.91822500	1.95684500	-0.71173000
C	-3.00249300	0.33240900	-4.09932300
H	-3.04463600	-0.67310000	-3.65580300
C	-3.95947900	0.37861700	-5.29127700
H	-3.87812500	1.33201100	-5.83695000
H	-5.01044400	0.26642300	-4.98621900
H	-3.72231900	-0.42697500	-6.00325800

C	-1.56150600	0.54780400	-4.56087600
H	-1.43920800	1.52879900	-5.04763800
H	-1.26721100	-0.23116000	-5.28070200
H	-0.86562600	0.49631500	-3.71499000
C	-4.21409200	1.56091400	0.72051100
H	-3.62969400	0.64994000	0.92003900
C	-5.68639400	1.18702900	0.90110200
H	-6.33823100	2.04131400	0.65847800
H	-5.88641900	0.89228100	1.94340900
H	-5.97699800	0.34547600	0.25673600
C	-3.78925100	2.61127700	1.74071500
H	-2.75177600	2.93785200	1.57959300
H	-3.85751400	2.20091200	2.75908900
H	-4.43606100	3.50195900	1.70760100
C	3.84836600	-1.25862800	-0.37909300
C	5.04458400	-2.11380800	-0.57818700
C	6.32070900	-1.54839400	-0.64925100
H	6.43831700	-0.46573600	-0.56989100
C	7.43413200	-2.36650500	-0.82900300
H	8.42929500	-1.92014300	-0.88751300
C	7.27853800	-3.74770900	-0.93360800
H	8.15337300	-4.38715600	-1.07207500
C	6.00527900	-4.31247300	-0.86282300
H	5.87924400	-5.39433300	-0.94456400
C	4.88883400	-3.49942800	-0.68858900
H	3.88910300	-3.93426700	-0.63021500
C	3.18885200	-0.79294300	-2.79472400
C	3.20805700	-2.21615500	-3.35217000
H	4.10795800	-2.76771600	-3.04835800
H	3.19292300	-2.18011200	-4.45180900
H	2.31659600	-2.76554400	-3.01452400
C	1.95657100	-0.06632000	-3.31915600
H	1.03881700	-0.58747600	-3.01160100
H	1.97675000	-0.01176400	-4.41712100
H	1.94902600	0.96048800	-2.92785200
C	4.44024200	-0.00947400	-3.20006200
H	5.35993800	-0.52179200	-2.88378200
H	4.41108900	0.99565100	-2.75083100
H	4.47823700	0.09898400	-4.29479600
C	3.88181700	-0.96721900	2.15507300
C	5.16912100	-0.16252500	2.35413900
H	5.00302000	0.87150700	2.01988400
H	6.00513700	-0.59686800	1.78795700
H	5.45219200	-0.15363000	3.41772800
C	2.79283100	-0.39923600	3.06096000
H	3.08874800	-0.49588000	4.11547300
H	1.84211400	-0.93140200	2.91192400
H	2.64892700	0.66644000	2.83444400
C	4.09704100	-2.44329800	2.49124000
H	4.34423100	-2.54046900	3.55894000
H	4.92101400	-2.88683800	1.91651200
H	3.17966000	-3.01902500	2.29742800
C	-0.58957600	0.10203900	0.40794100
H	-0.61488100	1.09281800	-0.07507900
C	-0.90304000	0.28096300	1.88844200
H	-0.62290200	-0.64851700	2.41035400
H	-1.99281200	0.37091700	2.01368300
C	-0.23938900	1.48676300	2.53335900
H	0.84757200	1.49146700	2.38247900
H	-0.60649300	2.42292600	2.09074200
H	-0.43096400	1.51469400	3.61794400
C	0.82362800	3.75479300	-1.16356400
C	2.24876100	3.95862200	-1.80372500
B	2.38625300	2.40172300	-0.04933500
O	1.11647500	3.09324100	0.04727200
O	3.00367500	2.88667800	-1.26471000
H	3.85176900	4.34194000	0.98924600
C	3.79667600	3.51656300	1.71905500
O	3.17358300	2.38860000	1.16516900

H	4.84305000	3.24590300	1.95747100
C	3.10871700	3.98284100	2.99514600
H	2.98154700	3.09865100	3.64562500
H	3.78588500	4.66669500	3.53864200
C	1.75897100	4.64677700	2.76313300
H	1.21139700	4.06906400	2.00301500
H	1.91797600	5.64708500	2.31818400
C	0.08110500	5.04725100	-0.84882000
H	0.63467900	5.66938700	-0.13475100
H	-0.10536900	5.63304500	-1.76243100
H	-0.89325800	4.80384700	-0.39848800
C	-0.09087300	2.87235400	-2.01083200
H	0.37131900	1.89980500	-2.22111900
H	-1.02843900	2.69195800	-1.46541900
H	-0.35615900	3.34048500	-2.96924600
C	2.27494400	3.87649300	-3.32365200
H	1.64714900	4.66045100	-3.77488500
H	3.30613300	4.01933200	-3.68054600
H	1.92701700	2.90184800	-3.68748500
C	2.89180700	5.27967400	-1.38095600
H	2.81329000	5.43601700	-0.29698000
H	3.95944700	5.24847000	-1.64395400
H	2.43286800	6.14305700	-1.88571100
C	0.90108800	4.77838900	4.01112900
H	1.42444100	5.37769200	4.77984200
H	0.76437700	3.77577900	4.45693300
C	-0.46729200	5.38723300	3.73581700
H	-0.33640000	6.40066200	3.31611600
H	-0.96250700	4.80075400	2.94155500
C	-1.36641700	5.44920500	4.95969400
H	-0.90970600	6.04987800	5.76317100
H	-2.34570100	5.89565500	4.72707700
H	-1.54913300	4.44199900	5.36880100

Cartesian coordinates of the optimized geometry of Int-**8b** at PBE0-D3BJ/def2-SVP level of theory:

Ga	-2.62443700	-0.32961400	0.24502500
Cl	-2.98433900	1.13410200	1.89614200
Si	1.29551300	1.35290400	0.27269200
O	-0.20168300	0.87334700	-0.26733300
N	-4.00937500	0.13177700	-1.05859800
N	-3.53348100	-1.93030200	0.93897400
N	1.04433800	2.83763400	1.37088800
N	1.62039100	2.90846000	-0.69629700
C	-5.27998900	-0.09458900	-0.72102400
C	-5.66329900	-1.01100500	0.26870200
H	-6.73503300	-1.09260700	0.44645800
C	-4.86292300	-1.94707200	0.95297600
C	-6.36777300	0.68397500	-1.40412400
H	-6.46282400	1.65812100	-0.89656800
H	-7.33441500	0.17001600	-1.33934700
H	-6.12920700	0.89199400	-2.45490100
C	-5.58618500	-2.99774300	1.74660700
H	-5.00397300	-3.92440500	1.82794000
H	-6.56699800	-3.21551500	1.30627800
H	-5.75086200	-2.62470500	2.76977400
C	-3.66530000	0.82011300	-2.26220100
C	-3.41766900	2.20670700	-2.25319000
C	-3.00333500	2.81521400	-3.44340300
H	-2.80210200	3.88953700	-3.44905700
C	-2.84904200	2.08349100	-4.61283600
H	-2.52423000	2.57720800	-5.53174800
C	-3.10926800	0.71655600	-4.61023000
H	-2.98343500	0.14644900	-5.53242800
C	-3.51273900	0.05945000	-3.44607900
C	-3.59307200	3.05377600	-1.00959500
H	-3.95204500	2.39889800	-0.20339000

C	-4.63514600	4.15243100	-1.21917600
H	-4.30643100	4.88619200	-1.97263700
H	-4.80935900	4.69881300	-0.27910700
H	-5.59854300	3.74254000	-1.55841200
C	-2.26046300	3.63753000	-0.54210300
H	-1.50891600	2.84840600	-0.39902200
H	-2.39193800	4.16077400	0.41788400
H	-1.86858800	4.36153000	-1.27580700
C	-3.78400300	-1.43436500	-3.46275400
H	-3.60196100	-1.79799900	-2.43958600
C	-5.24561500	-1.74434200	-3.79455900
H	-5.51285500	-1.34593800	-4.78647700
H	-5.93851700	-1.31392600	-3.06026300
H	-5.41367800	-2.83271000	-3.80933900
C	-2.86289000	-2.20420400	-4.40524800
H	-1.80356700	-1.96787900	-4.23025200
H	-3.08753400	-1.99133500	-5.46202400
H	-3.00019400	-3.28709900	-4.26403200
C	-2.75989000	-2.96215500	1.55655100
C	-2.50813400	-2.94622700	2.94429500
C	-1.71768100	-3.96694600	3.48443100
H	-1.51420500	-3.96697900	4.55797700
C	-1.17804500	-4.96629100	2.68741700
H	-0.55716100	-5.74831700	3.13039800
C	-1.42176300	-4.96095000	1.31816200
H	-0.98957800	-5.74418800	0.69325300
C	-2.21051600	-3.96961100	0.73045200
C	-3.03499600	-1.86433000	3.86663400
H	-3.65799900	-1.18087200	3.27155700
C	-3.89030200	-2.43893000	4.99655400
H	-3.29014900	-3.05704300	5.68289900
H	-4.70727800	-3.07102100	4.61811500
H	-4.33462400	-1.62584500	5.59121700
C	-1.88535300	-1.03132100	4.43209900
H	-1.22801100	-1.63861800	5.07504300
H	-2.27381300	-0.19445300	5.03219400
H	-1.27546300	-0.60486100	3.62639800
C	-2.50396600	-3.99987400	-0.75582500
H	-2.65261800	-2.95429800	-1.06575200
C	-3.81247900	-4.73416700	-1.05310400
H	-3.75823700	-5.78125500	-0.71520300
H	-4.01763900	-4.73637000	-2.13544000
H	-4.66974200	-4.26152100	-0.55384800
C	-1.35946500	-4.57050200	-1.58614700
H	-0.39987800	-4.09398300	-1.33837700
H	-1.54976900	-4.40809100	-2.65725900
H	-1.24761100	-5.65612800	-1.43992800
C	1.47460500	3.64591000	0.40192300
C	1.75746500	5.09631900	0.53410400
C	3.05297800	5.54440900	0.80734300
H	3.86223300	4.82083200	0.92510900
C	3.30405200	6.90893700	0.93259500
H	4.31716400	7.25482100	1.14922000
C	2.26706800	7.82828800	0.78192200
H	2.46692600	8.89785500	0.87894700
C	0.97450600	7.38182200	0.50898900
H	0.15920100	8.09905800	0.39168100
C	0.71668500	6.01893900	0.38766300
H	-0.29356300	5.66180400	0.17845500
C	0.92725900	3.03657400	2.81961400
C	-0.06697800	4.15227000	3.13963000
H	0.29766200	5.13693700	2.81707200
H	-0.23101600	4.19573800	4.22668300
H	-1.03349500	3.94894000	2.65490200
C	0.39158200	1.71523100	3.35887800
H	-0.58694300	1.48334500	2.91524500
H	0.27843800	1.76016600	4.45158300
H	1.10566000	0.91262800	3.12499600
C	2.30222500	3.31151000	3.43365000

H	2.70556700	4.27952200	3.10364000
H	3.00095000	2.50794600	3.15318700
H	2.22403500	3.33976800	4.53125200
C	2.05246800	3.25271800	-2.05370100
C	3.52249700	3.67913200	-2.05585900
H	4.12349200	2.91181500	-1.54714500
H	3.66523000	4.64436700	-1.55069400
H	3.88423100	3.78440200	-3.08981300
C	1.90335000	1.97649900	-2.87722500
H	2.20203600	2.16016300	-3.91917400
H	0.86018000	1.62845900	-2.86897900
H	2.55173800	1.19136100	-2.46129200
C	1.16074200	4.34431100	-2.64683100
H	1.44405400	4.52263800	-3.69497100
H	1.26001700	5.29593700	-2.10683900
H	0.10663100	4.03057900	-2.62335100
C	-0.74162500	-0.44219500	-0.37596400
H	-0.20413500	-1.12295300	0.30441600
C	-0.58939300	-0.96349900	-1.79836400
H	-0.93689200	-0.17867400	-2.49037800
H	-1.27973200	-1.80870600	-1.93876700
C	0.81576700	-1.41767800	-2.15977800
H	1.54947100	-0.61249800	-2.02384200
H	1.14613500	-2.24350800	-1.51338200
H	0.86989800	-1.74550500	-3.21004100
C	2.61842100	-1.96390700	1.81546100
C	3.57091800	-1.02947700	2.65211900
B	2.93583100	0.05356400	0.66866100
O	2.60453700	-1.34573900	0.54611300
O	3.41617800	0.23450900	2.02050900
H	5.54827200	0.46865900	0.66505800
C	5.16071600	0.37517300	-0.36649500
O	3.78901300	0.56995400	-0.40112500
H	5.64151100	1.18573700	-0.94947500
C	3.12148600	-3.39171300	1.65338800
H	4.07992800	-3.42071000	1.11985600
H	3.23905100	-3.88896400	2.62868400
H	2.39230500	-3.96878900	1.06496000
C	1.19674200	-2.00815900	2.37342700
H	0.77448200	-1.00019400	2.48185200
H	0.55277900	-2.57584100	1.68597700
H	1.14643000	-2.50505500	3.35260100
C	3.19916300	-0.90178400	4.12247600
H	3.25089300	-1.87786400	4.62894200
H	3.90626600	-0.22252000	4.62216900
H	2.19002700	-0.49411100	4.25853600
C	5.03449800	-1.45997000	2.55949600
H	5.33846000	-1.65421600	1.52298100
H	5.66559600	-0.65208900	2.95970600
H	5.23021900	-2.36985400	3.14596900
C	5.63403200	-0.93615400	-0.94791500
C	6.98322600	-1.29972600	-0.83306600
C	4.75292100	-1.79359800	-1.61299800
C	7.45075800	-2.49306400	-1.36608600
H	7.67754600	-0.63771400	-0.30750200
C	5.20406200	-2.99310300	-2.15169500
H	3.70021100	-1.52077600	-1.66835700
C	6.54809600	-3.32757400	-2.02126300
H	8.49380900	-2.79822600	-1.28369500
H	4.53333100	-3.68068900	-2.66692000
N	7.02724800	-4.59252100	-2.58075300
O	6.21981800	-5.30065500	-3.14349300
O	8.20362100	-4.85621900	-2.44832200

Cartesian coordinates of the optimized geometry of TS-**1a** at PBE0-D3BJ/def2-SVP level of theory:

Ga	-1.74067600	-0.50210300	-0.67655700

Cl	-1.04341000	-1.38083600	-2.61909700
Si	2.29071700	0.70574400	0.23999300
O	0.74580600	0.08146900	0.35640300
N	-2.42117500	-2.10657600	0.22765300
N	-3.51673000	0.21563000	-1.11965400
N	3.09716400	-0.19062300	-1.20681400
N	3.31475800	-0.73448700	0.85549900
C	-3.47869400	-2.69605100	-0.33257500
C	-4.33912100	-2.04688300	-1.23295900
H	-5.12268300	-2.66639600	-1.66915700
C	-4.43512900	-0.66810000	-1.50157200
C	-3.76778900	-4.13669400	-0.01951300
H	-3.11807000	-4.77246600	-0.64219900
H	-4.81104700	-4.39177100	-0.24093500
H	-3.54378500	-4.38003200	1.02733000
C	-5.64649300	-0.22244000	-2.27243900
H	-5.61880900	-0.70385500	-3.26248800
H	-5.67566800	0.86431700	-2.41190500
H	-6.57209900	-0.54969200	-1.77903600
C	-1.68923900	-2.72977700	1.28401200
C	-0.63643500	-3.62194300	0.99657600
C	0.08085100	-4.17392900	2.06329900
H	0.89863800	-4.86732900	1.85145800
C	-0.22424100	-3.85735700	3.37920100
H	0.35009400	-4.29549200	4.19890300
C	-1.26412300	-2.97434100	3.64946100
H	-1.49685000	-2.72737400	4.68614400
C	-2.01213700	-2.39622200	2.62042400
C	-0.23865900	-3.98218900	-0.41933300
H	-0.96277400	-3.52322600	-1.10658200
C	-0.24826200	-5.49139000	-0.65896200
H	0.52487600	-6.00480300	-0.06506000
H	-0.04701300	-5.70993200	-1.71918800
H	-1.21680400	-5.94343300	-0.39630800
C	1.12435700	-3.38182900	-0.75740500
H	1.13662600	-2.29542200	-0.59182100
H	1.37365900	-3.56953300	-1.81302000
H	1.91157400	-3.83248700	-0.13090100
C	-3.16074000	-1.45696200	2.94321900
H	-3.20594000	-0.71843500	2.12702300
C	-4.50301500	-2.19271900	2.95704000
H	-4.49365100	-3.01000400	3.69585400
H	-4.74868900	-2.62249200	1.97696500
H	-5.31749800	-1.50236600	3.22680100
C	-2.96194100	-0.70113600	4.25346800
H	-1.98177200	-0.20319200	4.29360900
H	-3.03986400	-1.36839600	5.12609100
H	-3.73774700	0.06835300	4.36973400
C	-3.78183500	1.61527800	-1.05745200
C	-3.06497900	2.50406900	-1.89464600
C	-3.30303000	3.87393800	-1.76784600
H	-2.75059800	4.56879300	-2.40096100
C	-4.22644800	4.36928200	-0.85437900
H	-4.39964000	5.44514800	-0.77721200
C	-4.91460200	3.48922100	-0.03253700
H	-5.62734300	3.88077700	0.69757700
C	-4.69918200	2.10875100	-0.10148200
C	-2.10488300	1.99447800	-2.95030300
H	-1.58787200	1.11324700	-2.54454900
C	-2.86722700	1.50223200	-4.18116200
H	-3.41308500	2.33109900	-4.66037200
H	-3.59171500	0.71825200	-3.92149500
H	-2.16886300	1.07335500	-4.91554800
C	-1.01542800	2.98815800	-3.33513500
H	-1.42377600	3.86755700	-3.85896500
H	-0.31375100	2.50293300	-4.02972100
H	-0.43550500	3.32628000	-2.46250800
C	-5.43559600	1.21909600	0.88500700
H	-5.10687900	0.18104900	0.72633500

C	-6.95149200	1.26993000	0.68612000
H	-7.34645000	2.27281700	0.91338000
H	-7.45458700	0.55628700	1.35745500
H	-7.24088400	1.03013100	-0.34598700
C	-5.08252200	1.59501200	2.32429100
H	-3.99771000	1.56006300	2.49574800
H	-5.56635800	0.90631600	3.03407500
H	-5.42443100	2.61283300	2.56750200
C	3.81155700	-0.93785500	-0.36678400
C	5.01360800	-1.74700500	-0.69500100
C	6.28302400	-1.17344800	-0.56948100
H	6.37621100	-0.13282800	-0.25055600
C	7.42089600	-1.92402100	-0.85753000
H	8.40919500	-1.46902900	-0.76014200
C	7.29735700	-3.24987200	-1.26964200
H	8.19002500	-3.83822800	-1.49404400
C	6.03249400	-3.82314500	-1.39716300
H	5.93110700	-4.86125700	-1.72138800
C	4.89258200	-3.07584400	-1.11296800
H	3.90094800	-3.52044400	-1.21007300
C	3.21885700	0.06494900	-2.64498500
C	4.49771900	0.85092800	-2.94698300
H	4.51899900	1.79092100	-2.37452100
H	4.54466300	1.10365600	-4.01699100
H	5.39580100	0.26683000	-2.69799900
C	3.19909800	-1.24499100	-3.43478900
H	4.09224000	-1.85661100	-3.24868500
H	3.16547300	-1.02070900	-4.51136600
H	2.30073400	-1.82551800	-3.18045800
C	1.98737300	0.88303000	-3.03725000
H	1.89012700	1.81089500	-2.45305300
H	1.07394800	0.28920800	-2.88729800
H	2.04593100	1.15645800	-4.10118600
C	3.68507100	-1.21550200	2.18335200
C	4.91546600	-0.46204500	2.69770700
H	4.73861200	0.62400600	2.66456400
H	5.80242900	-0.69070200	2.08898000
H	5.13622700	-0.74572900	3.73820000
C	2.48241100	-0.91798300	3.08166400
H	2.68031100	-1.25465200	4.10972300
H	1.58079400	-1.42379200	2.70733300
H	2.27879300	0.16423100	3.11312600
C	3.94377400	-2.72218400	2.18318100
H	4.09820100	-3.06653200	3.21665100
H	4.83698700	-2.98945000	1.60297600
H	3.07742800	-3.25833000	1.76991600
C	-0.51046300	0.74497900	0.23618900
H	-0.39461400	1.64054100	-0.39708200
C	-1.04113200	1.18754300	1.59065800
H	-1.11739100	0.30861900	2.25149900
H	-2.06904500	1.55986600	1.43589700
C	-0.21285100	2.27130300	2.26019100
H	0.79602600	1.90116500	2.50442100
H	-0.09140700	3.13332000	1.58735600
H	-0.67436900	2.60920600	3.20127600
C	2.37452500	2.99614600	-0.22577800
O	1.22379900	3.24124700	-0.65423900
H	3.19806000	2.80756400	-0.96809700
C	2.91552800	3.70472100	1.00641000
H	2.23028100	3.56014500	1.85479100
H	3.88810800	3.26237400	1.28541300
C	3.06120400	5.19737200	0.72625400
H	3.73551700	5.34602000	-0.13752700
H	2.07826600	5.58616100	0.41159700
C	3.57909700	5.99088700	1.91599700
H	4.55919500	5.58715800	2.23167500
H	2.90377900	5.83639600	2.77764900
C	3.71395300	7.48294700	1.64440900
H	2.73411600	7.87967500	1.32488000

H	4.38955400	7.63386400	0.78362600
C	4.22270200	8.27382100	2.83891700
H	5.21672800	7.92043400	3.15818000
H	4.30929100	9.34741000	2.61154200
H	3.54648600	8.17058300	3.70307100

Cartesian coordinates of the optimized geometry of TS-**1b** at PBE0-D3BJ/def2-SVP level of theory:

Ga	2.18200400	0.38045800	-0.53459300
Cl	1.66145600	1.48551100	-2.41794100
Si	-1.88442400	-0.61537900	-0.45150600
O	-0.44799500	-0.10337100	0.20964200
N	2.92791900	1.83191700	0.54549900
N	3.90418600	-0.45368100	-0.95435800
N	-2.50750300	0.62812500	-1.71563800
N	-3.12825600	0.50151100	0.34071600
C	4.06303600	2.37112900	0.09778700
C	4.91361800	1.73141500	-0.81966500
H	5.76825000	2.31686100	-1.15869800
C	4.91055800	0.37976400	-1.21251000
C	4.45780100	3.74410900	0.56143000
H	3.93464500	4.49004900	-0.05842900
H	5.53684100	3.90717600	0.45334300
H	4.15993200	3.92572900	1.60220000
C	6.11497500	-0.09701600	-1.97484500
H	6.15471300	0.45374100	-2.92785000
H	6.07289900	-1.17023800	-2.19293100
H	7.04336200	0.12741200	-1.43205600
C	2.18991500	2.41254800	1.62296200
C	1.23010000	3.41522300	1.37725400
C	0.49847200	3.91766100	2.45883200
H	-0.24923400	4.69420400	2.27942400
C	0.70096000	3.44793000	3.74821100
H	0.11751800	3.85054400	4.57954800
C	1.65015400	2.45768700	3.97692000
H	1.80234600	2.09091200	4.99280900
C	2.40718700	1.92181000	2.93206100
C	0.94654800	3.95536400	-0.00913300
H	1.65447800	3.49143900	-0.70996900
C	1.12771800	5.47122600	-0.08487000
H	0.38202500	6.00239700	0.52799400
H	1.00847200	5.81866900	-1.12288300
H	2.12251200	5.78391300	0.26658400
C	-0.45324300	3.54927500	-0.46701500
H	-0.57912300	2.45756600	-0.44425000
H	-0.63222100	3.89069400	-1.49773400
H	-1.22288600	3.99807200	0.18223800
C	3.45514400	0.85982900	3.21366700
H	3.48795800	0.20212200	2.33034200
C	4.84836200	1.47498700	3.36857000
H	4.86056300	2.21286200	4.18655300
H	5.18254200	1.97852700	2.45181400
H	5.58974800	0.69595500	3.60503800
C	3.12119600	0.00054700	4.42924000
H	2.10539100	-0.41727000	4.36860800
H	3.19797800	0.57220700	5.36724700
H	3.82764600	-0.83716600	4.50820400
C	4.05550500	-1.87152500	-1.00063600
C	3.31151400	-2.62964500	-1.93639300
C	3.44382400	-4.01945900	-1.92211500
H	2.86907800	-4.61501700	-2.63174000
C	4.29064700	-4.65907600	-1.02465900
H	4.38251500	-5.74746800	-1.03682500
C	5.00346500	-3.90665200	-0.10335900
H	5.65308900	-4.41183700	0.61564300
C	4.89012600	-2.51328000	-0.05689500
C	2.43538400	-1.96459300	-2.97858000

H	1.98408600	-1.07045200	-2.52487500
C	3.27904800	-1.45650900	-4.14834200
H	3.76540100	-2.29509400	-4.67226700
H	4.06164700	-0.76354400	-3.80958600
H	2.64805000	-0.91411900	-4.86871300
C	1.27994400	-2.83400100	-3.46246900
H	1.63263500	-3.70378700	-4.03941300
H	0.64307100	-2.24635300	-4.14022200
H	0.65237500	-3.19573400	-2.63077200
C	5.63698900	-1.77206300	1.03818000
H	5.39005200	-0.70237800	0.96321000
C	7.15387400	-1.91634800	0.90452600
H	7.46601800	-2.96247100	1.05106700
H	7.66911400	-1.30799600	1.66437500
H	7.51331400	-1.60268300	-0.08484100
C	5.18301700	-2.25071400	2.41740200
H	4.09473700	-2.15684400	2.53844100
H	5.67085800	-1.66462100	3.21137000
H	5.44416700	-3.30759400	2.58037400
C	-3.39412300	1.08803800	-0.82802200
C	-4.51940200	2.01491300	-1.09625800
C	-5.81932800	1.51289600	-1.22557600
H	-5.99560000	0.43989100	-1.12593500
C	-6.87814500	2.38019500	-1.47611100
H	-7.89022400	1.98321300	-1.58061900
C	-6.64753100	3.75176500	-1.58569300
H	-7.48077900	4.43161600	-1.77740900
C	-5.35519600	4.25446100	-1.44707900
H	-5.17236000	5.32823700	-1.52718600
C	-4.28946100	3.38913900	-1.20712800
H	-3.27493100	3.77715100	-1.09750300
C	-2.48205700	0.70220600	-3.18347800
C	-3.80959800	0.24608900	-3.79260200
H	-4.07109800	-0.76421800	-3.44116000
H	-3.72287500	0.20775100	-4.88862600
H	-4.63373800	0.92923500	-3.54536800
C	-2.13955100	2.12293700	-3.63419200
H	-2.91802000	2.84097100	-3.34209800
H	-2.05061500	2.15134700	-4.73042200
H	-1.17729200	2.43240100	-3.20326900
C	-1.36532400	-0.23714000	-3.63553900
H	-1.58527600	-1.28007000	-3.36491800
H	-0.40629000	0.05827700	-3.18557600
H	-1.25372900	-0.19023500	-4.72836800
C	-3.54373800	0.81126300	1.71345700
C	-5.06055400	0.73934000	1.89401500
H	-5.45013000	-0.23231200	1.55757600
H	-5.58099000	1.53818300	1.34986100
H	-5.30496000	0.84980000	2.96088600
C	-2.88179500	-0.24177100	2.59928900
H	-3.15620600	-0.07954800	3.65110600
H	-1.78765100	-0.18180500	2.51297000
H	-3.20686600	-1.25371800	2.31239300
C	-3.01862300	2.19730400	2.09500600
H	-3.25980200	2.41742900	3.14590300
H	-3.47728100	2.98037700	1.47280400
H	-1.92671200	2.24218000	1.97292800
C	0.78810000	-0.83243500	0.16505100
H	0.69016600	-1.68102600	-0.53495600
C	1.13707500	-1.39230600	1.53406500
H	1.16419200	-0.56167900	2.25941300
H	2.16655100	-1.78723300	1.47105500
C	0.21105500	-2.49573300	2.02116300
H	-0.80908800	-2.11920600	2.18517000
H	0.14040800	-3.29946100	1.27356600
H	0.56197700	-2.91521000	2.97703100
C	-2.07337100	-2.87825400	-1.00960600
O	-1.00843500	-3.49320500	-0.80411400
H	-2.38901600	-2.62209700	-2.05612500

C	-3.28809000	-3.06767500	-0.13841200
C	-3.17890400	-3.78401200	1.05985200
C	-4.54107500	-2.55907700	-0.52175400
C	-4.27637100	-3.91936300	1.90190000
H	-2.20815700	-4.21545900	1.31030300
C	-5.64223500	-2.68634600	0.30470300
H	-4.63371800	-2.03881100	-1.48080400
C	-5.49073800	-3.34961200	1.52627900
H	-4.21640300	-4.45296600	2.85093800
H	-6.62208500	-2.28912900	0.03861800
N	-6.63452700	-3.44245000	2.42400000
O	-6.50563000	-4.08948600	3.44205200
O	-7.64878900	-2.85411900	2.10355400

Cartesian coordinates of the optimized geometry of TS-**2a** at PBE0-D3BJ/def2-SVP level of theory:

Ga	-2.25964300	-0.34000100	-0.61184300
Cl	-1.79939500	-0.95334800	-2.71763100
Si	1.93031100	-0.46556900	0.24701100
O	0.30317700	-0.58180500	0.31013600
N	-3.06759600	-2.00416300	0.07992500
N	-3.96447100	0.60009500	-0.82648000
N	2.67479100	-1.12596100	-1.29405300
N	2.68481400	-2.07712900	0.63790400
C	-4.26135600	-2.35853500	-0.39993200
C	-5.09840900	-1.50147400	-1.13380800
H	-6.00009700	-1.95626900	-1.54046400
C	-5.00782900	-0.10733700	-1.25947100
C	-4.77036600	-3.75130600	-0.15356900
H	-5.83148900	-3.83143400	-0.41596400
H	-4.63193800	-4.04875400	0.89499300
H	-4.20453200	-4.47303500	-0.76092000
C	-6.14649800	0.60629000	-1.92857400
H	-7.03002700	-0.03700800	-2.01383400
H	-5.82467900	0.89636500	-2.94216600
H	-6.41459700	1.53418200	-1.40764700
C	-2.35390300	-2.92508700	0.90550000
C	-1.63079600	-3.98879300	0.32598600
C	-1.02652000	-4.91922900	1.17705000
H	-0.48455400	-5.76294000	0.74300200
C	-1.10268900	-4.79414600	2.55834000
H	-0.62474700	-5.53417300	3.20470700
C	-1.77654400	-3.71280500	3.11414900
H	-1.81756800	-3.60682400	4.19952900
C	-2.41037000	-2.76398300	2.30677800
C	-1.45304000	-4.12734500	-1.17311600
H	-2.13426900	-3.42071800	-1.66822000
C	-1.76138500	-5.53148900	-1.68931200
H	-1.03416200	-6.27367800	-1.32307100
H	-1.71815400	-5.54832400	-2.78903700
H	-2.76164700	-5.87650400	-1.38594600
C	-0.03711800	-3.70680500	-1.56566300
H	0.18246000	-2.68714700	-1.22269900
H	0.08199800	-3.72842100	-2.65953100
H	0.70241400	-4.39190200	-1.11921100
C	-3.17812100	-1.61531900	2.93096100
H	-3.13733900	-0.78094200	2.21325200
C	-4.65448900	-1.96772400	3.12365800
H	-4.76343300	-2.86005500	3.76059100
H	-5.15655500	-2.16734900	2.16677200
H	-5.19039100	-1.13791900	3.60965700
C	-2.56084000	-1.12709500	4.23773000
H	-1.48220600	-0.93971800	4.12951100
H	-2.70143800	-1.85125600	5.05554400
H	-3.03664800	-0.18709100	4.55254700
C	-4.08563400	2.01327800	-0.65434200
C	-3.63213200	2.89956500	-1.64993600

C	-3.73350000	4.27321700	-1.40605100
H	-3.36912400	4.97454200	-2.16038700
C	-4.29070800	4.75910700	-0.23063200
H	-4.36293400	5.83603700	-0.06238200
C	-4.74819100	3.86998200	0.73610400
H	-5.17749600	4.25822900	1.66236300
C	-4.64171700	2.48954100	0.55485000
C	-3.05462900	2.41286500	-2.96304800
H	-3.13426400	1.31648600	-2.98067400
C	-3.84360700	2.94956500	-4.15784800
H	-3.75285700	4.04383600	-4.24672600
H	-4.91610700	2.71243600	-4.08094000
H	-3.46353300	2.51030700	-5.09313500
C	-1.57133000	2.75690300	-3.08493100
H	-1.40938300	3.84604200	-3.12316800
H	-1.15007400	2.31686600	-4.00173000
H	-0.98392900	2.37887600	-2.23677700
C	-5.11088300	1.54277300	1.64575800
H	-4.68082000	0.55501400	1.42192900
C	-6.63270800	1.38728200	1.64643700
H	-7.12598300	2.35536900	1.83020700
H	-6.95084900	0.69030200	2.43784300
H	-7.00681200	0.99715800	0.68973500
C	-4.61427600	1.95860100	3.02878700
H	-3.52131300	2.07004000	3.04750200
H	-4.89874600	1.20445800	3.77847400
H	-5.05497600	2.91409700	3.35258500
C	3.18722600	-2.14363900	-0.60519800
C	4.19653600	-3.11670600	-1.08452000
C	5.55230400	-2.81041500	-0.93371300
H	5.84782500	-1.86639500	-0.47074300
C	6.52005200	-3.70815800	-1.37912400
H	7.57813900	-3.46435900	-1.26223000
C	6.13805300	-4.90914300	-1.97388200
H	6.89813200	-5.61111300	-2.32408200
C	4.78522200	-5.21285100	-2.12541300
H	4.48268000	-6.15088400	-2.59573800
C	3.81336400	-4.32030700	-1.68325700
H	2.75410200	-4.54820600	-1.81033400
C	2.89087000	-0.65465200	-2.67824000
C	4.22110100	0.09376800	-2.76313000
H	4.19443700	0.97890300	-2.10969600
H	4.37636100	0.44771300	-3.79351000
H	5.06527700	-0.56270900	-2.50235900
C	2.85661900	-1.82395200	-3.66141000
H	3.73859200	-2.47273500	-3.57940800
H	2.83679100	-1.41887900	-4.68323600
H	1.94834700	-2.42830500	-3.52093100
C	1.73171400	0.29033300	-2.98072900
H	1.72356200	1.15185000	-2.29593600
H	0.76601100	-0.23335400	-2.92846100
H	1.84694900	0.69881800	-3.99507800
C	2.86145900	-2.92035100	1.82731700
C	4.26970700	-2.75986300	2.40338800
H	4.48623100	-1.70571400	2.62835300
H	5.03104000	-3.13235000	1.70319500
H	4.36143400	-3.33375400	3.33753400
C	1.81488400	-2.43588400	2.83104200
H	1.86564700	-3.03641400	3.75017300
H	0.80199000	-2.52651100	2.41314000
H	1.98875800	-1.38517800	3.10723500
C	2.59489700	-4.38841500	1.49704700
H	2.59333600	-4.97658200	2.42651800
H	3.36563900	-4.80750100	0.83694400
H	1.61065600	-4.50006700	1.02346000
C	-0.68033600	0.48550900	0.27612100
H	-0.30068000	1.26871300	-0.40057300
C	-0.85270900	1.12146700	1.65023700
H	0.11806300	1.04525600	2.16440900

H	-1.57695700	0.56891300	2.26979400
C	-1.21641500	2.59469200	1.55849700
H	-0.39864000	3.15233400	1.08114300
H	-2.13292800	2.76337700	0.97596200
H	-1.37898400	3.02284700	2.55940100
C	2.75186500	1.09952100	0.92160100
O	1.83680100	2.09549300	0.65211000
H	3.69176200	1.23836400	0.34888700
C	3.08037700	1.06650900	2.41377000
H	2.15688700	0.86640000	2.98659900
H	3.77539800	0.23684100	2.63096100
C	3.66954100	2.38630000	2.88779300
H	4.66450100	2.52275500	2.42923400
H	3.03680800	3.19909200	2.49435900
H	0.93096400	2.74096400	-1.24269200
C	2.80275000	5.05016100	-1.03900600
C	3.89689900	3.97677700	-1.39102600
O	3.17621600	2.76550200	-1.30593900
O	2.01005100	4.37190400	-0.10226200
C	4.49136500	4.11254300	-2.78633000
H	4.95960100	5.09943000	-2.92953200
H	5.26704700	3.34593100	-2.94148900
H	3.72194100	3.97185000	-3.55686500
C	5.02333400	3.95541700	-0.35779700
H	4.60655500	3.92985800	0.65762100
H	5.62672900	3.04609900	-0.50761400
H	5.69156500	4.82623300	-0.44391400
C	1.95035600	5.41299300	-2.26114300
H	1.07081700	5.97347500	-1.91066000
H	2.49578700	6.03425700	-2.98811800
H	1.58903600	4.50602500	-2.76644500
C	3.34685900	6.32272900	-0.40761900
H	4.06666800	6.82843900	-1.07080100
H	2.51622700	7.01724500	-0.21053600
H	3.83563900	6.10845000	0.55199900
B	1.93761900	2.98815700	-0.53806700
C	3.78573200	2.49261800	4.39975700
H	4.39633800	1.65673600	4.79125400
H	2.78428900	2.36647400	4.85149500
C	4.38230400	3.81281400	4.86770400
H	5.38357500	3.93573500	4.41796700
H	3.77380000	4.64125600	4.46507600
C	4.48130700	3.93329300	6.37972400
H	5.11177900	3.13512900	6.80482400
H	4.91604300	4.89744500	6.68481200
H	3.48883000	3.85248200	6.85223100

Cartesian coordinates of the optimized geometry of TS-**2b** at PBE0-D3BJ/def2-SVP level of theory:

Ga	2.15629600	-0.79688500	0.63422500
Cl	1.19167900	-1.60007200	2.48940800
Si	-1.84774600	0.28020000	0.03492300
O	-0.43355200	-0.34216900	-0.49599800
N	3.28045500	-2.33601600	0.13309700
N	3.62052300	0.25345000	1.42125900
N	-2.68902900	-0.87036000	1.22991400
N	-3.09588500	-0.71345400	-0.88502900
C	4.20558900	-2.69455000	1.02688500
C	4.68455900	-1.83597300	2.02690400
H	5.39185900	-2.27522000	2.72857200
C	4.51524700	-0.44310000	2.12739800
C	4.75901400	-4.09013400	0.98628100
H	3.97756700	-4.79816800	1.30442000
H	5.62109900	-4.19539400	1.65499800
H	5.04932900	-4.38248900	-0.03182900
C	5.44342700	0.27432800	3.06685900
H	6.33001300	0.63962200	2.52796900

H	5.78735800	-0.41246400	3.85068500
H	4.96199000	1.14798100	3.52441600
C	3.01800300	-3.18498800	-0.98635200
C	2.03898800	-4.19476000	-0.90426000
C	1.81683600	-5.00273000	-2.02473400
H	1.05768800	-5.78695800	-1.97306600
C	2.54241800	-4.82881700	-3.19404600
H	2.36032600	-5.47355600	-4.05700000
C	3.49505300	-3.81829200	-3.26658800
H	4.04782000	-3.67441300	-4.19596300
C	3.74422600	-2.97506300	-2.18152300
C	1.19670800	-4.41177700	0.33528100
H	1.60715000	-3.79025300	1.14336700
C	1.21550100	-5.86333000	0.81156400
H	0.72365500	-6.53979600	0.09460700
H	0.68133700	-5.95558200	1.76984200
H	2.24224400	-6.23145200	0.95968900
C	-0.23522900	-3.93436200	0.09154700
H	-0.27109300	-2.86353200	-0.15753000
H	-0.84459600	-4.08721900	0.99432400
H	-0.69956600	-4.49255600	-0.73805900
C	4.75530300	-1.84942600	-2.30936500
H	4.41921300	-1.04760800	-1.63246800
C	6.15372200	-2.26216100	-1.84548300
H	6.52264200	-3.12320300	-2.42557500
H	6.17310900	-2.53320300	-0.78124300
H	6.86405100	-1.43186900	-1.98514100
C	4.80982500	-1.27787000	-3.72368400
H	3.80847100	-1.00857400	-4.09184600
H	5.25291500	-1.99021500	-4.43660200
H	5.43396400	-0.37414500	-3.74789100
C	3.75669600	1.67813600	1.38878600
C	2.85593500	2.50073200	2.10223700
C	3.11081200	3.87432800	2.14577200
H	2.42716400	4.52352700	2.69248700
C	4.20602600	4.43129100	1.49704400
H	4.38470500	5.50761300	1.55029000
C	5.04494700	3.61912400	0.74760800
H	5.87681300	4.06533100	0.19775900
C	4.83460000	2.23993900	0.66979900
C	1.65956400	1.93021800	2.83924500
H	1.25051500	1.12133300	2.21068700
C	2.06458000	1.30647400	4.17531600
H	2.52920500	2.06440900	4.82668600
H	2.76508800	0.47074200	4.04912900
H	1.17930700	0.90608400	4.69242600
C	0.53896500	2.94637100	3.03601900
H	0.80819600	3.70183800	3.79206100
H	-0.35818800	2.43377500	3.41413200
H	0.27973900	3.46265000	2.09846400
C	5.72620600	1.41586400	-0.24120600
H	5.52782200	0.35076700	-0.04966200
C	7.21741600	1.65535300	-0.00919900
H	7.51506200	2.67739800	-0.29047100
H	7.81509700	0.96195500	-0.62106300
H	7.50298500	1.50726500	1.04289400
C	5.36142800	1.69721500	-1.69909000
H	4.31410700	1.43645500	-1.91017300
H	6.00706500	1.12315500	-2.38010700
H	5.48542200	2.76519500	-1.93604200
C	-3.45400900	-1.33646500	0.23642100
C	-4.53240000	-2.34250400	0.36087900
C	-5.86582800	-1.92507400	0.42115800
H	-6.10486300	-0.86148700	0.39267300
C	-6.88227100	-2.86809400	0.52653700
H	-7.91994200	-2.53136100	0.58072900
C	-6.57309900	-4.22847600	0.55885100
H	-7.37290700	-4.96840100	0.63704500
C	-5.24430500	-4.64423400	0.48970900

H	-5.00031400	-5.70859400	0.51012900
C	-4.21965000	-3.70394700	0.39473900
H	-3.17790600	-4.02547400	0.33553900
C	-2.75429800	-1.11405400	2.68510700
C	-4.18279600	-1.09873900	3.23635800
H	-4.73805100	-0.20196900	2.93734900
H	-4.13300300	-1.11801200	4.33484800
H	-4.75661100	-1.97801800	2.91694600
C	-2.12597300	-2.46943400	3.01220000
H	-2.72533500	-3.28760800	2.58850800
H	-2.09520700	-2.60749400	4.10332600
H	-1.10009400	-2.52657000	2.63041300
C	-1.93777500	0.00679300	3.32445800
H	-2.39327400	0.99116500	3.13902700
H	-0.90906100	0.00513600	2.93462800
H	-1.87741800	-0.13766500	4.41224600
C	-3.29795800	-1.07208000	-2.29843300
C	-4.73457700	-1.49833700	-2.59992500
H	-5.45128900	-0.74848900	-2.23432900
H	-4.99088500	-2.47339200	-2.16648700
H	-4.85198600	-1.57657400	-3.69090000
C	-2.98742200	0.17669900	-3.12377300
H	-2.84422400	-0.09804800	-4.17836300
H	-2.08204700	0.69629500	-2.78053600
H	-3.81883300	0.88931700	-3.06842900
C	-2.32727300	-2.20642700	-2.64144800
H	-2.40356400	-2.46683400	-3.70757800
H	-2.56272400	-3.10592500	-2.05276300
H	-1.29175900	-1.91455800	-2.42169300
C	0.94101000	-0.01635300	-0.73921800
H	1.04153600	1.07874200	-0.68150400
C	1.36751100	-0.46634900	-2.12943700
H	1.27315400	-1.56066300	-2.22559000
H	2.45169600	-0.26374300	-2.17753100
C	0.69221800	0.23016500	-3.30116700
H	-0.32757200	-0.14353100	-3.45538300
H	0.62771500	1.31604400	-3.13191100
H	1.24643700	0.03769100	-4.23337000
C	-2.54544300	2.08624500	0.16417700
O	-1.97220100	2.73516100	-0.89790000
H	-2.24846000	2.50045100	1.14940700
C	-4.03876600	2.06649800	0.08569000
C	-4.69032600	2.36330000	-1.11835800
C	-4.80842500	1.74289600	1.20961200
C	-6.06771000	2.22375400	-1.23133900
H	-4.08141300	2.70714800	-1.95433100
C	-6.18305000	1.58330300	1.11306900
H	-4.31453400	1.61219100	2.17317900
C	-6.79127700	1.79076200	-0.12283400
H	-6.59582700	2.42689000	-2.16351100
H	-6.79485300	1.29424000	1.96815200
N	-8.22241100	1.52814500	-0.25814800
O	-8.77484700	1.91271900	-1.26448800
O	-8.76348400	0.91766000	0.64244400
B	-0.53396300	3.03052800	-0.63295400
O	0.14718500	3.12349300	-1.89844200
O	-0.28371100	4.26645400	0.07321300
C	0.88535900	4.32713600	-1.93549600
C	0.13485600	5.21933600	-0.88298200
C	2.31920600	4.01495700	-1.51650500
H	2.71398000	3.23570000	-2.18494000
H	2.98479600	4.88900600	-1.57939300
H	2.34532100	3.63007600	-0.48817400
C	0.87046000	4.87809600	-3.35300900
H	1.35018100	5.86838200	-3.40260800
H	1.42302200	4.19918200	-4.02062500
H	-0.15566700	4.96146200	-3.73377900
C	1.01029100	6.25775900	-0.20116200
H	1.43048300	6.96792500	-0.93074500

H	0.40957500	6.82828200	0.52345400
H	1.83481000	5.77976900	0.34339100
C	-1.10304100	5.89308600	-1.47846900
H	-1.72906900	5.15663100	-2.00175700
H	-1.69829300	6.31300400	-0.65399200
H	-0.84614300	6.70752800	-2.17296100
H	-0.18199500	2.06972000	0.13412900

Cartesian coordinates of the optimized geometry of TS-**3a** at PBE0-D3BJ/def2-SVP level of theory:

Ga	-2.60809200	-0.00895300	-0.47672600
Cl	-1.97269200	-0.82120500	-2.45963800
Si	1.36896000	-0.98495200	0.12768300
O	-0.21839600	-0.88061300	0.69911000
N	-4.14752500	-1.14084200	0.00368600
N	-3.69480200	1.54837400	-1.01607600
N	1.71981300	-2.04652900	-1.34036300
N	1.35316600	-2.92005900	0.60265400
C	-5.27762500	-1.01050500	-0.68551900
C	-5.57214900	0.10267400	-1.49128400
H	-6.51756000	0.05494300	-2.02957600
C	-4.89042100	1.32558900	-1.56805700
C	-6.31296100	-2.09878400	-0.64097300
H	-7.32151700	-1.68910100	-0.77735000
H	-6.27132300	-2.67526800	0.29123300
H	-6.12037800	-2.79867900	-1.46978200
C	-5.56853300	2.43751800	-2.31812500
H	-5.57926700	3.36943600	-1.73621000
H	-6.59652000	2.16469100	-2.58264500
H	-5.01266600	2.65411700	-3.24273900
C	-3.96597600	-2.21130600	0.93348300
C	-3.51070400	-3.46864900	0.49018600
C	-3.28846000	-4.46687000	1.44502500
H	-2.92822500	-5.44547000	1.11837500
C	-3.51259900	-4.23604900	2.79513100
H	-3.32444900	-5.02608000	3.52576300
C	-3.97824000	-2.99423100	3.21612700
H	-4.15829800	-2.82295900	4.27859600
C	-4.21683700	-1.96439400	2.30318700
C	-3.25968900	-3.76879500	-0.97356100
H	-3.53361600	-2.87773100	-1.55515500
C	-4.10365000	-4.94389100	-1.46820800
H	-3.81049200	-5.88536200	-0.97687100
H	-3.96774100	-5.08247200	-2.55206500
H	-5.17616900	-4.79447400	-1.27481700
C	-1.77823300	-4.02310400	-1.23578600
H	-1.15140800	-3.21819200	-0.82763300
H	-1.58733100	-4.08257600	-2.31814400
H	-1.46344100	-4.97481700	-0.77930800
C	-4.77604100	-0.63108300	2.76613500
H	-4.36013700	0.13544800	2.09440200
C	-6.29839700	-0.57943500	2.61475300
H	-6.77572000	-1.37885400	3.20353900
H	-6.61567900	-0.69240200	1.57012700
H	-6.68792400	0.38572700	2.97486900
C	-4.38372600	-0.26398600	4.19353200
H	-3.30008900	-0.34639800	4.35879400
H	-4.89042200	-0.90319100	4.93355300
H	-4.68253700	0.77275600	4.41049500
C	-3.20178800	2.88933900	-0.97291300
C	-2.50375800	3.44114700	-2.06729300
C	-2.12873700	4.78839800	-2.00283900
H	-1.59189300	5.23126300	-2.84499400
C	-2.44133700	5.57500900	-0.90266500
H	-2.15640300	6.62950300	-0.88220400
C	-3.09742900	5.00772200	0.18494900
H	-3.31805300	5.62309700	1.05961500

C	-3.47233400	3.66245500	0.17927700
C	-2.14044900	2.62995700	-3.29486400
H	-2.66163300	1.66373100	-3.23156900
C	-2.55873200	3.31089900	-4.59745800
H	-1.98526500	4.23278800	-4.78254400
H	-3.62577900	3.58158200	-4.59937200
H	-2.37860100	2.63846000	-5.44988600
C	-0.64448900	2.31960800	-3.30338400
H	-0.04285900	3.24300700	-3.33251900
H	-0.37898900	1.70906800	-4.17896400
H	-0.35703400	1.74489300	-2.41267800
C	-4.18481100	3.06318000	1.37608700
H	-4.02028700	1.97624500	1.32449900
C	-5.69689600	3.28366700	1.31411300
H	-5.93595900	4.35861400	1.27786300
H	-6.18550100	2.85792500	2.20462100
H	-6.14444000	2.80494000	0.43197500
C	-3.61719300	3.55785900	2.70402200
H	-2.52086100	3.46938600	2.73569100
H	-4.02962400	2.96897500	3.53724300
H	-3.87499400	4.61127500	2.89473000
C	1.76239500	-3.18445200	-0.61506300
C	2.31428500	-4.48722000	-1.07570700
C	3.65434200	-4.77214800	-0.79138200
H	4.27000200	-4.01899300	-0.29457400
C	4.20356100	-5.99801900	-1.15833200
H	5.25180200	-6.20943200	-0.93560800
C	3.41911100	-6.94809500	-1.81087800
H	3.84957000	-7.91038900	-2.09756200
C	2.08486500	-6.66505200	-2.09927700
H	1.46543900	-7.40521300	-2.61088400
C	1.53301900	-5.43933100	-1.73498100
H	0.48632000	-5.22379600	-1.94921600
C	2.14116800	-1.83536400	-2.73898000
C	3.62931400	-2.14731200	-2.92195900
H	4.21082100	-1.59832200	-2.16666200
H	3.95425800	-1.81587000	-3.92008100
H	3.84180600	-3.22195200	-2.84458200
C	1.27202900	-2.67544700	-3.67877700
H	1.43933300	-3.75170700	-3.54350600
H	1.51889200	-2.43312700	-4.72345000
H	0.20812900	-2.45071800	-3.51158600
C	1.92305200	-0.36386400	-3.07568800
H	2.64540600	0.26625300	-2.54005400
H	0.89211200	-0.06472100	-2.84488900
H	2.09029200	-0.21503400	-4.15272500
C	1.23133200	-3.76197800	1.79517000
C	2.57742500	-3.87054500	2.52223000
H	2.95323600	-2.88209400	2.82022000
H	3.33378300	-4.35412500	1.88778900
H	2.46335500	-4.47738900	3.43351000
C	0.21628000	-3.08189400	2.71657100
H	0.09723800	-3.66995400	3.63876500
H	-0.75984800	-2.98898200	2.22256300
H	0.54723200	-2.07101800	2.99009100
C	0.71980200	-5.17053000	1.48163100
H	0.48509700	-5.68321900	2.42646000
H	1.45912300	-5.77972100	0.94660700
H	-0.19998900	-5.12377100	0.88397800
C	-1.08036400	0.23125400	0.77931900
H	-0.56351100	1.13616300	0.41932100
C	-1.48998800	0.44233200	2.23033200
H	-1.90644800	-0.50786200	2.59960700
H	-2.30877000	1.17692200	2.27290100
C	-0.36228800	0.89483200	3.14400100
H	0.50005500	0.21703900	3.07885700
H	-0.00776900	1.90393800	2.87999300
H	-0.68402400	0.92480500	4.19655300
C	3.13686100	-0.73395000	0.88322500

O	3.91751500	-0.16933000	-0.13788300
H	3.50234800	-1.77193000	0.98285200
C	3.25863300	-0.11164400	2.26913600
H	2.91662600	0.93439400	2.25496800
H	2.56279000	-0.64828000	2.94076800
C	4.66866800	-0.18088800	2.84169200
H	4.96515000	-1.24147000	2.94881800
H	5.36481400	0.26986600	2.11748200
H	1.30440800	0.50615800	-0.48116900
C	5.76871700	1.95078900	-1.93804500
C	6.16818700	2.51122500	-0.52745800
O	5.45851100	1.65301500	0.34273600
O	4.43179500	1.53815700	-1.72430200
C	7.65092600	2.42485100	-0.20552100
H	8.25254200	2.99427200	-0.93110000
H	7.83596000	2.84353900	0.79531000
H	7.99250400	1.38175900	-0.20047900
C	5.68085000	3.94628600	-0.31948300
H	4.62722100	4.04896400	-0.61477700
H	5.75744900	4.18822700	0.75078000
H	6.27668200	4.67623100	-0.88776000
C	6.58982000	0.71929900	-2.31793300
H	6.11836200	0.24005600	-3.18856300
H	7.62933200	0.97049200	-2.57732400
H	6.59176000	-0.00760300	-1.49296600
C	5.80633800	2.96945900	-3.06428900
H	6.81889500	3.38141200	-3.19627800
H	5.50864400	2.48778900	-4.00796200
H	5.11035400	3.79771500	-2.87600300
B	4.29255300	1.18796600	-0.33838000
C	2.02322100	2.15782200	-0.48200500
H	2.09887700	1.96393000	-1.56333500
O	3.05062700	2.04670800	0.22321500
C	0.94325300	3.03760300	0.05380500
H	-0.00734200	2.88677800	-0.47444900
H	0.80184600	2.78237700	1.11374300
C	1.36607200	4.50958900	-0.04981800
H	1.23332700	4.85722200	-1.08887300
C	0.56483700	5.38431600	0.90238200
H	-0.50583400	5.14321400	0.80154400
H	0.83836500	5.12406200	1.94098600
C	0.76505100	6.87554000	0.68120200
H	1.83890600	7.11913300	0.76397400
H	0.48270200	7.12445200	-0.35755900
C	-0.03449300	7.73350400	1.64847400
H	-1.11439200	7.52911500	1.56205500
H	0.25244400	7.53070700	2.69274400
H	0.11727500	8.80786700	1.46450500
H	2.43991700	4.60171700	0.17992500
C	4.80761800	0.52699100	4.17992700
H	4.09983200	0.09511600	4.91314300
H	4.50471100	1.58300600	4.05724300
C	6.21820300	0.47751700	4.75006300
H	6.91496800	0.90858400	4.00990800
H	6.52472000	-0.57738400	4.86791400
C	6.36379300	1.20356100	6.07771700
H	7.39402800	1.15182000	6.46265000
H	6.09891700	2.26896100	5.97965700
H	5.70025200	0.77175400	6.84489800

Cartesian coordinates of the optimized geometry of TS-**3b** at PBE0-D3BJ/def2-SVP level of theory:

Ga	2.57589600	0.52558100	-0.75195400
Cl	1.94843500	1.81111200	-2.46478900
Si	-1.49067200	0.93736500	0.21973200
O	0.10342100	0.70036700	0.72507600
N	4.00774200	1.61091400	0.09010600

N	3.83630300	-0.71233300	-1.64429800
N	-1.82923000	2.32203500	-0.93557900
N	-1.54535600	2.65564900	1.18161600
C	5.12293900	1.81453800	-0.60896700
C	5.50454100	1.03330700	-1.71196200
H	6.41168800	1.35221500	-2.22272300
C	4.97165300	-0.19325100	-2.12538900
C	6.04463200	2.94611500	-0.24889900
H	7.08341200	2.70138500	-0.50283000
H	5.97928200	3.22162300	0.81029900
H	5.75627100	3.83137100	-0.83808900
C	5.74100400	-0.95210400	-3.16998700
H	5.92786700	-1.99042800	-2.86426400
H	6.69750700	-0.46285600	-3.38610500
H	5.15231600	-1.00273900	-4.09840800
C	3.77342600	2.35059300	1.29199000
C	3.20005700	3.63625500	1.22933800
C	2.94699600	4.30999700	2.42883300
H	2.49513300	5.30446000	2.39670800
C	3.26109400	3.74061200	3.65522300
H	3.04941000	4.27946000	4.58158900
C	3.85432900	2.48332600	3.70027000
H	4.11366300	2.04894700	4.66711200
C	4.12954700	1.76941800	2.53100100
C	2.87099100	4.31062700	-0.08677600
H	3.22216800	3.66172000	-0.90080800
C	3.56243600	5.66730900	-0.22433400
H	3.17491400	6.39487000	0.50641400
H	3.38669500	6.08476200	-1.22787800
H	4.64909400	5.59580000	-0.06965900
C	1.36458500	4.45705900	-0.26471500
H	0.84629500	3.49669700	-0.13909700
H	1.13282200	4.83681900	-1.27093400
H	0.95896800	5.16664200	0.47308400
C	4.85064200	0.43474800	2.59761900
H	4.51859200	-0.15027500	1.72740800
C	6.36515000	0.61144600	2.46344100
H	6.75676900	1.25643000	3.26604200
H	6.64633900	1.05936900	1.50144000
H	6.87207700	-0.36370100	2.53434900
C	4.52747000	-0.36816700	3.85396200
H	3.44430900	-0.46069100	4.01854000
H	4.96865100	0.08640800	4.75466200
H	4.94560900	-1.38241000	3.77178700
C	3.55660300	-2.08059300	-1.95116200
C	2.86355300	-2.42486100	-3.13076000
C	2.70827500	-3.78178400	-3.43580600
H	2.17482700	-4.06545900	-4.34608900
C	3.24554900	-4.77237100	-2.62219300
H	3.13038800	-5.82544200	-2.88871600
C	3.91563900	-4.41880100	-1.45562200
H	4.30930300	-5.20187200	-0.80500900
C	4.06486800	-3.07857200	-1.08851200
C	2.30570400	-1.37692200	-4.07413100
H	2.71837200	-0.40187100	-3.77751100
C	2.69632900	-1.62756900	-5.52996000
H	2.21685900	-2.53177400	-5.93661800
H	3.78379800	-1.74959400	-5.64893700
H	2.37982400	-0.78041200	-6.15717500
C	0.78880100	-1.26756500	-3.93517800
H	0.29265400	-2.21831700	-4.19178500
H	0.39207100	-0.48652800	-4.59980000
H	0.50333800	-0.98873800	-2.91089000
C	4.79478200	-2.72440400	0.19328700
H	4.55043300	-1.67494500	0.41598900
C	6.31312100	-2.80125700	0.02604900
H	6.62631200	-3.81583600	-0.26768900
H	6.81727400	-2.55399800	0.97339800
H	6.67810400	-2.09838000	-0.73608700

C	4.33461900	-3.57613100	1.37427600
H	3.24132800	-3.55526800	1.48873300
H	4.78265700	-3.20504500	2.30838500
H	4.62708300	-4.63100200	1.26642000
C	-1.95896400	3.21643000	0.06724900
C	-2.65976900	4.52420000	-0.03716500
C	-4.03231800	4.51936200	0.23823400
H	-4.53320200	3.57288100	0.45579200
C	-4.75620000	5.70803600	0.20559000
H	-5.82742900	5.69474800	0.41820500
C	-4.11578100	6.90726600	-0.10398000
H	-4.68326200	7.84037000	-0.12901300
C	-2.75053400	6.91200000	-0.38578400
H	-2.24473300	7.84862700	-0.63068200
C	-2.02231100	5.72517600	-0.35384400
H	-0.95249100	5.73385600	-0.56054300
C	-2.12021400	2.50034400	-2.37349700
C	-3.60576700	2.77137000	-2.61630200
H	-4.21068100	2.01014300	-2.10739700
H	-3.81238000	2.72013400	-3.69592500
H	-3.90290900	3.77027300	-2.26947200
C	-1.26282800	3.63232800	-2.94442600
H	-1.53095800	4.60675400	-2.51662600
H	-1.42592600	3.69595600	-4.03067600
H	-0.19685200	3.43401800	-2.76698500
C	-1.72538400	1.20401500	-3.07277800
H	-2.41866400	0.39320800	-2.81541300
H	-0.68877500	0.94109800	-2.82436100
H	-1.78434400	1.34951700	-4.16166500
C	-1.47260600	3.15046100	2.56112500
C	-2.76522400	2.82976500	3.32052400
H	-2.95323600	1.74810000	3.36626800
H	-3.63102500	3.32058900	2.85157600
H	-2.69000100	3.19700200	4.35515200
C	-0.29541600	2.43151000	3.22541100
H	-0.19018300	2.77021600	4.26659900
H	0.64146200	2.63498100	2.69016800
H	-0.44412400	1.34473300	3.23147000
C	-1.21676400	4.65690700	2.64468300
H	-0.99705600	4.91727700	3.69051600
H	-2.08206900	5.25199800	2.32769800
H	-0.34864600	4.94338500	2.03659500
C	1.09909300	-0.24049900	0.37621900
H	0.65001700	-1.06841900	-0.20094700
C	1.77135500	-0.80602900	1.61904700
H	2.25825000	0.02355000	2.15298000
H	2.58265400	-1.46248200	1.26577400
C	0.90934100	-1.58017300	2.60200700
H	0.29614500	-0.90647900	3.21524400
H	0.23624400	-2.28474200	2.09506000
H	1.53806200	-2.15928000	3.29590400
C	-3.33483200	0.50689300	0.79753400
O	-4.14685000	0.39541800	-0.32785400
H	-3.63304300	1.45138000	1.28583100
H	-1.46610100	-0.43454400	-0.67230300
C	-5.48934900	-1.05550300	-3.13262500
C	-6.21025500	-1.92469700	-2.04041700
O	-5.75970500	-1.32198800	-0.84192800
O	-4.27350300	-0.70419400	-2.48949300
C	-7.72806200	-1.86362100	-2.08167000
H	-8.11597600	-2.21772100	-3.04925600
H	-8.14392900	-2.50694600	-1.29191400
H	-8.08795300	-0.84184800	-1.90632600
C	-5.76102000	-3.38676100	-2.06523100
H	-4.66632100	-3.47201700	-2.09736300
H	-6.10663600	-3.87282800	-1.14118700
H	-6.17873700	-3.93308400	-2.92381500
C	-6.25423100	0.22965900	-3.43869600
H	-5.61509600	0.88611000	-4.04610000

H	-7.18282300	0.03763600	-3.99635100
H	-6.50151400	0.76215400	-2.50908200
C	-5.17638500	-1.79051800	-4.42487800
H	-6.09565600	-2.15574800	-4.90817700
H	-4.67170500	-1.10834900	-5.12556600
H	-4.50997400	-2.64500700	-4.24749200
B	-4.45816900	-0.79230800	-1.07260900
C	-2.24120600	-1.95684300	-1.00562300
H	-2.05122500	-1.65577700	-2.04756900
O	-3.39780200	-1.85252100	-0.52948600
C	-3.51125400	-0.54047400	1.86339600
C	-2.45409800	-0.84069300	2.72843800
C	-4.73983400	-1.19620200	2.04836700
C	-2.58753300	-1.77618300	3.74692000
H	-1.48675700	-0.36194300	2.57103400
C	-4.89046800	-2.13591300	3.05814200
H	-5.56225000	-1.00165200	1.35764300
C	-3.80996400	-2.42052400	3.89363300
H	-1.75970600	-2.03617600	4.40677900
H	-5.83061200	-2.66666500	3.20972100
C	-1.36481400	-2.95309200	-0.35624800
C	-1.81361700	-3.56945100	0.82164300
C	-0.14658300	-3.33497400	-0.92664400
C	-1.03864300	-4.53710600	1.44561900
H	-2.77934000	-3.27639500	1.23438500
C	0.63660300	-4.30317900	-0.31229000
H	0.19784000	-2.87750300	-1.85478500
C	0.18106700	-4.87935700	0.86819000
H	-1.35302200	-5.01845900	2.37199400
H	1.59073600	-4.61157500	-0.73391100
N	1.02286100	-5.88804500	1.52888300
O	0.60200600	-6.38518800	2.54719900
O	2.08711300	-6.15304400	1.01059300
N	-3.95503700	-3.44064200	4.93195200
O	-5.05444600	-3.92299200	5.09687100
O	-2.96308800	-3.74492600	5.56132800

Cartesian coordinates of the optimized geometry of TS-**4a** at PBE0-D3BJ/def2-SVP level of theory:

Ga	2.83100900	-0.18674500	-0.75126100
Cl	2.25475200	0.71048800	-2.69929500
Si	-1.07349500	1.08696500	0.28479400
O	0.51976000	0.84764000	0.55993900
N	4.62576600	0.55465200	-0.48120700
N	3.44592700	-1.97319800	-1.26710300
N	-1.43766400	2.36646000	-0.96338900
N	-1.52466400	2.59908400	1.18038600
C	5.53955000	0.19689800	-1.39064700
C	5.45587700	-0.97847100	-2.15087100
H	6.26087400	-1.13716700	-2.86706800
C	4.55432100	-2.05110600	-1.99849700
C	6.70014900	1.11305800	-1.65218400
H	6.34803200	1.92292300	-2.31320500
H	7.52360100	0.59232400	-2.15515600
H	7.06884500	1.58968500	-0.73543000
C	4.86893600	-3.32843100	-2.72055800
H	4.23017900	-3.40669900	-3.61440500
H	4.64845600	-4.20660300	-2.09874800
H	5.91729600	-3.35739800	-3.03955300
C	4.93173900	1.53746600	0.50993500
C	4.49351600	2.86864100	0.36579100
C	4.77084100	3.77773400	1.39273400
H	4.43060300	4.81132600	1.29237300
C	5.47482600	3.39586000	2.52598100
H	5.68662900	4.12223500	3.31401900
C	5.90743500	2.08032100	2.65329600
H	6.45710700	1.78261700	3.54849100

C	5.63992800	1.12984800	1.66585700
C	3.73645700	3.34738100	-0.85466500
H	3.69061200	2.51824700	-1.57393800
C	4.44957800	4.50853900	-1.54651400
H	4.47434700	5.41110700	-0.91516400
H	3.92968500	4.77211100	-2.48047300
H	5.49009900	4.25141900	-1.79724600
C	2.29532700	3.71266700	-0.49821800
H	1.75280400	2.85877200	-0.06676600
H	1.74827500	4.03173800	-1.39782600
H	2.26361400	4.53835200	0.23197100
C	6.10650100	-0.30241400	1.85564300
H	5.50148200	-0.92760300	1.18165600
C	7.57084900	-0.48810800	1.45225000
H	8.22839200	0.15290800	2.06126300
H	7.74400900	-0.24032400	0.39689300
H	7.88239800	-1.53355500	1.60331600
C	5.88962200	-0.80257000	3.28240500
H	4.85197400	-0.65337000	3.61580200
H	6.54869000	-0.29099800	4.00041800
H	6.12080200	-1.87625300	3.34855500
C	2.64081500	-3.12762400	-1.01306200
C	1.65892500	-3.53672800	-1.93764300
C	0.89561800	-4.67020400	-1.63499100
H	0.12417600	-4.99313100	-2.33784800
C	1.09431000	-5.38426300	-0.46208700
H	0.48979400	-6.26822600	-0.24713800
C	2.05177300	-4.95526700	0.45085300
H	2.18961400	-5.50930200	1.38070800
C	2.82544400	-3.81896000	0.20552500
C	1.36837300	-2.77349200	-3.21327600
H	2.12140700	-1.97864500	-3.31728800
C	1.44145400	-3.66112200	-4.45529900
H	0.64752000	-4.42424500	-4.45738400
H	2.40477600	-4.18849600	-4.53148500
H	1.31297700	-3.05381100	-5.36423300
C	0.00666900	-2.08856500	-3.11362800
H	-0.80993200	-2.82126100	-3.02173000
H	-0.18626900	-1.47057800	-4.00262900
H	-0.04564900	-1.42691500	-2.23899900
C	3.84791200	-3.35242400	1.22281300
H	3.95526100	-2.26552700	1.08255600
C	5.22238600	-3.97223000	0.96665200
H	5.16889900	-5.07143300	1.01506100
H	5.94968500	-3.63531100	1.72175800
H	5.61832100	-3.69534000	-0.02048900
C	3.40125000	-3.58734500	2.66230900
H	2.38862800	-3.19654500	2.83936600
H	4.08552800	-3.08696700	3.36181800
H	3.39873600	-4.65681500	2.92384100
C	-1.75579700	3.21724800	0.02328200
C	-2.24634300	4.60327300	-0.13208500
C	-3.61631400	4.87169300	-0.05814200
H	-4.30464500	4.04076400	0.12780700
C	-4.05498000	6.18720400	-0.17971800
H	-5.12405800	6.40440900	-0.12446400
C	-3.13960400	7.22447600	-0.36043400
H	-3.49244700	8.25457300	-0.45110600
C	-1.77394400	6.95008900	-0.42130400
H	-1.05512100	7.76097700	-0.55823500
C	-1.32269100	5.63677800	-0.31043400
H	-0.25496300	5.41115400	-0.35922300
C	-1.71261900	2.46039700	-2.41833100
C	-3.20080600	2.68274500	-2.68639600
H	-3.80332300	1.86533100	-2.26572900
H	-3.36465500	2.68867500	-3.77454800
H	-3.55098600	3.64730200	-2.29565800
C	-0.86656500	3.57964100	-3.02777700
H	-1.14723700	4.56993300	-2.64411700

H	-1.01459200	3.58995400	-4.11757900
H	0.20116900	3.40200000	-2.83266800
C	-1.29002800	1.13080400	-3.02917200
H	-1.89636600	0.31869700	-2.60536400
H	-0.22231600	0.94378500	-2.85500800
H	-1.45196400	1.16112400	-4.11643300
C	-1.50716000	3.03402400	2.58069300
C	-2.48659700	4.16723300	2.87506500
H	-3.49740900	3.88475500	2.55260000
H	-2.19745200	5.11049700	2.39393200
H	-2.49678700	4.33634100	3.96225000
C	-1.93923600	1.81430900	3.39426600
H	-1.87136000	2.02421100	4.47130400
H	-1.30530100	0.93965100	3.18272800
H	-2.97769500	1.56572800	3.13383900
C	-0.07714300	3.46305500	2.92233900
H	0.00695100	3.71368500	3.99055900
H	0.20378900	4.35271100	2.33779500
H	0.64259100	2.66604700	2.68931600
C	1.52897700	-0.13573000	0.75853000
H	1.06370000	-1.13580100	0.82238500
C	2.29005600	0.14958500	2.04917600
H	2.65074800	1.19052100	2.01732600
H	3.20182700	-0.46996000	2.03771900
C	1.55097700	-0.10436800	3.35157100
H	0.63708000	0.49926500	3.42815500
H	1.26375700	-1.16143000	3.45226100
H	2.18416800	0.15347000	4.21459200
C	-2.31157500	-0.33989900	0.31085400
O	-2.99141800	-0.22931700	-0.89335200
H	-2.98952800	-0.08372100	1.15437000
B	-4.41601200	-0.57073100	-0.87774800
O	-5.01015400	-0.17435300	-2.12052200
O	-4.72964500	-1.97926100	-0.68302300
C	-5.83645900	-1.22150000	-2.57835700
C	-5.17155300	-2.48511000	-1.92583200
C	-7.25644400	-0.98922300	-2.05933700
H	-7.57296900	0.02164700	-2.35653900
H	-7.97848300	-1.71439400	-2.46470400
H	-7.27960100	-1.04370700	-0.96186000
C	-5.83927900	-1.22103300	-4.09906300
H	-6.38588600	-2.08882600	-4.50074700
H	-6.33066300	-0.30743800	-4.46672800
H	-4.81482800	-1.23097700	-4.49369100
C	-6.11761400	-3.64954900	-1.68255200
H	-6.55973800	-4.01251400	-2.62379800
H	-5.56594900	-4.48275400	-1.22076400
H	-6.92802600	-3.36488100	-0.99826500
C	-3.95386500	-2.96078100	-2.72265000
H	-3.28205800	-2.11715800	-2.93546300
H	-3.39915300	-3.69048200	-2.11276700
H	-4.23395500	-3.44403200	-3.67134200
H	-4.88929500	0.06492200	0.14561400
C	-5.43609500	1.29763200	1.15084400
O	-4.56913800	2.16892000	1.24798800
H	-6.26023500	1.40857700	0.40735500
C	-1.76585500	-1.74372400	0.57068200
H	-2.56389300	-2.42601800	0.23704900
H	-0.89126400	-1.95769700	-0.06836800
C	-1.44788100	-2.00177700	2.03229900
H	-0.75058100	-1.23633800	2.41672100
H	-2.37417400	-1.87817600	2.62211100
C	-0.86321500	-3.38142600	2.28783400
H	0.09172500	-3.48289900	1.74150400
H	-1.53166700	-4.14057000	1.84332900
C	-0.64977500	-3.70067400	3.76067100
H	-1.60670400	-3.57840300	4.29809700
H	0.03258100	-2.95280500	4.20394500
C	-0.10344300	-5.09822500	4.00403200

H	0.87400800	-5.23486400	3.51524400
H	0.02976800	-5.30488900	5.07686200
H	-0.78223200	-5.86553100	3.59833600
C	-5.73447100	0.33971600	2.28810000
H	-4.77621300	0.01115100	2.72462600
H	-6.22494600	0.95234400	3.07031100
C	-6.60209500	-0.85199000	1.92339000
H	-7.57192000	-0.49353300	1.53290100
H	-6.11780400	-1.40036900	1.09838700
C	-6.84774900	-1.80581700	3.08185000
H	-7.35174400	-1.27804300	3.91334500
H	-5.87420300	-2.13966400	3.48705600
C	-7.66465300	-3.02784800	2.68508000
H	-8.63731500	-2.69578500	2.28015600
H	-7.15433200	-3.53879100	1.84978700
C	-7.89229600	-4.00531900	3.82659700
H	-8.42946100	-3.52759900	4.66229500
H	-8.48388300	-4.87690700	3.50677100
H	-6.93611400	-4.38154300	4.22556300

Cartesian coordinates of the optimized geometry of TS-**4b** at PBE0-D3BJ/def2-SVP level of theory:

Ga	3.03670900	0.10888300	-0.67222300
Cl	2.78944000	1.23970600	-2.56864100
Si	-1.04488700	1.28001800	0.07877300
O	0.54224800	1.03509300	0.32926100
N	4.70532700	0.83627200	0.04443400
N	3.80737500	-1.59319100	-1.26304000
N	-1.39225500	2.53163300	-1.19087100
N	-1.57822600	2.78638900	0.94526100
C	5.80539200	0.59947500	-0.67813300
C	5.92626400	-0.47787000	-1.56670900
H	6.87224900	-0.54541500	-2.10221500
C	5.05061000	-1.56954200	-1.73709300
C	6.96266600	1.55038400	-0.57320300
H	6.75053800	2.41328700	-1.22681400
H	7.89957600	1.08960700	-0.90832200
H	7.08631400	1.94308800	0.44373100
C	5.56325300	-2.74715300	-2.51335100
H	5.14787600	-2.71824800	-3.53278000
H	5.23755700	-3.69646300	-2.06758600
H	6.65699600	-2.73013300	-2.58425000
C	4.74476600	1.69065400	1.18834200
C	4.29928500	3.02467800	1.10649500
C	4.29001500	3.79749400	2.27312800
H	3.94265600	4.83245700	2.22196100
C	4.72392100	3.28027700	3.48577200
H	4.71436500	3.90122400	4.38459100
C	5.17202000	1.96506500	3.55038400
H	5.50944900	1.56159900	4.50705100
C	5.18334300	1.14610700	2.41944400
C	3.84552900	3.65091800	-0.19564000
H	3.98156900	2.90960200	-0.99510800
C	4.69005200	4.87259600	-0.55707200
H	4.55457700	5.69311800	0.16567200
H	4.40462700	5.25405100	-1.54965800
H	5.76294600	4.62850700	-0.58199000
C	2.35702900	3.99690100	-0.15686800
H	1.74096900	3.10752600	0.04029600
H	2.03839900	4.41943900	-1.12204000
H	2.14364400	4.74125000	0.62843600
C	5.65495200	-0.29264500	2.53428900
H	5.22731600	-0.83806200	1.67910900
C	7.17729300	-0.40720400	2.42736200
H	7.66906800	0.16304300	3.23178400
H	7.55568800	-0.02999700	1.46848500
H	7.49050200	-1.45908400	2.51734700

C	5.17045100	-0.97110300	3.81394000
H	4.08303100	-0.87247600	3.94581400
H	5.65623800	-0.55274200	4.70879700
H	5.41625200	-2.04324800	3.79107200
C	3.01086500	-2.78055000	-1.34291100
C	2.27540800	-3.07243200	-2.51013900
C	1.51362400	-4.24610800	-2.53705600
H	0.93195300	-4.48149300	-3.43143700
C	1.48015400	-5.11194000	-1.45233800
H	0.88350100	-6.02607700	-1.49655200
C	2.19295500	-4.80014500	-0.29909900
H	2.13686500	-5.46850600	0.56206400
C	2.95323100	-3.63098900	-0.21577500
C	2.24278000	-2.14768700	-3.70943000
H	2.94540200	-1.32208900	-3.52530000
C	2.66048000	-2.84811700	-5.00209000
H	1.94508400	-3.63607200	-5.28543500
H	3.65118500	-3.32009600	-4.91829500
H	2.69759300	-2.12545000	-5.83161800
C	0.85338700	-1.52945200	-3.85588700
H	0.10623000	-2.28961600	-4.13256500
H	0.85146400	-0.75155900	-4.63339000
H	0.51719200	-1.06204800	-2.92085400
C	3.71008400	-3.30454000	1.05617700
H	3.83046100	-2.21035500	1.07618200
C	5.11701700	-3.90412100	1.04392600
H	5.07232000	-5.00067200	0.95003800
H	5.64905000	-3.66689300	1.97854600
H	5.72084300	-3.51653200	0.21108000
C	2.95032800	-3.71142100	2.31505000
H	1.91690800	-3.33759400	2.30004000
H	3.44701300	-3.30686000	3.20858900
H	2.89947500	-4.80354100	2.43761400
C	-1.74966600	3.39391300	-0.23512900
C	-2.19895300	4.78986300	-0.42161200
C	-3.53883000	5.07671900	-0.69548700
H	-4.25259800	4.25178900	-0.77358200
C	-3.93202200	6.40661500	-0.82822600
H	-4.97874200	6.63814800	-1.03756100
C	-3.00268500	7.43707300	-0.69148800
H	-3.32029900	8.47694900	-0.79800400
C	-1.66674500	7.14245200	-0.41893900
H	-0.93551300	7.94710300	-0.31572100
C	-1.26115600	5.81816700	-0.28020300
H	-0.21504300	5.57807400	-0.07770500
C	-1.41309200	2.68539900	-2.66741100
C	-2.85070700	2.77692500	-3.17551100
H	-3.42414000	1.88869800	-2.88163000
H	-2.83518100	2.81449100	-4.27484600
H	-3.35875500	3.67952900	-2.81288200
C	-0.60209000	3.91855200	-3.07035400
H	-1.05732900	4.85688000	-2.72780500
H	-0.54257400	3.95913600	-4.16746200
H	0.42394500	3.84734800	-2.67981500
C	-0.74667000	1.45027400	-3.25730100
H	-1.34145400	0.55184800	-3.05688800
H	0.27158000	1.33179700	-2.86359600
H	-0.66771700	1.57286600	-4.34698400
C	-1.77090100	3.23435300	2.32947200
C	-2.89838600	4.25780800	2.46314700
H	-3.80101700	3.90062000	1.94709000
H	-2.63047100	5.24140000	2.05808900
H	-3.13028500	4.38649300	3.53054900
C	-2.16315400	1.99288600	3.12586900
H	-2.25434400	2.23353000	4.19438600
H	-1.40742200	1.20145800	3.02970600
H	-3.12580100	1.59822900	2.77372700
C	-0.44719300	3.80246600	2.84677300
H	-0.53584400	4.08263500	3.90710300

H	-0.16271200	4.70008300	2.27833400
H	0.36246800	3.06405000	2.74660700
C	1.47153200	-0.03205400	0.55020000
H	0.97675300	-0.99291200	0.33080200
C	1.93782300	-0.03428900	1.99862400
H	2.29330800	0.97669000	2.25618600
H	2.82913900	-0.67899400	2.05754600
C	0.92250800	-0.51782300	3.01699300
H	0.02849000	0.11763100	3.03162800
H	0.58850200	-1.54209300	2.79816000
H	1.34341400	-0.50820400	4.03391900
C	-2.24325000	-0.17248900	0.04857300
O	-2.27841400	-0.43577400	-1.31606600
H	-3.19736600	0.25433400	0.40869700
C	-1.91917100	-1.38342800	0.87862300
C	-1.14658000	-2.40722000	0.31721200
C	-2.39649800	-1.52563000	2.18704900
C	-0.80575800	-3.52543900	1.06318700
H	-0.83344500	-2.32298700	-0.72437000
C	-2.06429200	-2.64063300	2.94826700
H	-3.05481800	-0.76417300	2.60990500
C	-1.25122400	-3.61657400	2.37887000
H	-0.19424000	-4.32202800	0.64373200
H	-2.42292600	-2.77150200	3.96942600
N	-0.84757700	-4.77155900	3.18312600
O	-0.07894300	-5.56545800	2.67869300
O	-1.29217800	-4.85906900	4.30566300
B	-3.58974300	-0.76640600	-1.89067500
O	-3.55642800	-0.55965700	-3.30115500
O	-4.08373200	-2.09920900	-1.65161800
C	-4.23778700	-1.63299500	-3.92098700
C	-4.03654200	-2.79386100	-2.88568700
C	-5.70929500	-1.24858000	-4.07455700
H	-5.76547800	-0.28892600	-4.60912200
H	-6.28327400	-1.99759100	-4.64029800
H	-6.18153400	-1.11639200	-3.08971900
C	-3.62191400	-1.88469100	-5.28709000
H	-4.05058500	-2.78176600	-5.76034000
H	-3.81671800	-1.02450300	-5.94505800
H	-2.53341300	-2.00724200	-5.21149600
C	-5.11755000	-3.86075800	-2.90243100
H	-5.16534300	-4.36495200	-3.88023100
H	-4.89811100	-4.62038800	-2.13719400
H	-6.10480500	-3.43457500	-2.67954600
C	-2.65959700	-3.44648900	-3.01763700
H	-1.87425200	-2.67863500	-3.04990100
H	-2.48404100	-4.07594700	-2.13272500
H	-2.57866300	-4.07726700	-3.91608300
H	-4.40746600	0.05791100	-1.26142200
C	-5.33591300	1.17209200	-0.43853300
O	-4.71526500	2.22541300	-0.27442500
H	-6.02111600	1.03250900	-1.30359400
C	-5.61028500	0.27186000	0.73926700
C	-5.58422200	0.85361000	2.01316100
C	-5.88729300	-1.09426500	0.60547200
C	-5.80907800	0.08494500	3.14913500
H	-5.38526500	1.92386000	2.09007400
C	-6.10869800	-1.87469400	1.73315700
H	-5.83686200	-1.56417700	-0.37945400
C	-6.06096400	-1.27414300	2.98872300
H	-5.79069900	0.50800900	4.15363500
H	-6.29243300	-2.94655600	1.66205300
N	-6.26238800	-2.10125600	4.18342200
O	-6.55234600	-3.26481700	4.01714600
O	-6.12052900	-1.56702700	5.26292400

Cartesian coordinates of the optimized geometry of TS-**5a** at PBE0-D3BJ/def2-SVP level of theory:

Ga	-2.27064100	-0.61201100	-0.33787400
Cl	-1.94881300	-1.32139800	-2.42742300
Si	1.98628300	0.21813300	-0.33930600
O	0.45148800	-0.30121600	0.04271000
N	-3.03436600	-2.19977600	0.53149000
N	-3.99663600	0.32548100	-0.54579200
N	2.49874500	-0.62695700	-1.88435600
N	2.99898700	-1.25423100	0.11124500
C	-4.23995300	-2.61301500	0.13522200
C	-5.17112500	-1.78269600	-0.50191100
H	-6.11896500	-2.25030400	-0.76592900
C	-5.10326300	-0.39200300	-0.70956600
C	-4.63016900	-4.04721900	0.35402000
H	-4.16610400	-4.65665600	-0.43863500
H	-5.71704900	-4.18102400	0.29735000
H	-4.26021500	-4.43592000	1.31132300
C	-6.37336200	0.27683900	-1.15515500
H	-6.39668400	1.34082700	-0.88882700
H	-7.24844100	-0.22903100	-0.72815700
H	-6.45009400	0.20912400	-2.25165100
C	-2.25537200	-2.98862000	1.43181600
C	-1.31232900	-3.91485600	0.94537600
C	-0.53632300	-4.61882100	1.87307400
H	0.20144400	-5.33922700	1.51133200
C	-0.68946800	-4.42317400	3.23907300
H	-0.07144500	-4.98135200	3.94628200
C	-1.63362200	-3.51318500	3.70508500
H	-1.74823100	-3.36406500	4.78034500
C	-2.42821800	-2.78071200	2.82074800
C	-1.12032200	-4.16976600	-0.53580700
H	-1.87779200	-3.58624700	-1.07738600
C	-1.32140400	-5.64252800	-0.89123200
H	-0.54489400	-6.28065100	-0.43982800
H	-1.26822500	-5.78169500	-1.98217500
H	-2.29699500	-6.01842000	-0.54683700
C	0.24270700	-3.66969500	-1.01265800
H	0.40484400	-2.61745700	-0.73932400
H	0.31433700	-3.74723400	-2.10850800
H	1.05518300	-4.26798400	-0.56988200
C	-3.45680400	-1.79428300	3.34592500
H	-3.59307900	-1.03504200	2.56015000
C	-4.81960900	-2.45454500	3.56814200
H	-4.73972000	-3.28039500	4.29315300
H	-5.24081100	-2.85946800	2.63894200
H	-5.54036200	-1.72257000	3.96558300
C	-3.01174500	-1.07901800	4.61880500
H	-2.00734100	-0.64387900	4.51741600
H	-3.00079200	-1.75686500	5.48665900
H	-3.71419300	-0.26606300	4.85908900
C	-4.00086700	1.74572400	-0.69205400
C	-3.95839900	2.34238100	-1.96994100
C	-3.94300400	3.73988900	-2.04253000
H	-3.91085900	4.21830900	-3.02436700
C	-3.95921700	4.52730500	-0.89951300
H	-3.94328800	5.61660900	-0.98249700
C	-3.98951500	3.92396800	0.35361900
H	-3.99779800	4.54655000	1.24997300
C	-4.01103200	2.53381400	0.48140600
C	-3.93107400	1.53945800	-3.25705700
H	-4.00822900	0.47401800	-2.99610700
C	-5.10031700	1.90028600	-4.17530700
H	-5.00889600	2.92868300	-4.55932900
H	-6.07037400	1.82877000	-3.66168100
H	-5.12353900	1.22725100	-5.04627700
C	-2.60274500	1.71269400	-3.99273600
H	-2.44830900	2.75995700	-4.29861500
H	-2.58491500	1.08707600	-4.89816600

H	-1.75696200	1.40474700	-3.36551000
C	-4.09880700	1.88175500	1.84669400
H	-3.61000200	0.89991700	1.75581600
C	-5.55143100	1.61120500	2.24224000
H	-6.12117700	2.55205500	2.30427700
H	-5.59890100	1.11869000	3.22653700
H	-6.05725900	0.95696600	1.51859500
C	-3.37301500	2.65875500	2.93897300
H	-2.34509000	2.91177800	2.64264600
H	-3.31973500	2.05860400	3.85967900
H	-3.89450400	3.59488600	3.19311000
C	3.28236000	-1.44329900	-1.17564100
C	4.33368000	-2.33226200	-1.71770700
C	5.61312200	-1.80022000	-1.91411200
H	5.78863300	-0.76028000	-1.62236500
C	6.61450400	-2.61147000	-2.44148100
H	7.61538100	-2.20151700	-2.59543800
C	6.34629600	-3.94142400	-2.76693200
H	7.13632600	-4.57290300	-3.18047500
C	5.07084100	-4.46697800	-2.56357700
H	4.85840800	-5.50756400	-2.81905900
C	4.06054100	-3.66372700	-2.03936800
H	3.05427500	-4.06194700	-1.89494000
C	2.48005600	-0.28023900	-3.31008100
C	3.61126600	0.70016200	-3.62877000
H	3.49965800	1.61675700	-3.03321900
H	3.57769100	0.97192700	-4.69532500
H	4.59382600	0.25917900	-3.41343300
C	2.57939200	-1.52893000	-4.18614500
H	3.57440900	-1.99111200	-4.14981100
H	2.37842300	-1.24822300	-5.23056300
H	1.82942000	-2.27535500	-3.88354000
C	1.13081400	0.39311100	-3.55474300
H	1.04382500	1.29696200	-2.93488900
H	0.30135800	-0.28803300	-3.31949100
H	1.04905500	0.70017200	-4.60804200
C	3.56020400	-1.86167700	1.32304700
C	5.08623600	-1.74883600	1.36670100
H	5.38191400	-0.73306200	1.06747900
H	5.56467800	-2.46657000	0.68634200
H	5.44265100	-1.96515400	2.38538400
C	2.95597100	-1.08713600	2.49012100
H	3.32878200	-1.48274300	3.44552300
H	1.86046800	-1.17411000	2.48441200
H	3.23280300	-0.02445500	2.43098600
C	3.12020000	-3.32331400	1.42240000
H	3.50410500	-3.76418500	2.35512700
H	3.51477600	-3.91600300	0.58480400
H	2.02373300	-3.39877100	1.42785800
C	-0.72871100	0.29942100	0.54867400
H	-0.75353500	1.37201200	0.28592800
C	-0.78231200	0.16598700	2.06708100
H	-0.56500200	-0.88534200	2.31742000
H	-1.81752600	0.33866700	2.39751000
C	0.13163700	1.09611100	2.84868300
H	1.17717300	1.02117100	2.52456300
H	-0.15173400	2.14784700	2.70979300
H	0.09671000	0.86235500	3.92490900
C	1.03304300	4.24055000	0.22201800
C	1.01171300	4.02577900	-1.33372300
B	2.13252200	2.30659600	-0.26568500
O	1.32777800	2.93262300	0.70715300
O	1.91280800	2.93441700	-1.51765100
H	3.46359500	2.47831600	0.02023300
C	4.72609300	2.09028000	-0.29501700
O	4.74840700	0.82782700	-0.39052500
H	4.78009200	2.70104900	-1.23271000
C	5.41223000	2.78149900	0.87939500
H	6.50245500	2.65678800	0.73884500

H	5.21168000	3.86626600	0.82955400
C	4.99060500	2.22589400	2.22774200
H	5.23081500	1.15135600	2.26021800
H	3.88959700	2.29685300	2.30275100
C	1.51472700	5.21325400	-2.13868900
H	0.89154000	6.10366600	-1.96239600
H	2.55605100	5.45481400	-1.89105400
H	1.47114500	4.97301400	-3.21107000
C	2.15470200	5.17238200	0.67550100
H	1.95563800	6.22006300	0.40755300
H	2.24714600	5.10286200	1.76928400
H	3.11850600	4.87690000	0.23721400
C	-0.28906500	4.69522400	0.81402700
H	-0.18787400	4.80458900	1.90421200
H	-0.59121100	5.66978900	0.40084600
H	-1.09191500	3.97418900	0.61454000
C	-0.35538900	3.60223500	-1.85396400
H	-1.09408600	4.41317100	-1.79239500
H	-0.25944100	3.30604700	-2.90728100
H	-0.75180600	2.74035000	-1.29893200
C	5.61920400	2.92433800	3.42238800
H	6.72077600	2.86277600	3.34987200
H	5.37867600	4.00318600	3.38880400
C	5.16848500	2.35070900	4.75896600
H	4.06706500	2.40970600	4.82127900
H	5.40842300	1.27269900	4.78650200
C	5.78793500	3.04474700	5.96108700
H	5.44109500	2.60732300	6.90991200
H	6.88737400	2.96988500	5.94268400
H	5.53415800	4.11716900	5.97712200

Cartesian coordinates of the optimized geometry of TS-**5b** at PBE0-D3BJ/def2-SVP level of theory:

Ga	-2.78428200	-0.14859300	0.51176700
Cl	-2.36128600	0.69616700	2.53041300
Si	1.31732300	0.84324700	-0.03195700
O	-0.31004000	0.81723900	-0.30917000
N	-4.35628500	0.85051000	-0.09277600
N	-3.76122100	-1.79475200	0.95419700
N	1.75756900	1.91561100	1.36598400
N	1.93547600	2.41672200	-0.71348700
C	-5.49512700	0.65820600	0.57742800
C	-5.75974400	-0.49232700	1.33386500
H	-6.72889900	-0.51881000	1.82981200
C	-5.01227500	-1.68372500	1.39675000
C	-6.54867200	1.72788000	0.54856900
H	-6.25962400	2.51205500	1.26777300
H	-7.52984400	1.33698200	0.84302500
H	-6.62270500	2.20971800	-0.43456600
C	-5.68706200	-2.87949600	2.00475200
H	-5.46841500	-3.79476800	1.43805300
H	-6.77201500	-2.73325200	2.05924300
H	-5.30698000	-3.04215600	3.02455500
C	-4.27549000	1.83931200	-1.12210500
C	-3.74864100	3.11720300	-0.85497900
C	-3.64261200	4.02700000	-1.91320600
H	-3.23009800	5.02030100	-1.72011900
C	-4.05568300	3.69509900	-3.19561800
H	-3.96705300	4.42088700	-4.00731100
C	-4.58000400	2.43058300	-3.44398800
H	-4.89729200	2.17269700	-4.45610300
C	-4.69236800	1.48120600	-2.42661800
C	-3.30223600	3.54063700	0.52870600
H	-3.49857000	2.70815600	1.21920300
C	-4.08877900	4.75286100	1.02806000
H	-3.88402900	5.64908500	0.42083300
H	-3.81095700	4.98669000	2.06745800

H	-5.17461500	4.57576400	0.99731800
C	-1.79771300	3.80610500	0.56392700
H	-1.22746600	2.91943100	0.25225900
H	-1.48236900	4.07221100	1.58400400
H	-1.52763900	4.64045800	-0.10459100
C	-5.24797400	0.10161700	-2.73256300
H	-4.85470900	-0.57643200	-1.95965200
C	-6.77440500	0.06228400	-2.63034200
H	-7.23120300	0.77090800	-3.33972100
H	-7.12921200	0.31761100	-1.62339200
H	-7.15027100	-0.94529300	-2.86787500
C	-4.80202800	-0.42872200	-4.09329800
H	-3.71131900	-0.36805500	-4.22029300
H	-5.26847700	0.12577600	-4.92214600
H	-5.10328800	-1.48105600	-4.20731800
C	-3.10475200	-3.06515200	0.95028800
C	-2.52643800	-3.58116700	2.12924500
C	-1.93625100	-4.84814600	2.07557600
H	-1.48888100	-5.26354900	2.98162500
C	-1.89565100	-5.58022800	0.89681500
H	-1.43098500	-6.56885000	0.88006900
C	-2.43089900	-5.04049000	-0.26722200
H	-2.37997500	-5.60996800	-1.19707800
C	-3.03714500	-3.78207600	-0.26490000
C	-2.47339100	-2.79828400	3.42636500
H	-3.06639200	-1.88105200	3.29765500
C	-3.04040100	-3.57577400	4.61367000
H	-2.42565600	-4.45756300	4.85336700
H	-4.06489900	-3.93274000	4.42896700
H	-3.06054300	-2.93831400	5.51078800
C	-1.03700300	-2.35847500	3.70987400
H	-0.38932500	-3.22581300	3.91678600
H	-1.00082800	-1.68717000	4.58096200
H	-0.61215700	-1.81562100	2.85579200
C	-3.64708100	-3.22411300	-1.53448400
H	-3.65059300	-2.12902200	-1.42629900
C	-5.10598200	-3.65669500	-1.68742700
H	-5.18582500	-4.75456700	-1.72926200
H	-5.53847200	-3.24974400	-2.61473200
H	-5.72460700	-3.30364000	-0.85002200
C	-2.83768900	-3.55825000	-2.78328000
H	-1.77642900	-3.29734200	-2.65820800
H	-3.22254700	-2.99841100	-3.64814600
H	-2.89514400	-4.62764900	-3.03893100
C	2.20763700	2.85334100	0.51637700
C	2.72891900	4.18519800	0.90393800
C	4.08742100	4.41579500	1.13788100
H	4.77557300	3.57793300	0.98843100
C	4.50440700	5.69150000	1.50794300
H	5.56588300	5.87772000	1.68742500
C	3.58204600	6.72980700	1.64160800
H	3.91967600	7.72816000	1.93024700
C	2.22909800	6.49504800	1.40197800
H	1.50287000	7.30493700	1.50149200
C	1.79894300	5.22227000	1.03303900
H	0.74104600	5.03089000	0.84115000
C	1.74350400	1.83071000	2.83231900
C	3.11388900	2.14571200	3.43254900
H	3.90150400	1.54583200	2.95588800
H	3.09976200	1.91172700	4.50744600
H	3.37470200	3.20626100	3.32314800
C	0.68472000	2.77626300	3.40273300
H	0.92669800	3.82450700	3.17565000
H	0.64091500	2.66953200	4.49719000
H	-0.30668700	2.53431500	2.99521400
C	1.35988200	0.38593400	3.14562400
H	2.08637600	-0.32770900	2.72784000
H	0.36289400	0.16480100	2.73599200
H	1.30656600	0.23430900	4.23326300

C	2.27789300	2.89235300	-2.05776400
C	3.27724700	4.04380200	-2.04782700
H	4.19315300	3.73966300	-1.52431400
H	2.87207200	4.95063500	-1.57930800
H	3.52991400	4.28804400	-3.09034200
C	2.92163700	1.71013900	-2.78563900
H	3.14330900	1.97808700	-3.82931400
H	2.27130300	0.82247100	-2.79488300
H	3.85541500	1.44560600	-2.26856500
C	0.97196700	3.32310100	-2.73101100
H	1.15262700	3.61153000	-3.77744000
H	0.53543800	4.18468800	-2.20301200
H	0.22928400	2.51256700	-2.71560700
C	-1.19972900	-0.22753600	-0.69302600
H	-0.70566200	-1.20694300	-0.55290100
C	-1.59007100	-0.07796500	-2.15687900
H	-1.97549100	0.94289000	-2.31045000
H	-2.44331200	-0.74780900	-2.34741300
C	-0.49388000	-0.37857700	-3.16401800
H	0.35789700	0.30523100	-3.05494600
H	-0.09745200	-1.39637200	-3.04335000
H	-0.86855700	-0.27017900	-4.19375500
C	2.55899000	-2.85714200	-1.38504300
C	2.40633600	-3.10378600	0.16051500
B	2.38553900	-0.85630000	-0.29244700
O	2.16480500	-1.48652200	-1.51728900
O	2.60375800	-1.79859400	0.71955300
H	3.69514700	-0.05317400	-0.26371500
C	4.65199700	0.58987900	0.33845600
O	4.72008300	1.80464100	-0.04331000
H	4.28810800	0.36744400	1.37562900
C	3.43180500	-4.06112300	0.74069100
H	3.31530500	-5.06448500	0.30365400
H	4.45675100	-3.71840900	0.55852400
H	3.28373400	-4.14414100	1.82747100
C	3.99357700	-2.97702900	-1.88483000
H	4.34405200	-4.01916500	-1.88281300
H	4.03741700	-2.59838900	-2.91608800
H	4.68140400	-2.37542500	-1.27727900
C	1.64119300	-3.70955400	-2.24416600
H	1.78296900	-3.45066000	-3.30348800
H	1.87282700	-4.77823900	-2.12031400
H	0.58488500	-3.55198600	-1.99100100
C	1.00809700	-3.55688800	0.56221900
H	0.79282000	-4.58320900	0.23601900
H	0.92516600	-3.52063200	1.65684200
H	0.22872100	-2.89995700	0.15014700
C	5.78915500	-0.34557300	0.00381400
C	6.61815300	-0.05500600	-1.08452700
C	6.03034700	-1.49171600	0.76979700
C	7.64642200	-0.91819100	-1.43816700
H	6.43209500	0.87178300	-1.63161100
C	7.06055700	-2.36231600	0.43570200
H	5.38982300	-1.69929100	1.63023900
C	7.84854100	-2.06671300	-0.67409700
H	8.30232300	-0.72674700	-2.28765800
H	7.27082300	-3.26515700	1.00937900
N	8.92588000	-2.98638600	-1.04266400
O	9.07636000	-3.97880400	-0.36146300
O	9.60121200	-2.70388200	-2.00928900

Cartesian coordinates of the optimized geometry of TS-**6a** at PBE0-D3BJ/def2-SVP level of theory:

Ga	2.18650500	0.53350800	-0.44869700
Cl	1.75683300	1.44373800	-2.44707900
Si	-1.97316500	0.01283000	-0.19274700
O	-0.48727300	0.57744700	0.41104400

N	3.24207800	1.94962300	0.42095700
N	3.74558700	-0.60403300	-0.86606700
N	-2.62109400	1.11816300	-1.55534400
N	-2.90143200	1.53482600	0.53590800
C	4.44257700	2.23046100	-0.08339400
C	5.16268400	1.35000000	-0.90744600
H	6.11424600	1.72554600	-1.28086500
C	4.88847900	0.00033900	-1.18823000
C	5.07106800	3.56341300	0.20952000
H	4.64688500	4.31103800	-0.48005000
H	6.15664200	3.53479400	0.05710800
H	4.85047100	3.90767300	1.22806800
C	5.95316300	-0.77775300	-1.90838800
H	6.06023400	-1.79315200	-1.50465600
H	6.91967900	-0.26210800	-1.86516800
H	5.66477200	-0.88756000	-2.96573800
C	2.64948000	2.78354100	1.41800500
C	1.86478000	3.89382700	1.04846100
C	1.27164000	4.65404500	2.06207700
H	0.65665300	5.51544500	1.79013800
C	1.44382700	4.33193900	3.40111200
H	0.96758300	4.93572000	4.17722700
C	2.22300300	3.23406500	3.75158800
H	2.35293300	2.98503800	4.80611100
C	2.83737700	2.44308400	2.77799100
C	1.63161800	4.27467000	-0.39938600
H	2.24975000	3.61860300	-1.02764300
C	2.03240300	5.72070500	-0.68840200
H	1.38952500	6.43756300	-0.15291500
H	1.93766400	5.93456800	-1.76433300
H	3.07179800	5.92657600	-0.39046100
C	0.17888600	4.01973700	-0.79838400
H	-0.12369700	2.98540400	-0.57817600
H	0.04293500	4.18784100	-1.87787000
H	-0.49308100	4.70039300	-0.25112800
C	3.70737100	1.26496100	3.17668600
H	3.62189500	0.52620100	2.36577300
C	5.18410800	1.65545700	3.27216600
H	5.32956300	2.45590200	4.01523200
H	5.58311300	2.00693700	2.31177100
H	5.79039400	0.78998100	3.58295700
C	3.26051600	0.59097300	4.46997100
H	2.19125900	0.33594000	4.45216800
H	3.44537200	1.22784500	5.34932900
H	3.82859000	-0.33849600	4.62526400
C	3.61827600	-2.02131900	-1.00770200
C	3.22698600	-2.59143800	-2.23666200
C	3.13379100	-3.98445100	-2.32202100
H	2.82840800	-4.43843000	-3.26775100
C	3.41219000	-4.79724600	-1.23218000
H	3.33115700	-5.88308600	-1.32103800
C	3.78028200	-4.22144700	-0.02144300
H	3.98861300	-4.86319600	0.83664000
C	3.88702000	-2.83543400	0.11636100
C	2.88039200	-1.75334900	-3.45037000
H	3.12662300	-0.70616800	-3.22393000
C	3.67064500	-2.16699800	-4.69125400
H	3.39686700	-3.17769200	-5.03321400
H	4.75620800	-2.16530800	-4.50850900
H	3.46454600	-1.47390400	-5.52134800
C	1.37750000	-1.80104600	-3.72186100
H	1.05194200	-2.82020500	-3.98642200
H	1.11140900	-1.12884300	-4.55111000
H	0.80112700	-1.47715400	-2.84484000
C	4.31917900	-2.23142900	1.43840700
H	3.91872400	-1.20667700	1.45911900
C	5.84087100	-2.11079500	1.53876100
H	6.31928000	-3.09907800	1.44669100
H	6.13045200	-1.68275800	2.51157100

H	6.25344800	-1.45973100	0.75584500
C	3.75793000	-2.97884600	2.64485500
H	2.67203000	-3.13289300	2.56043500
H	3.94899400	-2.40948000	3.56675800
H	4.23086500	-3.96508000	2.77235700
C	-3.20010500	1.94933300	-0.68160600
C	-4.06680300	3.10542000	-1.02755300
C	-5.44952200	2.92080500	-1.12821800
H	-5.87769800	1.93486600	-0.93581000
C	-6.27497400	3.98978500	-1.46784300
H	-7.35366700	3.83709600	-1.54648400
C	-5.72601700	5.24933200	-1.70436500
H	-6.37405700	6.08759800	-1.97038600
C	-4.34810100	5.43579000	-1.60218600
H	-3.91225600	6.41952800	-1.79050900
C	-3.51891500	4.36831000	-1.26787600
H	-2.43895800	4.50684200	-1.20851900
C	-2.81005300	0.95235300	-3.00228500
C	-2.81725100	2.29400200	-3.73702200
H	-3.72028900	2.88568500	-3.53876800
H	-2.77464300	2.10534500	-4.81998800
H	-1.93416000	2.88999300	-3.46141800
C	-1.62049800	0.13320500	-3.50046300
H	-0.67545800	0.65047200	-3.28137600
H	-1.69811700	-0.00987800	-4.58882900
H	-1.61016600	-0.85915000	-3.02932900
C	-4.11001200	0.18512600	-3.26303500
H	-4.98202000	0.74783600	-2.89616800
H	-4.07063100	-0.79557800	-2.76693300
H	-4.24559800	0.02033800	-4.34329800
C	-3.07627200	2.17311900	1.84226600
C	-4.55248900	2.45040600	2.13909600
H	-5.14919500	1.53068100	2.04656500
H	-4.97254300	3.20787800	1.46386000
H	-4.65921700	2.82336800	3.16895600
C	-2.52516800	1.19704700	2.88050500
H	-2.67848100	1.59486200	3.89397900
H	-1.45046800	1.04522200	2.71816400
H	-3.02695900	0.22208200	2.80720900
C	-2.26361300	3.46718600	1.92239800
H	-2.31840400	3.88567700	2.93913500
H	-2.64466700	4.22685500	1.22530700
H	-1.20961400	3.26412700	1.69002600
C	0.64213600	-0.27809600	0.49355900
H	0.42775500	-1.22888700	-0.03552400
C	0.94685400	-0.59708300	1.95201700
H	0.98310600	0.35976800	2.49950900
H	1.95993600	-1.02197200	2.01956700
C	-0.02667000	-1.55653000	2.61957100
H	-1.06089000	-1.18809700	2.57483300
H	-0.03090100	-2.53334800	2.11594600
H	0.23013600	-1.70410200	3.68099800
C	-1.55620700	-4.16682100	-0.51733200
C	-2.54832800	-3.89486100	-1.70258900
B	-2.16482200	-1.94705000	-0.52283600
O	-1.71385500	-3.00951200	0.29127100
O	-2.54771500	-2.47160500	-1.77616900
H	-5.22908600	-1.82669600	-0.29605000
C	-4.81328400	-1.14398500	0.46620500
O	-3.41636200	-1.07623400	0.28768400
H	-5.24405400	-0.14374400	0.27733400
C	-5.17513200	-1.61379800	1.86447000
H	-4.80807600	-0.86639600	2.58877000
H	-6.27539200	-1.62103900	1.96573900
C	-4.59393400	-2.97639500	2.21080400
H	-3.53846800	-2.99893400	1.89161000
H	-5.10853500	-3.75384200	1.61688900
C	-1.87998600	-5.39909600	0.31083600
H	-2.87254000	-5.32597000	0.77339300

H	-1.84118300	-6.31178600	-0.30384500
H	-1.14041900	-5.50155900	1.11898600
C	-0.10113000	-4.21823500	-0.97355700
H	0.14967800	-3.34604400	-1.59194200
H	0.55477100	-4.19782300	-0.09121300
H	0.12909100	-5.12749200	-1.54796100
C	-2.10128200	-4.44793100	-3.04522100
H	-1.99158800	-5.54275000	-3.00741000
H	-2.85167800	-4.20585700	-3.81255700
H	-1.14632500	-4.00681400	-3.35828900
C	-3.96910100	-4.36038400	-1.40107000
H	-4.29482800	-4.01004700	-0.41325700
H	-4.64742200	-3.93715000	-2.15675400
H	-4.06048300	-5.45639300	-1.42933800
C	-4.68486700	-3.33332600	3.68588800
H	-5.73989300	-3.31665800	4.01786400
H	-4.17320200	-2.55221600	4.27835800
C	-4.07067200	-4.68778600	4.01219800
H	-4.58208500	-5.46830600	3.42064300
H	-3.02183500	-4.69457700	3.66682200
C	-4.12962000	-5.04407200	5.48863700
H	-3.59280500	-4.30024600	6.09958600
H	-5.16983000	-5.07586900	5.85178200
H	-3.67743300	-6.02729900	5.69008800

Cartesian coordinates of the optimized geometry of TS-**6b** at PBE0-D3BJ/def2-SVP level of theory:

Ga	2.63769300	0.16887300	-0.15585400
Cl	2.69908100	1.29755800	-2.08655900
Si	-1.51409400	0.46631100	-0.61884600
O	-0.09469800	0.67029300	0.28074300
N	3.77667500	1.27531800	1.00321100
N	4.00082300	-1.22285100	-0.46438600
N	-1.72240700	1.77141700	-1.93607000
N	-2.30228700	2.05676000	0.11438700
C	5.07628300	1.34373200	0.71756300
C	5.74626000	0.39967300	-0.07763300
H	6.80109600	0.60057900	-0.25917500
C	5.27509500	-0.84099600	-0.54075500
C	5.88631900	2.49585300	1.24073400
H	5.74729200	3.35669100	0.56685000
H	6.95529100	2.25260300	1.26694400
H	5.55556400	2.81363200	2.23771900
C	6.28218400	-1.76189300	-1.16919600
H	6.11311700	-2.80843600	-0.88377800
H	7.30555200	-1.47018300	-0.90548100
H	6.17915600	-1.71219600	-2.26473000
C	3.19185400	2.13246900	1.98554200
C	2.70205500	3.40371400	1.62646600
C	2.10173100	4.18778000	2.61740800
H	1.71209400	5.17365500	2.35201700
C	1.98608800	3.73577200	3.92448700
H	1.50971500	4.36192700	4.68255100
C	2.47635500	2.47930400	4.26547200
H	2.38116100	2.12909900	5.29465300
C	3.08420100	1.65746400	3.31362500
C	2.79281900	3.93707800	0.21116300
H	3.36752900	3.21715700	-0.38775400
C	3.51057800	5.28483800	0.14828900
H	2.93880000	6.07597000	0.65924600
H	3.64225100	5.59842600	-0.89891700
H	4.50450700	5.24317200	0.61904800
C	1.40632300	4.02727800	-0.42429300
H	0.87869800	3.06344400	-0.37850400
H	1.48780200	4.31747500	-1.48313600
H	0.79579100	4.78143400	0.09819000
C	3.64270600	0.30259000	3.70837700

H	3.56587500	-0.33846900	2.81742600
C	5.12707100	0.38567800	4.07192600
H	5.28489500	1.07847100	4.91398300
H	5.74157100	0.72966500	3.22979000
H	5.50468200	-0.60452600	4.37201800
C	2.86521100	-0.36957200	4.83545400
H	1.78649700	-0.40550800	4.62645000
H	3.00944300	0.14648100	5.79762400
H	3.21915000	-1.40235100	4.97265700
C	3.63190000	-2.56835600	-0.77925700
C	3.35976900	-2.94963900	-2.10930800
C	3.01517200	-4.28192100	-2.36013300
H	2.79942000	-4.58977400	-3.38601400
C	2.93504700	-5.21468100	-1.33570800
H	2.66223400	-6.24965600	-1.55450600
C	3.19052100	-4.82191300	-0.02666300
H	3.11818000	-5.55622200	0.77771600
C	3.53850600	-3.50344900	0.27692800
C	3.40253400	-1.96977400	-3.26432600
H	3.79471500	-1.01436000	-2.88730500
C	4.31363400	-2.44225000	-4.39673600
H	3.92226200	-3.34846900	-4.88551100
H	5.32867200	-2.67470400	-4.04000400
H	4.39505000	-1.66271600	-5.16979400
C	1.99218100	-1.69120700	-3.78196300
H	1.53610500	-2.59940400	-4.20849000
H	2.01146100	-0.91685500	-4.56324300
H	1.33736600	-1.32677200	-2.97925500
C	3.84571300	-3.10526400	1.70738300
H	3.64946000	-2.02483500	1.77955300
C	5.32324500	-3.30686500	2.04886200
H	5.61393900	-4.36217300	1.92329200
H	5.51915200	-3.02305500	3.09502000
H	5.97893600	-2.69642100	1.41291800
C	2.95705300	-3.81300800	2.72626800
H	1.89194300	-3.73519000	2.46307000
H	3.09342700	-3.36782100	3.72310900
H	3.20650200	-4.88182000	2.81513600
C	-2.29458000	2.62424700	-1.07727700
C	-2.85828400	3.95514700	-1.41461100
C	-4.20283700	4.06150400	-1.78339400
H	-4.82652100	3.16579000	-1.81803100
C	-4.74460800	5.30582800	-2.09434800
H	-5.79560700	5.38287900	-2.38161700
C	-3.94874200	6.44928000	-2.03561300
H	-4.37495000	7.42527600	-2.27869200
C	-2.60805500	6.34418000	-1.66760600
H	-1.97968600	7.23657000	-1.62418100
C	-2.06104000	5.10120300	-1.35939200
H	-1.00841100	5.01286700	-1.08870400
C	-1.66772500	1.77144200	-3.40388600
C	-1.25547300	3.13782700	-3.95400900
H	-2.03200500	3.90250900	-3.82227300
H	-1.05977800	3.04436100	-5.03249000
H	-0.32997600	3.48348200	-3.46917300
C	-0.60468900	0.74687300	-3.79554500
H	0.36905300	1.01440900	-3.36104800
H	-0.50266600	0.71854100	-4.89073900
H	-0.89185500	-0.25814800	-3.45672500
C	-3.02527700	1.34202800	-3.96832800
H	-3.81461600	2.05825900	-3.69440600
H	-3.28652300	0.34267600	-3.58986100
H	-2.98293000	1.29358700	-5.06743000
C	-2.56619800	2.59720500	1.44967000
C	-3.87382300	3.38982100	1.50923500
H	-4.71718200	2.78843200	1.13629500
H	-3.82765800	4.32169900	0.93100300
H	-4.08742600	3.65530000	2.55533100
C	-2.67015200	1.39913800	2.39227000

H	-2.86386000	1.74310700	3.41835000
H	-1.72775900	0.83633000	2.39023700
H	-3.48177300	0.72072600	2.09406800
C	-1.38958200	3.47194800	1.88839200
H	-1.51741100	3.79408600	2.93316500
H	-1.31390500	4.37339400	1.26313300
H	-0.44983800	2.90878600	1.80789300
C	0.83486900	-0.39268100	0.45095600
H	0.54764400	-1.24333100	-0.19978900
C	0.81334300	-0.86415900	1.89948200
H	0.88963800	0.03181100	2.53825100
H	1.72034500	-1.45445500	2.09884300
C	-0.39888300	-1.69725800	2.28716800
H	-1.34282000	-1.17030300	2.08845100
H	-0.44217600	-2.62781900	1.70359300
H	-0.37783100	-1.95184300	3.35885500
C	-1.88157000	-3.67031700	-1.33506600
C	-2.68271300	-3.10470500	-2.55776500
B	-2.04126800	-1.38010300	-1.17407700
O	-1.89405300	-2.57322800	-0.42872900
O	-2.36796700	-1.71363400	-2.50463900
H	-4.98269100	-0.50443100	-1.43284000
C	-4.58488200	-0.18482600	-0.45035100
O	-3.20455000	-0.37859500	-0.42179500
H	-4.80378100	0.89476300	-0.35193200
C	-2.51485100	-4.87741900	-0.66488300
H	-3.51619900	-4.64782300	-0.27785500
H	-2.59363400	-5.72274500	-1.36580000
H	-1.89021200	-5.19668500	0.18260100
C	-0.42552100	-3.96899900	-1.67693500
H	0.04158000	-3.11616600	-2.18742600
H	0.13410500	-4.14545200	-0.74712000
H	-0.31660100	-4.85853200	-2.31468500
C	-2.24909300	-3.64716000	-3.90806800
H	-2.37730700	-4.73944600	-3.95666600
H	-2.86146000	-3.19571000	-4.70282500
H	-1.19858900	-3.40424700	-4.11384900
C	-4.19144200	-3.26503600	-2.39256900
H	-4.52163800	-2.93374000	-1.39800200
H	-4.69764900	-2.64652000	-3.14857500
H	-4.51290500	-4.30792200	-2.53057300
C	-5.29009100	-0.93995200	0.64522500
C	-6.59976500	-0.60092700	1.00802000
C	-4.64352400	-1.99283000	1.30350200
C	-7.26412000	-1.29876400	2.00812200
H	-7.10632300	0.22647300	0.50310600
C	-5.29775700	-2.69993500	2.30690200
H	-3.62142700	-2.25155900	1.01226300
C	-6.59872600	-2.34276000	2.64518100
H	-8.28144900	-1.05266300	2.31205700
H	-4.81796900	-3.52428600	2.83474900
N	-7.29037200	-3.08205600	3.70534000
O	-6.68896500	-3.98636300	4.24243400
O	-8.42171700	-2.74297300	3.97858500

Cartesian coordinates of the optimised geometry of compound **3** at PBE0-D4/def2-TZVPP,def2-QZVP(for all atoms E>Ne) level of theory (number of imaginary frequencies = 0).

Ga	9.76941565413571	11.78889093700755	5.04293487027929
N	9.24318713173379	12.97123543100091	6.49733140936048
N	9.53998440467291	13.12474047135688	3.62421629217731
C	8.93637043578163	10.00411750616958	4.75955167395018
Cl	11.99870230548103	11.74433933809330	5.23858652599682
Si	6.28677271977633	9.08957177419927	4.74714691280318
O	7.53456273644034	10.19094060922646	4.57750068186871
N	7.02972946481883	7.75144065209964	5.85764901089143
N	6.79259780396568	7.53562954876880	3.74935655843109
C	7.01491306457314	6.86087234370938	4.87009897952997
C	9.82694768332067	14.38171078910397	3.90736990973240
C	9.26167409751110	12.69321530146999	2.29197232855313
C	9.62150425449553	14.24473823808449	6.40944098195382
C	8.60355725154823	12.52008231491460	7.69083573859319
C	6.50050226576325	7.07046926273344	2.39566255917621
C	7.14076118302506	7.57372369126481	7.30303824062357
C	9.97858203727609	14.86552320490828	5.21511090404539
C	10.01127559028129	15.38335512448005	2.80726243696280
C	9.68270082232221	15.07295991218019	7.65766370038362
C	10.30807679369645	12.38771322181482	1.41170829834986
C	7.91739388795926	12.51482801206848	1.91890314972434
C	9.98489050753423	11.88338657061732	0.15530804456457
C	11.76540759133560	12.58330682336021	1.76210235958684
C	8.67271653621581	11.69005686778417	-0.22346478507703
C	7.65081283446261	12.00901381198769	0.65399768251763
C	6.78996458528372	12.87833501499113	2.85943225801367
C	12.51907864285582	11.25662131768720	1.77310401066779
C	12.43994651834239	13.55986914189527	0.80047216120945
C	5.47682226190165	12.19067105670869	2.52257322385284
C	6.57012101323562	14.38924312421011	2.92059875614623
C	9.34858693678906	11.92854007767176	8.71973440737671
C	7.21613358910522	12.69883017037370	7.81133206452789
C	8.68567423713669	11.54782994499693	9.88111824765177
C	10.83151017035297	11.68018642005145	8.59181972118184
C	7.32652422286464	11.74674215531748	10.02849099525995
C	6.60169557642949	12.31157385424406	8.99565096447725
C	6.39171289945550	13.32904563991843	6.71094041314276
C	11.10247857232556	10.19562710609752	8.35998461713902
C	11.62035447957156	12.18905775428596	9.79291570042963
C	5.13842342276681	12.52346700744934	6.39450870909733
C	6.02207010892872	14.77163639534165	7.05195293402647
C	7.14760650863017	5.39120625481978	5.00833865947378
C	8.40829423895066	4.80922769293542	4.93193109089452
C	6.03232560917380	4.58738408278454	5.21373228423190
C	6.17718899868255	3.21554442563832	5.34282300228269
C	7.43627327663781	2.63854103650186	5.26605797415909
C	8.55133700738199	3.43718536807017	5.06001261306563
C	6.41392901453650	8.32554067200942	1.53239837480557
C	5.15944863559541	6.33985280474253	2.33757665120325
C	7.61243044678689	6.17196948674275	1.85942099808526
C	6.00857398251413	6.71346141801665	7.86329291313022
C	7.03011528546873	8.96708649685626	7.90662953557428
C	8.49403049931533	6.97141528029667	7.67138506421844
C	9.58049057162675	9.25216024104575	3.59549202819995
C	10.83548859393149	8.50499720707268	4.01390867275432
H	10.28831736434387	15.89729905008605	5.29494228107872
H	11.08034541970700	15.49727250537327	2.61164136382369
H	9.62466308890675	16.35617157012957	3.10653786506895
H	9.53279752316814	15.07037032193913	1.88219077236124
H	9.80962400227967	16.12669044227024	7.42150788288441
H	10.53422476086404	14.74561520129452	8.26065395675190
H	8.79469005166355	14.94414543143842	8.27406665316757
H	10.78302685751995	11.63585623158234	-0.53468545878276
H	8.44106153501886	11.29043664157333	-1.20357886931510
H	6.62512546472617	11.85270845313793	0.3488101042733
H	11.82521505978898	12.99441444867317	2.76999324710381
H	12.49055483584572	13.14630539601840	-0.20946070364575

H	13.46272296967691	13.76353174404131	1.12500607128516
H	11.90558135580780	14.50886500390656	0.73802944736087
H	12.51966945910629	10.79680019660752	0.78153516061274
H	12.07738302338870	10.55380376384467	2.47751772861376
H	13.55681125780103	11.41521100179925	2.07304357906875
H	7.08390876984619	12.53260851383624	3.85259280228308
H	6.32494570560023	14.78344121613986	1.93106368516984
H	7.44357746682982	14.92333025956997	3.29026241518750
H	5.73715478585281	14.61982672401279	3.58883479403206
H	5.05113566231341	12.56154372484936	1.58633492032924
H	4.74710373954861	12.38354350919040	3.30931320608891
H	5.59592589712995	11.11064805618489	2.44798971614630
H	9.24932040361348	11.09139311172816	10.68613182331084
H	6.82888990014488	11.45265358856269	10.94503206468820
H	5.53396714772110	12.45413268237942	9.11014052568844
H	11.18996200842475	12.21360704161484	7.71139454026609
H	10.56689551527091	9.82724635124630	7.48373973265123
H	10.78521282948138	9.60219055039067	9.22175106474092
H	12.16676356783998	10.02232357313004	8.19146559389341
H	12.69072217523325	12.07020392048346	9.61283094743885
H	11.37885435065980	11.63678289505845	10.70398747958062
H	11.42418062437789	13.24587805369530	9.98492784275544
H	7.00836254242051	13.35072407467877	5.81187973582597
H	5.42439006345877	15.20976260876931	6.24948581620797
H	6.90302143226811	15.39942780166620	7.18915260231813
H	5.43284097321932	14.81463922870405	7.97164813377922
H	4.58830459927497	12.99695254874628	5.57855048044716
H	4.46186134236558	12.47343621411818	7.25054871436116
H	5.37775448777850	11.50459026929855	6.08620107884210
H	5.05094704546672	5.04040210232049	5.27965979722499
H	5.30398634386452	2.59590074566228	5.50591995056513
H	7.54845689008912	1.56605061047451	5.36708426121548
H	9.53650396757451	2.99167007780206	4.99845043076377
H	9.27589289215654	5.43685494114823	4.76741335162322
H	6.20722606950086	8.05506896123573	0.49589975365671
H	7.34361300973749	8.89449215346937	1.56461722302388
H	5.60646904002404	8.97222433170849	1.88203639502701
H	4.89806254689313	6.11458143866635	1.30120501348789
H	4.37220332797013	6.96417467948078	2.76405404045699
H	5.19471335748914	5.39740572547128	2.88397856809568
H	7.41840748931012	5.94150448650932	0.81005607620769
H	7.67211265327718	5.22983118301259	2.40252644020357
H	8.58008064713866	6.67268924103819	1.92330324807278
H	5.04189284205667	7.11769105978948	7.55653118168869
H	6.04865514322005	6.71570349281664	8.95459934284331
H	6.07887299722486	5.67905248444024	7.52947026823549
H	8.59325642984893	5.94896663342256	7.30702691734246
H	8.60695340168261	6.95332615024525	8.75720248355943
H	9.30757771675048	7.56929753490348	7.25706334459273
H	7.82962308256821	9.62126109096388	7.55793009469899
H	7.09363946991196	8.91336445682088	8.99303080036608
H	6.07513810753691	9.42695539874114	7.64448338461829
H	9.09134312431793	9.39540728809330	5.65934867410793
H	8.84643746373566	8.53199810500911	3.23285539361562
H	9.78947235399092	9.92150659116035	2.75582325955752
H	11.28804922558951	7.98768407037837	3.16517675932342
H	10.59496664534834	7.75474683583204	4.77325868029718
H	11.58715773073061	9.17208380043328	4.43921055652458

Cartesian coordinates of the optimised geometry of compound **4** at PBE0-D4/def2-TZVPP,def2-QZVP(for all atoms E>Ne) level of theory (number of imaginary frequencies = 0).

Ga	1.38815106956439	6.78337542541204	3.31141639512529
N	1.92293697979900	6.42147859288942	8.21209239808510
Cl	2.40069728446024	8.55459005664458	2.42737296039323
Si	1.42357872049907	4.82787558292908	7.33435853848450
O	1.48911859888836	5.26860529893083	5.70933329494348
N	3.23100487265948	4.75414789942011	7.92718335814318

N	1.48146754570540	5.46674562609134	1.87237370712233
N	-0.50764289532376	7.19164841292302	3.09108409531015
C	-1.37917102410161	8.96966099055605	4.54964591220019
C	3.07655102986517	5.88791633163300	8.59970933018568
C	-0.99409248850403	7.18096664979334	1.85457201828041
C	0.86633086577119	3.02957521085838	3.22780550240939
H	0.56743633943989	3.98419710063990	3.66514854115409
C	0.95984484926170	4.86011032981935	-0.43537905343222
H	0.05295250997913	4.77981009646007	-1.03076684764931
H	1.34413207519974	3.86493120882525	-0.21931511278153
H	1.71362571053092	5.37677231561107	-1.03358731046068
C	-3.04028951839798	8.40855399159754	6.21163450368505
H	-3.70958248713913	8.71660499128309	7.00610731202479
C	1.21430005470393	7.60664030123659	8.68744609007997
C	-2.97217808400131	7.07841774779826	5.83951602435683
H	-3.58990313920198	6.35077571166241	6.35052656972764
C	4.01502964439961	6.44930015577450	9.59913927284871
C	-1.34091569856645	7.62274388438936	4.16765509212415
C	5.96864963382498	7.78214401629269	10.03409440123361
H	6.78076833157075	8.39880726228153	9.66910391382175
C	-2.12503027396245	6.65895487410422	4.82236782496619
C	-0.37635337273577	6.53313884572360	0.78497730447263
H	-0.87524093343286	6.62403222933475	-0.16841896331412
C	4.11817945323785	3.62480976931574	8.21543987721141
C	2.48361048456340	4.45071918439827	1.88078913127049
C	-2.27354517881054	7.90543547435110	1.56177212315143
H	-2.67844102011012	7.60532146694773	0.59833119483633
H	-2.07240364590014	8.97951459552263	1.53351098730128
H	-3.02176913026620	7.74506430896037	2.33618529902801
C	3.18707000078235	2.27399715646782	2.60010284115918
H	2.99432923964847	1.34564823497846	3.12092219144547
C	-2.10175541506902	5.20643716511871	4.40480927002866
H	-1.16232409470663	5.03774735396864	3.87757114674243
C	3.61197472563023	6.32988348651703	5.07411087825191
C	3.72793477756294	4.67359734009504	1.27919982131259
C	2.20206947329346	3.25162703590301	2.55712368720598
C	-0.51122752891053	10.01920593203453	3.89785253825088
H	-0.02837294533225	9.57344286315442	3.02763381261496
C	0.70334760168176	5.64247108847068	0.81656406666156
C	4.27281873510916	5.16385346566608	4.69967995459893
H	3.69411679726761	4.28206051436683	4.46237768937101
C	4.41880646742484	2.47271064481794	2.00034977332930
H	5.17621811177211	1.69925634212839	2.04774994907225
C	-2.24202174305744	9.33895121842341	5.57610160976440
H	-2.28689429542626	10.37786404191654	5.88066542754738
C	3.88949657850494	6.19733265182262	10.95942280345509
H	3.07453104184494	5.58277136093957	11.32047324231287
C	3.87006433345470	2.59458287583601	7.11943652954695
H	4.13594198635258	2.99344517206347	6.14234335832006
H	2.81760687784372	2.30170541938180	7.10436312139768
H	4.46871997072771	1.70156894800875	7.30451389371789
C	5.84331243331386	7.52711144519269	11.39184821863575
H	6.55660008684855	7.94564144160494	12.09126305264562
C	-3.23666587462257	4.90281604139921	3.42746200290099
H	-3.15582359903697	5.49025598445745	2.51290739790452
H	-4.20635636389258	5.11819302233954	3.88355604301513
H	-3.22457192837384	3.84781133482432	3.14531199607142
C	4.54172248554469	5.72530488364750	-0.85637875235582
H	3.81168839921174	5.15026623165032	-1.42870558206659
H	5.48632633114525	5.17759182133828	-0.89121270106199
H	4.69848653773341	6.67941883502102	-1.36383790778252
C	4.37673973072970	7.46232624054602	5.34261372291085
H	3.87967891149709	8.38477804074980	5.62128420916210
C	5.05994187456771	7.24323936361512	9.13838474063423
H	5.15988249293551	7.42168520840224	8.07445134305881
C	-0.20577109742503	2.60785124533775	2.22511741903514
H	-0.38927766276545	3.36735418970611	1.46537749759023
H	-1.15142520156741	2.42266380701576	2.73955228236214
H	0.08540110106977	1.68416439138237	1.71857271475120

C	2.11789644591510	6.40198192228129	5.13655682670104
H	1.86503510090507	7.29343268751149	5.72155377019404
C	-0.09688196396281	7.65173273470910	7.91479651708304
H	0.06906502359587	7.72732223866874	6.84002328072644
H	-0.68596072504150	8.51609773919159	8.22049847691793
H	-0.68743313348231	6.75311593204713	8.10349434395537
C	5.58804839834830	4.03166645576972	8.19927635132979
H	6.20904355157680	3.13547481062541	8.26291715936510
H	5.84386837122755	4.67125658074267	9.04262578179854
H	5.83258861514193	4.55619431054348	7.27542714315201
C	4.09203024420386	5.96460395064649	0.58348282262941
H	3.21279074660723	6.60983092195085	0.56598170659789
C	3.75878347186052	2.99011134330414	9.55990867002757
H	2.69855372830116	2.73078054198535	9.58197405501208
H	3.97354550391591	3.66513327774996	10.38800212057186
H	4.34101934565025	2.07884949106332	9.71363625434499
C	4.68055949799926	3.66185304923651	1.35212173271793
H	5.65133608946173	3.82083519293717	0.89815870567543
C	-2.14924255527593	4.24670164949799	5.58454651312830
H	-2.05140571121709	3.21928535765987	5.22747966867160
H	-3.09945230574137	4.30701599475133	6.11990813631667
H	-1.33997806411681	4.43266476161213	6.29256561543782
C	0.92963724077273	2.03104067298602	4.37280866482488
H	1.09615822318231	1.01069606530336	4.01799775763405
H	-0.01372254208783	2.03746389702852	4.91959159667623
H	1.71948634569786	2.28716213714914	5.07802510792775
C	4.80402544735032	6.73289530007789	11.85262623471067
H	4.70253356233176	6.52956046854432	12.91159909734161
C	5.17940344941126	6.70889276913782	1.35365338970199
H	6.09629927336613	6.11639683419393	1.40370034917300
H	4.86688758603745	6.93545928843068	2.37115210209647
H	5.41025180088488	7.65369775320818	0.85728476385966
C	-1.31403082240103	11.22671873100608	3.42474075793248
H	-0.66659260248122	11.91604051297319	2.87904212962820
H	-1.74636869246277	11.77789953680290	4.26292583295049
H	-2.13332977677187	10.93603005330005	2.76441106626850
C	0.88861113277585	7.51123923997607	10.17823178564086
H	0.21021760797625	8.31928450951010	10.45984415528679
H	1.78158299578808	7.59697502161867	10.79556540903695
H	0.39767949710480	6.56122315753694	10.39758885113072
C	5.65339278248868	5.12436900103823	4.61474025681473
H	6.14149092438224	4.20400605662416	4.31675682322072
C	6.40679274643725	6.25592103499880	4.89188419832290
H	7.48718088403390	6.22672348510067	4.81995353914756
C	5.75949679686515	7.42976050279986	5.24837304521067
H	6.33298491360148	8.32615667323814	5.45463140947144
C	2.01410927940767	8.87544548986783	8.40782393084185
H	2.24839318707003	8.95913723358379	7.34593665184803
H	2.94700118555409	8.89474567035368	8.97063412112233
H	1.42864971254791	9.75140165636460	8.69385647473610
C	0.59824637836238	10.45913814999474	4.84883606032477
H	1.20573994933190	9.61119824486216	5.16648074614626
H	0.18290023116269	10.93331957299953	5.74213914440829
H	1.26220510962369	11.17168055418126	4.35624790434897

Cartesian coordinates of the optimised geometry of compound **5** at PBE0-D4/def2-TZVPP,def2-QZVP(for all atoms E>Ne) level of theory (number of imaginary frequencies = 0).

Br	14.73002363469404	12.44512589921813	3.77603440784643
C	15.86210505875314	13.90231919450611	4.19073806499639
C	17.22608408706195	13.70179886841940	4.29075026725938
C	15.28421872855577	15.14405822542244	4.39524457204570
C	18.05300543386581	14.76768994332594	4.63715939122569
H	17.65621182802158	12.72868827882304	4.10054532440014
H	14.21419845091972	15.27272708815443	4.30812359173129
C	16.11531729152074	16.21051724595350	4.70011078263986
C	19.53408506297357	14.60176722005255	4.76535929658009
C	17.48077301415979	16.02329933746140	4.82747471702637

H	15.68888468305144	17.19592033970272	4.84566331326197
Ga	20.40685056382878	14.95392434488655	2.99542948782391
O	19.95157054902673	13.36784483366250	5.31440949176289
H	19.88698500351158	15.42316682357689	5.39938849779219
H	18.11746291796187	16.86546842555430	5.06906834831892
Cl	19.63812174096820	16.86375294347124	2.14838930091036
N	22.34364400877571	15.15855706230969	2.88663094948666
N	20.25123420369140	13.67662748718078	1.53638559898296
Si	19.91465056838162	12.86662639213474	6.92601874741915
C	22.89643518578561	15.10238339684496	1.67910469400530
C	23.15840204847143	15.48563971811133	4.01295996906735
C	21.10595339629851	13.76544641203355	0.52925740398442
C	19.15981531470455	12.75668425478541	1.47176236129284
N	18.08326020548721	12.99604948263015	7.45978455558664
N	19.56849367969856	14.48724556482530	7.83961242158900
C	18.34153642255534	14.09294359935824	8.16193550899630
C	22.27528729873415	14.53386021227561	0.56733659081230
C	24.26332630185993	15.68133470458221	1.47080915932529
C	23.31448445132291	16.81683458000491	4.42078045552168
C	23.80712282754061	14.43797632273617	4.68749756161441
C	20.84033958720424	13.00314054414030	-0.73244431165333
C	17.98524680620044	13.09938022448277	0.79045958693044
C	19.29007051479502	11.52440106891170	2.13332815504951
C	17.06584133783673	11.96946170995996	7.70480235202096
C	20.38034065077962	15.58996601640525	8.34858345194429
C	17.42478524471258	14.75531634783497	9.11908397561299
H	22.83700301667879	14.57370277582159	-0.35382249686427
H	24.18136893837293	16.77068290364980	1.43039430154708
H	24.69514236967374	15.33371589761915	0.53538352952215
H	24.93718186417815	15.44507904650719	2.29231086984818
C	24.14776739568379	17.08076821703300	5.50251828768821
C	22.61424449330594	17.96345114633235	3.73136106361923
C	24.63304595064107	14.75461854867616	5.75851437343185
C	23.67166363473299	13.00045635538564	4.23925588408622
H	20.44965108866299	12.00870235269288	-0.52114340584857
H	21.74489463195592	12.91967768479402	-1.33033114153267
H	20.08386324639862	13.52507199807160	-1.32162599649618
C	16.95783150833779	12.16335032784391	0.74583839467137
C	17.77809297703628	14.44435792441757	0.13302186967419
C	18.23387619490753	10.62563855030735	2.05773981709060
C	20.54491796738226	11.17511093637108	2.90185301668192
C	17.33698161389889	11.25890690243056	9.03182415767071
C	15.65233863140566	12.54416462332839	7.69152988265451
C	17.20262699768176	10.94861308676487	6.58118049960231
C	19.76056848856791	16.93873246394521	7.99249546446916
C	20.58619890472616	15.49671261117362	9.86050649330581
C	21.73915916438790	15.45671113920060	7.67534912268137
C	17.44358469819523	14.47928303243600	10.48044032758350
C	16.50648558914648	15.67035415863603	8.61379948128348
H	24.28188007234230	18.10580042745114	5.82718652398682
C	24.81049040956745	16.06497174058731	6.16303344692793
H	22.12959783603788	17.57834929999523	2.83391343738916
C	23.58228062133125	19.06380687976976	3.30682919999492
C	21.51486694336072	18.53379564713261	4.62231075073905
H	25.14729703709346	13.96050466519737	6.28529226187945
H	22.77837514475902	12.93526394752270	3.61767290603318
C	24.86371977824227	12.58408382944809	3.37807452154577
C	23.49636072986603	12.03136943650122	5.40016600007863
H	16.03884306808223	12.41224113158138	0.22925709236931
C	17.07926151468568	10.93493047917281	1.36139680261199
H	18.71580517763192	14.99929371200795	0.18181865755553
C	17.37169254729124	14.31323565134454	-1.33279549612939
C	16.73380255858579	15.25634900871316	0.89495799184350
H	18.30978712031164	9.67058635503820	2.55988732178294
H	20.87370840356078	12.08671999854494	3.40545726519119
C	21.66745115260279	10.70860704303762	1.97698294253962
C	20.30981114637968	10.13924162771645	3.99062268895354
H	17.21350115685177	11.93589733645274	9.87704909302611
H	18.35328234036523	10.86033455621820	9.04642802015177

H	16.63831905413775	10.42987190499265	9.16404167136750
H	14.93027092838108	11.72575378874447	7.67312839082513
H	15.49461245355325	13.15677731582061	6.80424353341261
H	15.44805702041159	13.14958911033640	8.57331301803184
H	18.22591398221201	10.56838845486894	6.53476663778987
H	16.94194632521735	11.38259543995132	5.61796752244262
H	16.53499671105936	10.10510116218544	6.76207363954072
H	18.79971258024043	17.08157237353343	8.48682022024236
H	19.60906936327917	17.02537727251426	6.91625804804514
H	20.42579913527985	17.74608569031736	8.30471660280226
H	21.32525459468129	16.23587325556615	10.17652033775948
H	20.95795689764444	14.50641060696102	10.13033717074105
H	19.66638852361806	15.69023521469872	10.41002630042396
H	22.39762729599648	16.26591601447802	7.98936735813729
H	21.65883570383176	15.49662986163524	6.58890097710547
H	22.21013929566350	14.50891047661620	7.94304497488099
H	18.16234259486974	13.77345911829645	10.87705723339734
C	16.54658203812219	15.10816646360467	11.32922038290188
C	15.61495611587216	16.30108175098247	9.46532151242181
H	16.48910111037146	15.87121592448073	7.54925344058927
H	25.46068020371305	16.29222323396168	6.99948457843755
H	24.02097445523103	19.57172531275952	4.16869045259433
H	23.05702134582441	19.81646454517231	2.71538422022419
H	24.40389700200430	18.67143731028925	2.70452045518503
H	20.78182555699441	17.76884344692600	4.87915270440010
H	20.98240857179447	19.33571331993200	4.10752513493500
H	21.92991942361629	18.93418957021543	5.55105156993179
H	25.79627997955993	12.67834021450378	3.94037576332484
H	24.95132957693577	13.19209877916649	2.47751219648870
H	24.76283959369741	11.54222973035204	3.06659188849230
H	22.64234215416458	12.29369161454534	6.02680278825046
H	24.38605005992427	11.98605298229575	6.03240957999040
H	23.32851419380429	11.02301195098455	5.01599928286408
H	16.26407255075141	10.22312092501033	1.32006005269430
H	16.37686215066761	13.87342123024323	-1.43203145720486
H	18.06158469480290	13.68625363554453	-1.90065040171690
H	17.34203109200199	15.29829886199991	-1.80295134781690
H	17.03216004509312	15.42040694896102	1.92884784192207
H	15.76782265242339	14.74497130469053	0.89496804212786
H	16.60297525501976	16.23487421407584	0.42827831617122
H	22.54834406601205	10.43524694487145	2.56208079433234
H	21.97028109641907	11.48014407852747	1.27011612602884
H	21.35667376383343	9.82829697538006	1.40856154627645
H	21.19545418922177	10.05869802159830	4.62162919295989
H	20.11276825906810	9.14790087156985	3.57447874463080
H	19.47390955807021	10.41627202366768	4.63220516769788
H	16.56406596478148	14.88577987713170	12.38905634989500
C	15.63154320425068	16.01973127494556	10.82357740798039
H	14.90078225925642	17.00991938741002	9.06466178396817
H	14.93089312799259	16.51007064206678	11.48823898402219

Cartesian coordinates of the optimised geometry of compound **6** at PBE0-D4/def2-TZVPP,def2-QZVP(for all atoms E>Ne) level of theory (number of imaginary frequencies = 0).

Ga	15.30298300046337	17.29949664441369	1.22166589604816
Cl	17.17902180919890	16.48442911987236	2.09784067617836
N	15.60306838682170	17.14872873972297	-0.70246573481189
N	14.05005592133958	15.81152470516928	1.35053541058446
C	14.88759370897697	19.08248265912095	2.02613119510987
C	15.59963955093402	15.92995503829739	-1.22888628589839
C	15.96116703679913	18.26471301622846	-1.51904049399620
C	14.20512697627471	14.78785237339271	0.52440002458219
C	13.09003645823723	15.74028298677399	2.40492774902414
O	13.75006753820679	19.66747479987309	1.41503884247428
H	15.77393407038492	19.68584297613579	1.80068098754900
C	14.80361577710392	18.96694160972072	3.51555604923915
C	15.02381689463817	14.81931041438468	-0.60949877724935
C	16.24692412627697	15.70345355787826	-2.56195003608404

C	17.29729024514639	18.66797336809226	-1.63502867179029
C	14.93596922121430	18.93665206501019	-2.20573061051924
C	13.46458053802591	13.51203759472881	0.78655047190350
C	13.40229512661490	15.09275841813659	3.60595413246172
C	11.84767310231416	16.36809086110647	2.21516999166647
Si	13.29627053993987	21.29032601571879	1.38472359847424
C	13.60975483616201	18.66115974905421	4.15777251943524
C	15.93726530104943	19.14381872808815	4.30367644618225
H	15.10992991608774	13.88738909535934	-1.14762154621962
H	16.01874065588816	16.50084087720503	-3.26715567269225
H	17.33196716910480	15.69629709248567	-2.43085537291096
H	15.94557455532787	14.74785593802200	-2.98444438415855
C	17.59158716140024	19.73780652482754	-2.47395199385803
C	18.41649207795219	17.99900290015076	-0.87352807671110
C	15.28186248184100	19.99972337854009	-3.02945963778956
C	13.49494098555928	18.49059326115922	-2.10533865732095
H	13.47550359645223	12.87224939310576	-0.09254884564524
H	13.93692241740767	12.97555414513448	1.61155752960971
H	12.43390660333260	13.70480081060185	1.08250869259698
C	12.43045333702677	15.05242620145565	4.59954985535219
C	14.75518248896385	14.48197732459302	3.88619769978362
C	10.91224916717736	16.29964258768404	3.23928754287800
C	11.53877948765718	17.11231123904926	0.93562682278231
N	14.88420929696886	22.15481496609906	1.94245801054560
N	13.19408484733311	21.84254288297037	3.21622240576317
C	14.34180812337057	22.50104016996087	3.10421055502587
H	12.72143542357280	18.49998685575456	3.56380106298278
C	13.54071722351146	18.57451032977666	5.53649259288074
C	15.86910093134240	19.03973695697515	5.68355714413102
H	16.88708312517390	19.36595795927234	3.83034407217882
H	18.62116122283357	20.05947466538652	-2.57750055318806
C	16.59932730898646	20.39605152546415	-3.17242327694670
H	18.01674550021318	17.10910468585494	-0.38679155673808
C	19.56146861329552	17.56492755570828	-1.78351004653265
C	18.93328300047381	18.91879271465447	0.22951010380151
H	14.50609551809922	20.52812203115601	-3.56859301098493
H	13.38496663571400	17.96783744226630	-1.15455326760370
C	13.15142195165793	17.50211305432136	-3.21964857280127
C	12.50718378860619	19.64833079034647	-2.10999827024119
H	12.65693981197023	14.55304217978624	5.53301600972992
C	11.19525363293143	15.64096208412459	4.42353117712891
H	15.34696390548826	14.51496967549892	2.97056545269373
C	14.65305873396786	13.02874128139028	4.34265814235222
C	15.49830262413597	15.30565248093926	4.93468294427905
H	9.94999878912953	16.77839257697235	3.11452837213101
H	12.46116664996546	17.60995060536092	0.62832921922276
C	11.11248650589937	16.16568101274818	-0.18413291818921
C	10.49887085803544	18.20783258892351	1.11369119271000
C	16.10493038052017	22.61407871082656	1.28495734995297
C	12.05053756942429	22.14387997669971	4.08180929325165
C	14.90927808746388	23.44066817656063	4.09984666272024
H	12.58563304012194	18.36185228997161	6.00423754639542
C	14.66739304796728	18.76813854451429	6.33122693757184
H	16.76834997653662	19.18755229356640	6.27335237298952
H	16.84928079443936	21.22648871677741	-3.82197064286371
H	20.07638158072645	18.42246224444321	-2.22267187016010
H	20.29902455961377	16.99771703022278	-1.21206423324154
H	19.21235018417246	16.93670069418473	-2.60518673064617
H	19.35256288469297	19.83635537469598	-0.19171124540375
H	18.13561066727358	19.19532121104848	0.91924298540132
H	19.71006388918797	18.41901393046714	0.81109214472840
H	13.32167327941534	17.95483065378995	-4.19988852762839
H	13.74658013848231	16.59107158559828	-3.16058825863849
H	12.09982095100121	17.21367093576462	-3.16075601112838
H	12.48526127207308	20.16267467938488	-3.07372259471671
H	11.49835550752778	19.27002226292360	-1.93116350312072
H	12.73138056117800	20.37862898293979	-1.33072083047198
H	10.45386618990677	15.59806387126039	5.21281753034225
H	15.65097456692597	12.59621027588201	4.43939601375588

H	14.16819080172975	12.95134752556181	5.31876891513000
H	14.08180393381973	12.41200062675313	3.64619228781397
H	15.58980746705271	16.34878618689152	4.63738110606124
H	14.97307481154203	15.27045444757386	5.89157586759723
H	16.50527389190836	14.91011089196610	5.08212560835479
H	11.90054312456861	15.46441884333807	-0.45652857647301
H	10.23147616891548	15.59133141377614	0.11356865294166
H	10.85409902966785	16.73415782872842	-1.08009214419281
H	10.45351635117543	18.82148301519653	0.21310543618856
H	9.49885943752231	17.80035331442770	1.28306674973770
H	10.74883992589921	18.86486228595551	1.94601439687133
C	16.15341032193943	21.90297927081869	-0.06042428049111
C	16.07630651995393	24.12062964743971	1.02524580471477
C	17.34128598864779	22.25161951430072	2.10311414729142
C	11.00226384269935	21.07396941217269	3.79943559973607
C	12.41758145464732	22.10285225866751	5.56259753976714
C	11.45314881821739	23.50560190425452	3.72332273215798
C	14.71080781735011	24.81355369360205	4.02460779633551
C	15.64900036036048	22.90757771576544	5.15098280541874
C	14.61188763472761	18.67496286636995	7.82869198720498
H	17.04613709387209	22.19927140475991	-0.61069275364043
H	16.17666629582896	20.81921955984948	0.05276805459943
H	15.28050417870053	22.15872151395340	-0.66392464697900
H	15.15508185326619	24.39748132356853	0.50899165000666
H	16.14958872905817	24.69321247507340	1.94839706029386
H	16.92027575181269	24.40083811527905	0.39145642099904
H	18.24310171592557	22.52068023347895	1.54970819265939
H	17.35935715480282	22.77936208366353	3.05647137060480
H	17.37465042857121	21.18004350711512	2.30261144798925
H	10.74984442688392	21.05850090644695	2.73656737903053
H	11.36419001941543	20.08893787264838	4.08870618142940
H	10.09199894129520	21.28653251421887	4.36206672517052
H	13.04506992866132	22.94392666614421	5.85318571091472
H	11.50385519002202	22.14880407289640	6.15921145193896
H	12.94031859107919	21.17678392412053	5.80239101681126
H	11.21025759266770	23.54329319350228	2.65958631301383
H	10.53739824508805	23.67733785902376	4.29351387537319
H	12.14521765580055	24.31511378845893	3.95473933887337
H	14.13842580272745	25.23111147428524	3.20593674320498
C	15.24413443474840	25.64724943461276	4.99497641333028
C	16.18577261235759	23.74409951856889	6.11535116681014
H	15.78845626567527	21.83471306727149	5.21144644108269
H	15.41460595332309	19.28917983228830	8.24557814146403
H	13.67216342092338	19.10540578271188	8.19088272274035
C	14.74657945256657	17.25074390458843	8.37399266299821
H	15.08213063700146	26.71633154169524	4.93247385082623
C	15.98284955822371	25.11442937951647	6.04075504562077
H	16.75981529479801	23.32236957946330	6.93128536343673
H	14.97048845576802	17.30376750255663	9.44497456808831
H	15.60938464633260	16.76816873037079	7.90237143725213
C	13.50789994316702	16.39441526055694	8.17536621225136
H	16.39937965828651	25.76746659367833	6.79786740825609
H	12.66264677004504	16.86550866195331	8.69241827899967
H	13.23974961380024	16.37206001902289	7.11482423171039
C	13.67676022019218	14.97159710014965	8.68015860925476
H	14.46385203349136	14.47284317571085	8.10160528743436
H	14.03557760788929	14.98959977050517	9.71637288596124
C	12.40112278087164	14.14790514331438	8.61097502573573
H	12.00430519043860	14.17491939547526	7.59050872002448
H	11.63657479582579	14.61860817408396	9.23852167002101
C	12.60062934998006	12.70493681642224	9.03979522416015
H	13.33261831082390	12.20365519756216	8.40129187257991
H	12.96920404269765	12.64734377796775	10.06709207731468
H	11.67056623431087	12.13569976436843	8.98834200850390

Cartesian coordinates of the optimised geometry of compound **C** at PBE0-D4/def2-TZVPP,def2-QZVP(for all atoms E>Ne) level of theory (number of imaginary frequencies = 0).

Ga	2.70380786333892	3.04625409314722	12.58384147400649
Cl	1.04504353021230	1.96694509573806	11.55456043155751
N	4.26586395813591	2.51396157887698	11.53091463871264
N	2.58139680562247	4.84344016119334	11.80609033736780
C	2.64997242332541	2.90468311857926	14.56067977262813
C	4.31749531521417	2.94268908246599	10.27574240008818
C	5.20712875964200	1.54119582398758	11.98999940367274
C	2.81785253525166	4.94943775956877	10.50687579498678
C	2.07335391553562	5.94343443277169	12.56008786590384
O	2.56723799148839	1.52659128274874	14.89748569095343
H	1.72672767434517	3.41779625691407	14.86842047054409
C	3.82770553355205	3.56228129432742	15.25997448448240
C	3.55374036300940	4.00637815360038	9.78301367274421
C	5.23568736944920	2.26098883363919	9.30705272169660
C	4.96965121338568	0.17512366732333	11.79317975319415
C	6.36314724442908	1.98660828997806	12.65315030876075
C	2.25574639835934	6.11028703483772	9.74436527353445
C	0.69576398688602	6.10357172096860	12.75184695075586
C	2.99372845911468	6.82900745692596	13.14903882282639
Si	1.58928036719177	0.95876064017594	16.14699752195573
H	4.70169766198732	2.92122033894929	15.10720818894593
H	4.05665709463606	4.51909855369771	14.77660671645882
C	3.63519365357553	3.82257062393779	16.75444343092169
H	3.64083297788885	4.19377374789126	8.72310934250213
H	4.83377617875053	1.27647680719955	9.05794284176302
H	5.33340006935086	2.83916076301195	8.39145779063201
H	6.22222106766656	2.09687932173915	9.73939554556877
C	5.92548623040121	-0.73188166059818	12.23901053549134
C	3.70362817115741	-0.34718424600490	11.15896512377189
C	7.28741541265991	1.04112231339026	13.07856938275210
C	6.61215595079231	3.45833796059157	12.89500945715157
H	2.22585924178182	7.01947734559994	10.34152714466247
H	2.81537639194188	6.29147068668673	8.82913826918611
H	1.22393227213237	5.87067544619892	9.47137263520373
C	0.25677097584761	7.15615870307696	13.54874246991224
C	-0.32206958159378	5.17992394734834	12.12795808394516
C	2.50510365068684	7.86789827699254	13.92965057913335
C	4.48189498325140	6.68084617892417	12.92491556449687
N	0.64077909140831	-0.46990504145791	15.30979173411587
N	2.45555633198350	-0.68428827989464	16.41292937490010
C	1.45247327433759	-1.36158229728394	15.86741946324901
H	3.22962083702861	2.91069046808710	17.20958826747600
C	4.96880532232672	4.12911776945414	17.41736050893480
C	2.63327250385548	4.94012448316096	16.99996490322022
H	5.75421316499049	-1.79184852626218	12.09474416776885
C	7.07927750374725	-0.30990119746603	12.86719781764748
H	3.14972842666859	0.49558344837016	10.74517064764110
C	3.97699721283669	-1.33693776222445	10.03094860089908
C	2.81442047572863	-0.98494738488854	12.22333255341460
H	8.18447256502093	1.36563084189568	13.58923099890707
H	5.63496201584941	3.92109963087773	13.04377855236724
C	7.25973463575459	4.13710356582969	11.68922059922585
C	7.44120302764200	3.72708162615061	14.14398712389244
H	-0.80679011004387	7.28704825079425	13.70969094164986
C	1.14687755935990	8.03331616450021	14.13416978915893
H	0.20527275970229	4.46981154678646	11.49141423359064
C	-1.31460448604746	5.94129492497982	11.25396336253430
C	-1.05375146514522	4.37190911382463	13.19513680449122
H	3.19754737555061	8.55751293105385	14.39513745488727
H	4.66983054252616	5.62417960030094	12.72584045181574
C	4.94400416391136	7.46032396625721	11.69436107402688
C	5.31397217608797	7.08953806405809	14.13320720081505
C	-0.75643579190887	-0.60060145274445	14.89522448826121
C	3.77610316619823	-1.10627792192441	16.86246030273458
C	1.24430655379517	-2.82608302646283	15.95369508368475
H	4.84981758956436	4.30104564223050	18.48964974611217
H	5.67597225306863	3.30605228263168	17.28679108984723
H	5.42378567695080	5.02756555966304	16.98816518383475
H	2.49310437242609	5.11137790005569	18.06959895742113

H	2.97293266797320	5.87799817558016	16.54964252488791
H	1.65473579040866	4.70463010146209	16.57813650586601
H	7.81145093181437	-1.03271016372796	13.20664823543154
H	4.44252736253803	-2.25245264513007	10.40325128606024
H	3.03955412117470	-1.61995941165967	9.54788808522340
H	4.63842289564399	-0.92115286729759	9.26796950986078
H	2.59639452510392	-0.29186977905864	13.03581964917855
H	1.86622508479221	-1.29846875276622	11.78243715269686
H	3.30659387866220	-1.86509697771242	12.64755868914666
H	8.20486017083877	3.64967724043641	11.43732567907934
H	6.61805883989881	4.11352126592345	10.80930930025252
H	7.47244492225569	5.18503993698696	11.91304249634332
H	7.04882386471174	3.20020266591176	15.01448054069448
H	8.48410626453408	3.43071898403034	14.00948889811896
H	7.43822833045315	4.79449620386558	14.36938347017236
H	0.78566615182735	8.84514724198363	14.75383207484473
H	-1.94058404327558	6.61282891768989	11.84630258278797
H	-0.80772542378668	6.54588815263790	10.49928632507848
H	-1.97684341444773	5.24058533106727	10.74104760309278
H	-1.61804724836316	5.02520568396077	13.86549989491365
H	-1.75103164691274	3.67241784818089	12.73060945634444
H	-0.35791019287293	3.78926681572729	13.79890612287495
H	4.70045241122801	8.52064532958739	11.79822850725055
H	6.02596439118425	7.37230134693093	11.57223449023337
H	4.47868159219279	7.09461026021404	10.78045950384711
H	4.97719085593153	6.59720395569599	15.04629172179589
H	6.36043374783916	6.82567048280069	13.97010701800469
H	5.28139604793498	8.16815998975609	14.30103798757642
C	-1.65101027243975	-1.01355079959681	16.06328320148591
C	-0.89561948696082	-1.58882638098032	13.74122809292195
C	-1.17380046161044	0.78283165395035	14.41008468032951
C	3.72472141927822	-2.24086027991891	17.88244738502390
C	4.41596067343122	0.11481947722450	17.51342677677012
C	4.61153775536607	-1.51807054899047	15.65130492016573
C	0.69508276064927	-3.39432057244294	17.09768473728782
C	1.62064186388488	-3.64286637799178	14.89445734941370
H	-1.51346592133278	-0.32891358965775	16.90255282047138
H	-2.70054266324961	-0.98080052766303	15.76242542810098
H	-1.43216116274912	-2.02775402243230	16.39793578610897
H	-0.66690828755547	-2.60897807875674	14.04804852479972
H	-1.92220012450371	-1.57298875785496	13.36956407093446
H	-0.23120255620725	-1.30865876838886	12.92323214448741
H	-1.09884995300020	1.51206080732669	15.22070270958696
H	-0.53965829309109	1.11115615046737	13.58599839640110
H	-2.20888217119945	0.76324721022671	14.06457206462860
H	3.06856307825291	-1.98218596705951	18.71566104961178
H	3.37570625642217	-3.17396091199290	17.44273208779472
H	4.72709610632894	-2.41433169253324	18.27949559517819
H	5.41357813593263	-0.13539508408347	17.87729720748272
H	4.50595880042551	0.92789833251396	16.79200435558462
H	3.81661910719688	0.46507031593838	18.35616330696067
H	5.63439256028147	-1.75323152632125	15.95441988642062
H	4.18997242679383	-2.40313177806760	15.17114546448303
H	4.64289672247685	-0.70922501022217	14.92165052857536
H	0.40984698947706	-2.75717169879885	17.92575527064427
C	0.52221944457766	-4.76572049432351	17.17925991872307
C	1.45537001148550	-5.01624461753121	14.98155799985842
H	2.03699150410331	-3.19624576375370	14.00046730481541
H	0.09120561700997	-5.20117132250130	18.07240917604218
C	0.90550951089342	-5.57973561019365	16.12277558891779
H	1.75378896441602	-5.64681913736720	14.15302868519482
H	0.77470593035369	-6.65283573805528	16.18958980173651

V. References

(1) Schoening, J.; Gelhaar, A.; Wölper, C.; Schulz, S. Selective [2+1+1] Fragmentation of P_4 by heteroleptic Metallasilylenes. *Chem. Eur. J.* **2022**, *28*, e202201031.

(2) Kapp, L.; Wölper, C.; Siera, H.; Haberhauer, G.; Schulz, S. Catalytic hydroboration of aldehydes and ketones with an electron-rich acyclic metallasilylene. *Chem. Sci.* **2024**, *15*, 4161–4170.

(3) Leong, B.-X.; Lee, J.; Li, Y.; Yang, M.-C.; Siu, C.-K.; Su, M.-D.; So, C.-W. A Versatile NHC-Parent Silyliumylidene Cation for Catalytic Chemo- and Regioselective Hydroboration. *J. Am. Chem. Soc.* **2019**, *141*, 17629–17636.

(4) Rao, B.; Chong, C. C.; Kinjo, R. Metal-Free Regio- and Chemoselective Hydroboration of Pyridines Catalyzed by 1,3,2-Diazaphosphenium Triflate. *J. Am. Chem. Soc.* **2018,** *140*, 652–656.

(5) Mukhejee, D.; Shirase, S.; Spaniol, T. P.; Mashima, K.; Okuda, J. Magnesium hydridotriphenylborate $[Mg(thf)_6][HBPh_3]_2$: a versatile hydroboration catalyst. *Chem. Commun.* **2016**, 52, 13155–13158.

(6) Lawson, J. R.; Wilkins, L. C.; Melen, R. L. Tris(2,4,6-trifluorophenyl)borane: An Efficient Hydroboration Catalyst. *Chem. Eur. J.* **2017**, *23*, 10997–11000.

(7) Sheldrick, G. M. SHELXTL, and Ray Instruments. *Acta Cryst.* **1990**, *46*, 467–473.

(8) Sheldrick, G. M. Crystal structure refinement with SHELXL. *Acta Cryst.* **2015**, *71*, 3–8.

(9) Hübschle, C. B.; Sheldrick, G. M.; Dittrich, B. ShelXle: a Qt graphical user interface for SHELXL. *J. Appl. Cryst.* **2011**, *44*, 1281–1284.

(10) F. Neese, F. Wennmohs, U. Becker, C. Riplinger, *J. Chem. Phys.* **2020**, *152*, 224108.

(11) C. Adamo, V. Barone, *J. Chem. Phys.* **1999**, *110*, 6158–6170.

(12) E. Caldeweyher, J.-M. Mewes, S. Ehlert, S. Grimme, *Phys. Chem. Chem. Phys.* **2020**, *22*, 8499–8512.

(13) F. Weigend, R. Ahlrichs, *Phys. Chem. Chem. Phys.* **2005**, *7*, 3297–3305.

(14) F. Weigend, *Phys. Chem. Chem. Phys.* **2006**, *8*, 1057–1065.

(15) M. J. Frisch, G. W. Trucks, H. B. Schlegel, G. E. Scuseria, M. A. Robb, J. R. Cheeseman, G. Scalmani, V. Barone, G. A. Petersson, H. Nakatsuji, X. Li, M. Caricato, A. V. Marenich, J. Bloino, B. G. Janesko, R. Gomperts, B. Mennucci, H. P. Hratchian, J. V. Ortiz, A. F. Izmaylov, J. L. Sonnenberg, D. Williams-Young, F. Ding, F. Lipparini, F. Egidi, J. Goings, B. Peng, A. Petrone, T. Henderson, D. Ranasinghe, V. G. Zakrzewski, J. Gao, N. Rega, G. Zheng, W. Liang, M. Hada, M. Ehara, K. Toyota, R. Fukuda, J. Hasegawa, M. Ishida, T. Nakajima, Y. Honda, O. Kitao, H. Nakai, T. Vreven, K. Throssell, J. A. Montgomery, Jr.; , J. E. Peralta, F. Ogliaro, M. J. Bearpark, J. J. Heyd, E. N. Brothers, K. N. Kudin, V. N. Staroverov, T. A. Keith, R. Kobayashi, J. Normand, K. Raghavachari, A. P. Rendell, J. C. Burant, S. S. Iyengar, J. Tomasi, M. Cossi, J. M. Millam, M. Klene, C. Adamo, R. Cammi, J. W. Ochterski, R. L. Martin, K. Morokuma, O. Farkas, J. B. Foresman, D. J. Fox, Gaussian, Inc., Wallingford CT, **2016**.

(16) S. Grimme, S. Ehrlich, L. Goerigk, Effect of the Damping Function in Dispersion Corrected Density Functional Theory*, J. Comp. Chem.* **2011**, *32*, 1456–1465.

(17) A. V. Marenich, C. J. Cramer, D. G. Truhlar, Universal Solvation Model Based on Solute Electron Density and on a Continuum Model of the Solvent Defined by the Bulk Dielectric Constant and Atomic Surface Tensions*, J. Phys. Chem. B* **2009**, *113*, 6378–6396.

(18) M. Mammen, E. I. Shakhnovich, J. M. Deutch, G. M. Whitesides, Estimating the Entropic Cost of Self-Assembly of Multiparticle Hydrogen-Bonded Aggregates Based on the Cyanuric Acid·Melamine Lattice*, J. Org. Chem.* **1998**, *63*, 3821–3830.

(19) M. Besora, P. Vidossich, A. Lledós, G. Ujaque, F. Maseras, Calculation of Reaction Free Energies in Solution: A Comparison of Current Approaches*, J. Phys. Chem. A* **2018**, *122*, 1392–1399.

(20) Y. Liang, S. Liu, Y. Xia, Y. Li, Z.-X. Yu, Mechanism, Regioselectivity, and the Kinetics of Phosphine-Catalyzed [3+2] Cycloaddition Reactions of Allenoates and Electron-Deficient Alkenes*, Chem. Eur. J.* **2008**, *14*, 4361–4373.

(21) Z.-X. Yu, K. N. Houk, Intramolecular 1,3-Dipolar Ene Reactions of Nitrile Oxides Occur by Stepwise 1,1-Cycloaddition/Retro-Ene Mechanisms*, J. Am. Chem. Soc.* **2003**, *125*, 13825–13830.

(22) R. E. Plata, D. A. Singleton, A Case Study of the Mechanism of Alcohol-Mediated Morita Baylis–Hillman Reactions. The Importance of Experimental Observations*, J. Am. Chem. Soc.* **2015**, *137*, 3811–3826.
